# Comprehensive Proteoform Characterization of Plasma Complement Component C8αβγ by Hybrid Mass Spectrometry Approaches

**DOI:** 10.1007/s13361-018-1901-6

**Published:** 2018-03-12

**Authors:** Vojtech Franc, Jing Zhu, Albert J. R. Heck

**Affiliations:** 1 Biomolecular Mass Spectrometry and Proteomics, Bijvoet Center for Biomolecular Research and Utrecht Institute for Pharmaceutical Sciences, University of Utrecht, Padualaan 8, 3584 CH Utrecht, The Netherlands; 2 Netherlands Proteomics Center, Padualaan 8, 3584 CH Utrecht, The Netherlands

**Keywords:** Complement component C8, Membrane attack complex, Glycosylation, Plasma proteins, Native mass spectrometry, Ion-exchange chromatography, Glycopeptide-centric proteomics, *N*-glycosylation, *O*-glycosylation, *C*-glycosylation

## Abstract

The human complement hetero-trimeric C8αβγ (C8) protein assembly (~ 150 kDa) is an important component of the membrane attack complex (MAC). C8 initiates membrane penetration and coordinates MAC pore formation. Here, we charted in detail the structural micro-heterogeneity within C8, purified from human plasma, combining high-resolution native mass spectrometry and (glyco)peptide-centric proteomics. The intact C8 proteoform profile revealed at least ~ 20 co-occurring MS signals. Additionally, we employed ion exchange chromatography to separate purified C8 into four distinct fractions. Their native MS analysis revealed even more detailed structural micro-heterogeneity on C8. Subsequent peptide-centric analysis, by proteolytic digestion of C8 and LC-MS/MS, provided site-specific quantitative profiles of different types of C8 glycosylation. Combining all this data provides a detailed specification of co-occurring C8 proteoforms, including experimental evidence on N-glycosylation, C-mannosylation, and O-glycosylation. In addition to the known N-glycosylation sites, two more N-glycosylation sites were detected on C8. Additionally, we elucidated the stoichiometry of all C-mannosylation sites in all the thrombospondin-like (TSP) domains of C8α and C8β. Lastly, our data contain the first experimental evidence of O-linked glycans located on C8γ. Albeit low abundant, these O-glycans are the first PTMs ever detected on this subunit. By placing the observed PTMs in structural models of free C8 and C8 embedded in the MAC, it may be speculated that some of the newly identified modifications may play a role in the MAC formation.

**Electronic supplementary material:**

The online version of this article (10.1007/s13361-018-1901-6) contains supplementary material, which is available to authorized users.

**Graphical Abstract:**
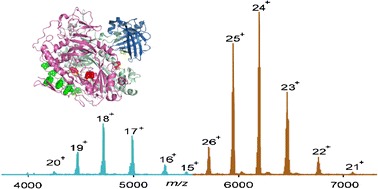
ᅟ

## Introduction

In humans, the complement system forms the first line of defense against microbial infections. Three major complement cascades (the classical, the alternative, and the lectin pathway) can initiate the terminal pathway. This process includes the formation of the membrane attack complex (MAC), consisting of the complement component C5, 6, 7, 8, and 9, an important innate immune effector that forms cytotoxic pores in the bacterial cell membrane. Although MAC assembly and its action have been extensively functionally and structurally investigated for many years, the molecular mechanism behind these processes and the role of protein post-translational modifications (PTMs) therein remain largely elusive. Recent progress in structural biology has provided several cryoEM maps on the MAC and new insights into the molecular architecture of this fascination assembly, albeit that PTMs are mostly not visible in such structural models [1].

**Figure 1:**
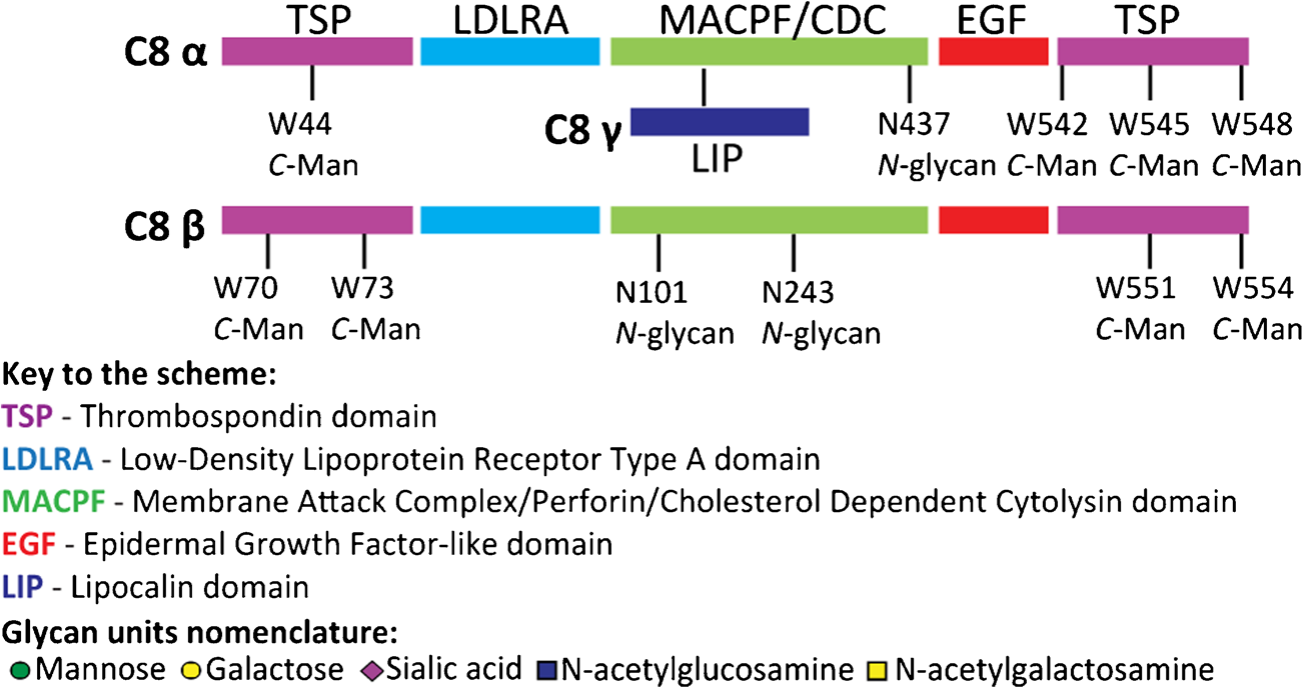
Schematic of domain composition and primary structure of C8αβγ. Previously reported sites of N-glycosylation [3, 14, 15] and *C*-mannosylation [16] are indicated. The used glycan nomenclature is depicted at the bottom

PTMs are well known to modulate protein function and physical-chemical properties of proteins [2]. Therefore, to fully understand the function of all MAC components in detail, it is important to systematically complement these high-resolution structural data with information on PTMs as provided by techniques such as high-resolution mass spectrometry (MS) and chromatography. Since all terminal complement proteins are glycoproteins [3], elucidating their detail glycosylation patterns is of particular interest. An emerging powerful analytical method to analyze protein proteoforms is high-resolution native MS. This method is primarily known for its use in the characterization of non-covalent protein assemblies [4, 5], but has more recently also been explored in the analysis of various glycoproteins and/or biotherapeutics [6, 7]. Recently, high-resolution native MS has been combined with peptide-centric proteomics for an in-depth analysis of PTMs on plasma or recombinant proteins [8, 9]. We proposed this hybrid MS strategy for the efficient and reliable analysis of biologically important (glyco)proteins, as has been first demonstrated by our analysis of complement component C9 [10]. Here, we extend this analysis to C8. C8 is a heterotrimeric complex (~ 150 kDa) comprising of the poly-peptide chains C8α, C8β, and C8γ and is incorporated into the membrane-bound assembly. C8 undergoes a conformational rearrangement in which the C8α subunit becomes the first component to penetrate the lipid bilayer [11]. All three subunits of C8 are synthesized in the liver [12]. C8 consists of a disulfide-linked αγ hetero dimer non-covalently bound to the β subunit. C8 subunits α (~ 64 kDa) and C8β (~ 64 kDa) are structurally highly homologous and contain several different domains including a low-density lipoprotein receptor class A repeat (LDLRA), a membrane attack complex/perforin (MACPF) domain, an epidermal growth factor (EGF)-like domain and N-terminal and C-terminal thrombospondin domains (TSP) (Fig. 1) [13]. Both, the α and β subunits have previously been reported to be N-glycosylated [3, 14, 15] and also modified by a less frequently occurring type of glycosylation: C-mannosylation [16]. The C8γ subunit (~ 22 kDa) belongs to the lipocalin family of small proteins, which has the common ability to bind small hydrophobic ligands [17]. Here, we report an in-depth analysis of the C8 complement component protein assembly purified from pooled normal human blood plasma using hybrid MS techniques combined with ion exchange chromatography. Our data show that chromatographic separation of C8 from human plasma allows for the isolation and subsequent MS detection of different C8 forms. This allows us to obtain a detailed view of the modifications co-occurring on each of the three C8 subunits. The identified PTMs are validated by peptide-centric analysis (LC-MS/MS). In addition to the previously reported modification sites on C8α and C8β, our data revealed also the structural composition of the detected N-glycans and revealed the occurrence of O-linked glycans in the C8γ subunit, providing the first experimental evidence of any modification on C8γ. We also detected some previously unreported low abundant N-glycans on the α and β subunit and revealed the stoichiometry of occupancy of various co-occurring C-mannosylation sites. Overall our findings bring detailed new insights into the structural composition of C8, an essential factor in complement activation and thus our immune response.

## Materials and Methods

### Chemicals and Materials

Complement component C8 (UniProt Code: P07357 (α), P07358 (β), P07360 (γ)) purified from pooled human blood plasma (several healthy donors) was acquired from Complement Technology, Inc. (Texas, USA). The sample was purified according to a reported standard protocol [18] (the certificate of analysis is attached in the Supporting information – S1). Dithiothreitol (DTT), iodoacetamide (IAA) and ammonium acetate (AMAC) were purchased from Sigma-Aldrich (Steinheim, Germany). Formic acid (FA) was from Merck (Darmstadt, Germany). Acetonitrile (ACN) was purchased from Biosolve (Valkenswaard, The Netherlands). POROS Oligo R3 50-μm particles were obtained from PerSeptive Biosystems (Framingham, MA, USA) and packed into GELoader pipette tips (Eppendorf, Hamburg, Germany). Sequencing grade trypsin was obtained from Promega (Madison, WI). Asp-N, PNGase F, and Sialidase were obtained from Roche (Indianapolis, USA).

### Sample Preparation for Native MS

Unprocessed protein solution in a phosphate buffer at pH 7.2, containing ~ 30–40 μg of C8, was buffer exchanged into 150 mM aqueous AMAC (pH 7.5) by ultrafiltration (vivaspin500, Sartorius Stedim Biotech, Germany) using a 10 kDa cutoff filter. The resulting protein concentration was measured by UV absorbance at 280 nm and adjusted to 2–3 μM prior to native MS analysis. The enzyme Sialidase was used to remove sialic acid residues from C8. PNGase F was used to cleave the *N*-glycans of C8 [19]. All samples were buffer exchanged into 150 mM AMAC (pH 7.2) prior to native MS measurements.

### Native MS Analysis

Samples were analyzed on a modified Exactive Plus Orbitrap instrument with extended mass range (EMR) (Thermo Fisher Scientific, Bremen) using a standard *m/z* range of 500–10,000, as described in detail previously [20]. The voltage offsets on the transport multi-poles and ion lenses were manually tuned to achieve optimal transmission of protein ions at elevated *m/z*. Nitrogen was used in the higher-energy collisional dissociation (HCD) cell at a gas pressure of 6–8 × 10^−10^ bar. MS parameters used: spray voltage 1.2–1.3 V, source fragmentation 30 V, source temperature 250 °C, collision energy 30 V, and resolution (at *m/z* 200) 30,000. The instrument was mass calibrated as described previously, using a solution of CsI [20].

### Native MS Data Analysis

The accurate masses of the observed C8 proteoforms were calculated manually averaging over all detected charge states of C8. For PTM composition analysis, data were processed manually and glycan structures were deduced based on known biosynthetic pathways. Average masses were used for the PTM assignments, including hexose (e.g., Glucose, Glc; mannose, Man; Galactose, Gal; 162.1424 Da), *N*-acetylhexosamine (e.g., GlcNAc or GalNAc; 203.1950 Da) and *N*-acetylneuraminic acid (NeuAc, 291.2579 Da). All used symbols and text nomenclature are according to recommendations of the Consortium for Functional Glycomics.

### Dual-Column Ion-Exchange Chromatography Separation of Purified C8

An Agilent 1290 Infinity HPLC system (Agilent Technologies, Waldbronn. Germany) consisting of a vacuum degasser, binary pump, refrigerated autosampler with 500-μL injector loop, thermostated two column compartment, auto collection fraction module and multi-wavelength detector, was used in this study. The dual-column set-up, comprising a tandem WAX-CAT (PolyWAX LP, 200 × 2.1 mm i.d., 5 μm, 1000 Å; PolyCAT A, 50 × 2.1 mm i.d., 5 μm, 1000 Å) two-stage set-up. All columns were obtained from PolyLC Inc. (Columbia, USA) [21]. The column compartment was cooled to 17 °C while the other bays were chilled to 4 °C minimize sample degradation. Mobile phase Buffer A consisted of 100 mM AMAC in water, and mobile phase Buffer B consisted of 2.5 M AMAC in water. A small amount (final concentration of 3 mM) of NaN_3_ was added to minimize microbial outgrowth to each solution, which was filtered using a 0.22 μm disposable membrane cartridge (Millipore) before use. Injections were typically 250 μg total protein per run. Elution was achieved using multi-step gradient, consisting of five transitions with increasing proportions of Buffer B: (step 1; equilibration) 0%B, 0–6 min; (step 2; salt gradient) 0–60%B, 6–42 min; (step 3; high salt rinse) 60–100%B, 42–60 min; (step 4; high salt wash) 100%B, 60–61 min; (step 5; restoration) 100–0%B. The flow rate was set to 800 μL min^−1^. The chromatograms were monitored at 280 nm and peak based fractions collected using an automated fraction collector.

### In-Solution Digestion for Peptide-Centric Glycoproteomics

Intact human C8 protein in PBS buffer (10 mM sodium phosphate, 145 mM NaCl, pH 7.3) at a concentration of 1 mg/ml was reduced with 5 mM DTT at 56 °C for 30 min and alkylated with 15 mM IAA at room temperature for 30 min in the dark. The excess of IAA was quenched by using 5 mM DTT. C8 was digested overnight with trypsin at an enzyme-to-protein-ratio of 1:100 (*w*/*w*) at 37 °C. Another C8 sample was digested for 4 h by using Asp-N at an enzyme to-protein-ratio of 1:75 (*w*/*w*) at 37 °C and the resulted peptide mixtures were further treated with trypsin (1:100; *w*/*w*) overnight at 37 °C. All proteolytic digests containing modified glycopeptides were desalted by GELoader tips filled with POROS Oligo R3 50 μm particles [22], dried and dissolved in 40 uL of 0.1% FA prior liquid chromatography (LC)-MS and MS/MS analysis.

### LC-MS/MS Analysis

All peptides (typically 300 fmol of C8 peptides) were separated and analyzed using an Agilent 1290 Infinity HPLC system (Agilent Technologies, Waldbronn. Germany) coupled on-line to an Orbitrap Fusion mass spectrometer (Thermo Fisher Scientific, Bremen, Germany). Reversed-phase separation was accomplished using a 100 μm inner diameter 2 cm trap column (in-house packed with ReproSil-Pur C18-AQ, 3 μm) (Dr. Maisch GmbH, Ammerbuch-Entringen, Germany) coupled to a 50 μm inner diameter 50 cm analytical column (in-house packed with Poroshell 120 EC-C18, 2.7 μm) (Agilent Technologies, Amstelveen, The Netherlands). Mobile-phase solvent A consisted of 0.1% FA in water, and mobile-phase solvent B consisted of 0.1% FA in ACN. The flow rate was set to 300 nL/min. A 45 min gradient was used as follows: 0–10 min, 100% solvent A; 10.1–35 min 10% solvent B; 35–38 min 45% solvent B; 38–40 min 100% solvent B; 40–45 min 100% solvent A. Nanospray was achieved using a coated fused silica emitter (New Objective, Cambridge, MA) (outer diameter, 360 μm; inner diameter, 20 μm; tip inner diameter, 10 μm) biased to 2 kV. The mass spectrometer was operated in positive ion mode and the spectra were acquired in the data-dependent acquisition mode. For the MS scans, the mass range was set from 300 to 2000 *m/z* at a resolution of 60,000 and the AGC target was set to 4 × 10^5^. For the MS/MS measurements, HCD and electron-transfer and higher-energy collision dissociation (EThcD) were used in the two LC-MS/MS runs for every sample. First, HCD was performed with two independent scan events. One scan event with a normalized collision energy of 15% and the other with 35%. The second LC-MS/MS run was performed using EThcD. A supplementary activation energy of 20% was used for EThcD. For the MS/MS scans the mass range was set from 100 to 2000 *m/z* and the resolution was set to 30,000; the AGC target was set to 5 × 10^5^; the precursor isolation width was 1.6 Da and the maximum injection time was set to 300 ms.

### LC-MS/MS Data Analysis

Raw data were interpreted by using the Byonic software suite (Protein Metrics Inc.) [23] and further validation of the key MS/MS spectra was manually checked. The following parameters were used for data searches: precursor ion mass tolerance, 10 ppm; product ion mass tolerance, 20 ppm; fixed modification, Cys carbamidomethyl; variable modification: Met oxidation, Trp Mannosylation, and both N- and O-glycosylation from mammalian glycan databases. A non-enzyme specificity search was chosen for all samples. The database used contained the C8 protein amino acid sequence (Uniprot Code: P02748). Profiling and relative quantification of PTM modified C8 peptides were achieved by use of the extracted ion chromatograms (XICs) from two independently processed C8 samples. The peptide mixtures were prepared with different combinations of proteolytic enzymes as described above (1. Trypsin; 2. AspN + Trypsin). Both samples were analyzed in two independent LC-MS/MS runs using EThcD and HCD respectively. Each peptide that contains PTM sites was normalized individually so that the sum of all its proteoform areas was set at 100%. The average peptide ratios from all measurements were taken as a final estimation of the abundance. The XICs were obtained using the software Thermo Proteome Discoverer 2.2.0.388. The glycan structures of each glycoform were manually annotated. Hereby reported glycan structures are depicted without the linkage type of glycan units since the acquired MS/MS patterns do not provide such information.

### Integrating Native MS and Peptide-Centric Proteomic Data

Reliability and completeness of the obtained proteoform profiles of C8 were assessed by an integrative approach combining the native MS data with the glycopeptide centric proteomics data. Details of this approach have been described in detail previously [9]. Briefly, in silico data construction of the “intact protein spectra” was performed based on the masses and relative abundances of all site-specific PTMs derived from the glycopeptide centric analysis. Subsequently, the constructed spectrum was compared to the experimental native MS spectra of C8. The similarity between the two independent data sets (Native MS spectra and constructed spectra based on glycopeptide centric data) was expressed by a Pearson correlation factor. All R scripts used for the spectra simulation are available at github (https://github.com/Yang0014/glycoNativeMS).

### I-TASSER Structural Modeling of C8

Detailed descriptions of I-TASSER can be found in Refs [24, 25]. Briefly, the structural monomer of poly-C9 (EMDB code: 3134) [26] was selected as a template for the structural remodeling of the soluble C8β (UniProt code: P07358) to its putative membrane form. Using Monte Carlo simulations, the template and regions modeled with ab initio methods are assembled into a large number of full length conformations. By clustering the conformations, cluster centroids are identified, and the final models are built by additional refinements of the cluster centroids. The models were eventually processed using PyMOL Molecular Graphic System.

## Results

### Native MS of the C8 Complement Assembly

**Figure 2:**
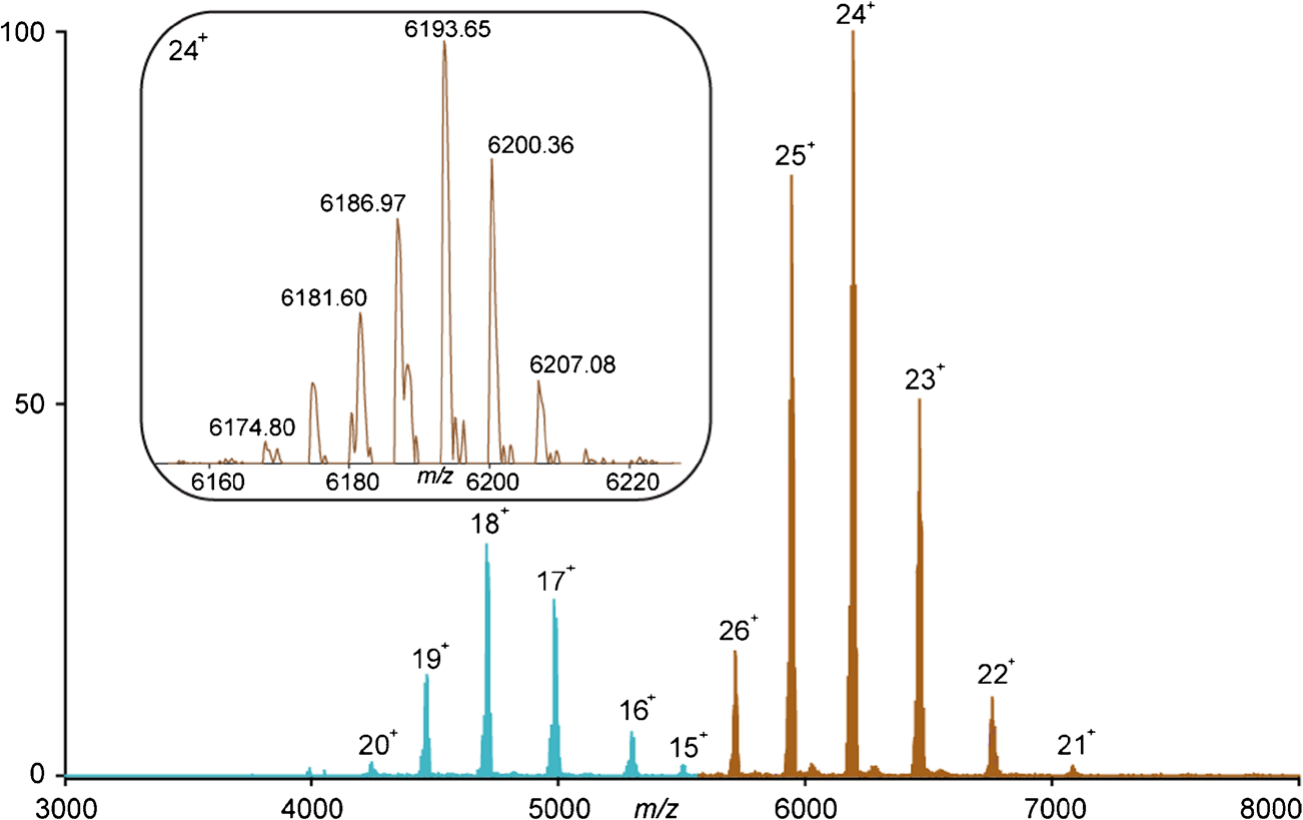
Full native ESI-MS spectrum of intact C8. Two major ion series were detected corresponding to C8αγ (blue) and C8αβγ (red). The charge states are indicated. The inset, zoomed in into the 24^+^ charged state of C8αβγ, reveals approximately 20 distinct ion signals

The main objective in this study was to characterize in detail human plasma-purified C8, including its chain composition and all PTMs they harbor. We started our investigation by acquiring high-resolution native ESI-MS spectra of plasma-purified C8 (Fig. 2). Somewhat surprising, the native MS spectra of C8 displayed two major ion series with different masses corresponding to the lower abundant heterodimer C8αγ and the more abundant heterotrimer C8αβγ. The first ion series contains at least six different charge states, ranging from [M + 15H]^15+^ to [M + 20H]^20+^ and the second ion series contains six charge states ranging from [M + 21H]^21+^ to [M + 26H]^26+^. The occurrence of the minor C8αγ has not been reported, but may represent an artifact of the purification. Therefore, we focused our attention here mostly to the fully assembled C8αβγ hetero-trimeric protein complex. Based on their distinguishable masses as shown in the inset in Fig. 2, taking a 1% cutoff in intensity, we could distinguish at least ~ 20 co-occurring MS signals for the fully assembled C8. To simplify the description, below we use the mass spectrum centered around the most intense charge state (24^+^), unless stated otherwise. From the gene sequences we can determine that the average mass of the amino acid protein backbone of C8 is 143,075.63 Da. In this mass calculation, we used the mass of the C8α, β and γ sequences lacking the *N*-terminal signal peptides and propeptide of the α and the β subunit and the N-terminal signal peptide of the γ subunit. The calculated mass was further adjusted by the mass shift induced by the 22 disulfide bonds present in C8 (8 in α, 12 in β, 1 in γ subunit and 1 inter disulfide bond between the α and γ subunits) and by the mass shift − 18.01 Da corresponding to the conversion of the N-terminal Gln on the γ subunit to pyroglutamic acid [27–29]. Determining the exact backbone mass allowed us to calculate a mass shift of 5547.97 Da induced by the PTMs on the most abundant peak in the 24^+^ charge state with *m/z* 6193.65 (148,623.60–143,075.63 = 5547.97 Da). Next, we enzymatically treated C8 attempting to the remove either all N-glycans or only the sialic acid moieties, which results in specific mass shifts that can be measured by native MS (Supplementary Fig. 1). We used all this information to calculate and predict the PTM composition of the most abundant C8 proteoform. For inducing the cleavage of *N*-glycosylations, we used PNGaseF, and sialidase for the specific removal of sialic acids. The incubation of C8 with PNGase F resulted in a removal of only one N-glycan (Supplementary Fig. 1b). The mass difference of 2206 Da between the most abundant intact C8 proteoform with *m/z* 6193.61 and the N-deglycosylated C8 with *m/z* 6101.94, indicated the attachment of an N-glycan with the carbohydrate composition of HexNAc_4_Hex_5_NeuAc_2_. The same mass shift was observed also on the most intense peak of the αγ dimer, indicating that it is likely present on α. Based on our calculation and from information in the literature, the most abundant C8 proteoform contains at least two N-glycans; however, even prolonged incubation of the sample with PNGase F did not lead to the complete removal of N-glycans. Incomplete enzymatic removal is a common problem when the deglycosylation reaction is performed under native conditions. Steric hindrance is usually the cause of incomplete release of N-glycans. Denaturing conditions may be beneficial to achieve complete removal [30]. The treatment of C8 with sialidase resulted in the removal of four sialic acid moieties from the most abundant C8 proteoform (Supplementary Fig. 1c). Based on the observed mass shifts, all four released sialic acids can only originate from two N-glycans with identical composition (2 x HexNAc_4_Hex_5_NeuAc_2_). Notably, the heterogeneity of the proteoform profile of the partially N-deglycosylated or desialylated C8 remained very similar, indicating that the main source of structural micro-heterogeneity is not originating from N-glycosylation.

The observed heterogeneity among the most abundant species in the C8 proteoform profile is represented by mass differences of 162 Da corresponding to Hex moieties. C8α and β are structurally and genetically related protein subunits, both belonging to the MAC protein family [31]. In addition to other structural similarities, both the α and β subunits contain two highly conserved TSP domains, harboring C-mannosylations [16, 32]. Previous C8 studies suggested four fully occupied C-mannosylation sites at each subunit. However, our native MS experiments on C8 strongly suggest structural heterogeneity caused by partial occupancy of the C-mannosylation sites. Our final calculation of the overall PTM composition of the most abundant C8 proteoform includes two N-glycans and seven mannoses (143,075.63 + 2 × 2206.01 + 7 × 162.14 = 148,622.63 Da) which in the end corresponds to an acceptable small mass error 6.52 ppm with respect to the observed C8 mass; 148,623.60 Da. These findings were further supported by our peptide-centric proteomic analyses, which are described below.

### Non-denaturing Ion Exchange Chromatography of C8 Enables the More Detailed Study of Individual Components

**Figure 3:**
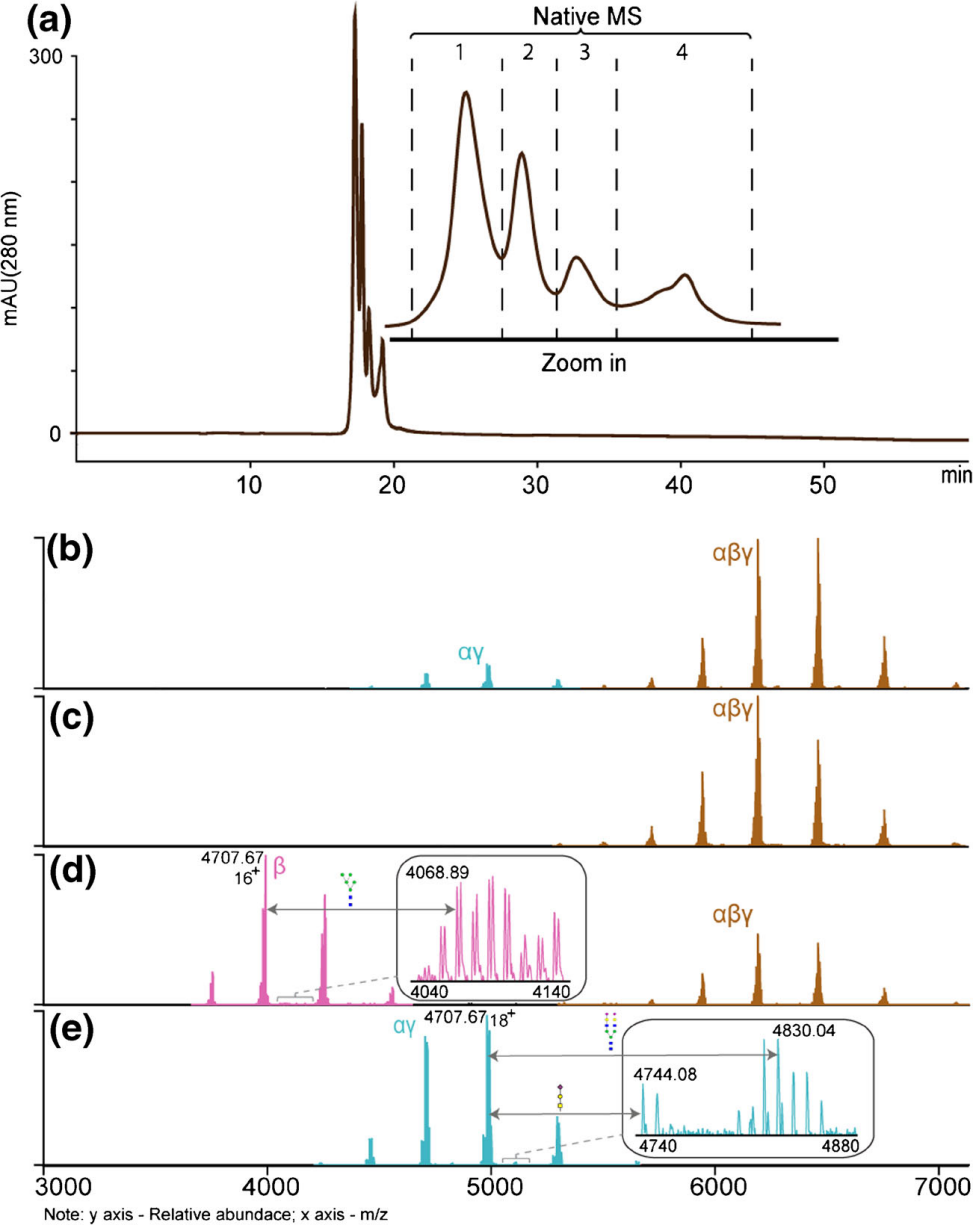
IEX fractionation of C8. The chromatographic fractionation of C8 was achieved with a tandem WAX-CAT dual-column set-up using an ammonium acetate gradient. The separation and fractionation was accomplished due to the instability of intact C8 at higher ionic strengths (**a**). The collected four fractions were directly analyzed by native MS (**b**, **c**, **d**, **e**) enabling the detection of low abundant ion series in C8β (**e**) and C8α (**d**) revealing novel C8 post-translational modifications

Interestingly, C8 subunits isolated either under denaturing or non-denaturing conditions retain their function and physically re-associate in solution to form native fully assembled C8 [18, 33]. An old procedure, reported in 1984, for purifying the αγ and β chains of C8 using a high ionic strength buffer [34] inspired us to use ion-exchange chromatography to separate and fractionate C8 subunits/sub-complexes and analyze these individually by high-resolution native MS. We loaded 200 μg of C8 purified from human plasma without any further pre-treatment on the dual column mixed-bed ion exchange chromatography system, comprising a weak cation exchange (CAT) column preceded by a weak anion exchange (WAX) column. This system performs a separation by using a gradient of AMAC in a concentration range from 100 mM to 2.5 M, which are conditions compatible with native MS. In Fig. 3a, a representative chromatographic profile is displayed, as recorded by UV-absorption (absorbance at 280 nm). Using this chromatographic set-up for C8 led to at least four distinct chromatographic peaks. The four collected fractions were subsequently concentrated and subjected to native MS, as displayed in Fig. 3b–e, respectively. Notably, by analyzing individual sub-complexes and subunits new proteoforms could be observed, not detectable or resolvable prior to chromatographic separation. Particularly, zooming in into the native MS spectrum of C8β (fraction 3) we observed that this subunit contains not one but two N-glycans. The observed mass differences correspond in this newly found N-glycosylation site to the presence of high mannose glycans (Fig. 3d). Similarly, the native MS spectrum of C8αγ (fraction 4) revealed low abundant proteoforms containing two N-glycans (the mass difference 2206) and likely also O-glycans (Fig. 3e). The mass difference of 656 Da between the peaks with *m/z* of 4707.67 and 4744.08 correspond to a glycan composition of HexNAc_1_Hex_1_NeuAc_1_. This may correspond to an O-glycan or alternatively a variability in the number of antennas on the N-glycans. These experiments together with the data obtained from the native MS measurements of intact, N-deglycosylated and desialylated C8 suggest, that the PTM profile of fully assembled C8 proteoforms includes, in total, two highly abundant and two low abundant N-glycosylation sites, possibly several low abundant O-glycans and several partially occupied C-mannosylated sites, making it a quite complicated glycoprotein assembly.

### Peptide-Centric LC-MS/MS Analysis of C8

To further validate our findings and calculations based on the native MS measurements, we next performed proteolytic digestion of C8 using different proteases and the resulting peptide mixtures were analyzed by LC-MS/MS. Data interpretation provided information about the site location, glycan type, composition and abundance of N-glycosylated, O-glycosylated and C-mannosylated proteoforms. Starting our description with C8α, MS/MS of the tryptic peptide with amino acid sequence ^425^GGSSGWSGGLAQNR^438^ clearly confirmed the composition of the high abundant N-glycans at N437 with the major glycoform corresponding to the N-glycan composition of HexNAc_4_Hex_5_NeuAc_2_. The MS/MS spectra revealed that the major N-glycan composition on this site is. The native MS measurements also revealed a novel low abundant N-glycosylation site on C8α, which we unfortunately did not detect in our LC-MS/MS data, probably due to its low abundance. Additionally, we detected peptides originating from the TSP domains; ^31^AATPAAVTCQLSNWSEWTDCFPCQDKK^57^ and ^538^ADGSWSCWSSWSVCR^552^. These peptides contain the sequence motif WXXW, which is known to be frequently C-mannosylated [16]. Our EThcD and HCD MS/MS spectra unequivocally confirmed that in C8α, W44, and W542 are fully occupied by C-mannosylation, whereas W47, W545, and W548 are only partially occupied.

A similar set of modifications was found on C8β. From the three potential N-glycosylation sites following the canonical N-X-S/T motif, two were found to be occupied. Interpretation of the LC-MS/MS spectra of the glycopeptides with the sequences ^86^YAYLLQPSQFHGEPCNFSDKEVEDCVTNRPCR^117^ and ^233^EYESYSDFERNVTEK^247^ revealed high mannose N-glycans with low occupancy on N101 and biantennary complex N-glycans on N243. Attachment of C-mannoses was confirmed by fragmentation of the peptides ^55^SVDVTLMPIDCELSSWSSWTTCDPCQK^81^ and ^541^NTPIDGKWNCWSNWSSCSGR^560^. In C8β W70 and W551 are fully occupied and W73, W548, W554 are partially modified. Furthermore, we identified and quantified two distinct positional isomers of the peptide ^425^NTPIDGKWNCWSNWSSCSGR^425^ modified by two Man either on W548 and W551 or W551 and W554 (Supplementary Fig. 2). Finally, C8γ was found to be partially O-glycosylated close to its N-terminus. EThcD and HCD MS/MS spectra of the O-glycopeptides with amino acid sequence ^28^RPASPISTIQPK^39^ exposed the structural composition of the attached O-glycans. These were HexNAc_1_, HexNAc_1_Hex_1_ and HexNAc_1_Hex_1_NeuAc_1_ attached to T35. All MS/MS spectra supporting these modifications can be found in the Supplementary data – S4.

### Integration of Native MS and Peptide-Centric Data

**Figure 4:**
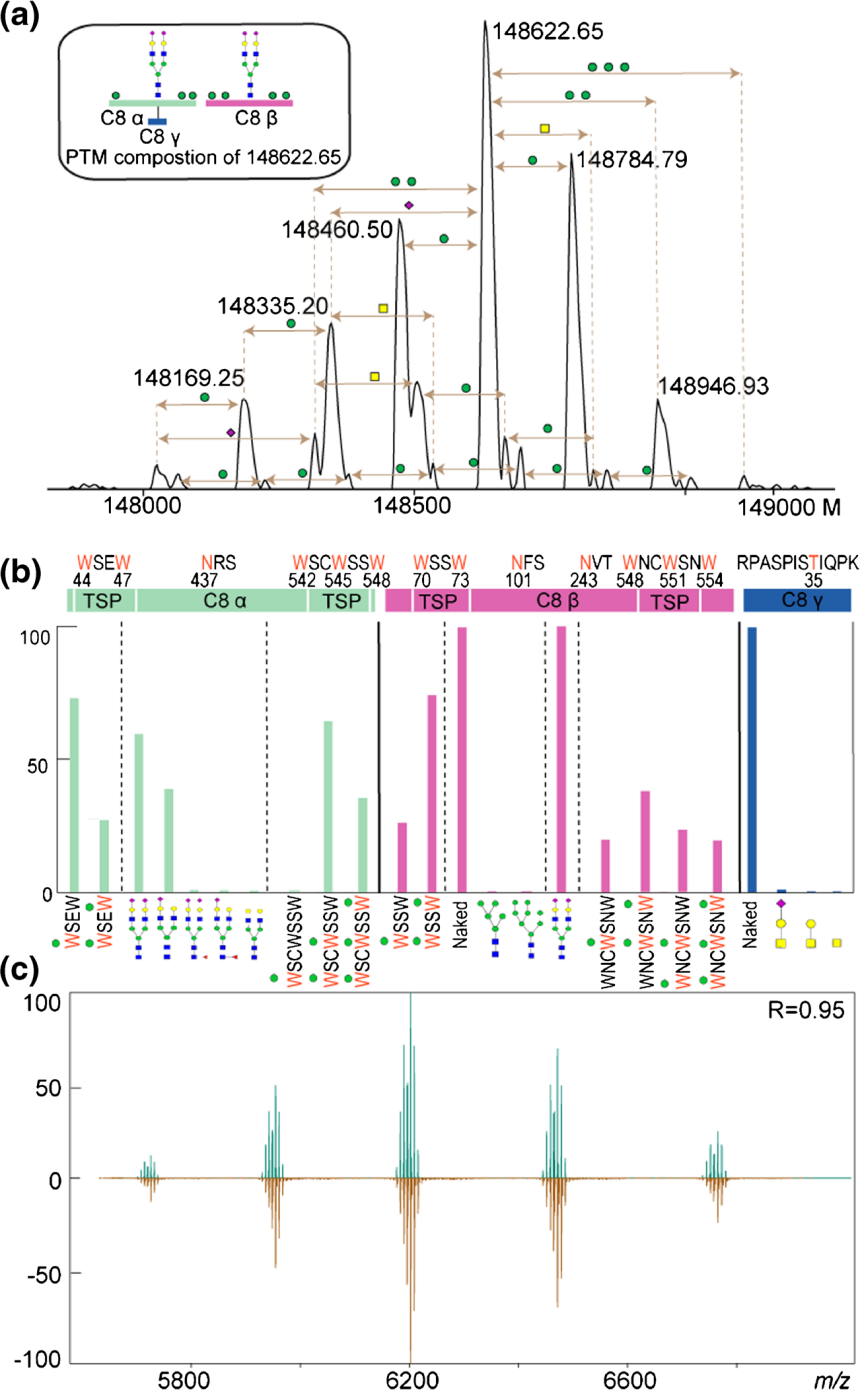
Overview of PTM assignments on C8. (**a**) Zero charge deconvoluted native MS spectrum of C8. The overall PTM composition of the most abundant proteoform with 148,622.65 Da was deduced based on the observed mass differences and mass shifts in MS spectra of PNGase F and sialidase treated C8 samples. Mass differences among the peaks match with specific glycan moieties. (**b**) Relative abundances of peptide proteoforms, as estimated from their corresponding extracted ion chromatograms (XICs). Each PTM modified peptide was normalized individually so that the sum of all proteoforms was set to 100%. For clarity, only the parts of the peptide sequence carrying the PTMs are shown below the graph. (**c**) a comparison of the intact C8 native MS spectrum (dark goldenrod) with the in silico reconstructed spectrum (sea green) based on the quantitative peptide-centric proteomics data. The correlation between these two spectra is high (*R* = 0.95)

Having both the native MS data and peptide-centric data on C8 and it subunits available, we cross- validated all data to come to a comprehensive description of the proteoform profile of C8 (Fig. 4). Figure 4a describes all annotated C8 proteoforms in the zero charge deconvoluted native spectra of C8αβγ. In Fig. 4b, all observed modified sites are displayed, their PTM composition and occupancy as extracted from the peptide-centric LC MS/MS data (Fig. 4b, Supplementary Table 1). The confirmed N-glycosylation site N437 on C8α is fully occupied by complex biantennary N-glycans with sialylated and non-sialylated antennas (the occupancy with two sialic acids is 59.5%, with one sialic acid 38.7% and zero sialic acids 0.3%). These N-glycan structures were also found to be core fucosylated, albeit to a minor content (< 1%). An N-glycan with the same structural composition and two sialic acids on the antennas fully occupies the N243 site on C8β. The relative abundance of the high mannose modified glycopeptides spanning the second N-glycosylation site at N101 on C8β is low. Both detected high mannose N-glycans with the composition of HexNAc_2_Hex_5_ and HexNAc_2_Hex_8_ have the relative abundance less than 0.5%. The identification of this novel site is of interest, but its low abundance will make it challenging to determine its potential function. C-mannosylation contributes most to the observed C8 heterogeneity with a variable occupancy of W residues by Man in the sequence motifs WXXW and WXXWXXW. The first tryptophan (W44) located at the N-terminal TSP domain of C8α is fully occupied, while the second site (W47) is only partially occupied (27%). C8β containing a similar sequence motif in its N-terminal TSP domain harbors mannoses on W70 (100%) and W73 (74%). The TSP domains at the C-terminus of C8α and C8β contain a WXXWXXW motif. The first tryptophan in the motif at C8α (W542) is fully occupied by Man, while the analogous site (W548) at C8β is only partially occupied (57.1%). The second tryptophan in the sequence is almost fully occupied by Man in C8α (W545) and in all cases also on C8β (W551). Finally, the third C-mannosylation site is occupied by Man in 35.4% in C8α (W548) and in 42.5% in C8β (W554). The O-glycosylated peptide detected in C8γ is mostly non-modified (T35) with all its O-glycosylated forms contributing less than 0.5% to the ion signals.

Next, we made a correlative comparison between the native MS spectrum of the intact fully assembled C8 with an in silico constructed MS spectrum based on all the quantitative information we gathered from the LC-MS/MS peptide centric data (Fig. 4c). All C8 species predicted from the peptide-centric data were filtered by taking 1% cutoff in relative intensity of the peaks in the experimental native spectrum and mass deviations were manually checked. This validation process resulted in a list containing 14 distinct C8 variants and covered most of the detected signals of the C8 proteoforms (Supplementary Table 2). This comparison reveals a high degree of consistency between our native MS and peptide-centric MS approach (*R* = 0.95), indicating that we have nearly annotated all proteoforms detected in the native MS spectrum. The unmatched low abundant ion signals mostly correspond to adducts bearing Na^+^ and/or K^+^ ions, which are frequently observed in the ESI ionization process. Some of the peaks in the constructed spectrum show a different intensity compare to the experimental native MS spectrum. This is caused by the fact that labile PTMs are easily lost during the peptide-centric LC-MS/MS analysis [35–37] and ionization at the peptide level is more biased when compared to the native intact protein measurements. Although we could explain most of the ion signals observed in the native MS spectra originating from the fully assembled C8, this analysis did not cover some of the lower abundant modifications that we only detected on the individual sub-complexes and proteins following the fractionation of C8 sub-complexes and subunits by IEX. Therefore, these low abundant species are not included in the final list of the validated C8 proteoforms. These include the C8 proteoforms containing the novel albeit low abundant N-glycosylation sites on C8α and C8β.

## Discussion

C8 is a protein assembly consisting of three distinct subunits. C8 possesses an atypical structure and unusual physicochemical properties relative to other related complement proteins and even to most plasma proteins in general. Due to a complicated assembly process and secretion pathway of C8, it is not fully clear, which structural variants of C8 are present in circulation and what is their biological relevance. The proposed biosynthetic mechanism suggests that precursors of C8αγ and β are likely associated intracellularly, and the pre-mature C8 complex is converted to its final form by terminal glycosylation reactions within the Golgi complex [12]. Mature C8 is then thought to be secreted intact. Alternatively, precursors of C8αγ and β could be processed independently or associated with their mature counterparts. Since less β subunit is synthesized in the liver compared to the α and γ subunits, independent secretion of C8β is not expected under normal conditions. However, independent secretion of αγ hetero-dimers and the β subunit may still be possible, especially in cases of hereditary C8 deficiency, wherein the αγ dimer and/or β subunit are found in their non-bound forms [38, 39]. Our native MS analysis of the Complement component C8 purified from pooled plasma hints at the co-existence of free C8αγ and fully assembled C8 in the sample solution. Up to now, no reports exist on the presence of free C8αγ dimers in normal plasma. In our analysis, we work with relatively high concentrations of C8 (~ 1 mg/mL), which is approximately 20× higher than the physiological concentration in normal human plasma. Therefore, we cannot exclude whether the C8αγ dimer is actually present in human plasma or whether it is an artifact of the purification process, or some product of dynamic assembly and disassembly between the C8 subunits. For that reason, although we find it an interesting finding, we focused our analysis on the proteoforms from the fully assembled C8 complex.

As with many other human plasma proteins, the C8 complex is synthetized in the liver. As a consequence, C8 is expected to contain mostly complex biantennary N-glycans with varying degrees of sialylation. The primary structures of C8α and β contain two and three potential N-glycosylation sites, respectively. Two of them (N437 on α and N243 on β) were known to harbor complex N-glycans, but with a lower level of sialylation compared to the other complement components. Additionally, C8β was also found to attach bisected glycan structures [3]. In comparison to these previous reports, we did not observe any abundant bisected structures and all detected N-glycopeptides attaching biantennary N-glycans showed relatively homogenous structures containing mostly sialylated antennas. These minor discrepancies may be explained by a variability between analyzed samples and/or more advanced technological possibilities in the current study. Beside the two known N-glycosylation sites, the native MS measurements of the IEX fractions revealed some low abundant glycoforms of C8β (fraction 3) and C8αγ (fraction 4) both containing additional N-glycans. Although the β chain was predicted to harbor an N-glycan on N101 based on the sequence, no experimental evidence had been reported previously. Our data show that N101 can be indeed glycosylated; however, its occupancy is low. Interestingly, we detected only the presence of high mannose N-glycans, suggesting a low accessibility of this potential N-glycosylation site for the glycosylation machinery in the Golgi. The second low abundant N-glycan detected on C8αγ is likely located on C8α, since the γ subunit does not possess any potential N-glycosylation site. C8α contains a potential N-glycosylation site (N43) in the first TSP domain that overlaps with a sequon for C-mannosylation. Previous reports dealing with the C-mannosylation of C8 protein speculate that C-mannosylation precedes the addition of N-glycans on this site [3]. Although the mass differences observed in the native MS spectra of the αγ dimer clearly revealed the presence of unreported complex biantennary N-glycans, we did not detect any N-glycopeptide confirming the position of this N-glycan probably due to its very low abundance. Nevertheless, if N43 was modified by this N-glycan, we could speculate that the proposed function of the Man at W44 precluding the attachment of N-glycans may be actually correct. The question remains if that could be possible, since W44 is fully C-mannosylated and therefore the N-glycan would have to co-exist next to this Man. Another option is that the detected N-glycan is located on another potential, non-canonical, N-glycosylation site.

C-mannosylation is a significant feature of the MAC. It has been hypothesized to play an important role in adhesion of the terminal complement proteins to each other and in the assembly of the MAC [40, 41]. Regarding to C-mannosylation of C8 in particular, we identified that occupancy of the potential C-mannosylation sequons in TSP domains of α and β subunit is the major source of micro-heterogeneity in C8. This is in contradiction with older data, which suggested only completely modified or non-modified W on C8 [16]. Interestingly, we also identified positional isomers on C-terminal TSP of C8β. Our peptide-centric data on C8β show that the second W in the WXXWXXW motif is always modified while the second Man is added either on the first or the third W without apparent preference. We did not observe this on analogical C-man sites on the C-terminal TSP of C8α, where the first and the second W are clearly preferred to be modified compare to the third W in the motif. This may support hypothesis that the signal for C-mannosylation could be formed apart from the WXXW motif by the three-dimensional structure of a protein [32]. Since mechanistic insights about C-mannosylation have not been clarified, our results provide another important piece of information contributing to the discussion about this quite rare modification.

The last modification detected in our work was found on C8γ. There are several possible functions of C8γ described; however, a more precise biological role of C8γ in the complement system remains rather elusive. Here, we provide the first experimental evidence for O-glycans located on T35. Although detected O-glycosylated peptides showed very low abundance which is typically caused by weak ionization response of O-glycopeptides in general [35], the native MS profile of C8 clearly revealed proteoforms containing O-glycans with more than 2% of relative peak intensity. It could be that this modification is present only in parts of the population requiring analysis of C8 from individuals to determine a possible functional significance.

More generically, our analysis was performed on C8 purified from pooled plasma originating from several healthy donors. It may therefore also be of interest to investigate whether the occupancies we here report of all PTMs are equal of different in each individual. Such studies come within reach if more efficient purification methods would become available, and the analysis of individual proteoform profiles can be done more automatically. Our full analysis reported here would still take a week per individual.

**Figure 5:**
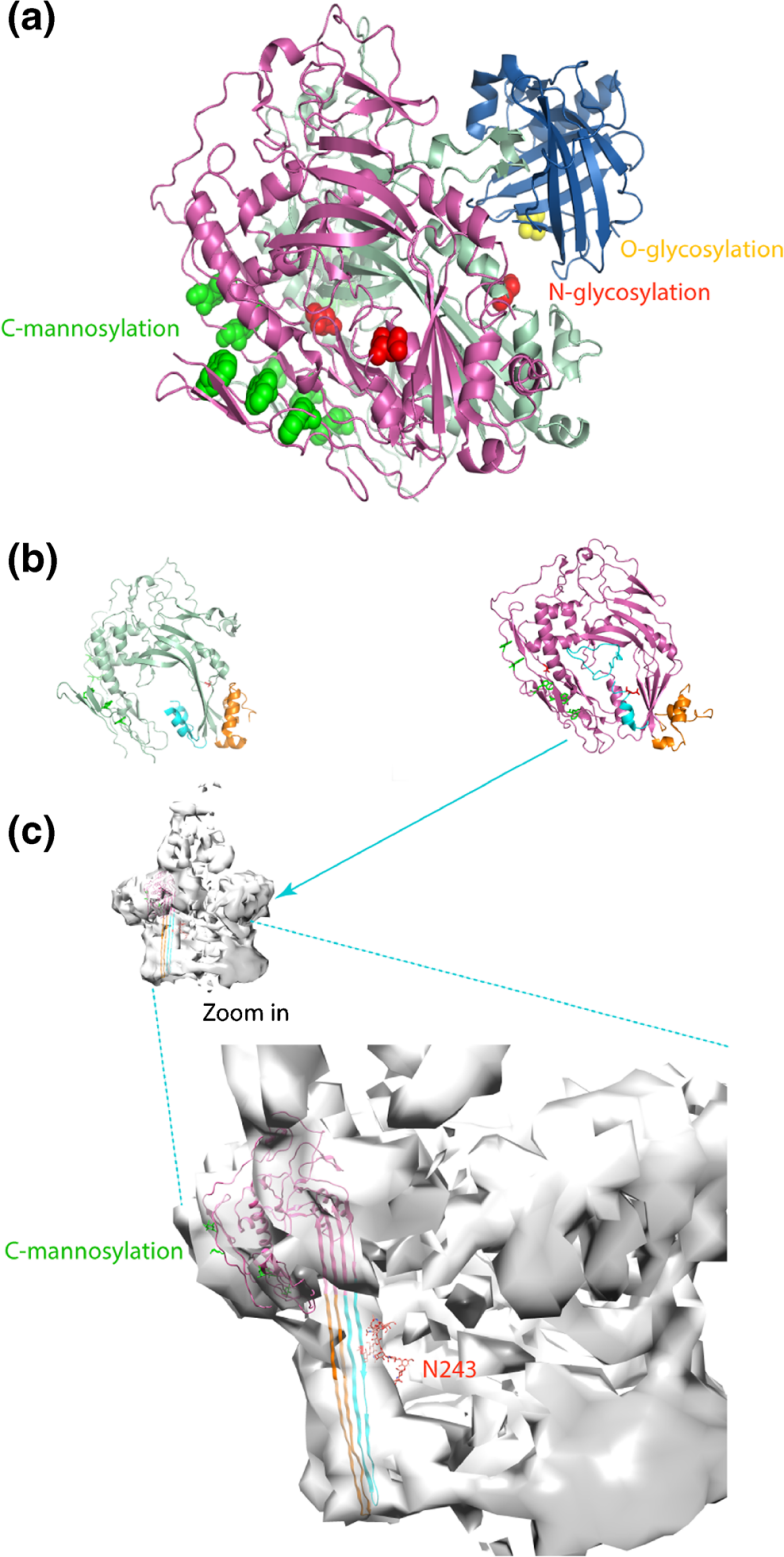
Structural models of C8 [28] (**a**) structural model of soluble fully assembled C8 consisting of α (vista blue), β (magenta), and γ (dark blue) (PDB code: 3OJY) with the sites of the detected PTMs indicated. The locations of C-mannosylation are highlighted with green spheres, N-glycosylation as red and O-glycosylation as yellow. (**b**) Structural models of soluble C8α and Cβ with the transmembrane β-hairpin segments (TMH) highlighted. The two segments are colored in cyan (TMH1) and orange (TMH2). (**c**) When incorporated into the MAC, the TMH regions refold into long β hairpins that span the membrane. N-glycosylation on C8β at N243 is in close proximity to the transmembrane segment and may therefore affect pore formation. The structural monomer of poly-C9 (EMDB code: 3134) [26] was chosen as template for C8β using I-Tasser [24] and models were processed using PyMOL molecular graphic system [43]

Although our data not directly reveal structure-function relationships in between the observed glycosylations and MAC and pore formation, it is intriguing to speculate their potential role in these processes based on their localization. Mapping all identified PTMs on structural models of C8 may hint at their putative roles (Fig. 5). C-mannosylation on C8α and C8β takes place at the external area of the assembled MAC, and has been suggested to play a role in the MAC assembly process. The detailed information about the stoichiometry of C-mannosylation on C8, which we provide here, can contribute to better understanding of this modification and potentially its function. Interestingly, the C8β N243 glycosylation is in the C8β MACPF domain. These MACPF domains undergo a large conformational change when the soluble C8 is incorporated into the MAC [42]. Especially, the transmembrane β-hairpin (TMH) segments play a direct role in MAC membrane penetration.

The applied complementary mass-spectrometry based methods herein represent powerful tools for the unbiased in-depth analysis of C8 but also other plasma glycoproteins. In the near future we can foresee that we should be able to analyze glycoproteoform profile of individual proteins, isolated from individual personalized body fluids, such as blood, serum and urine. Such proteoform profiles may represent a new level of analysis in biomarker discovery.

## Electronic Supplementary Material

ESM 1 (DOCX 122 kb)

ESM 2 (PDF 197 kb)

ESM 3 (XLSX 23 kb)

ESM 4 (PDF 1782 kb)

ESM 5 (XLSX 12 kb)

## **Abbreviations:**

ACN: Acetonitrile
AMAC: Ammonium acetate
DTT: Dithiothreitol
EGF: Epidermal growth factor
EMR: Extended mass range
ESI: Electrospray ionization
EThcD: Electron-transfer and higher-energy collision dissociation
FDR: False discovery rate
HCD: Higher-energy collisional dissociation
IAA: Iodoacetamide
LC: Liquid chromatography
LDLRA: Low-density lipoprotein receptor class A repeat
MAC: Membrane attack complex
MS: Mass spectrometry
MS/MS: Tandem mass spectrometry
PTM: Post-translational modification
SDS-PAGE: Sodium dodecyl sulfate polyacrylamide gel electrophoresis
TFA: Trifluoroacetic acid
TMH: Transmembrane β-hairpin
TSP: Thrombospondin
XIC: Extracted ion chromatogram
