# Supplementary material for: Comprehensive Proteoform Characterization of Plasma Complement Component C8αβγ by Hybrid Mass Spectrometry Approaches
Source: J Am Soc Mass Spectrom. 2018 Mar 12;29(6):1099–110. doi: 10.1007/s13361-018-1901-6 (PMC6003997; doi:10.1007/s13361-018-1901-6)
Supplement: Supplementary file 1 — (DOCX 122 kb) [file 13361_2018_1901_MOESM1_ESM.docx]

**Supporting Information for Publication**

Comprehensive proteoform characterization of the human plasma Complement component C8 by hybrid mass spectrometry approaches

Vojtech Franc^1,2#^, Jing Zhu^1,2#^, and Albert J.R. Heck^1,2*^

^1^ Biomolecular Mass Spectrometry and Proteomics, Bijvoet Center for Biomolecular Research and Utrecht Institute for Pharmaceutical Sciences, University of Utrecht,

Padualaan 8, 3584 CH Utrecht, The Netherlands

^2^ Netherlands Proteomics Center, Padualaan 8, 3584 CH Utrecht, The Netherlands

Correspondence: Albert Heck, [a.j.r.heck@uu.nl](mailto:a.j.r.heck@uu.nl)

# contributed equally

**S1 – Supplementary document** - the certificate of analysis of the purified C8 sample

(Franc_et_al_S1_Supplementary document.pdf)

**S2 – Supplementary figure 1** – Native MS spectra of intact, N-deglycosylated and desialylated C8

**S3 – Supplementary figure 2** – XIC of positional isomers of C-mannosylated peptide derived from C8β and their MS/MS spectra.

**S4 – Supplementary data** – MS/MS spectra of all PTM modified peptides derived from proteolytic digestion of C8

(Franc_et_al_S4_Supplementary data.pdf)

**S5 – Supplementary Table 1** – Peptide-centric proteomic data; Site-specific quantification of PTMs on C8 based on peptide data

(Franc_et_al_S5_Suplementary Table1.xlsx)

**S6 – Supplementary Table 2** – Native MS data; list of validated C8 proteoforms

(Franc_et_al_S6_Suplementary Table2.xlsx)

FIGURE LEGENDS FOR SUPPORTING INFORMATION

**Supplementary Figure 1 – S2**

Zoom in on the 24^+^ charged state of the full native ESI-MS spectrum of the intact C8 sprayed from aqueous ammonium acetate (a). In (b) alike spectrum of C8 treated with PNGase F resulted on a removal of one N-glycan from the most abundant C8 proteoform. In (c) C8 was enzymatically desialylated which released four sialic acids from the most abundant C8 proteoform. The differences in mass between C8 proteoforms in the unprocessed and treated samples allows for the deduction of the PTM composition of these most abundant C8 proteoforms.

**Supplementary Figure 2 – S3**

XIC of the C-mannosylated peptide with amino acid sequence ^425^NTPIDGKWNCWSNWSSCSGR^425^ (a). MS/MS spectra clearly reveals that the peptide eluting in RT 32.24 attaches Man on W551 and W554 (b) while the peptide eluting in RT 32.60 attaches Man on W548 and W551 (c).


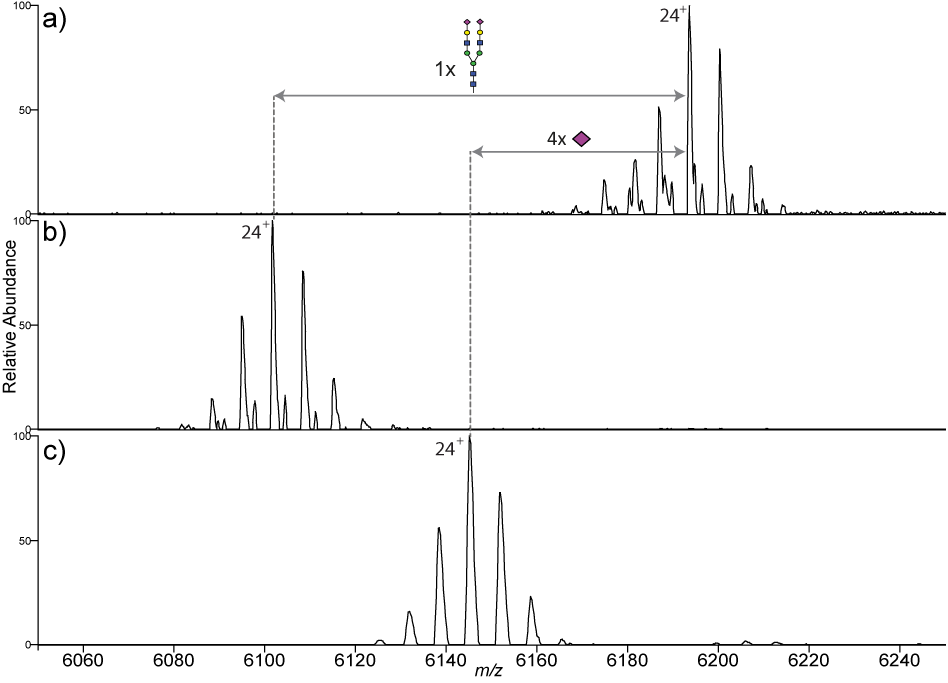


Supplementary Figure 1


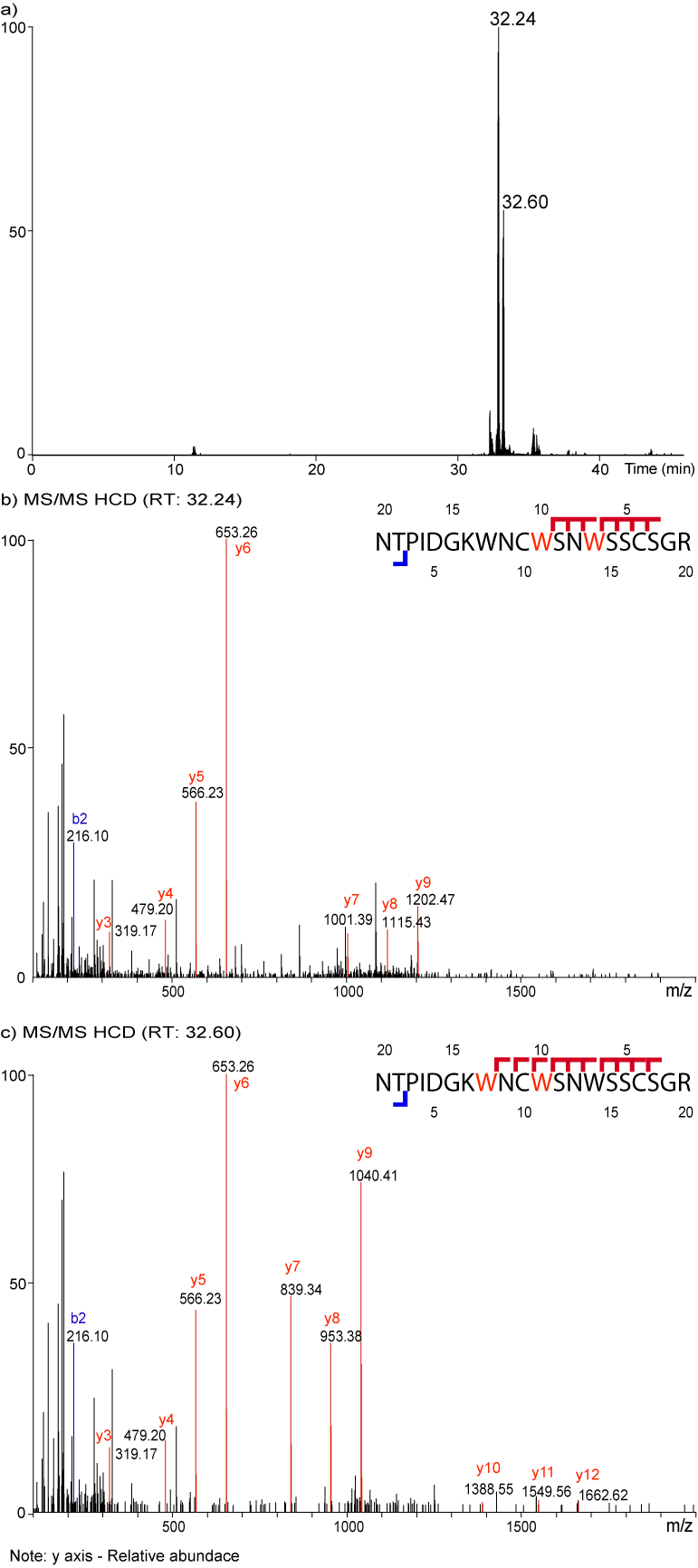


Supplementary Figure 2
