# Supplementary material for: Comprehensive Proteoform Characterization of Plasma Complement Component C8αβγ by Hybrid Mass Spectrometry Approaches
Source: J Am Soc Mass Spectrom. 2018 Mar 12;29(6):1099–110. doi: 10.1007/s13361-018-1901-6 (PMC6003997; doi:10.1007/s13361-018-1901-6)
Supplement: Supplementary file 2 — (PDF 197 kb) [file 13361_2018_1901_MOESM2_ESM.pdf]

CERTIFICATE OF ANALYSIS

Complement Technology, Inc.
4801 Troup Hwy, Suite 701
Tyler, Texas 75703, USA

Product: **C8 Protein**
Catalog # **A125 Lot # 127**
Exp. Date: 11/24/2019

Description: C8 Purified Human Complement Protein

Specifications	Limits	Results
PROTEIN CONCENTRATION	0.95 – 1.2 mg/mL C8 has an extinction coefficient of $E^{1\%}/_{280nm}$ = 14.9	1.03 mg/ml
FILL VOLUME	0.250 – 0.275 mL	0.270 mL
BUFFER	Phosphate buffered saline, pH 7.2	Phosphate buffered saline, pH 7.2
PRESERVATIVE	None, filtered through a 0.22 µm pore size filter.	None, filtered through a 0.22 µm pore size filter.
PURITY	> 85% by SDS PAGE	> 95%
FUNCTIONAL ACTIVITY	Titer >150,000 C8H50/mg	401,000 Units/mg
	>70% of C8 activity in NHS on a mg/mg basis.	100%
PRENSENCE OF OTHER FUNCTIONALLY ACTIVE COMPLEMENT COMPONENTS	≤ Trace amounts (≤0.1%) of functionally active C5, C6, C7, or C9	Conforms
IMMUNOCHEMISTRY (DILUTION OUCHTERLONIES)	≤ trace amounts of IgG, IgA, IgM, albumin, C3, C4, Factor B, Factor H or ceruloplasmin	Conforms
HUMAN SERUM/PLASMA STARTING MATERIAL		
HBsAg	Negative	Negative
ANTI-HBc	Negative	Negative
ANTI-HIV I & 2 Plus O	Negative	Negative
ANTI-HCV	Negative	Negative
ANTI-Syphilis	Negative	Negative
HCV by NAT	Negative	Negative
HIV by NAT	Negative	Negative
HTLV 1 and 2	Negative	Negative
West Nile Virus (WNV)	Negative	Negative

Store at -70°C or below.
Avoid Repeated Freeze/Thaw

FOR RESEARCH USE ONLY - NOT FOR HUMAN OR DRUG USE

Jerry Langburn
Signature of Analyst

11/25/2014
Date of Analysis

Phone: 1-903-581-8284 FAX: 1-903-581-0491 Email: contactCTI@aol.com
Web Site: www.ComplementTech.com
