# Supplementary material for: Comprehensive Proteoform Characterization of Plasma Complement Component C8αβγ by Hybrid Mass Spectrometry Approaches
Source: J Am Soc Mass Spectrom. 2018 Mar 12;29(6):1099–110. doi: 10.1007/s13361-018-1901-6 (PMC6003997; doi:10.1007/s13361-018-1901-6)
Supplement: Supplementary file 4 — (PDF 1782 kb) [file 13361_2018_1901_MOESM4_ESM.pdf]

C8 alpha subunit: MS/MS spectra of all PTM modified peptides

EThcD spectra

Glycosylation Site

TSR1_WSEW	AATPAAVT*C*QLSN*W*SEWTD*C*FP*C*QDKK<br>AATPAAVT*C*QLSN*W*SE*W*TD*C*FP*C*QDKK
TSR2_WSCWSSW	ADGS*W*S*C*WSSWSV*C*R<br>ADGS*W*S*C**W*SSWSV*C*R<br>ADGS*W*S*C**W*SS*W*SV*C*R
N437	VRGGSSGWSGGLAQ*N*[+2204.8]R<br>VRGGSSGWSGGLAQ*N*[+1914.8]R<br>VRGGSSGWSGGLAQ*N*[+2350.8]R<br>VRGGSSGWSGGLAQ*N*[+2059.7]R<br>VRGGSSGWSGGLAQ*N*[+1622.6]R

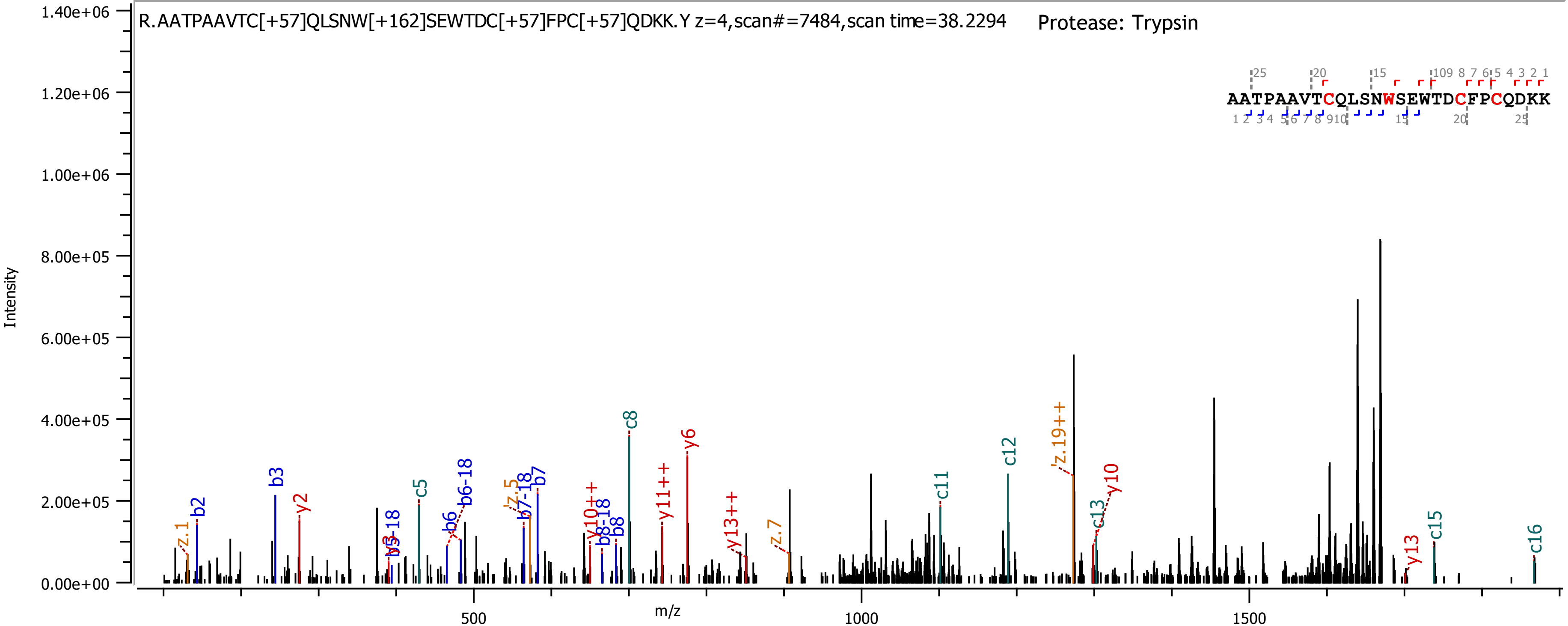
R.AATPAAVTC[+57]QLSNW[+162]SEWTDC[+57]FPC[+57]QDKK.Y z=4,scan#=7484,scan time=38.2294
Protease: Trypsin
AATPAAVTCQLSNWSEWTDCFPCQDKK
Intensity
m/z
0.00e+00
2.00e+05
4.00e+05
6.00e+05
8.00e+05
1.00e+06
1.20e+06
1.40e+06
500
1000
1500
z.1
b2
b3
y2
b3
b5-18
c5
b6
b6-18
z.5
b7-18
b7
y10++
b8-18
b8
c8
y11++
y6
y13++
z.7
c11
c12
z.19++
c13
y10
y13
c15
c16

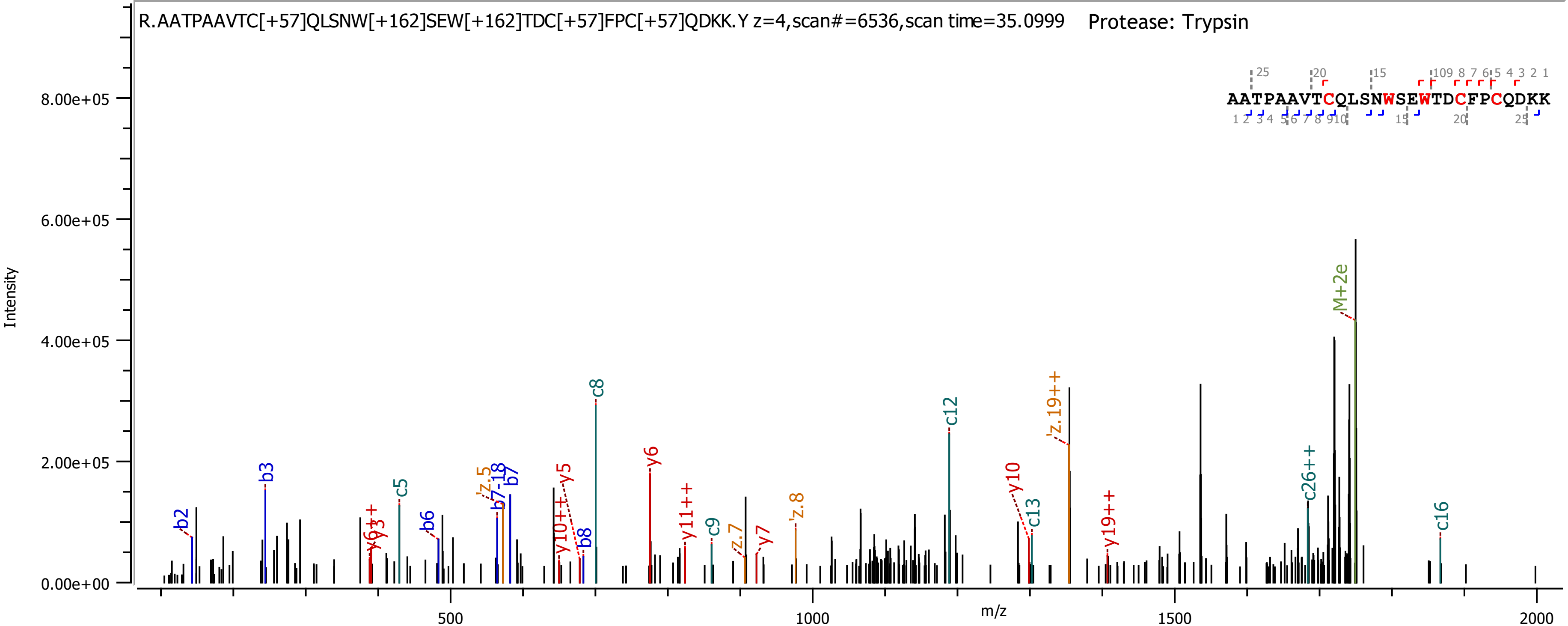
R.AATPAAVTC[+57]QLSNW[+162]SEW[+162]TDC[+57]FPC[+57]QDKK.Y z=4,scan#=6536,scan time=35.0999
Protease: Trypsin
AATPAAVTCQLSNWSEWTDCFPCQDKK
Intensity
m/z
8.00e+05
6.00e+05
4.00e+05
2.00e+05
0.00e+00
500
1000
1500
2000
M+2e
c8
c12
'z.19++
c26++
b3
c5
'z.5
b7-18
b7
y5
y6
y10
c13
c16
b2
b6
y6++
y3
y10++
b8
y11++
c9
z.7
y7
'z.8
y19++

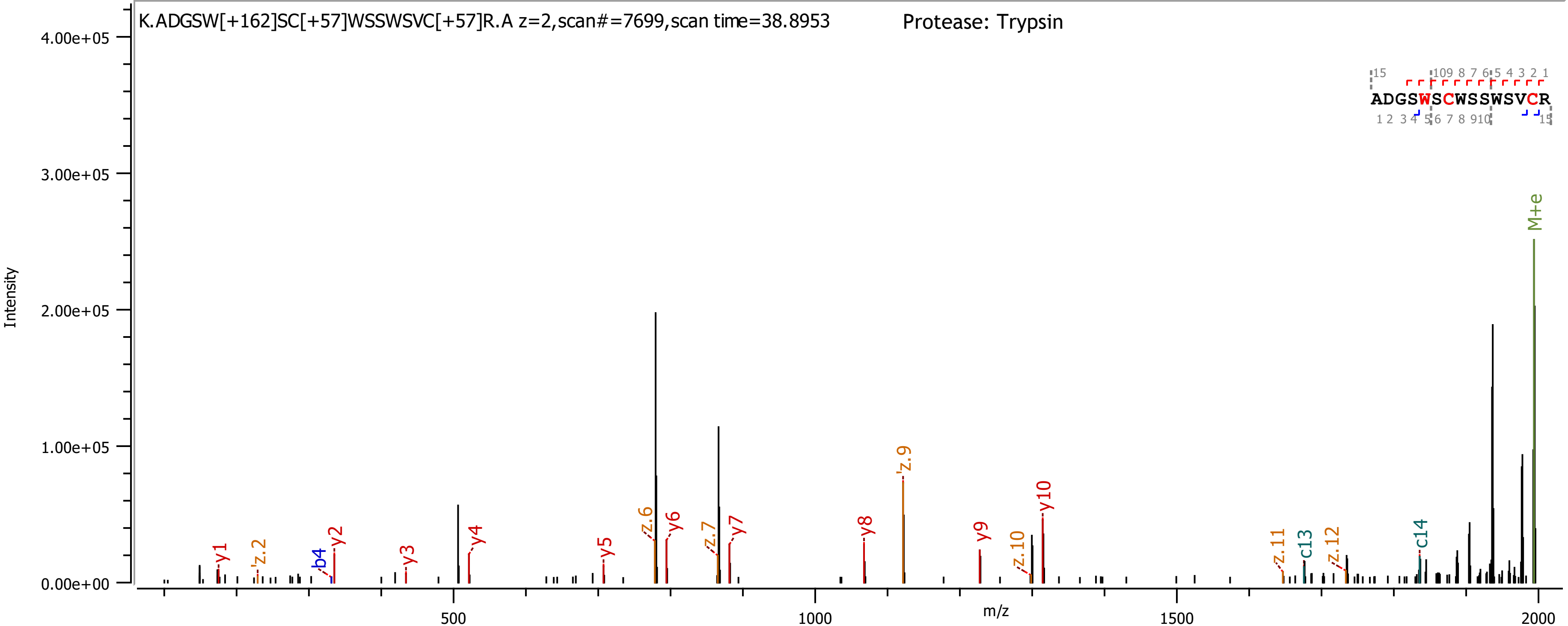

K.ADGSW[+162]SC[+57]WSSWSVC[+57]R.A z=2,scan#=7699,scan time=38.8953
Protease: Trypsin
ADGSWSCWSSWSVCR
Intensity
m/z
4.00e+05
3.00e+05
2.00e+05
1.00e+05
0.00e+00
500
1000
1500
2000
y1
'z.2
b4
y2
y3
y4
y5
z.6
y6
z.7
y7
y8
'z.9
y9
z.10
y10
z.11
c13
z.12
c14
M+e

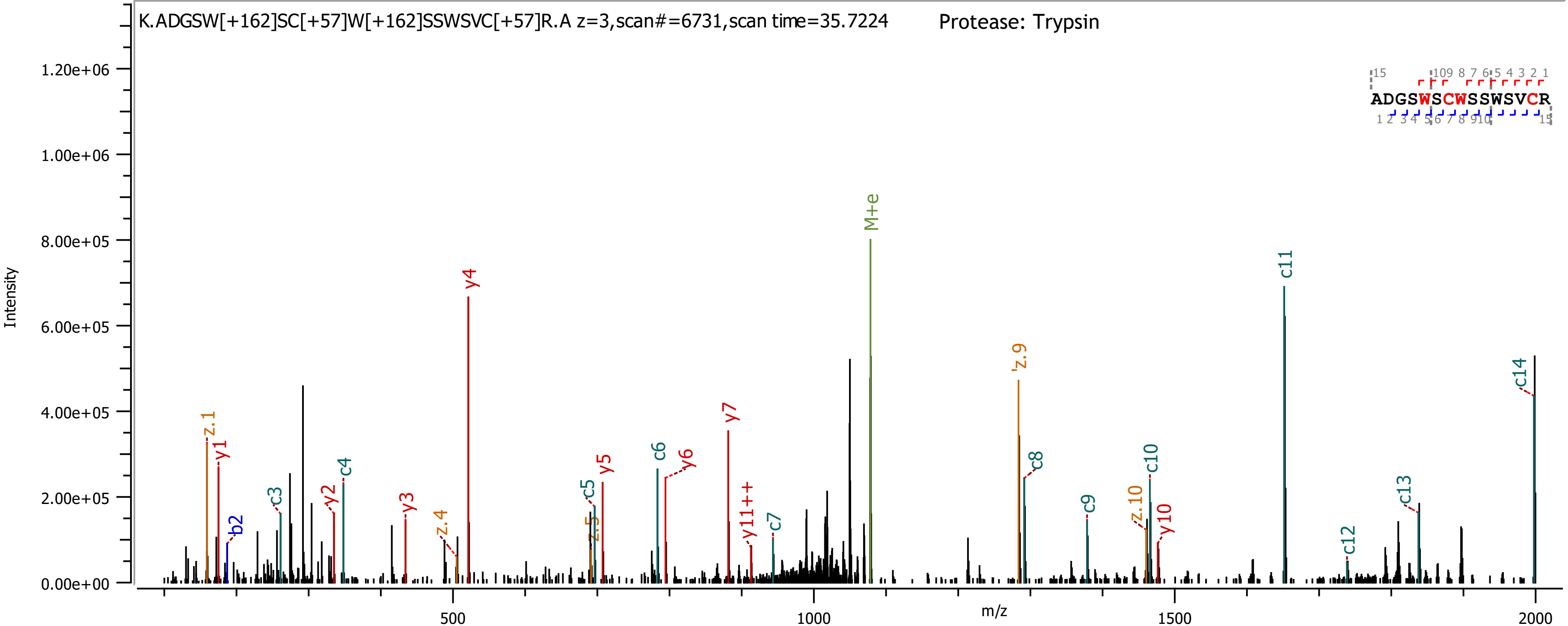

K.ADGSW[+162]SC[+57]W[+162]SSWSVC[+57]R.A z=3,scan#=6731,scan time=35.7224
Protease: Trypsin
ADGSWSCWSSWSVCR
Intensity
m/z
M+e
y4
c11
z.9
c14
y7
z.1
y1
c6
y6
y5
c4
c8
c10
c3
y2
y3
c5
b2
y11++
z.4
c9
z.10
y10
c13
c7
c12

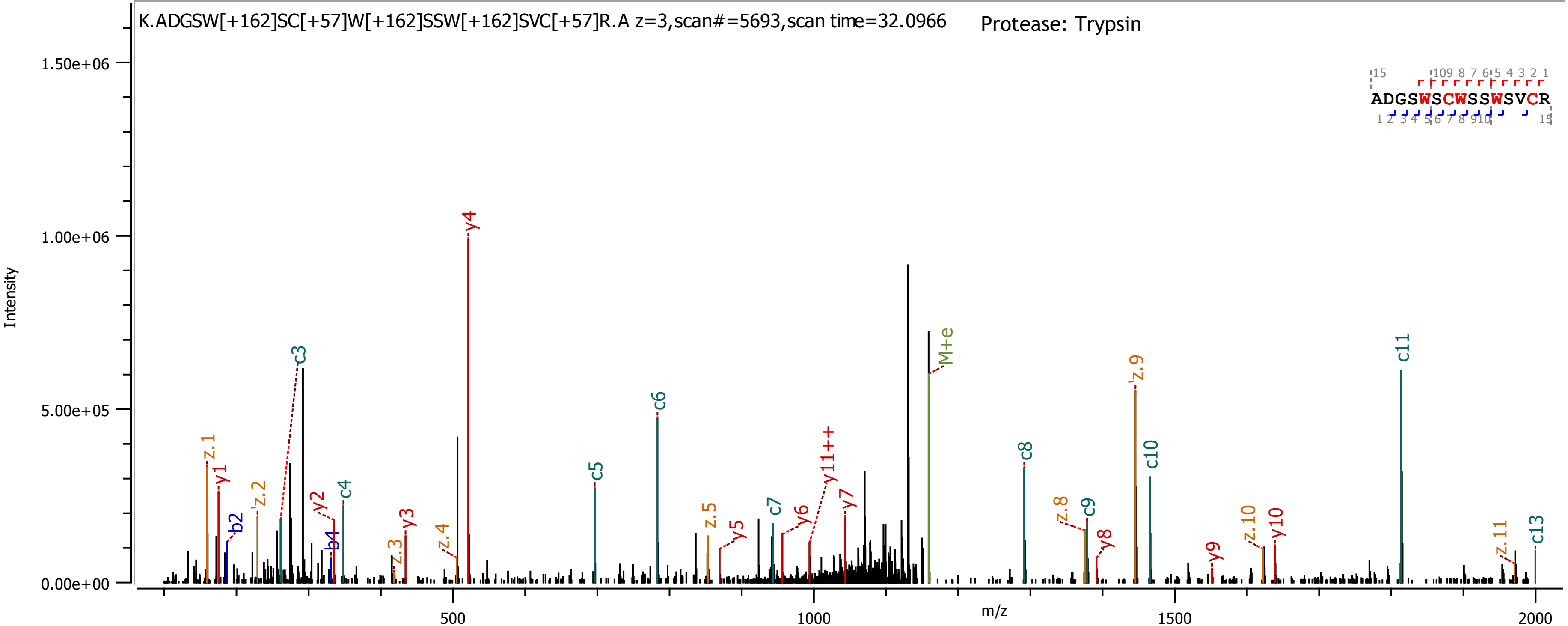

K.ADGSW[+162]SC[+57]W[+162]SSW[+162]SVC[+57]R.A z=3,scan#=5693,scan time=32.0966
Protease: Trypsin
ADGSWSCWSSWSVCR
Intensity
1.50e+06
1.00e+06
5.00e+05
0.00e+00
m/z
500
1000
1500
2000
z.1
y1
b2
'z.2
c3
y2
b4
c4
z.3
y3
z.4
y4
c5
c6
z.5
y5
c7
y6
y11++
y7
M+e
c8
z.8
c9
y8
'z.9
c10
y9
z.10
y10
c11
z.11
c13

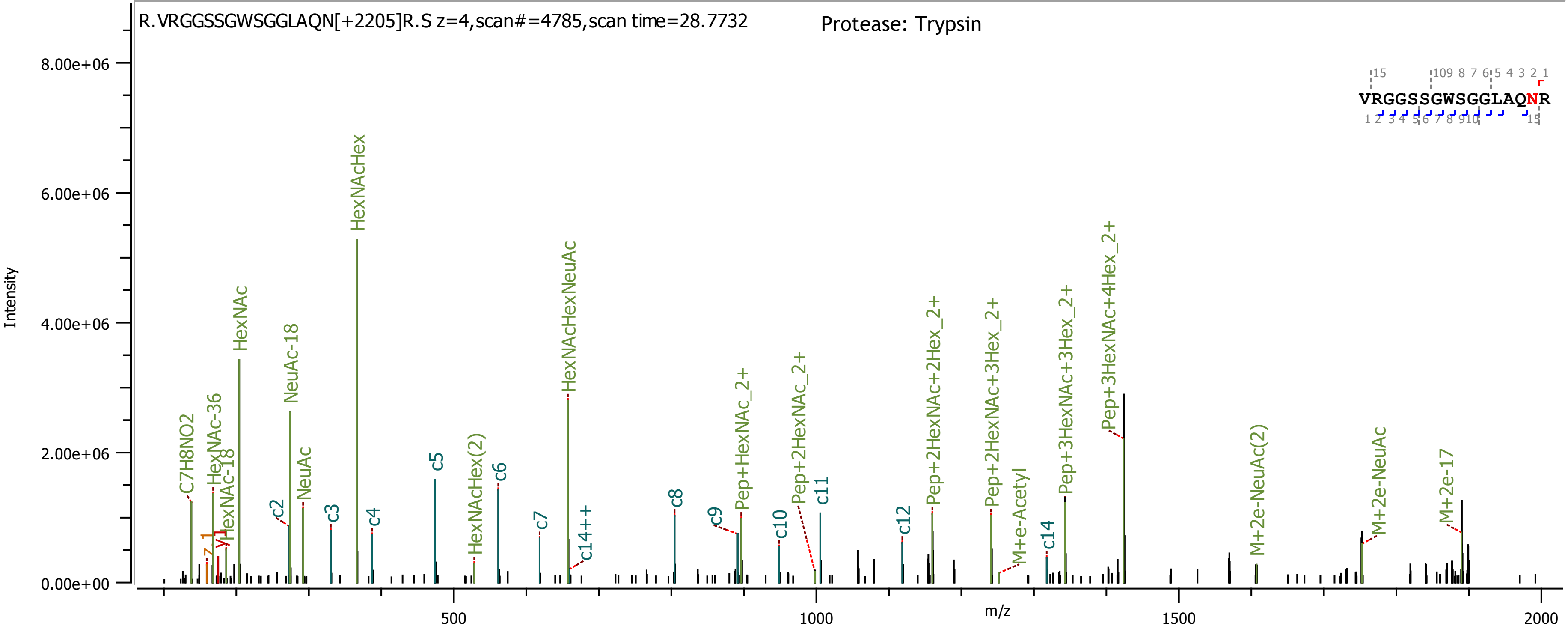

R.VRGGSSGWSGGLAQN[+2205]R.S z=4,scan#=4785,scan time=28.7732
Protease: Trypsin
VRGGSSGWSGGLAQNR
Intensity
m/z
8.00e+06
6.00e+06
4.00e+06
2.00e+06
0.00e+00
500
1000
1500
2000
C7H8NO2
HexNAc-36
HexNAc-18
HexNAc
NeuAc-18
NeuAc
HexNAcHex
HexNAcHexNeuAc
HexNAcHex(2)
c2
c3
c4
c5
c6
c7
c8
c9
c10
c11
c12
c14
c14++
y1
z 1
Pep+HexNAc_2+
Pep+2HexNAc_2+
Pep+2HexNAc+2Hex_2+
Pep+2HexNAc+3Hex_2+
Pep+3HexNAc+3Hex_2+
Pep+3HexNAc+4Hex_2+
M+e-Acetyl
M+2e-NeuAc(2)
M+2e-NeuAc
M+2e-17

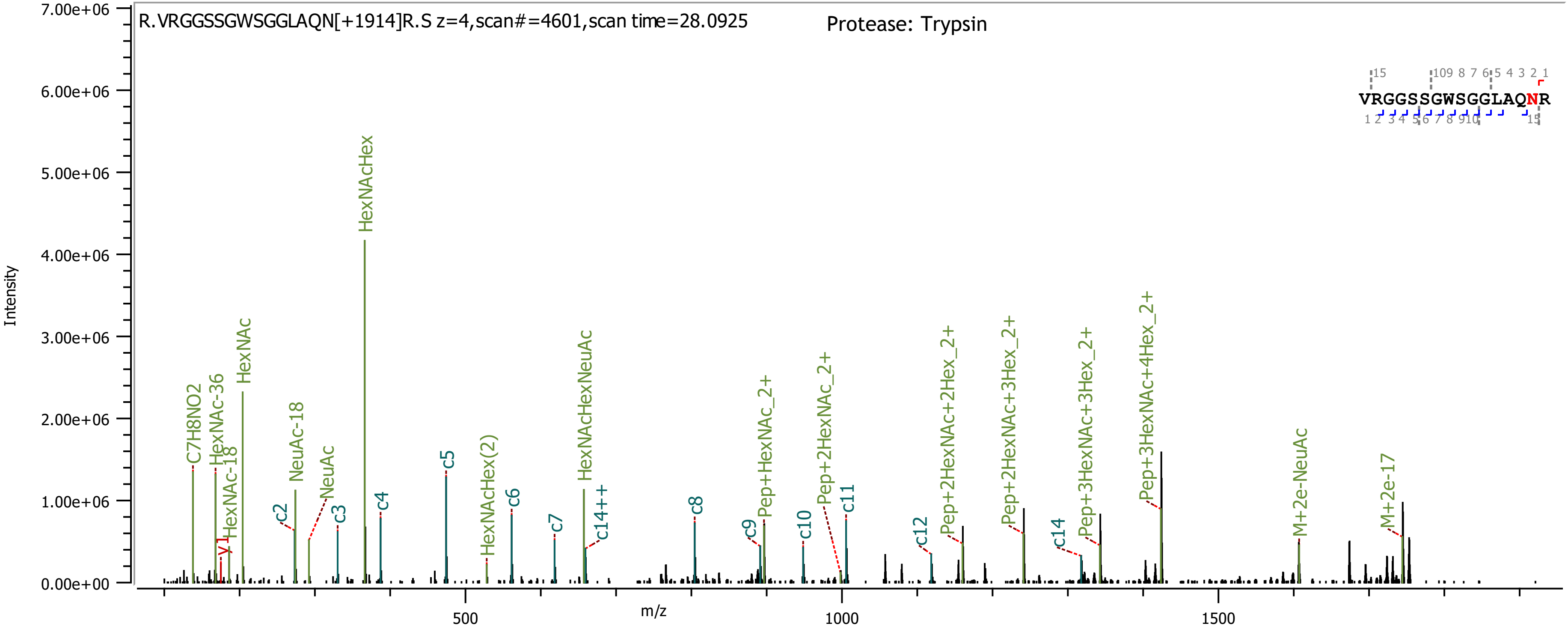

R.VRGGSSGWSGGLAQN[+1914]R.S z=4,scan#=4601,scan time=28.0925
Protease: Trypsin
VRGGSSGWSGGLAQNR
Intensity
m/z
C7H8NO2
HexNAc-36
HexNAc-18
y1
HexNAc
c2
NeuAc-18
NeuAc
c3
HexNAcHex
c4
c5
HexNAcHex(2)
c6
c7
HexNAcHexNeuAc
c14++
c8
c9
Pep+HexNAc_2+
c10
Pep+2HexNAc_2+
c11
c12
Pep+2HexNAc+2Hex_2+
Pep+2HexNAc+3Hex_2+
c14
Pep+3HexNAc+3Hex_2+
Pep+3HexNAc+4Hex_2+
M+2e-NeuAc
M+2e-17

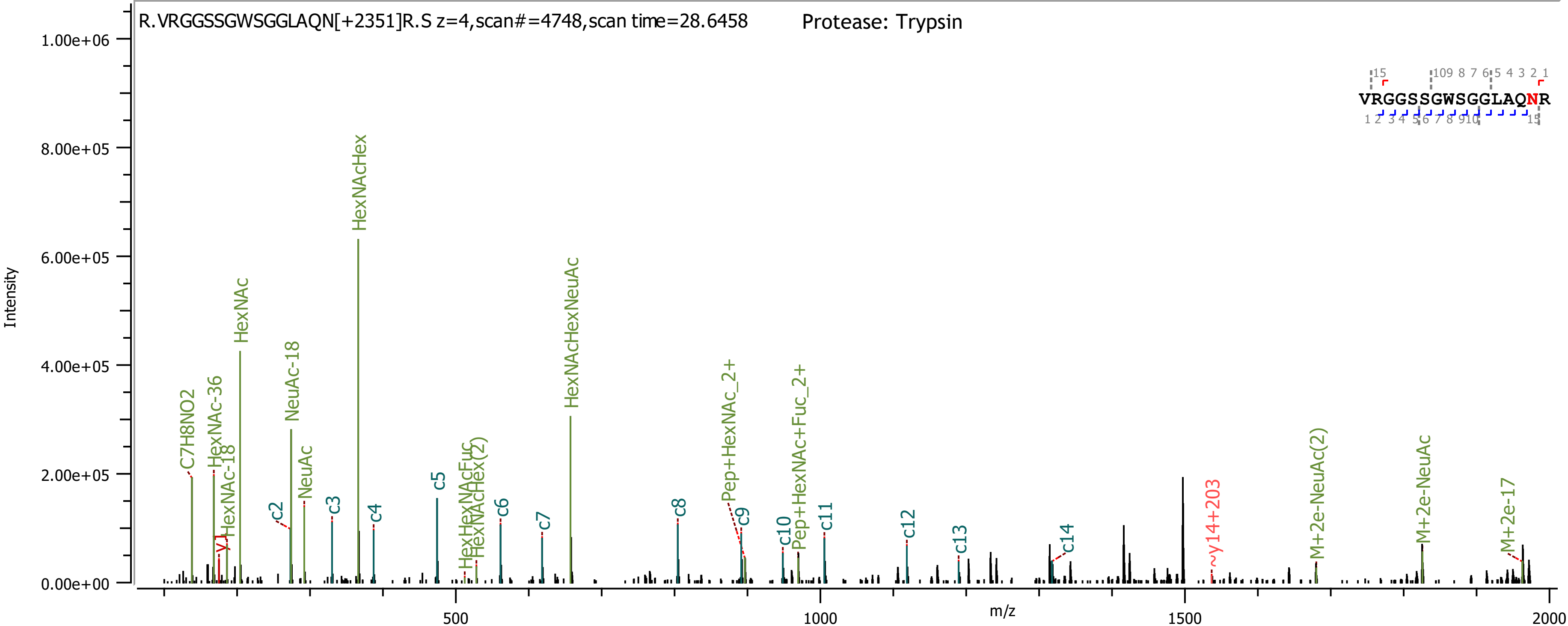
R.VRGGSSGWSGGLAQN[+2351]R.S z=4,scan#=4748,scan time=28.6458
Protease: Trypsin
VRGGSSGWSGGLAQNR
Intensity
m/z
C7H8NO2
HexNAc-36
HexNAc-18
HexNAc
NeuAc-18
NeuAc
HexNAcHex
HexNAcHexNeuAc
HexHexNAcFuc
HexNAcHex(2)
Pep+HexNAc_2+
Pep+HexNAc+Fuc_2+
c2
c3
c4
c5
c6
c7
c8
c9
c10
c11
c12
c13
c14
y1
~y14+203
M+2e-NeuAc(2)
M+2e-NeuAc
M+2e-17

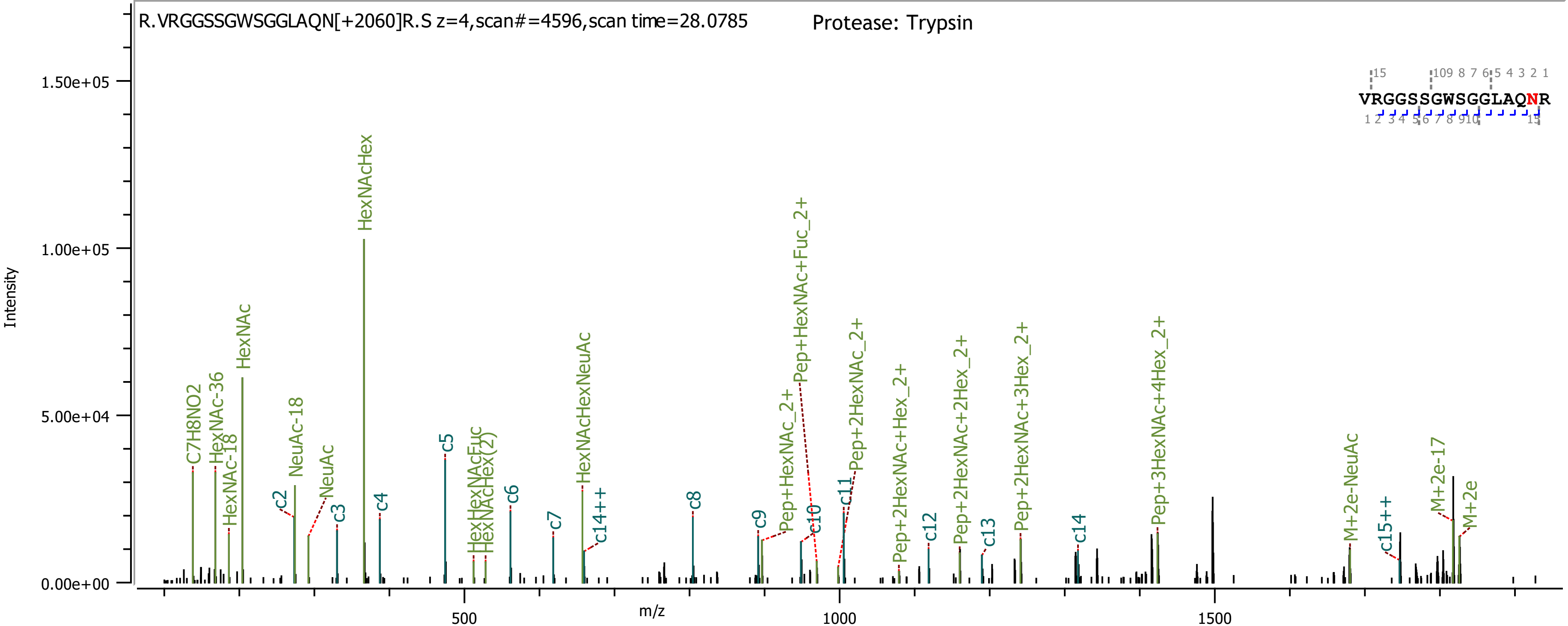

R.VRGGSSGWSGGLAQN[+2060]R.S z=4,scan#=4596,scan time=28.0785
Protease: Trypsin
VRGGSSGWSGGLAQNR
Intensity
1.50e+05
1.00e+05
5.00e+04
0.00e+00
m/z
500
1000
1500
C7H8NO2
HexNAc-36
HexNAc-18
HexNAc
c2
NeuAc-18
NeuAc
c3
HexNAcHex
c4
c5
HexHexNAcFuc
HexNAcHex(2)
c6
c7
HexNAcHexNeuAc
c14++
c8
c9
Pep+HexNAc_2+
Pep+HexNAc+Fuc_2+
c10
c11
Pep+2HexNAc_2+
Pep+2HexNAc+Hex_2+
c12
Pep+2HexNAc+2Hex_2+
c13
Pep+2HexNAc+3Hex_2+
c14
Pep+3HexNAc+4Hex_2+
M+2e-NeuAc
c15++
M+2e-17
M+2e

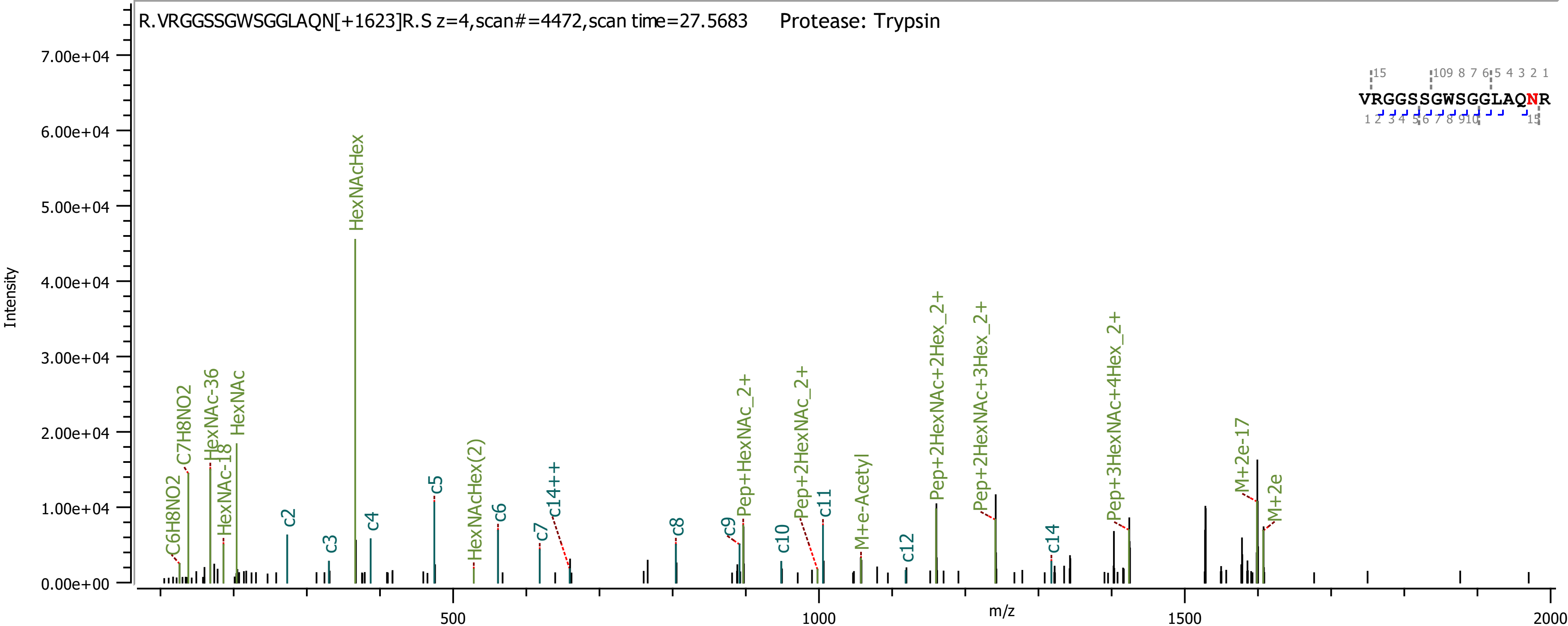

R.VRGGSSGWSGGLAQN[+1623]R.S z=4,scan#=4472,scan time=27.5683
Protease: Trypsin
VRGGSSGWSGGLAQNR
Intensity
m/z
0.00e+00
1.00e+04
2.00e+04
3.00e+04
4.00e+04
5.00e+04
6.00e+04
7.00e+04
500
1000
1500
2000
C6H8NO2
C7H8NO2
HexNAc-36
HexNAc-18
HexNAc
c2
c3
HexNAcHex
c4
c5
HexNAcHex(2)
c6
c7
c14++
c8
c9
Pep+HexNAc_2+
c10
Pep+2HexNAc_2+
c11
M+e-Acetyl
c12
Pep+2HexNAc+2Hex_2+
Pep+2HexNAc+3Hex_2+
c14
Pep+3HexNAc+4Hex_2+
M+2e-17
M+2e

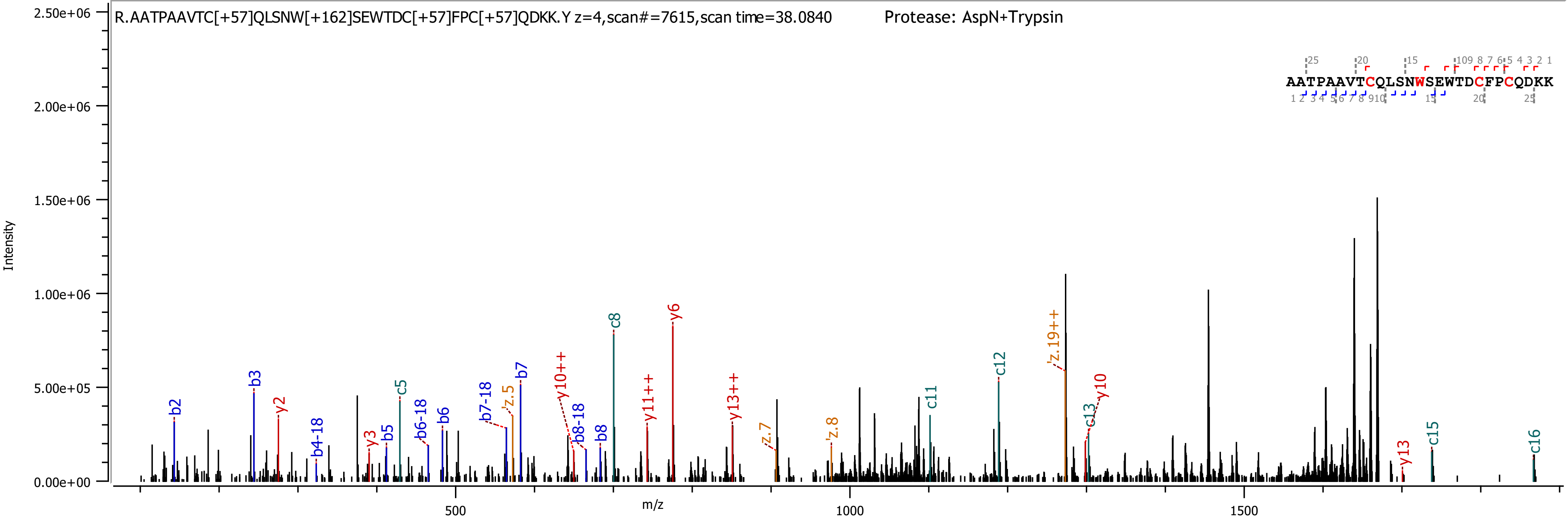
R.AATPAAVTC[+57]QLSNW[+162]SEWTDC[+57]FPC[+57]QDKK.Y z=4,scan#=7615,scan time=38.0840
Protease: AspN+Trypsin
AATPAAVTCQLSNWSEWTDCFPCQDKK
Intensity
m/z
2.50e+06
2.00e+06
1.50e+06
1.00e+06
5.00e+05
0.00e+00
500
1000
1500
b2
b3
y2
b4-18
y3
b5
c5
b6-18
b6
b7-18
'z.5
b7
y10++
b8-18
b8
c8
y11++
y6
y13++
'z.7
'z.8
c11
c12
'z.19++
c13
y10
y13
c15
c16

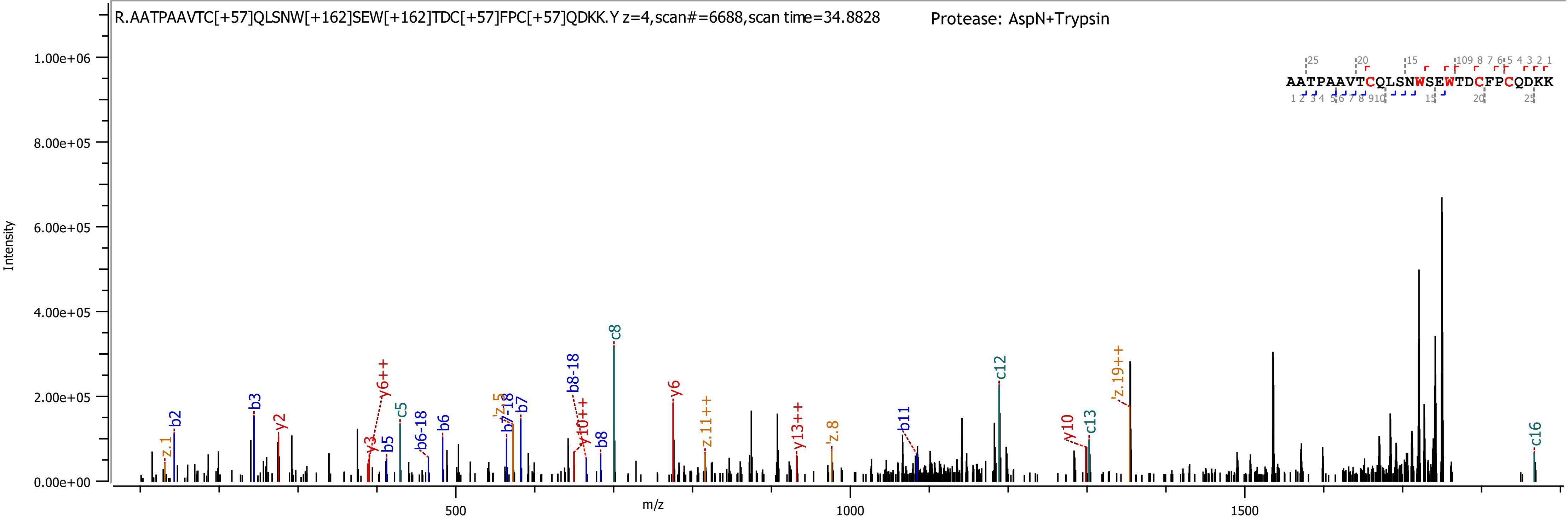
R.AATPAAVTC[+57]QLSNW[+162]SEW[+162]TDC[+57]FPC[+57]QDKK.Y z=4,scan#=6688,scan time=34.8828
Protease: AspN+Trypsin
AATPAAVTCQLSNWSEWTDCFPCQDKK
Intensity
1.00e+06
8.00e+05
6.00e+05
4.00e+05
2.00e+05
0.00e+00
m/z
500
1000
1500
z.1
b2
b3
y2
y3
y6++
b5
c5
b6-18
b6
z.5
b7-18
b7
b8-18
y10++
b8
c8
y6
z.11++
y13++
z.8
b11
c12
y10
c13
z.19++
c16

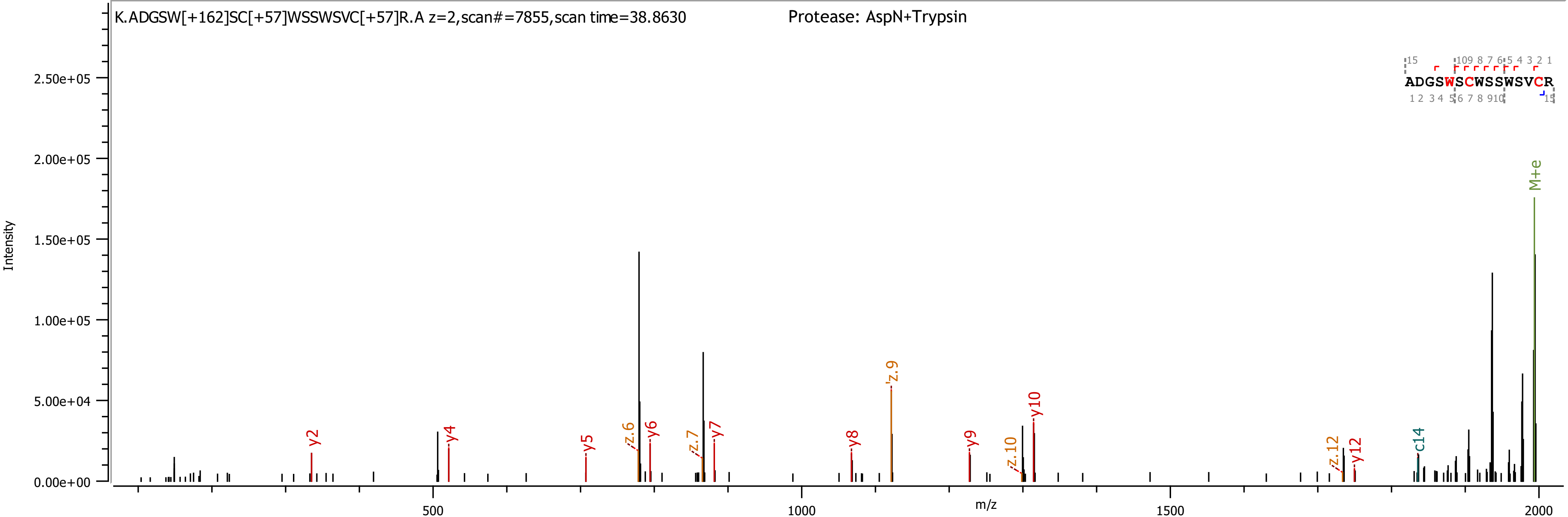
K.ADGSW[+162]SC[+57]WSSWSVC[+57]R.A z=2,scan#=7855,scan time=38.8630
Protease: AspN+Trypsin
ADGSWSCWSSWSVCR
Intensity
m/z
2.50e+05
2.00e+05
1.50e+05
1.00e+05
5.00e+04
0.00e+00
500
1000
1500
2000
y2
y4
y5
z.6
y6
z.7
y7
y8
z.9
y9
z.10
y10
z.12
y12
c14
M+e

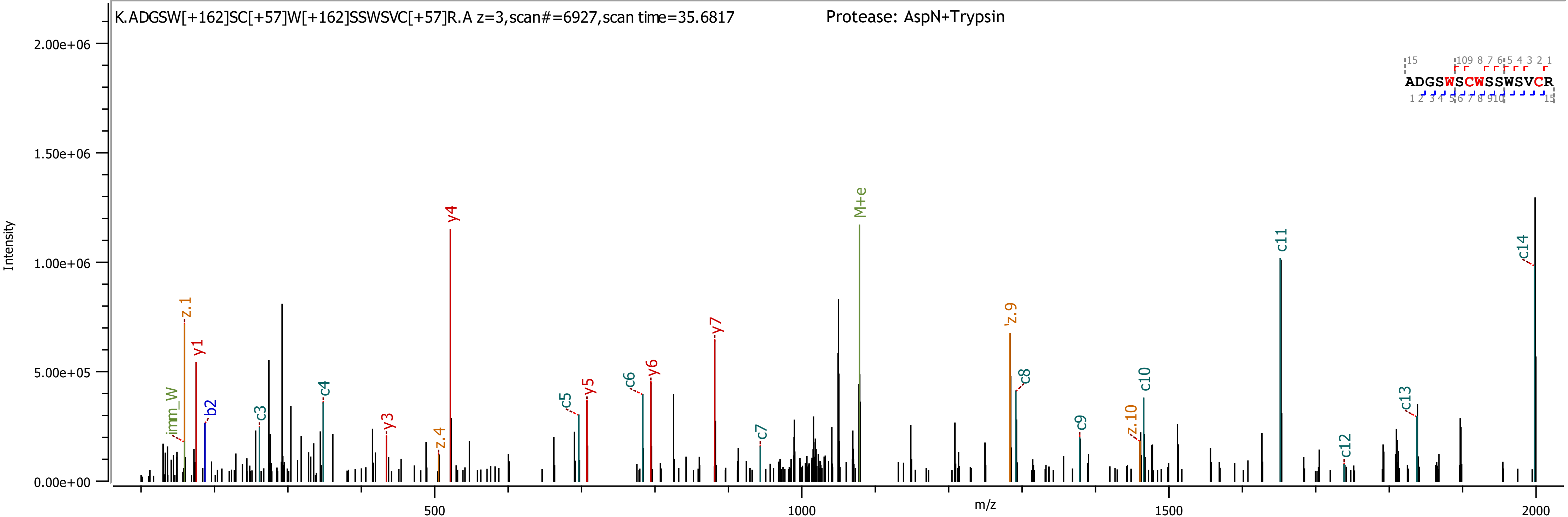

K.ADGSW[+162]SC[+57]W[+162]SSWSVC[+57]R.A z=3,scan#=6927,scan time=35.6817
Protease: AspN+Trypsin
ADGSWSCWSSWSVCR
Intensity
m/z
2.00e+06
1.50e+06
1.00e+06
5.00e+05
0.00e+00
500
1000
1500
2000
imm_W
z.1
y1
b2
c3
c4
y3
z.4
y4
c5
y5
c6
y6
y7
c7
M+e
'z.9
c8
c9
z.10
c10
c11
c12
c13
c14

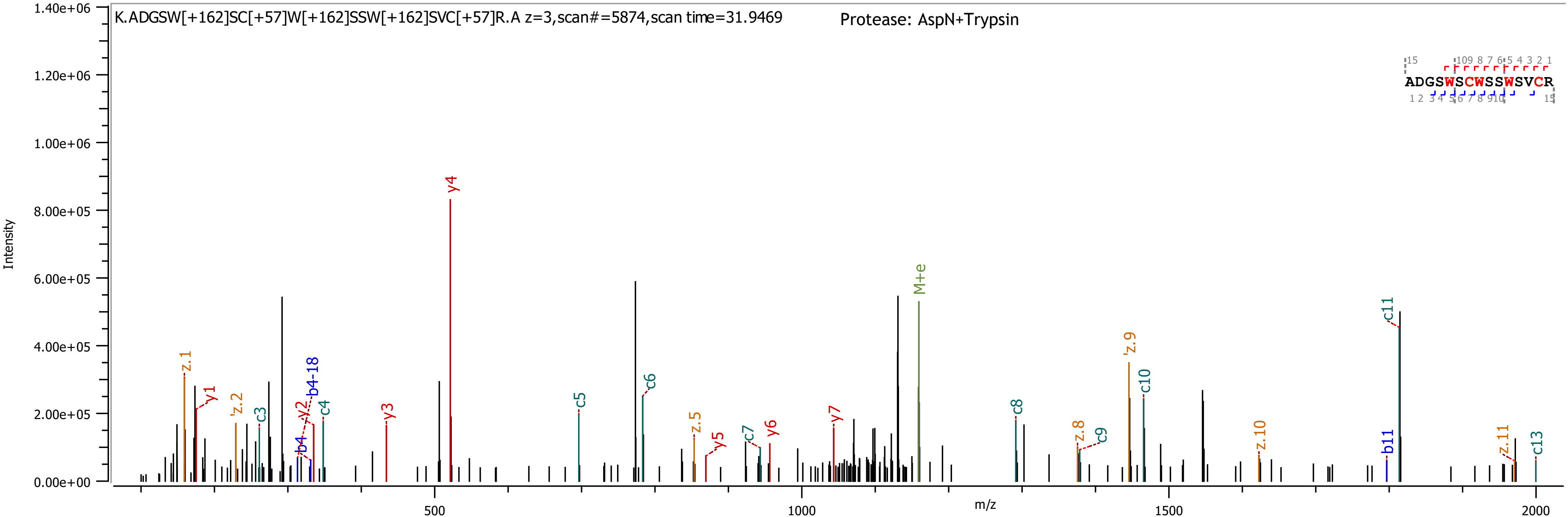
K.ADGSW[+162]SC[+57]W[+162]SSW[+162]SVC[+57]R.A z=3,scan#=5874,scan time=31.9469
Protease: AspN+Trypsin
ADGSWSCWSSSWSVCR
Intensity
m/z
y4
M+e
c11
c6
c5
z.1
y1
'z.2
c3
b4
y2
b4-18
c4
y3
z.5
y5
c7
y6
y7
c8
z.8
c9
'z.9
c10
z.10
b11
z.11
c13

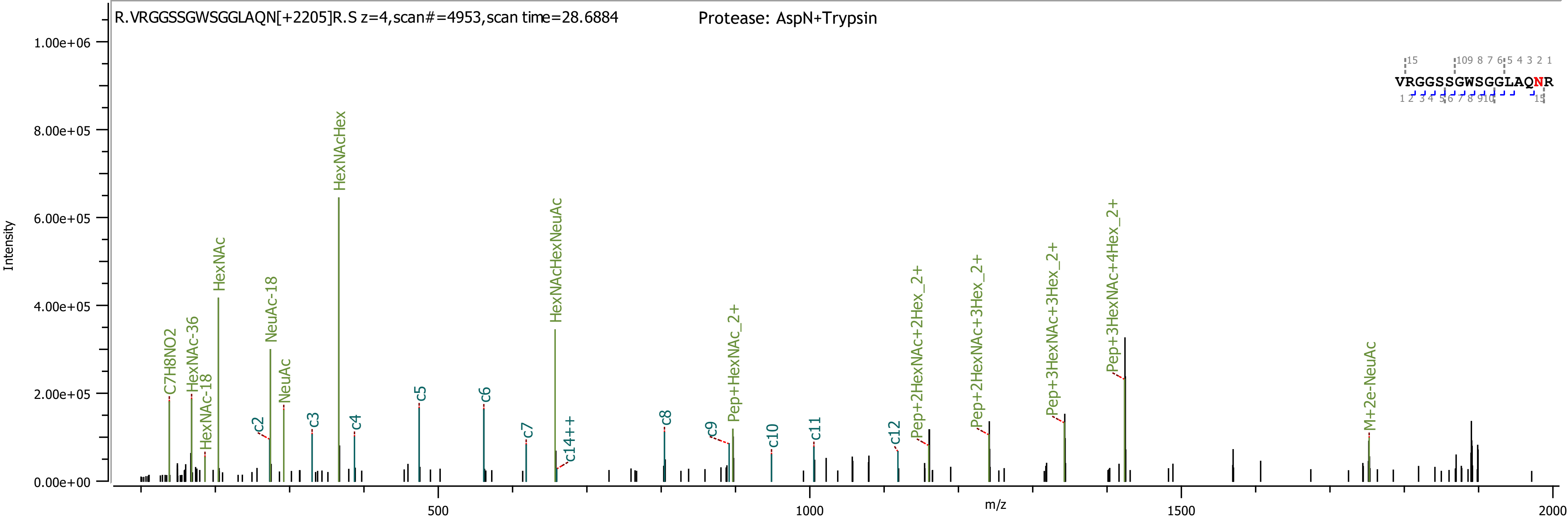
R.VRGGSSGWSGGLAQN[+2205]R.S z=4,scan#=4953,scan time=28.6884
Protease: AspN+Trypsin
VRGGSSGWSGGLAQNR
Intensity
m/z
C7H8NO2
HexNAc-36
HexNAc-18
HexNAc
c2
NeuAc-18
NeuAc
c3
HexNAcHex
c4
c5
c6
c7
HexNAcHexNeuAc
c14++
c8
c9
Pep+HexNAc_2+
c10
c11
c12
Pep+2HexNAc+2Hex_2+
Pep+2HexNAc+3Hex_2+
Pep+3HexNAc+3Hex_2+
Pep+3HexNAc+4Hex_2+
M+2e-NeuAc

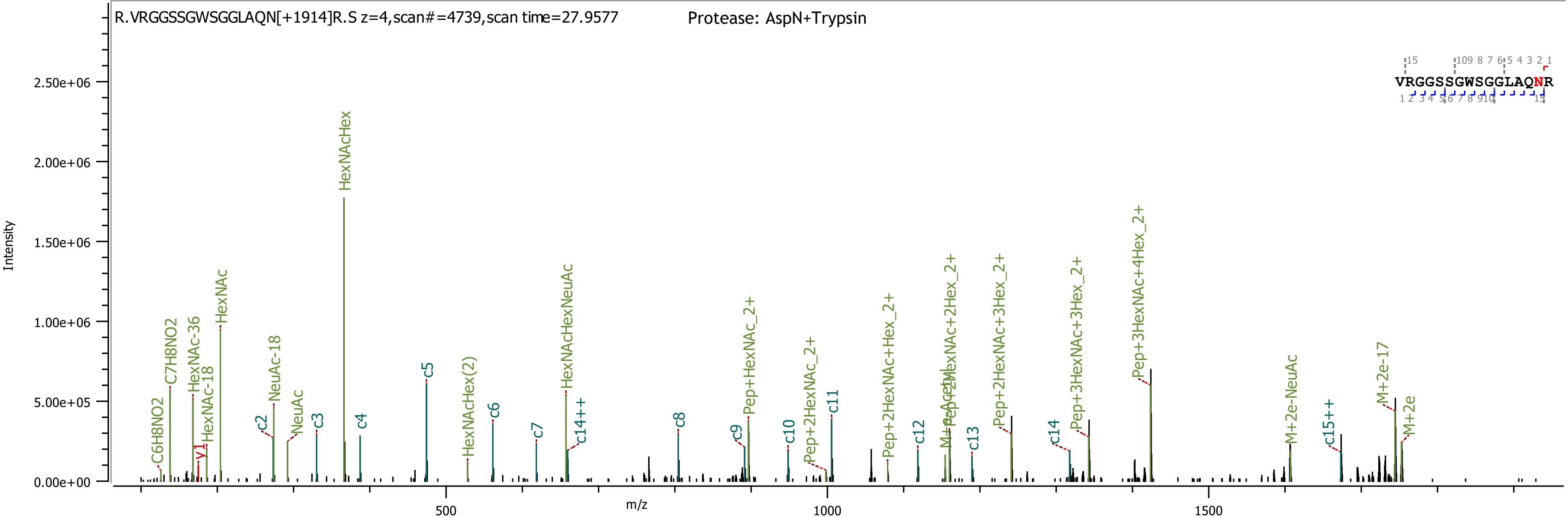
R.VRGGSSGWSGGLAQN[+1914]R.S z=4,scan#=4739,scan time=27.9577
Protease: AspN+Trypsin
VRGGSSGWSGGLAQNR
Intensity
m/z
2.50e+06
2.00e+06
1.50e+06
1.00e+06
5.00e+05
0.00e+00
500
1000
1500
C6H8NO2
C7H8NO2
HexNAc-36
y1
HexNAc-18
HexNAc
c2
NeuAc-18
NeuAc
c3
HexNAcHex
c4
c5
HexNAcHex(2)
c6
c7
HexNAcHexNeuAc
c14++
c8
c9
Pep+HexNAc_2+
c10
Pep+2HexNAc_2+
c11
Pep+2HexNAc+Hex_2+
c12
Pep+2HexNAc+2Hex_2+
c13
Pep+2HexNAc+3Hex_2+
c14
Pep+3HexNAc+3Hex_2+
Pep+3HexNAc+4Hex_2+
M+2e-NeuAc
c15++
M+2e-17
M+2e

HCD spectra

Glycosylation Site

TSR1_WSEW	AATPAAVTCQLSN*W*SEWTDCFPCQDKK
	AATPAAVTCQLSN*W*SE*W*TDCFPCQDKK
TSR2_WSCWSSW	ADGS*W*SCWSSWSVCR
	ADGS*W*SC*W*SSWSVCR
	ADGSWSC*W*SS*W*SVCR
	ADGS*W*SC*W*SS*W*SVCR
N437	VRGGSSGWSGGLAQ*N*[+2204.8]R
	VRGGSSGWSGGLAQ*N*[+1914.8]R
	VRGGSSGWSGGLAQ*N*[+1622.6]R

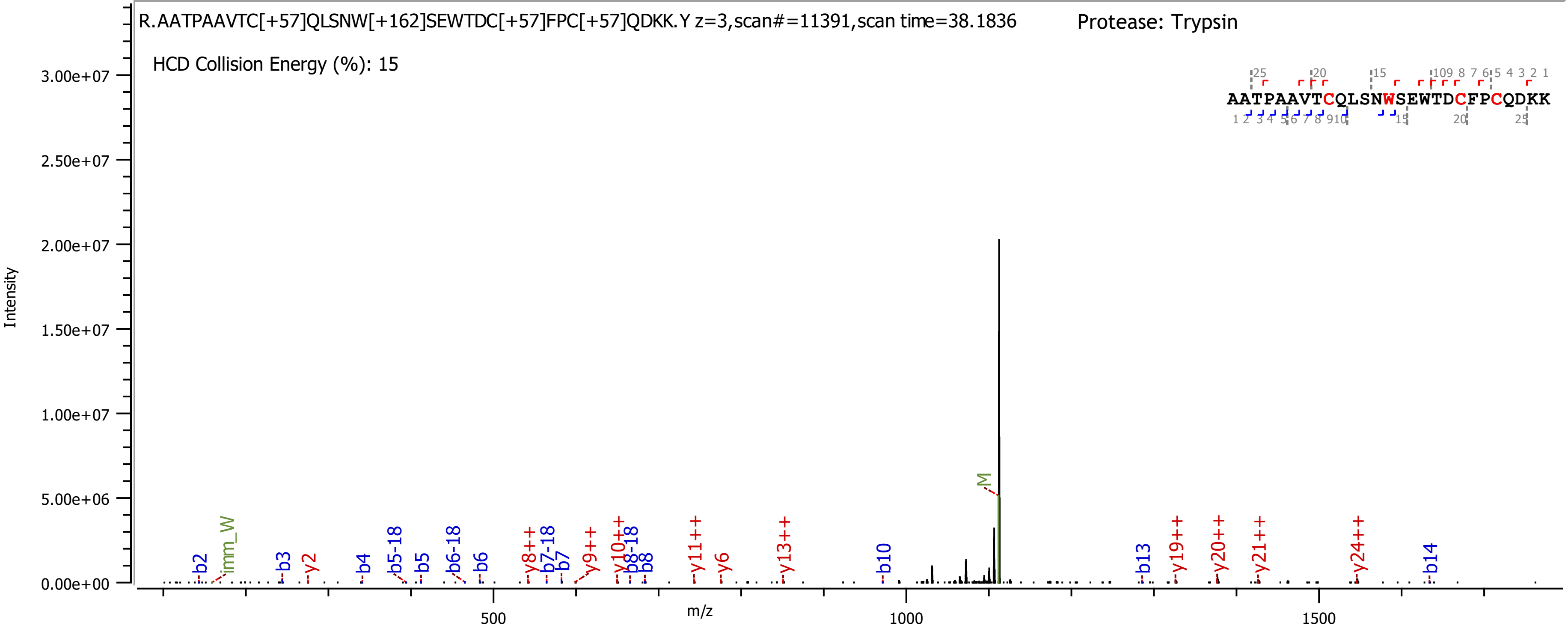

R.AATPAAVTC[+57]QLSNW[+162]SEWTDC[+57]FPC[+57]QDKK.Y z=3,scan#=11391,scan time=38.1836
Protease: Trypsin
HCD Collision Energy (%): 15
AATPAAVTCQLSNWSEWTDCFPCQDKK
Intensity
m/z
b2
imm_W
b3
y2
b4
b5-18
b5
b6-18
b6
y8++
b7-18
b7
y9++
y10++
b8-18
b8
y11++
y6
y13++
b10
M
b13
y19++
y20++
y21++
y24++
b14

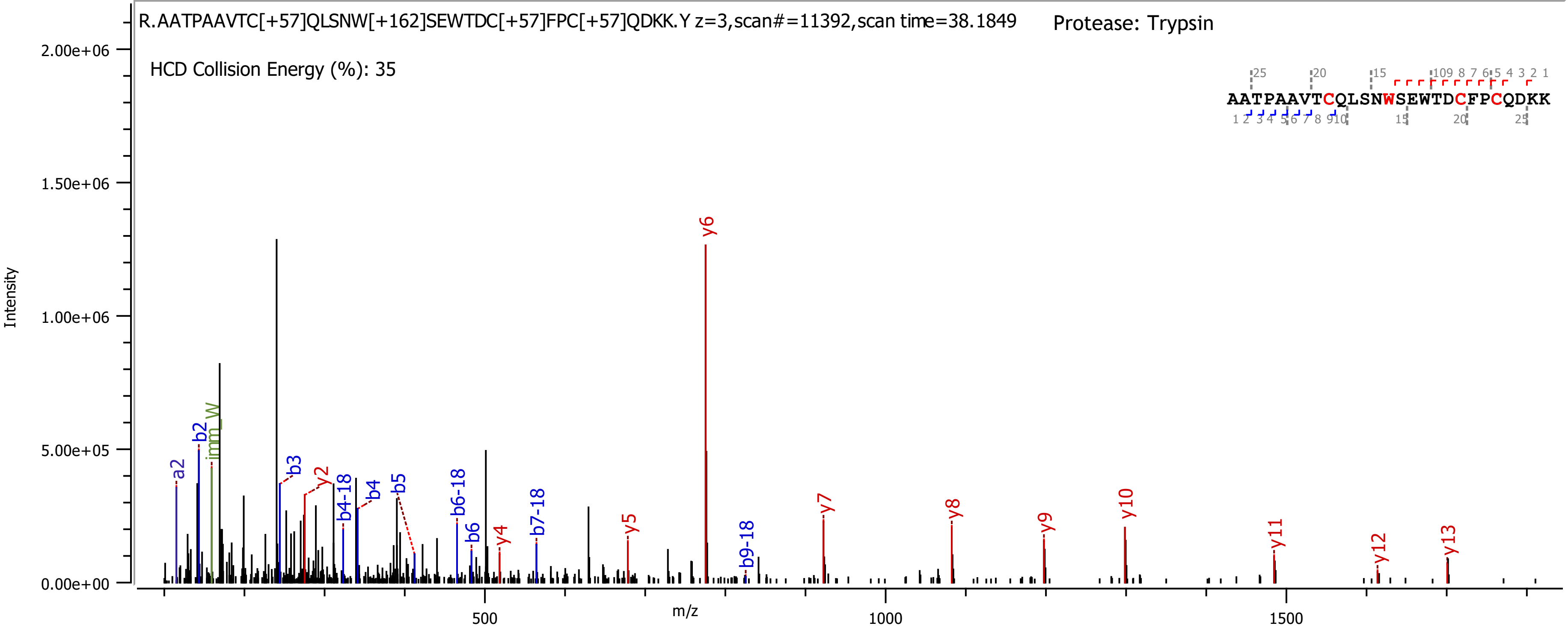
R.AATPAAVTC[+57]QLSNW[+162]SEWTDC[+57]FPC[+57]QDKK.Y z=3,scan#=11392,scan time=38.1849
Protease: Trypsin
HCD Collision Energy (%): 35
AATPAAVTCQLSNWSEWTDCFPCQDKK
Intensity
m/z
2.00e+06
1.50e+06
1.00e+06
5.00e+05
0.00e+00
500
1000
1500
a2
b2
imm_W
b3
y2
b4-18
b4
b5
b6-18
b6
y4
b7-18
y5
y6
b9-18
y7
y8
y9
y10
y11
y12
y13

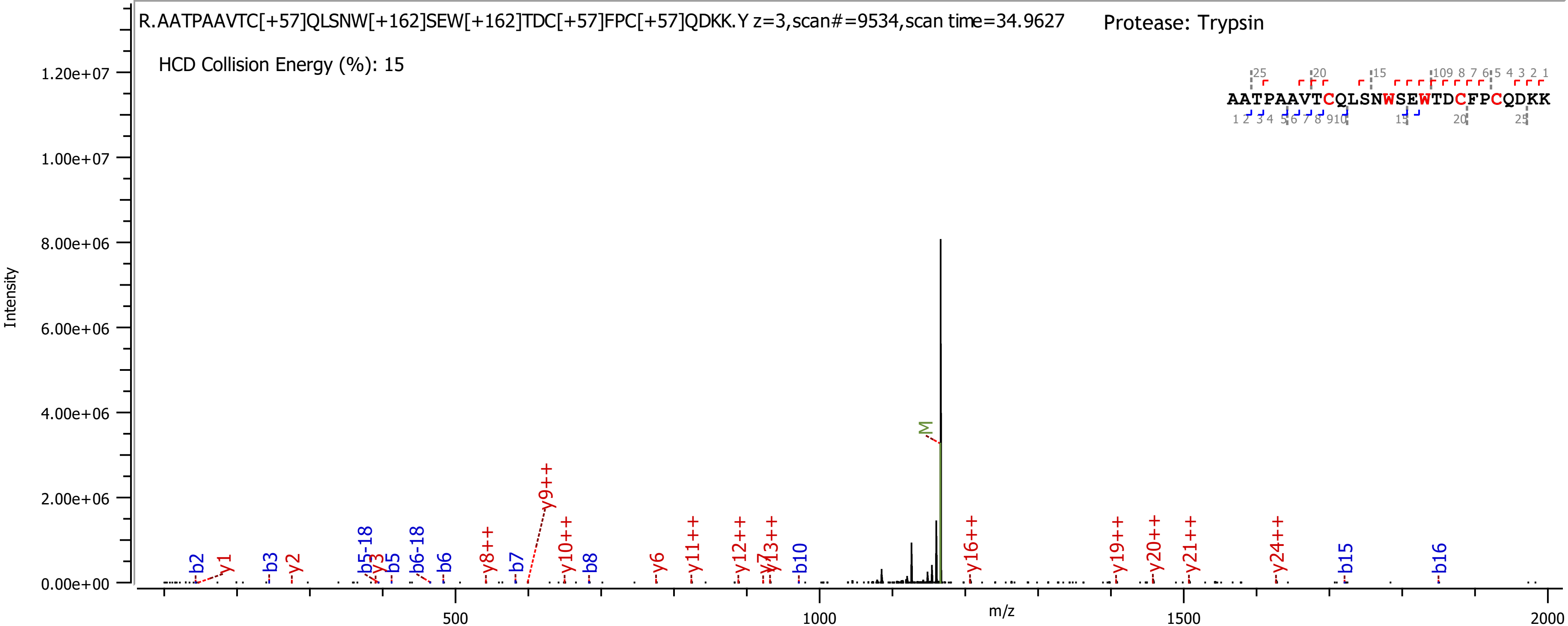
R.AATPAAVTC[+57]QLSNW[+162]SEW[+162]TDC[+57]FPC[+57]QDKK.Y z=3,scan#=9534,scan time=34.9627
Protease: Trypsin
HCD Collision Energy (%): 15
AATPAAVTCQLSNWSEWTDCFPCQDKK
Intensity
m/z
b2 y1 b3 y2 b5-18 y3 b5 b6-18 b6 y8++ b7 y9++ y10++ b8 y6 y11++ y12++ y7 y13++ b10 M y16++ y19++ y20++ y21++ y24++ b15 b16

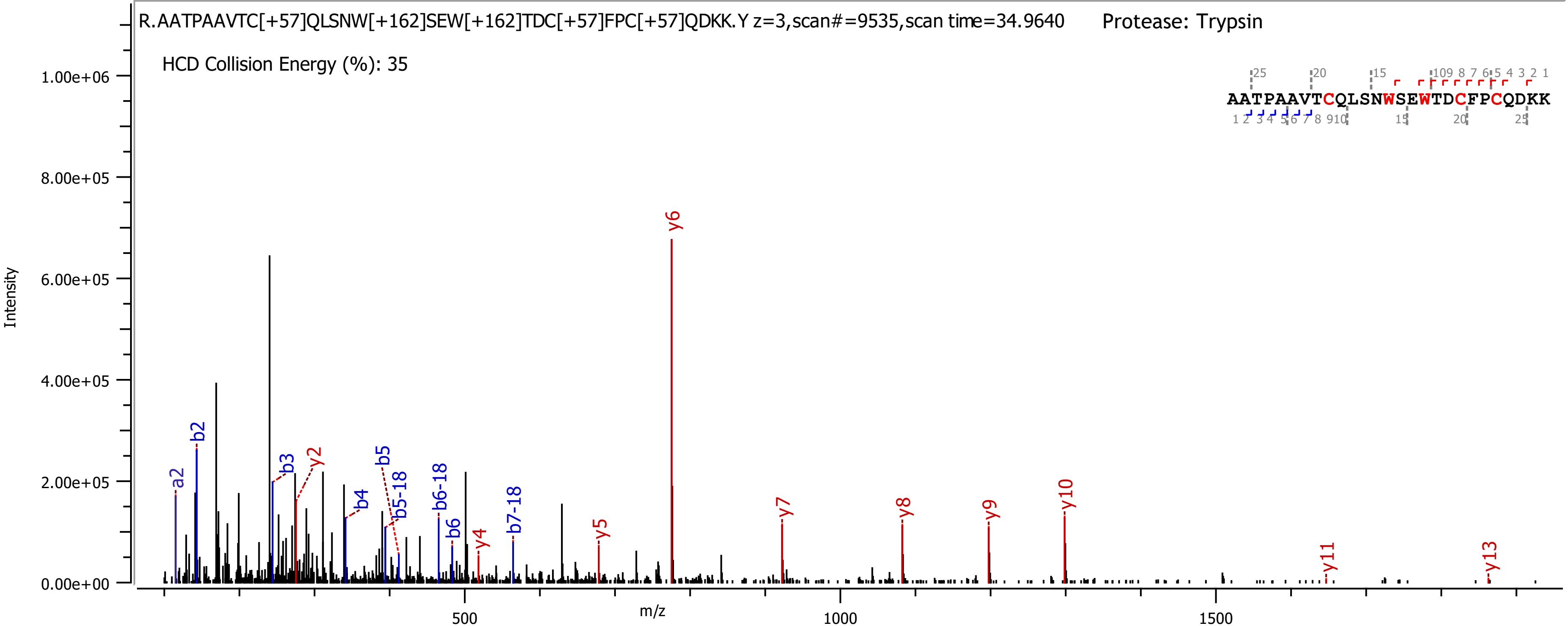
R.AATPAAVTC[+57]QLSNW[+162]SEW[+162]TDC[+57]FPC[+57]QDKK.Y z=3,scan#=9535,scan time=34.9640
Protease: Trypsin
HCD Collision Energy (%): 35
AATPAAVTCQLSNWSEWTDCFPCQDKK
Intensity
m/z
a2
b2
b3
y2
b4
b5
b5-18
b6-18
b6
y4
b7-18
y5
y6
y7
y8
y9
y10
y11
y13

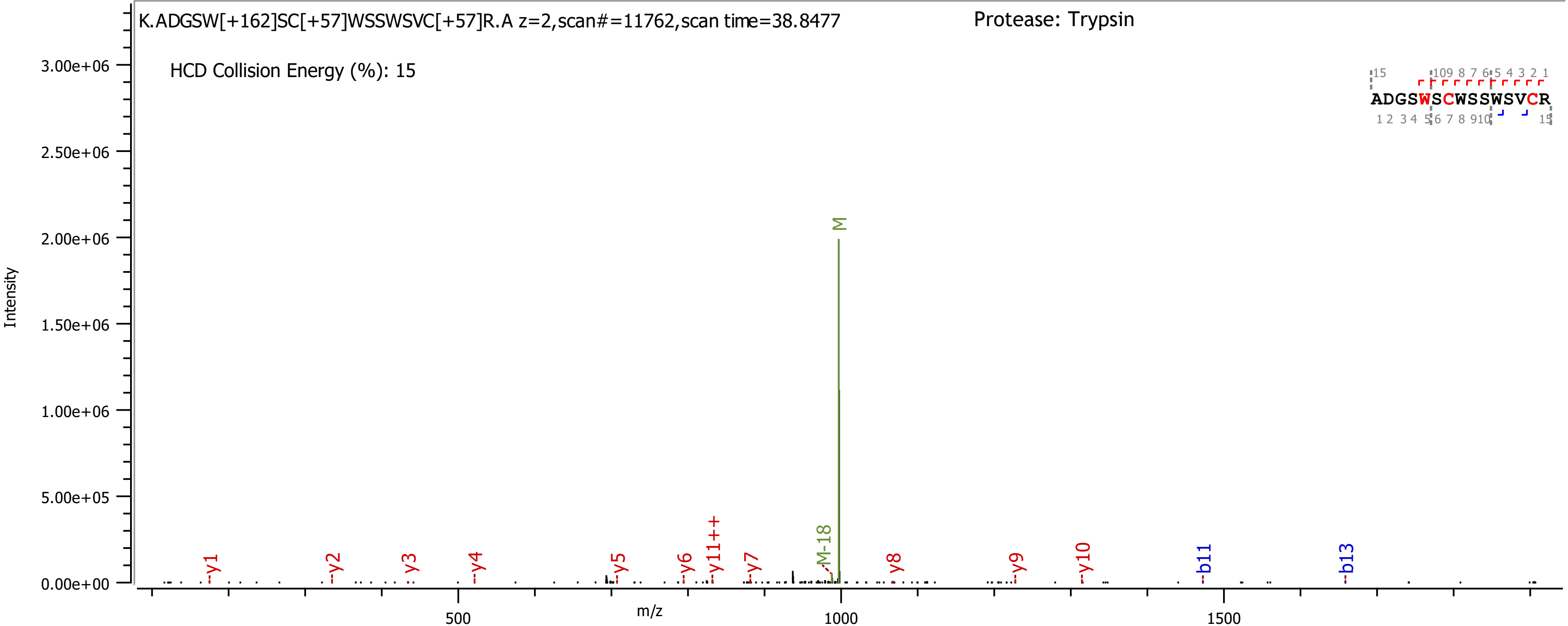
K.ADGSW[+162]SC[+57]WSSWSVC[+57]R.A z=2,scan#=11762,scan time=38.8477
Protease: Trypsin
HCD Collision Energy (%): 15
ADGSWSCWSSWSVCR
Intensity
m/z
M
M-18
y1
y2
y3
y4
y5
y6
y11++
y7
y8
y9
y10
b11
b13
3.00e+06
2.50e+06
2.00e+06
1.50e+06
1.00e+06
5.00e+05
0.00e+00
500
1000
1500

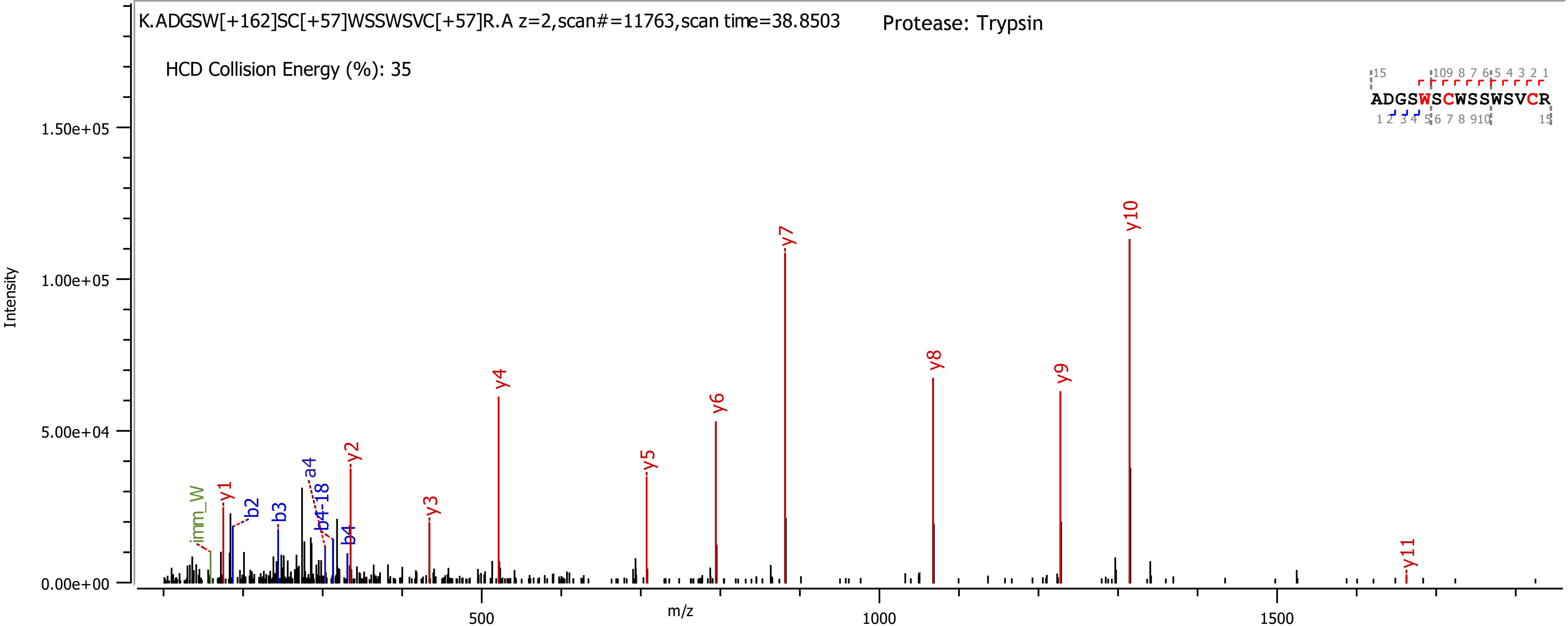
K.ADGSW[+162]SC[+57]WSSWSVC[+57]R.A z=2,scan#=11763,scan time=38.8503
Protease: Trypsin
HCD Collision Energy (%): 35
ADGSWSCWSSWSVCR
Intensity
m/z
1.50e+05
1.00e+05
5.00e+04
0.00e+00
500
1000
1500
imm_W
y1
b2
b3
a4
b4-18
b4
y2
y3
y4
y5
y6
y7
y8
y9
y10
y11

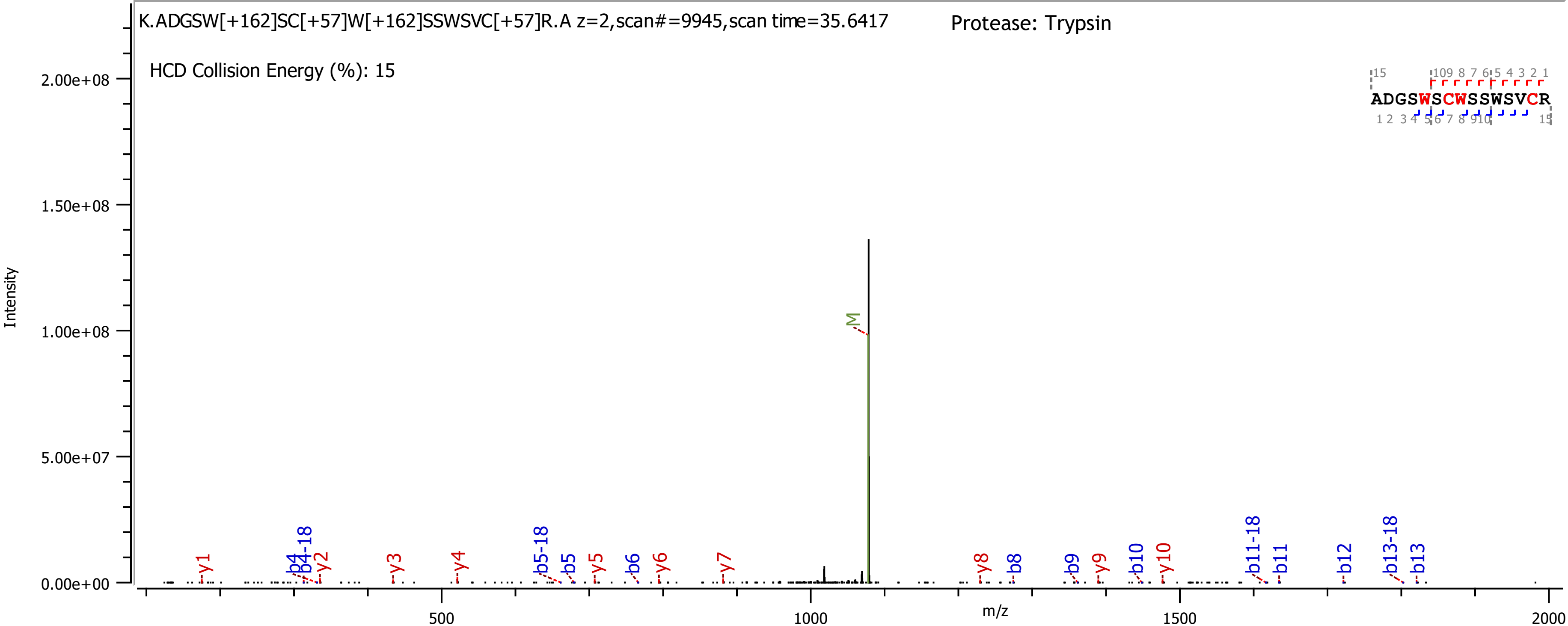

K.ADGSW[+162]SC[+57]W[+162]SSWSVC[+57]R.A z=2,scan#=9945,scan time=35.6417
Protease: Trypsin
HCD Collision Energy (%): 15
ADGSWSCWSSWSVCR
Intensity
m/z
2.00e+08
1.50e+08
1.00e+08
5.00e+07
0.00e+00
500
1000
1500
2000
M
y1
b4
b4-18
y2
y3
y4
b5-18
b5
y5
b6
y6
y7
y8
b8
b9
y9
b10
y10
b11-18
b11
b12
b13-18
b13

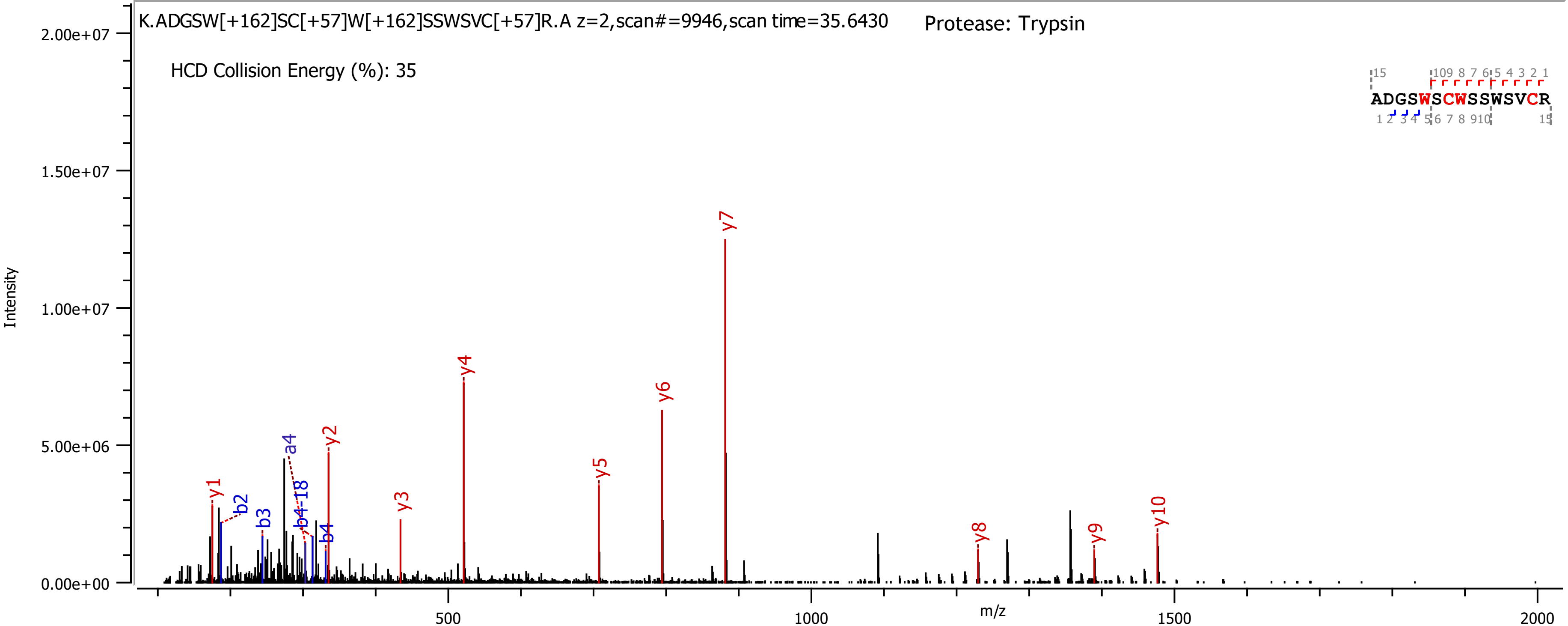
K.ADGSW[+162]SC[+57]W[+162]SSWSVC[+57]R.A z=2,scan#=9946,scan time=35.6430
Protease: Trypsin
HCD Collision Energy (%): 35
ADGSWSCWSSWSVCR
Intensity
m/z
y1
b2
b3
a4
b4-18
b4
y2
y3
y4
y5
y6
y7
y8
y9
y10

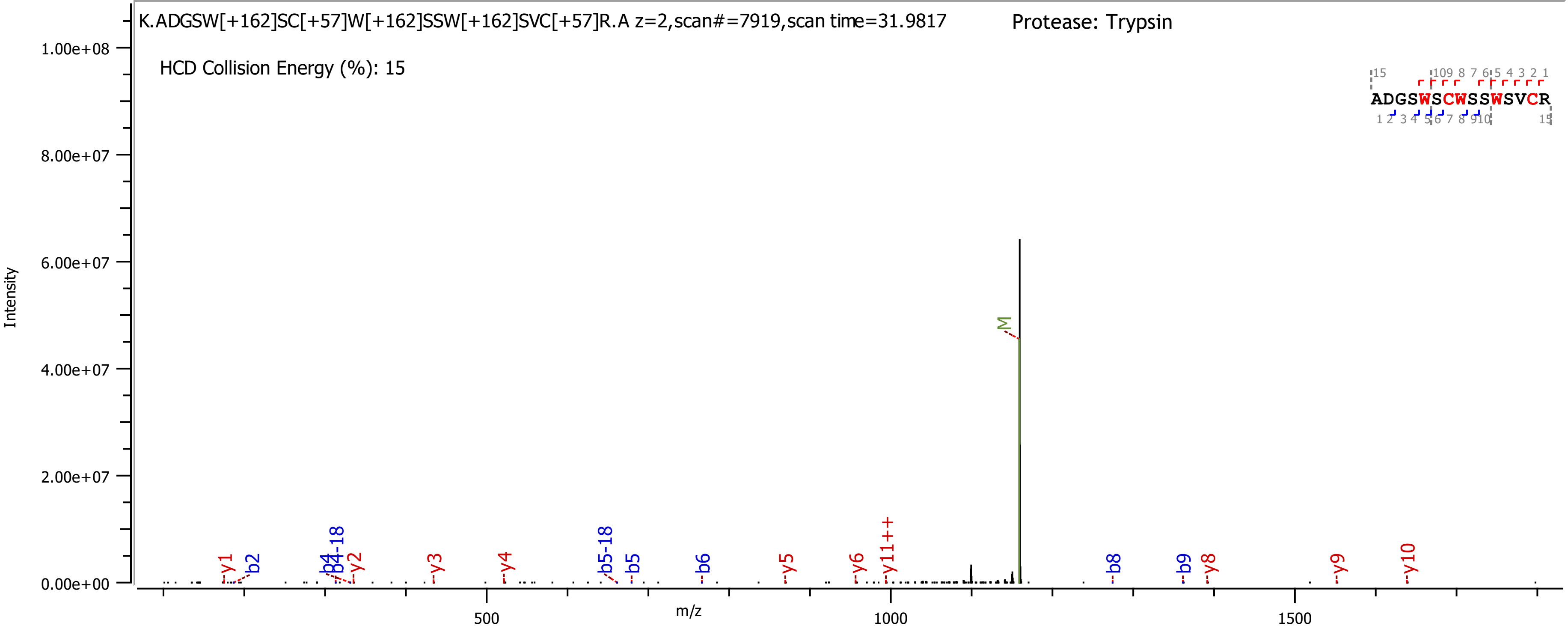
K.ADGSW[+162]SC[+57]W[+162]SSW[+162]SVC[+57]R.A z=2,scan#=7919,scan time=31.9817
Protease: Trypsin
HCD Collision Energy (%): 15
ADGSWSCWSSWSVCR
Intensity
m/z
1.00e+08
8.00e+07
6.00e+07
4.00e+07
2.00e+07
0.00e+00
500
1000
1500
M
y1
b2
b4
b4-18
y2
y3
y4
b5-18
b5
b6
y5
y6
y11++
b8
b9
y8
y9
y10

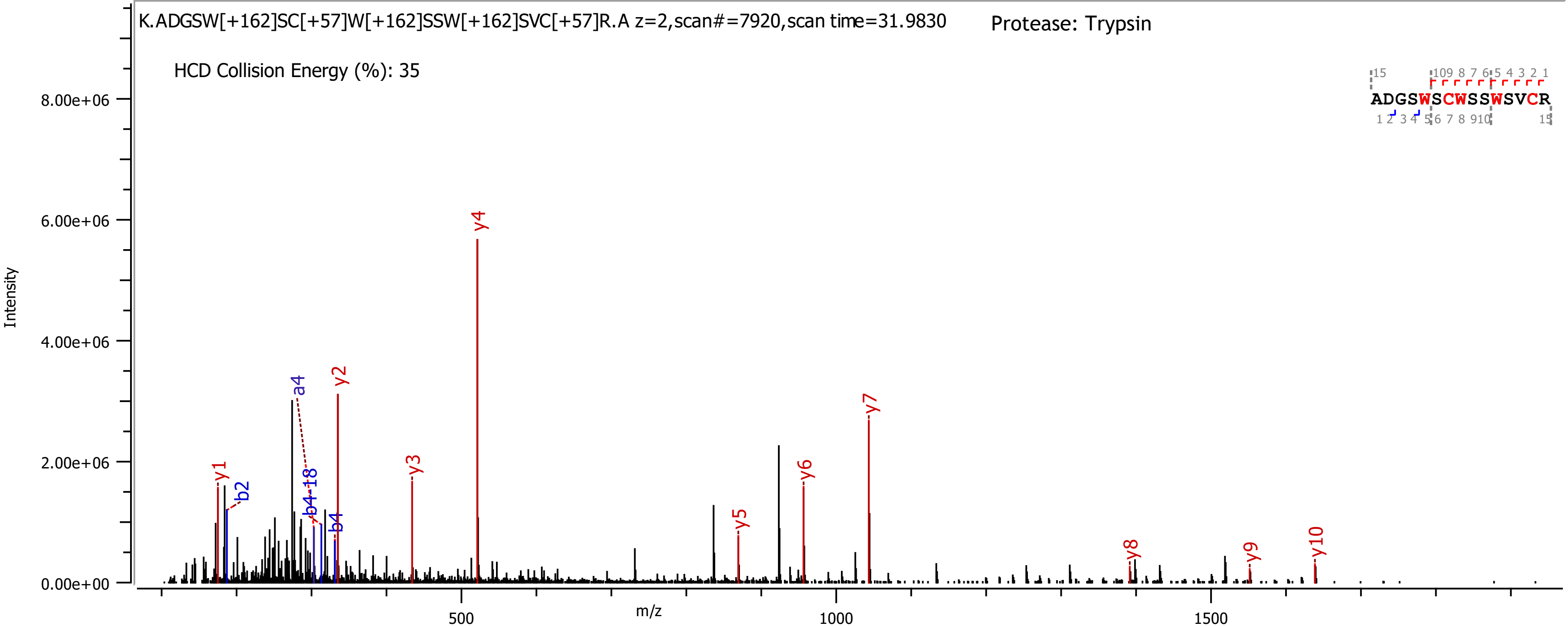
K.ADGSW[+162]SC[+57]W[+162]SSW[+162]SVC[+57]R.A z=2,scan#=7920,scan time=31.9830
Protease: Trypsin
HCD Collision Energy (%): 35
ADGSWSCWSSWSVCR
Intensity
m/z
0.00e+00
2.00e+06
4.00e+06
6.00e+06
8.00e+06
500
1000
1500
y1
b2
a4
b4-18
b4
y2
y3
y4
y5
y6
y7
y8
y9
y10

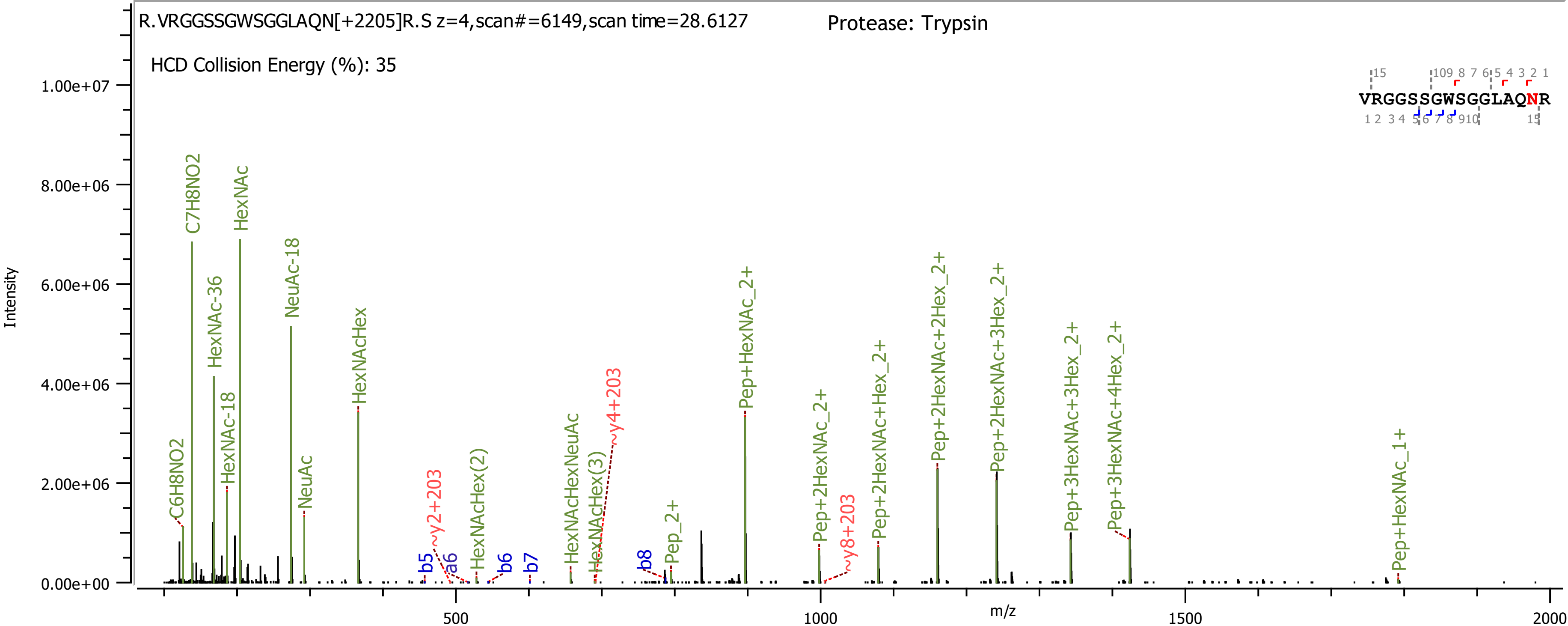

R.VRGGSSGWSGGLAQN[+2205]R.S z=4,scan#=6149,scan time=28.6127
Protease: Trypsin
HCD Collision Energy (%): 35
VRGGSSGWSGGLAQNR
Intensity
m/z
1.00e+07
8.00e+06
6.00e+06
4.00e+06
2.00e+06
0.00e+00
500
1000
1500
2000
C6H8NO2
C7H8NO2
HexNAc-36
HexNAc-18
HexNAc
NeuAc-18
NeuAc
HexNAcHex
b5
~y2+203
a6
HexNAcHex(2)
b6
b7
HexNAcHexNeuAc
HexNAcHex(3)
~y4+203
b8
Pep_2+
Pep+HexNAc_2+
Pep+2HexNAc_2+
~y8+203
Pep+2HexNAc+Hex_2+
Pep+2HexNAc+2Hex_2+
Pep+2HexNAc+3Hex_2+
Pep+3HexNAc+3Hex_2+
Pep+3HexNAc+4Hex_2+
Pep+HexNAc_1+

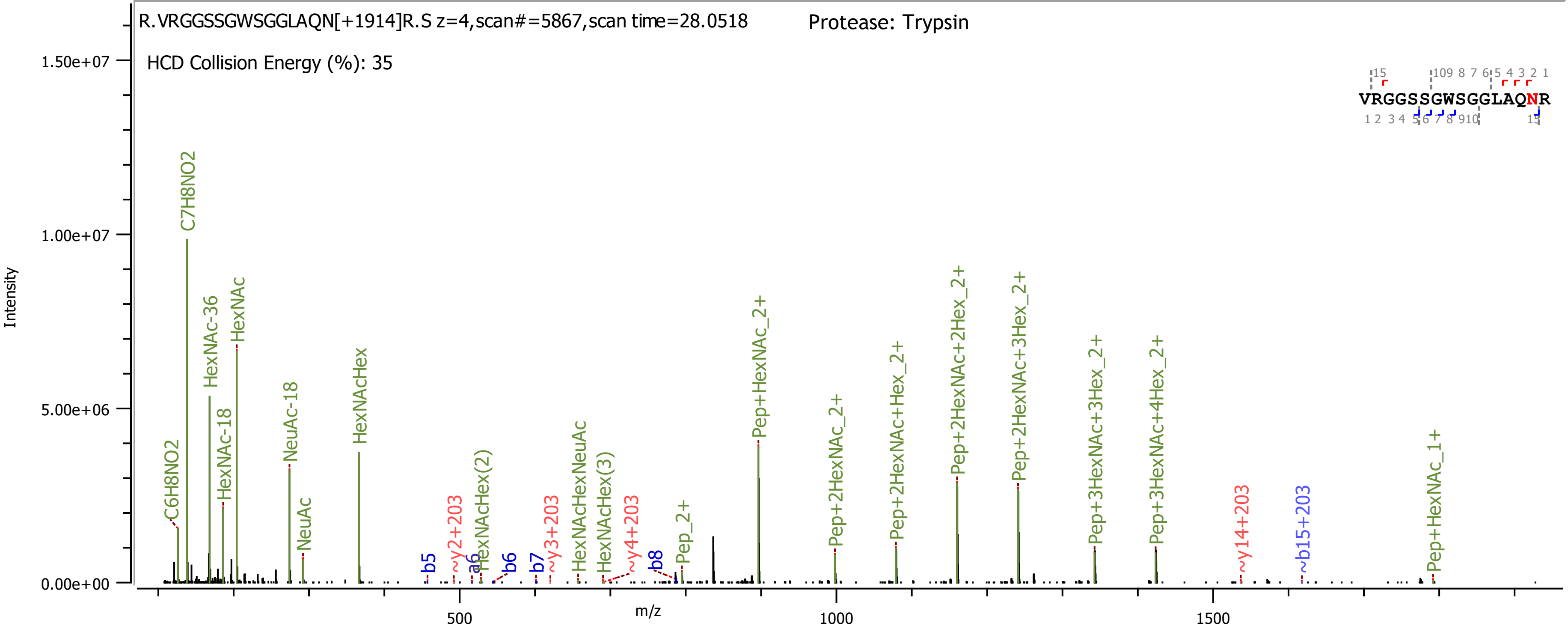

R.VRGGSSGWSGGLAQN[+1914]R.S z=4,scan#=5867,scan time=28.0518
Protease: Trypsin
HCD Collision Energy (%): 35
VRGGSSGWSGGLAQNR
Intensity
m/z
1.50e+07
1.00e+07
5.00e+06
0.00e+00
500
1000
1500
C6H8NO2
C7H8NO2
HexNAc-36
HexNAc-18
HexNAc
NeuAc-18
NeuAc
HexNAcHex
b5
~y2+203
a6
HexNAcHex(2)
b6
b7
~y3+203
HexNAcHexNeuAc
HexNAcHex(3)
~y4+203
b8
Pep_2+
Pep+HexNAc_2+
Pep+2HexNAc_2+
Pep+2HexNAc+Hex_2+
Pep+2HexNAc+2Hex_2+
Pep+2HexNAc+3Hex_2+
Pep+3HexNAc+3Hex_2+
Pep+3HexNAc+4Hex_2+
~y14+203
~b15+203
Pep+HexNAc_1+

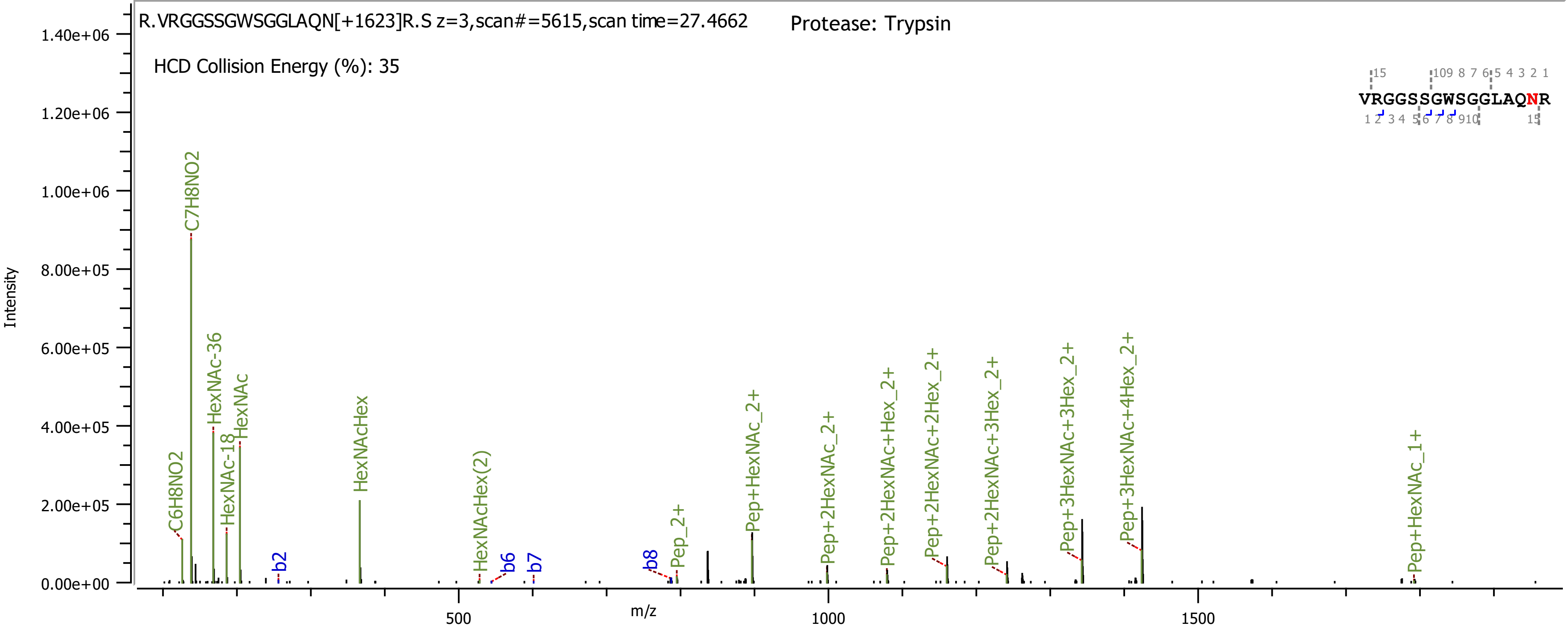
R.VRGGSSGWSGGLAQN[+1623]R.S z=3,scan#=5615,scan time=27.4662
Protease: Trypsin
HCD Collision Energy (%): 35
VRGGSSGWSGGLAQNR
Intensity
m/z
C7H8NO2
C6H8NO2
HexNAc-36
HexNAc-18
HexNAc
HexNAcHex
HexNAcHex(2)
b2
b6
b7
b8
Pep_2+
Pep+HexNAc_2+
Pep+2HexNAc_2+
Pep+2HexNAc+Hex_2+
Pep+2HexNAc+2Hex_2+
Pep+2HexNAc+3Hex_2+
Pep+3HexNAc+3Hex_2+
Pep+3HexNAc+4Hex_2+
Pep+HexNAc_1+

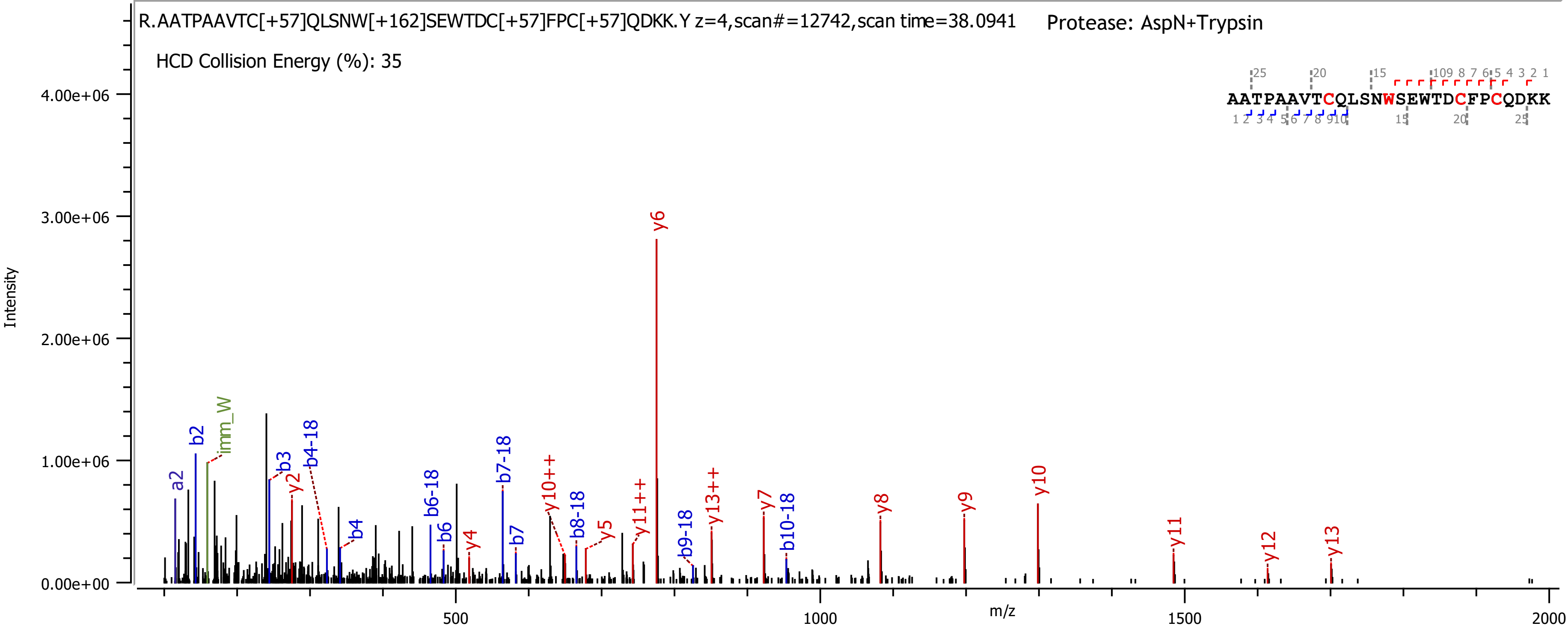
R.AATPAAVTC[+57]QLSNW[+162]SEWTDC[+57]FPC[+57]QDKK.Y z=4,scan#=12742,scan time=38.0941
Protease: AspN+Trypsin
HCD Collision Energy (%): 35
AATPAAVTCQLSNWSEWTDCFPCQDKK
Intensity
m/z
a2
b2
imm_W
b3
y2
b4-18
b4
b6-18
b6
y4
b7-18
b7
y10++
b8-18
y5
y11++
y6
b9-18
y13++
y7
b10-18
y8
y9
y10
y11
y12
y13

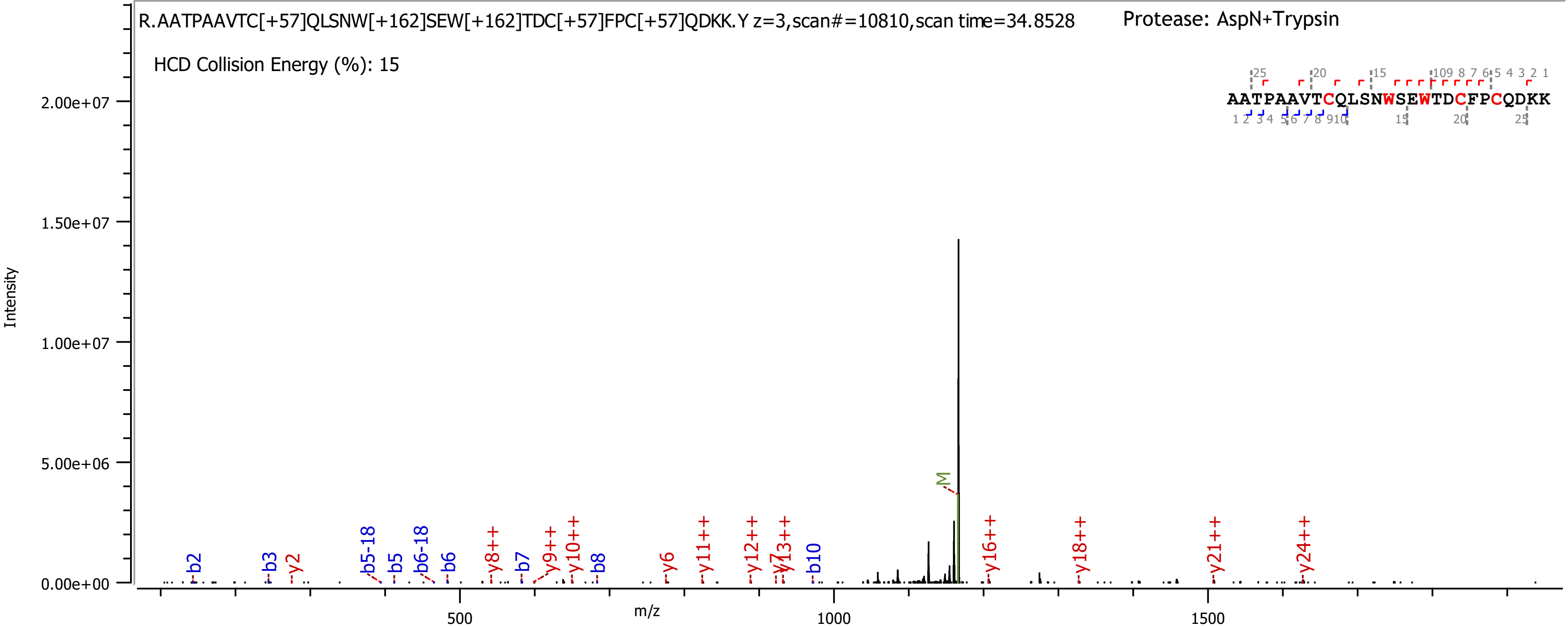
R.AATPAAVTC[+57]QLSNW[+162]SEW[+162]TDC[+57]FPC[+57]QDKK.Y z=3,scan#=10810,scan time=34.8528
Protease: AspN+Trypsin
HCD Collision Energy (%): 15
AATPAAVTCQLSNWSEWTDCFPCQDKK
Intensity
m/z
b2 b3 y2 b5-18 b5 b6-18 b6 y8++ b7 y9++ y10++ b8 y6 y11++ y12++ y7 y13++ b10 M y16++ y18++ y21++ y24++

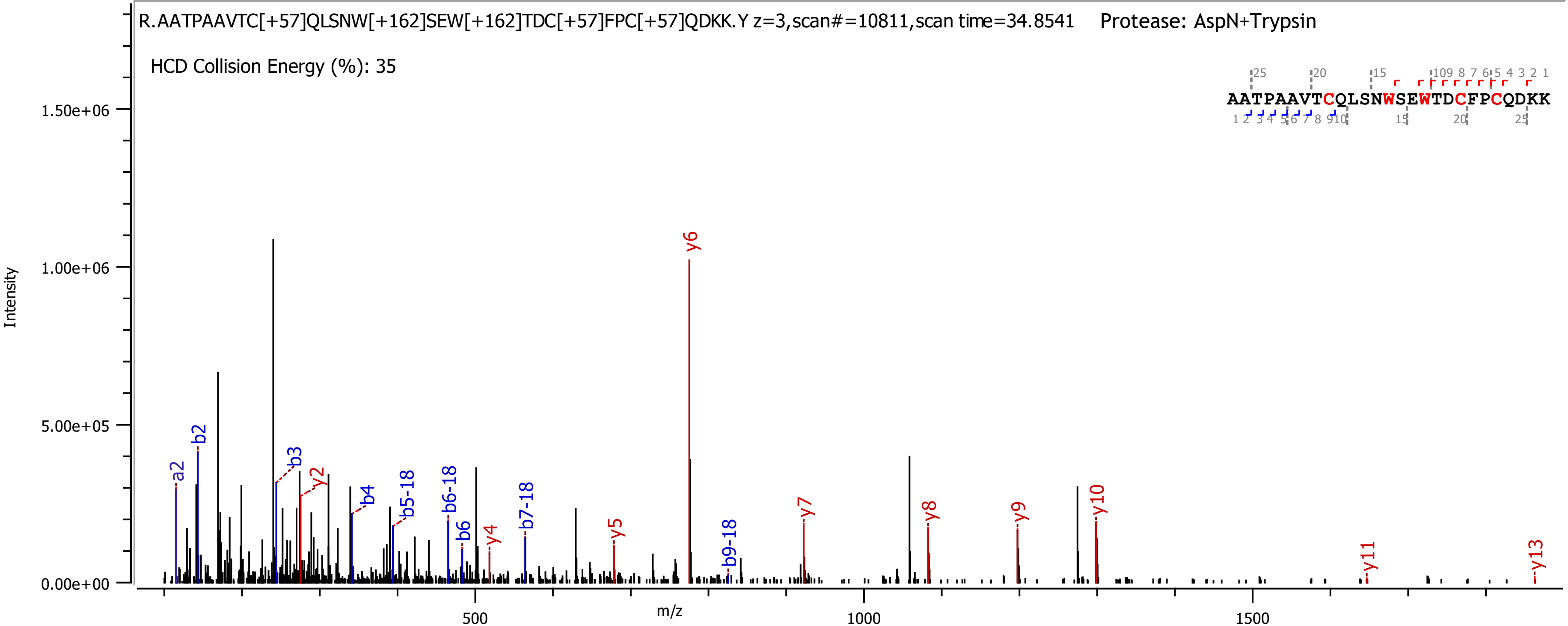
R.AATPAAVTC[+57]QLSNW[+162]SEW[+162]TDC[+57]FPC[+57]QDKK.Y z=3,scan#=10811,scan time=34.8541
Protease: AspN+Trypsin
HCD Collision Energy (%): 35
AATPAAVTCQLSNWSEWTDCFPCQDKK
Intensity
m/z
0.00e+00
5.00e+05
1.00e+06
1.50e+06
500
1000
1500
a2
b2
b3
y2
b4
b5-18
b6-18
b6
y4
b7-18
y5
y6
b9-18
y7
y8
y9
y10
y11
y13

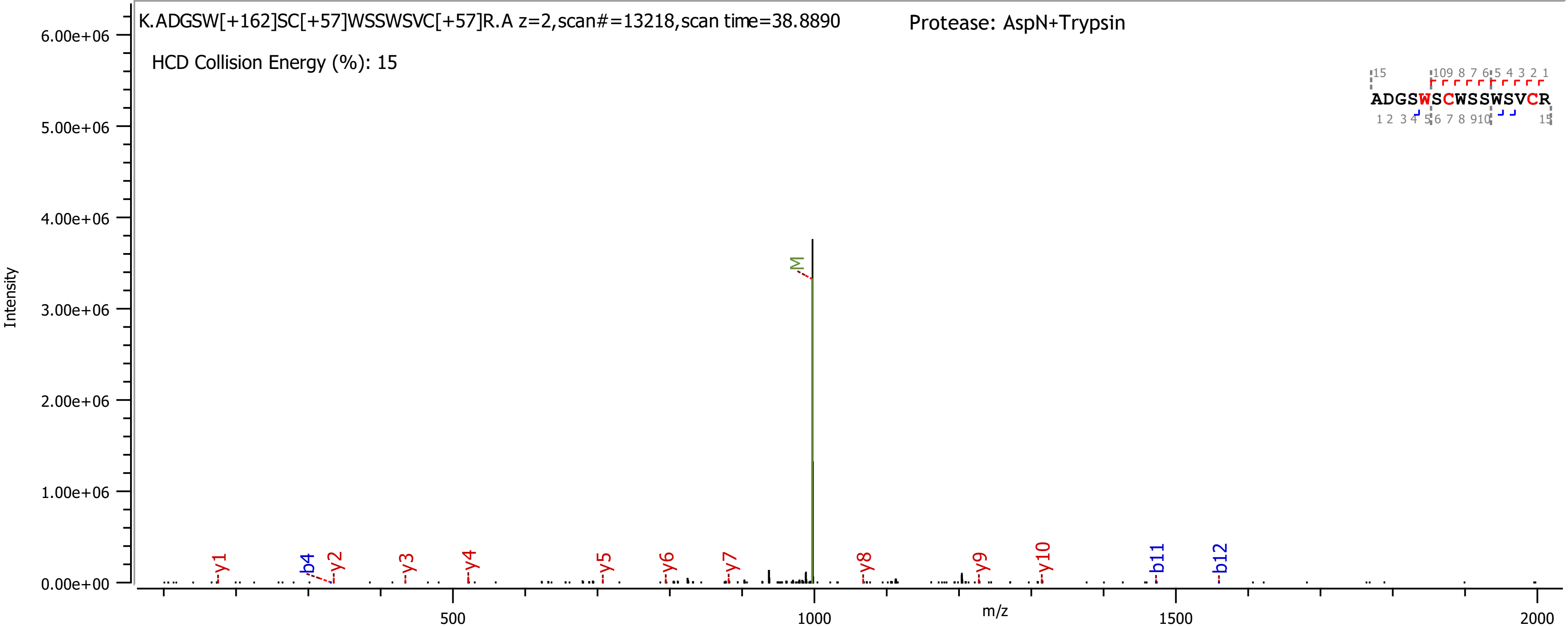

K.ADGSW[+162]SC[+57]WSSWSVC[+57]R.A z=2,scan#=13218,scan time=38.8890
Protease: AspN+Trypsin
HCD Collision Energy (%): 15
ADGSWSCWSSWSVCR
Intensity
m/z
6.00e+06
5.00e+06
4.00e+06
3.00e+06
2.00e+06
1.00e+06
0.00e+00
500
1000
1500
2000
M
y1
b4
y2
y3
y4
y5
y6
y7
y8
y9
y10
b11
b12

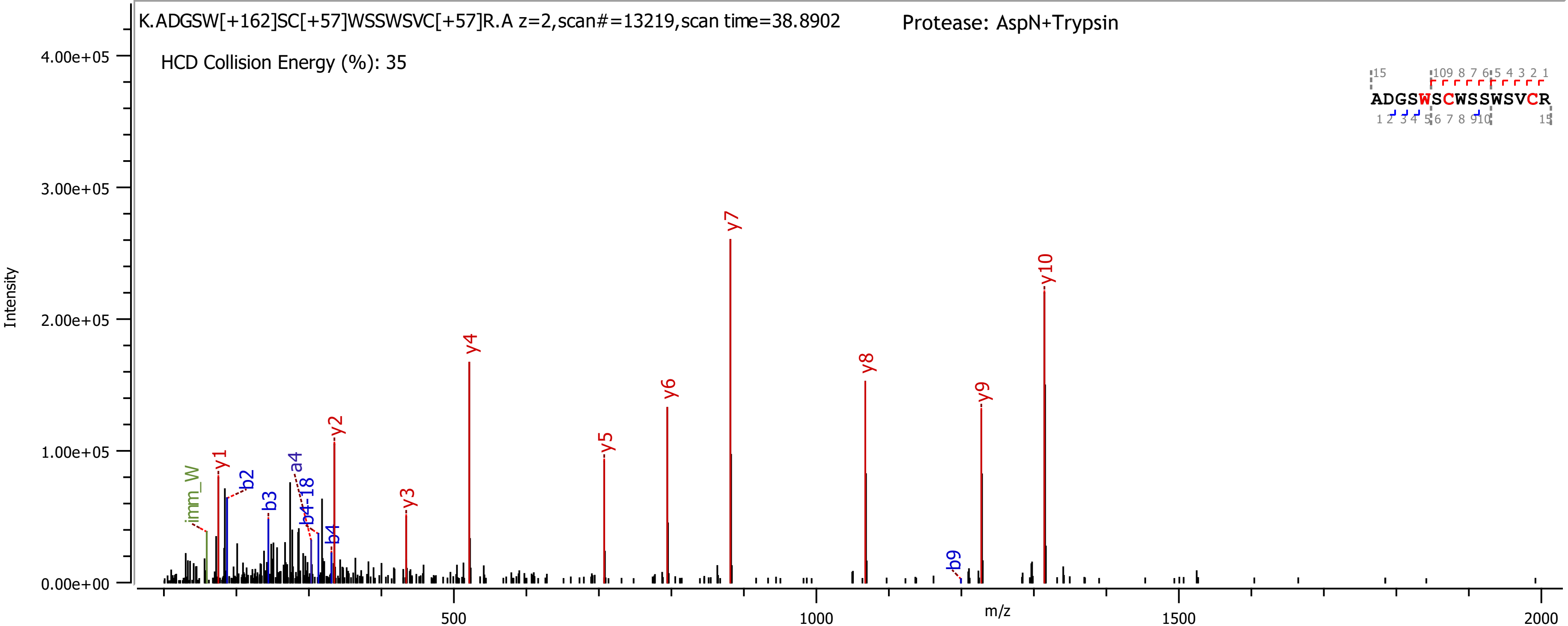
K.ADGSW[+162]SC[+57]WSSWSVC[+57]R.A z=2,scan#=13219,scan time=38.8902
Protease: AspN+Trypsin
HCD Collision Energy (%): 35
ADGSWSCWSSWSVCR
Intensity
m/z
imm_W
y1
b2
b3
a4
b4-18
b4
y2
y3
y4
y5
y6
y7
y8
b9
y9
y10

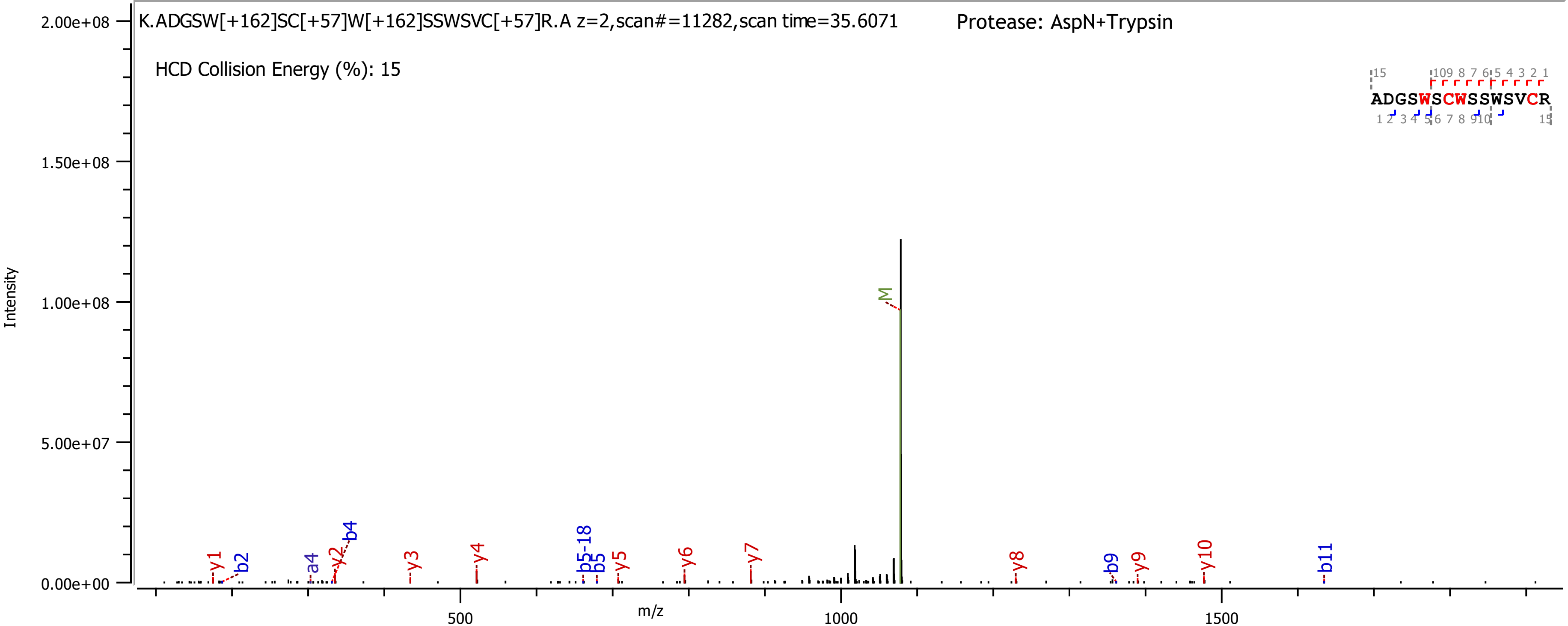

K.ADGSW[+162]SC[+57]W[+162]SSWSVC[+57]R.A z=2,scan#=11282,scan time=35.6071
Protease: AspN+Trypsin
HCD Collision Energy (%): 15
ADGSWSCWSSWSVCR
Intensity
m/z
2.00e+08
1.50e+08
1.00e+08
5.00e+07
0.00e+00
500
1000
1500
M
y1
b2
a4
y2
b4
y3
y4
b5-18
b5
y5
y6
y7
y8
b9
y9
y10
b11

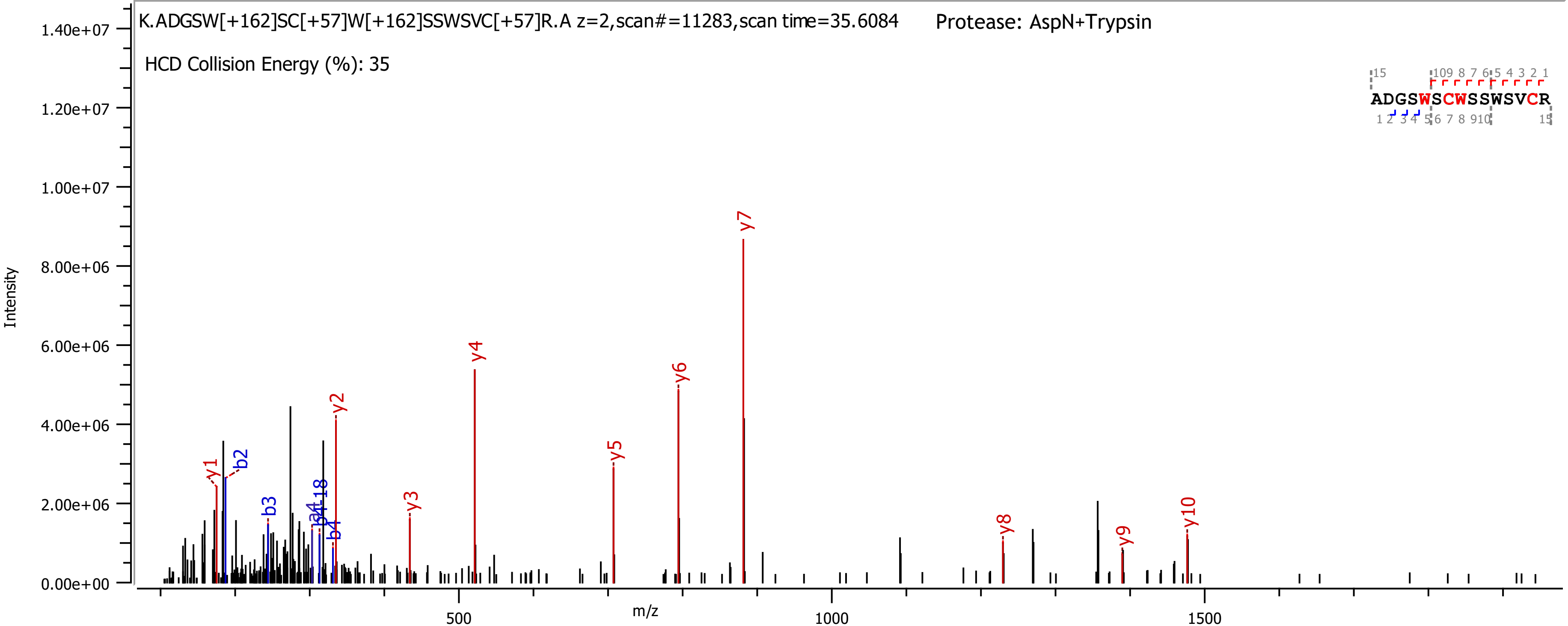

K.ADGSW[+162]SC[+57]W[+162]SSWSVC[+57]R.A z=2,scan#=11283,scan time=35.6084
Protease: AspN+Trypsin
HCD Collision Energy (%): 35
ADGSWSCWSSWSVCR
Intensity
m/z
y1
b2
b3
a4
b4-18
b4
y2
y3
y4
y5
y6
y7
y8
y9
y10
0.00e+00
2.00e+06
4.00e+06
6.00e+06
8.00e+06
1.00e+07
1.20e+07
1.40e+07
500
1000
1500

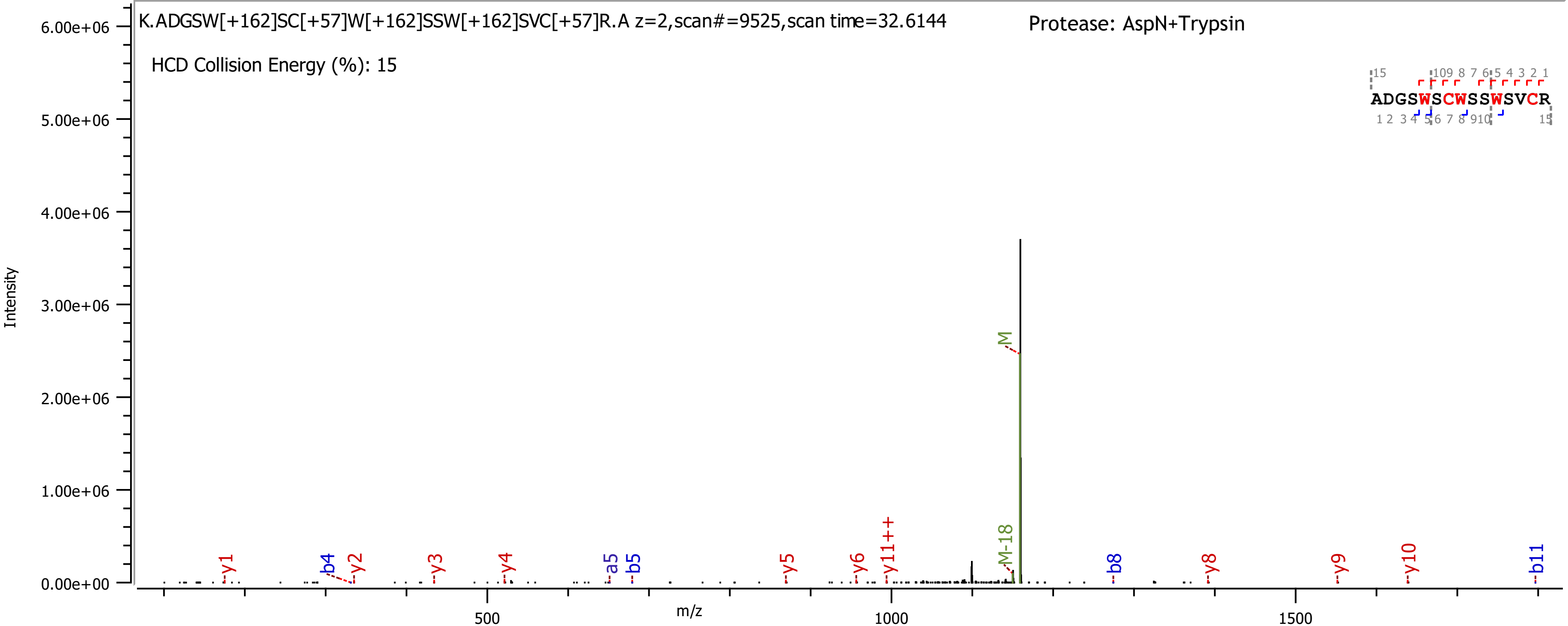

K.ADGSW[+162]SC[+57]W[+162]SSW[+162]SVC[+57]R.A z=2,scan#=9525,scan time=32.6144
Protease: AspN+Trypsin
HCD Collision Energy (%): 15
ADGSWSCWSSWSVCR
Intensity
m/z
M
M-18
y1
b4
y2
y3
y4
a5
b5
y5
y6
y11++
b8
y8
y9
y10
b11

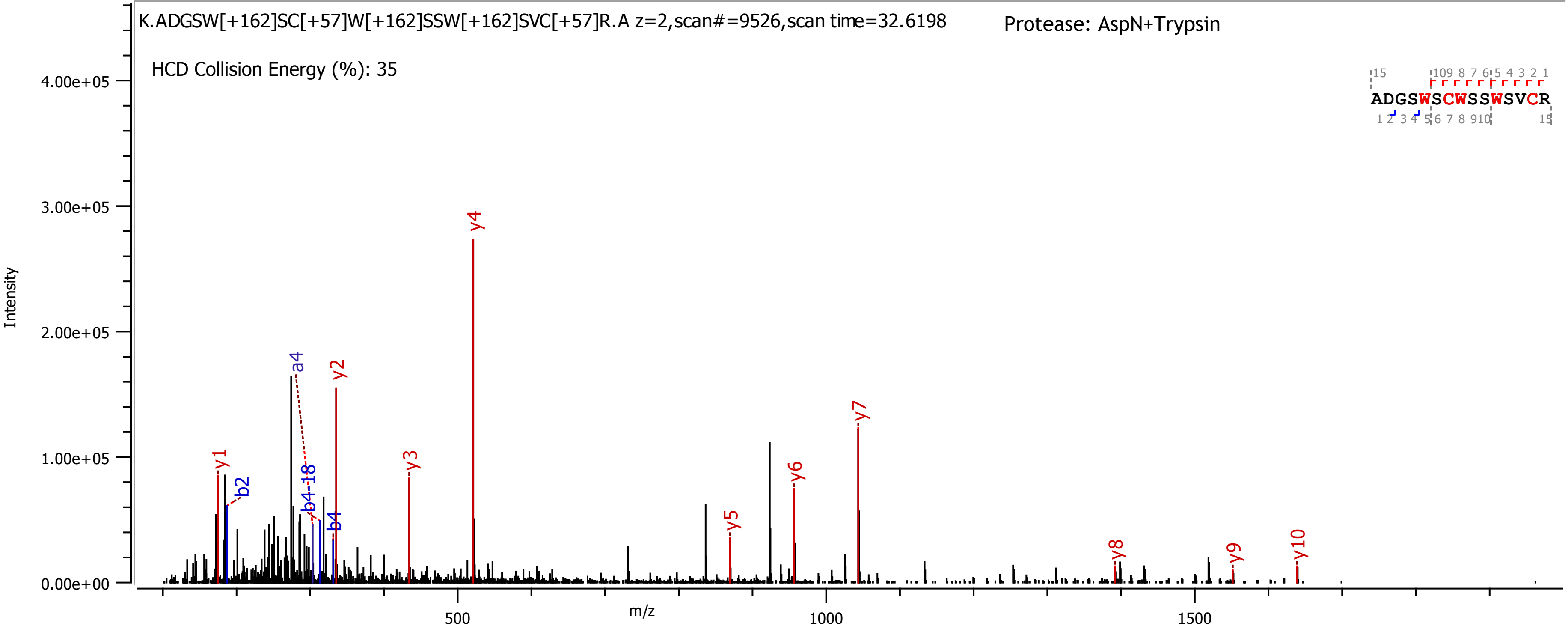

K.ADGSW[+162]SC[+57]W[+162]SSW[+162]SVC[+57]R.A z=2,scan#=9526,scan time=32.6198
Protease: AspN+Trypsin
HCD Collision Energy (%): 35
ADGSWSCWSSWSVCR
Intensity
m/z
4.00e+05
3.00e+05
2.00e+05
1.00e+05
0.00e+00
500
1000
1500
y1
b2
a4
b4-18
b4
y2
y3
y4
y5
y6
y7
y8
y9
y10

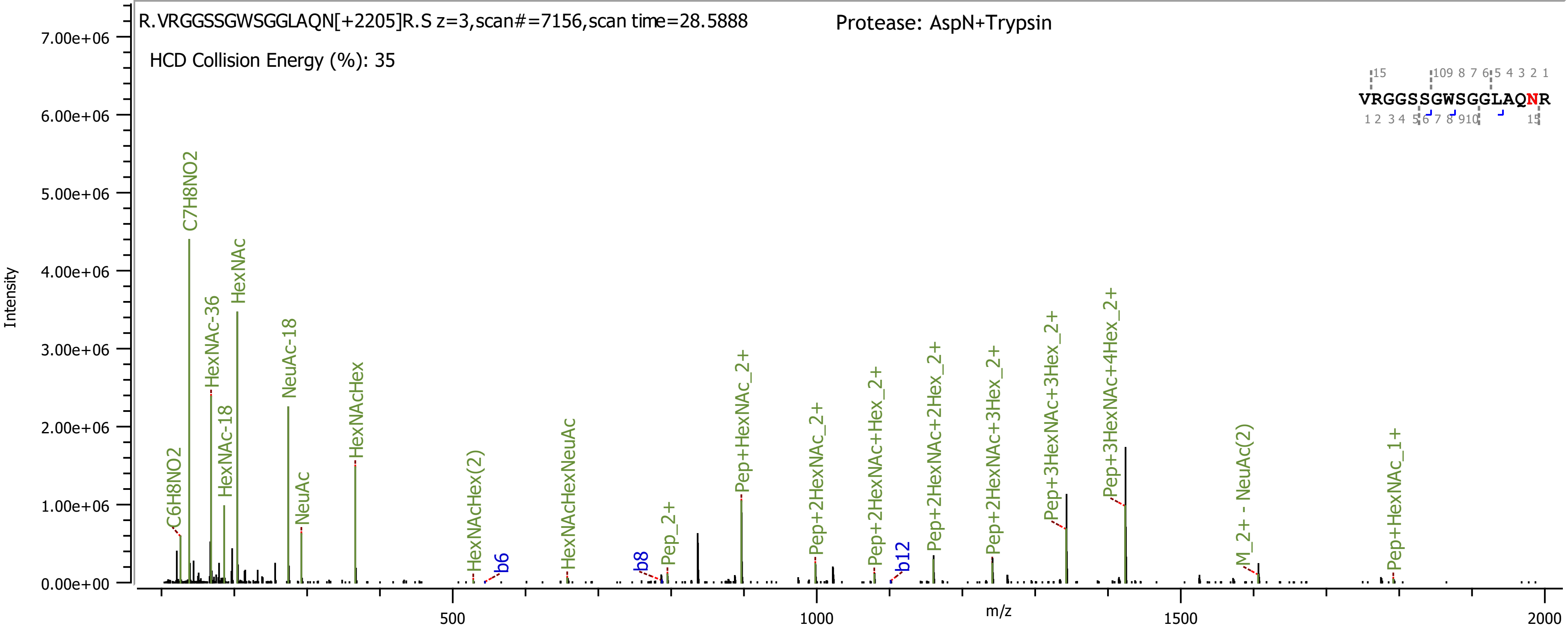
R.VRGGSSGWSGGLAQN[+2205]R.S z=3,scan#=7156,scan time=28.5888
Protease: AspN+Trypsin
HCD Collision Energy (%): 35
VRGGSSGWSGGLAQNR
Intensity
m/z
C6H8NO2
C7H8NO2
HexNAc-36
HexNAc-18
HexNAc
NeuAc-18
NeuAc
HexNAcHex
HexNAcHex(2)
b6
HexNAcHexNeuAc
b8
Pep_2+
Pep+HexNAc_2+
Pep+2HexNAc_2+
Pep+2HexNAc+Hex_2+
b12
Pep+2HexNAc+2Hex_2+
Pep+2HexNAc+3Hex_2+
Pep+3HexNAc+3Hex_2+
Pep+3HexNAc+4Hex_2+
M_2+ - NeuAc(2)
Pep+HexNAc_1+

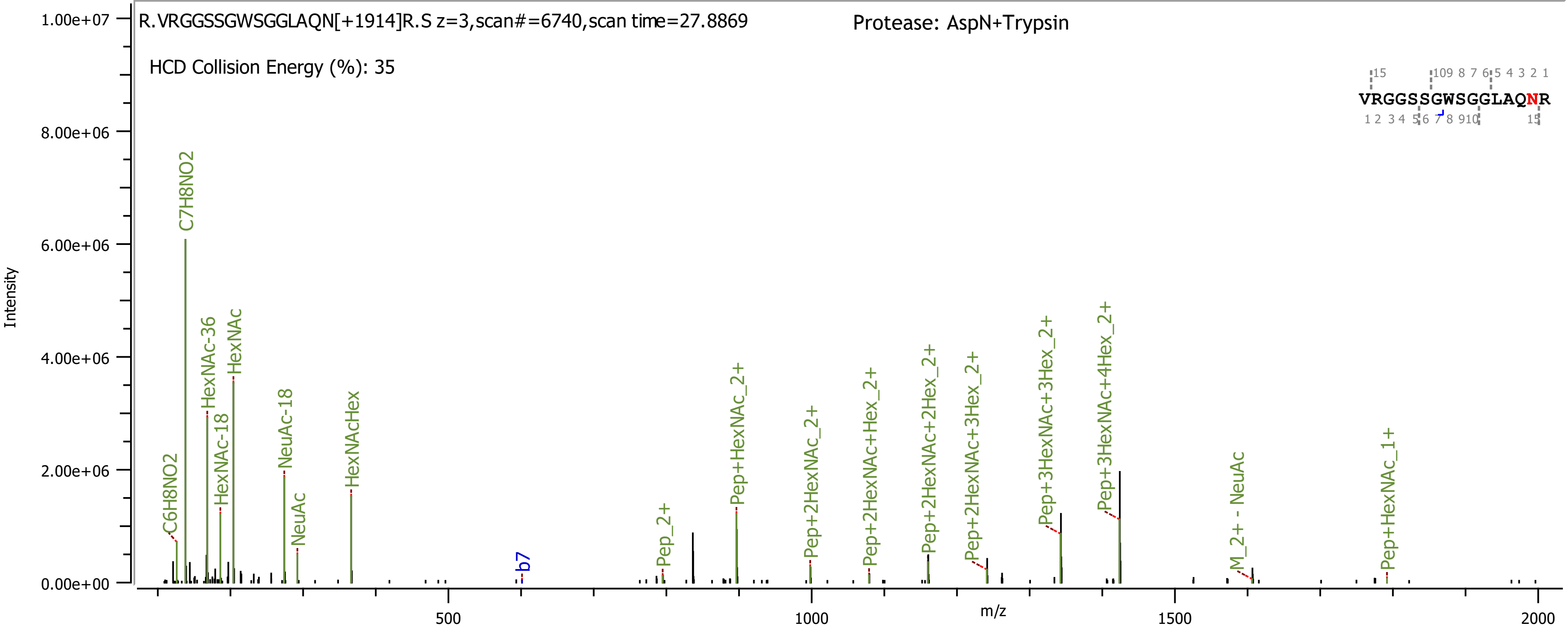

R.VRGGSSGWSGGLAQN[+1914]R.S z=3,scan#=6740,scan time=27.8869
Protease: AspN+Trypsin
HCD Collision Energy (%): 35
VRGGSSGWSGGLAQNR
Intensity
m/z
C7H8NO2
C6H8NO2
HexNAc-36
HexNAc-18
HexNAc
NeuAc-18
NeuAc
HexNAcHex
b7
Pep_2+
Pep+HexNAc_2+
Pep+2HexNAc_2+
Pep+2HexNAc+Hex_2+
Pep+2HexNAc+2Hex_2+
Pep+2HexNAc+3Hex_2+
Pep+3HexNAc+3Hex_2+
Pep+3HexNAc+4Hex_2+
M_2+ - NeuAc
Pep+HexNAc_1+

C8 beta subunit: MS/MS spectra of all PTM modified peptides

EThcD spectra

Glycosylation Site

TSR1_WSSW	SVDVTLMPIDCELSSWSSWTTCDPCQK<br>SVDVTLMPIDCELSSWSSWTTCDPCQK<br>SVDVTLMPIDCELSSWSSWTTCDPCQK<br>SVDVTLMPIDCELSSWSSWTTCDPCQK
TSR2_WNCWSNW	NTPIDGKWNCWSNWSSCSGR<br>NTPIDGKWNCWSNWSSCSGR<br>NTPIDGKWNCWSNWSSCSGR<br>NTPIDGKWNCWSNWSSCSGR
N101	YAYLLQPSQFHGEPCNFSDKEVEDCVTNRPCR<br>YAYLLQPSQFHGEPCN[+1216.4]FSDKEVEDCVTNRPCR<br>YAYLLQPSQFHGEPCN[+1702.6]FSDKEVEDCVTNRPCR
N243	EYESYSDFERN[+2204.8]VTEK

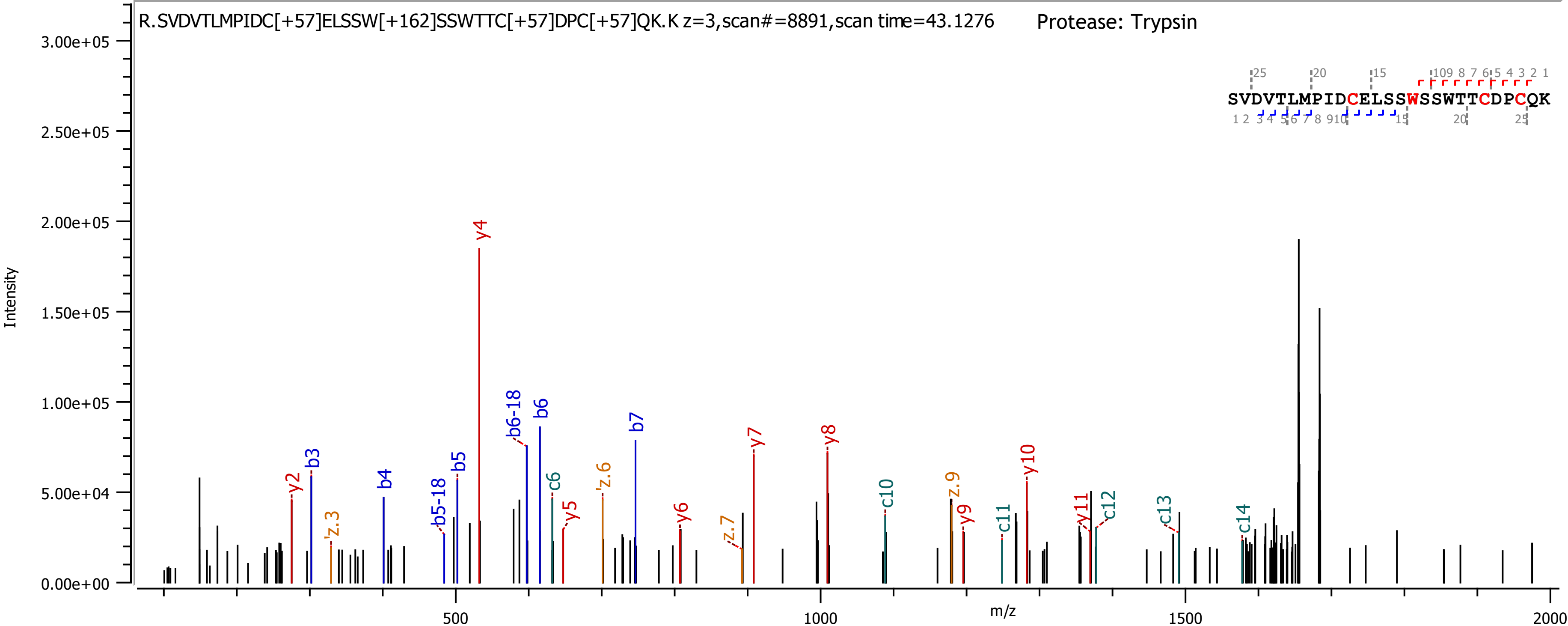
R.SVDVTLMPIDC[+57]ELSSW[+162]SSWTTC[+57]DPC[+57]QK.K z=3,scan#=8891,scan time=43.1276
Protease: Trypsin
SVDVTLMPIDCELSSWSSWTTCDPCQK
Intensity
m/z
3.00e+05
2.50e+05
2.00e+05
1.50e+05
1.00e+05
5.00e+04
0.00e+00
500
1000
1500
2000
y2
b3
z.3
b4
b5-18
b5
y4
b6-18
b6
c6
y5
z.6
b7
y6
z.7
y7
y8
c10
z.9
y9
c11
y10
y11
c12
c13
c14

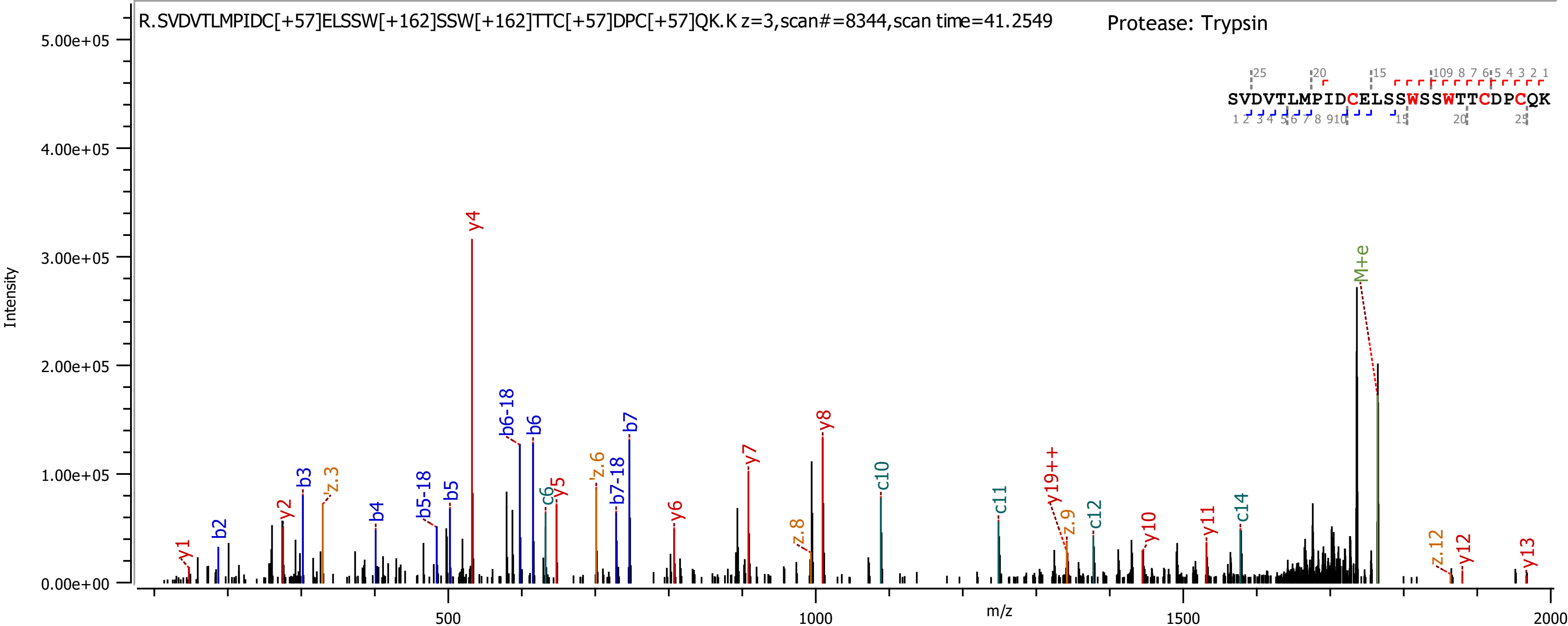

R.SVDVTLMPIDC[+57]ELSSW[+162]SSW[+162]TTC[+57]DPC[+57]QK.K z=3,scan#=8344,scan time=41.2549
Protease: Trypsin
SVDVTLMPIDCELSSWSSWTTCDPCQK
Intensity
m/z
0.00e+00
1.00e+05
2.00e+05
3.00e+05
4.00e+05
5.00e+05
500
1000
1500
2000
y1
b2
y2
b3
z.3
b4
b5-18
b5
y4
b6-18
b6
c6
y5
z.6
b7-18
b7
y6
y7
z.8
y8
c10
c11
y19++
z.9
c12
y10
y11
c14
M+e
z.12
y12
y13

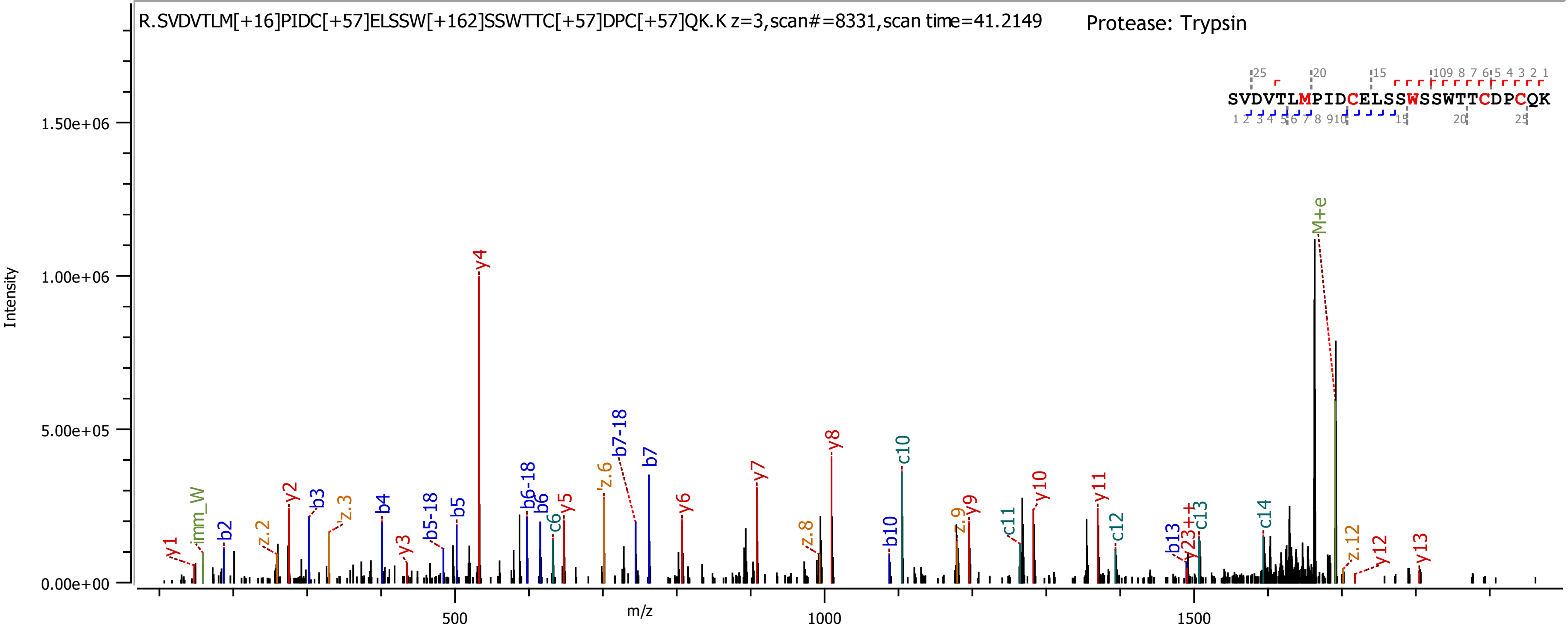

R.SVDVTLM[+16]PIDC[+57]ELSSW[+162]SSWTTC[+57]DPC[+57]QK.K z=3,scan#=8331,scan time=41.2149
Protease: Trypsin
SVDVTLMPIDCELSSWSSWTTCDPCQK
Intensity
m/z

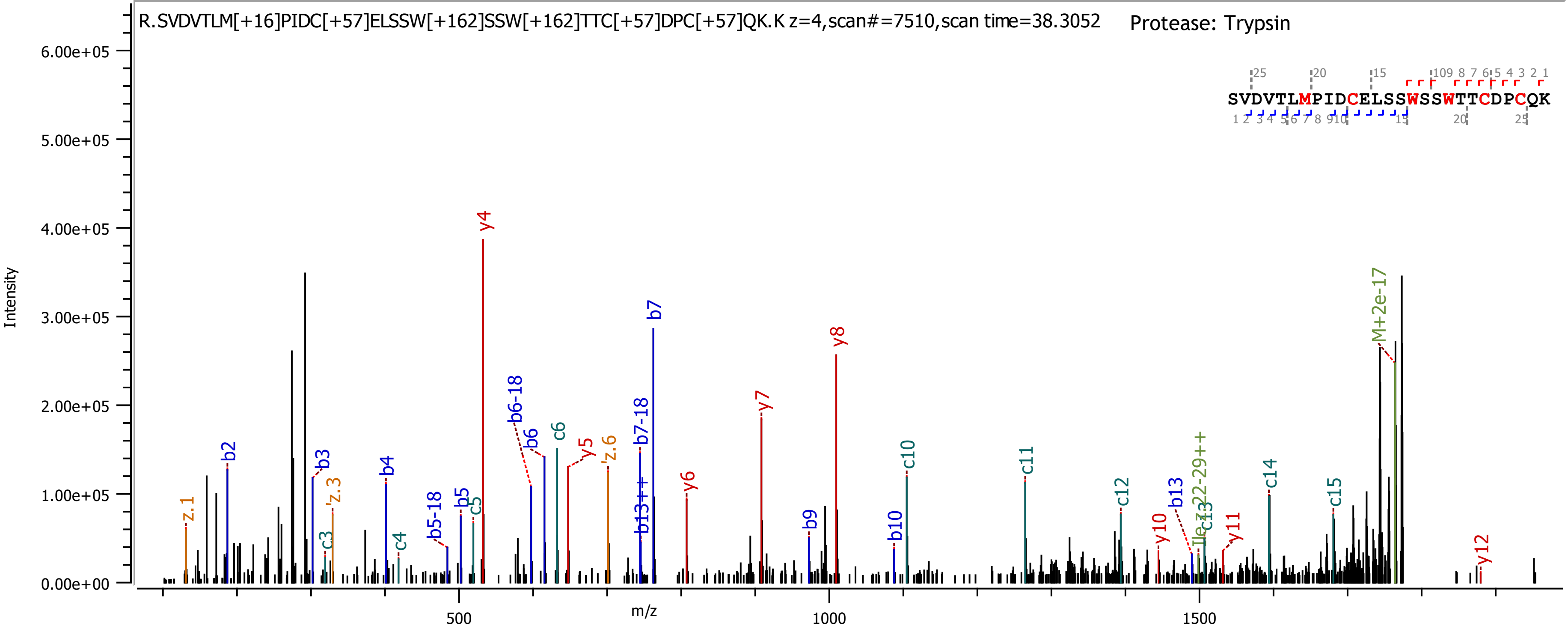

R.SVDVTLM[+16]PIDC[+57]ELSSW[+162]SSW[+162]TTC[+57]DPC[+57]QK.K z=4,scan#=7510,scan time=38.3052
Protease: Trypsin
SVDVTLMPIDCELSSWSSWTTCDPCQK
Intensity
m/z
6.00e+05
5.00e+05
4.00e+05
3.00e+05
2.00e+05
1.00e+05
0.00e+00
500
1000
1500
z.1
b2
c3
b3
'z.3
b4
c4
b5-18
b5
c5
y4
b6-18
b6
c6
y5
'z.6
b13++
b7-18
b7
y6
y7
b9
y8
b10
c10
c11
c12
y10
b13
Ile-z3
22-29++
c13
y11
c14
c15
M+2e-17
y12

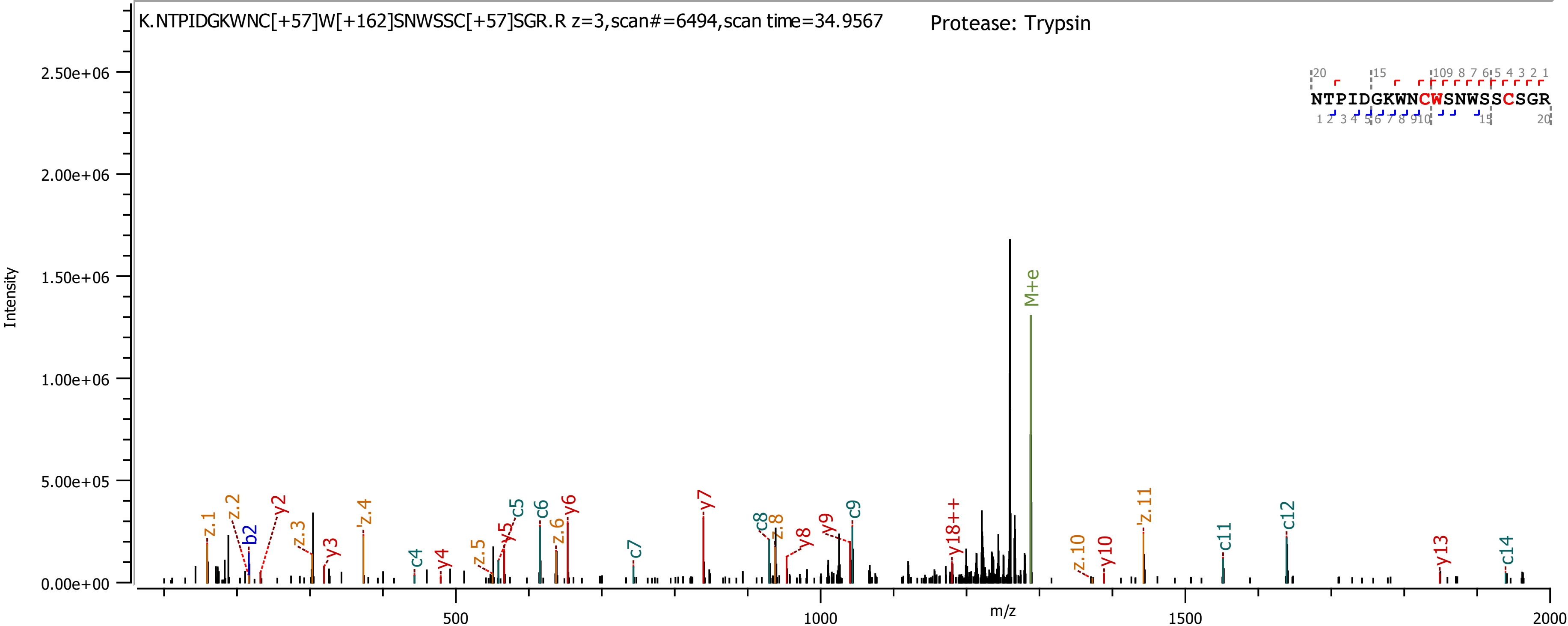
K.NTPIDGKWNC[+57]W[+162]SNWSSC[+57]SGR.R z=3,scan#=6494,scan time=34.9567
Protease: Trypsin
NTPIDGKWNCWSNWSSCSGR
Intensity
m/z
M+e
z.1
z.2
b2
y2
z.3
y3
'z.4
c4
y4
z.5
y5
c5
c6
z.6
y6
c7
y7
c8
z.8
y8
y9
c9
y18++
z.10
y10
'z.11
c11
c12
y13
c14

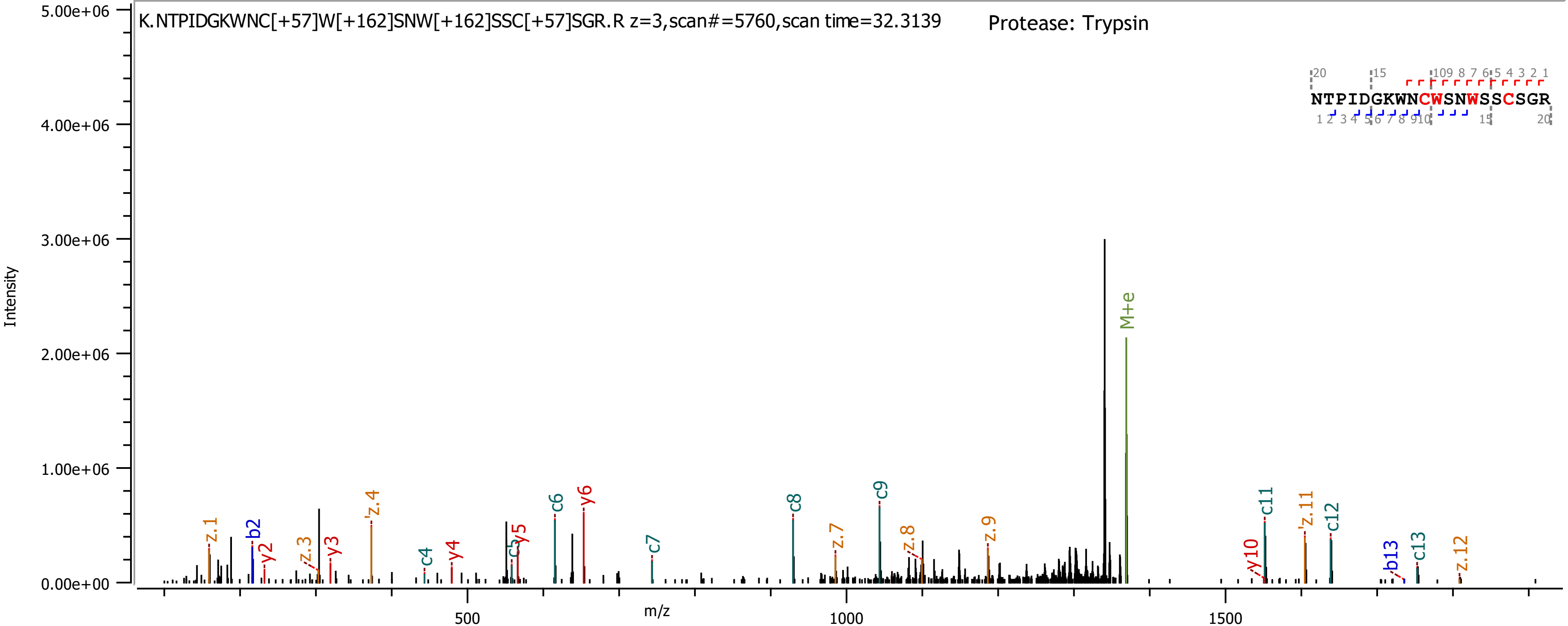

K.NTPIDGKWNC[+57]W[+162]SNW[+162]SSC[+57]SGR.R z=3,scan#=5760,scan time=32.3139
Protease: Trypsin
NTPIDGKWNCWSNWSSCSGR
Intensity
m/z
M+e

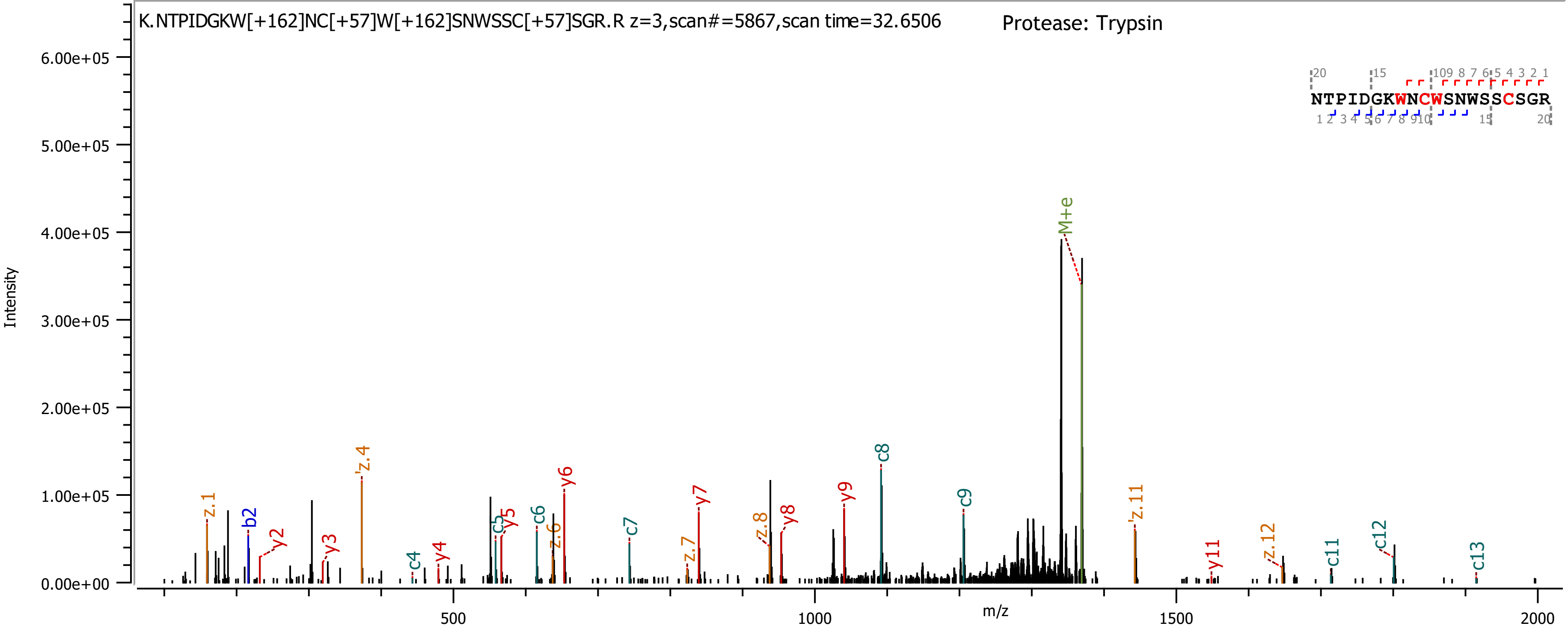

K.NTPIDGKW[+162]NC[+57]W[+162]SNWSSC[+57]SGR.R z=3,scan#=5867,scan time=32.6506
Protease: Trypsin
NTPIDGKWNCWSNWSSCSGR
Intensity
m/z
M+e

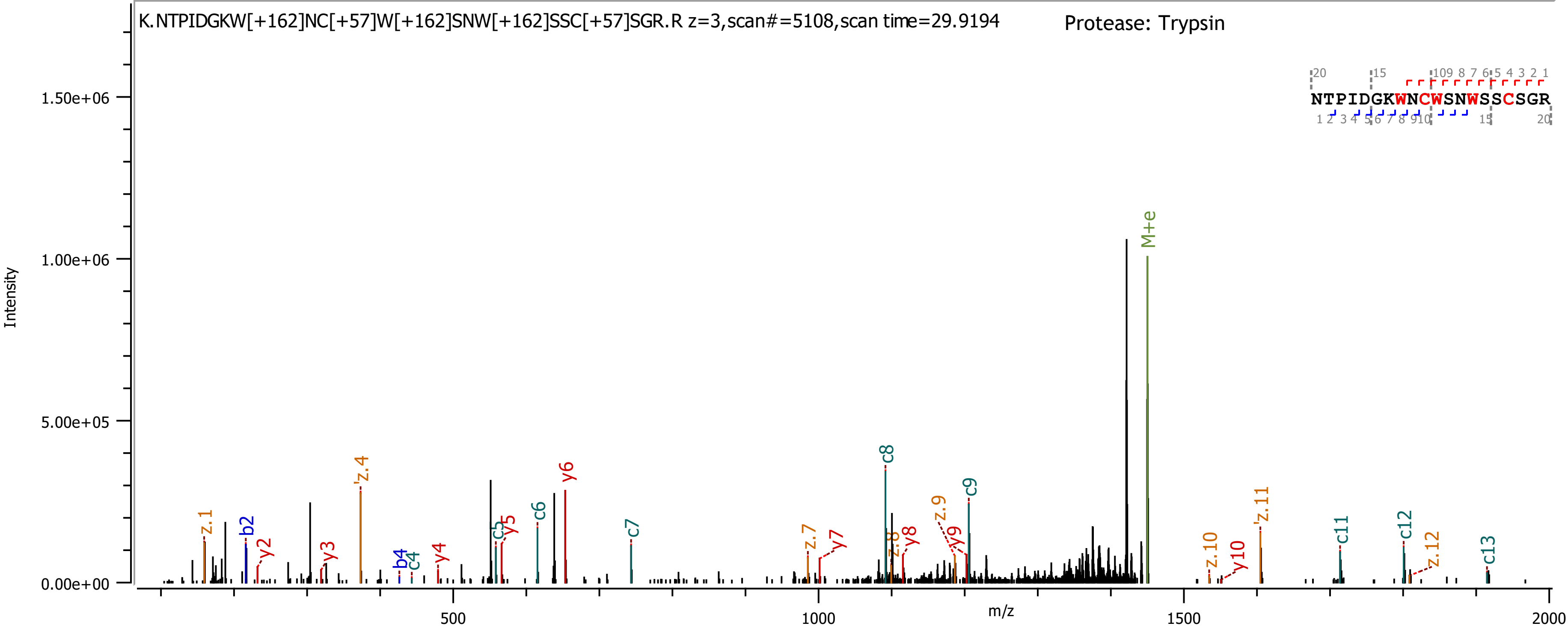

K.NTPIDGKW[+162]NC[+57]W[+162]SNW[+162]SSC[+57]SGR.R z=3,scan#=5108,scan time=29.9194
Protease: Trypsin
NTPIDGKWNCWSNWSSCSGR
Intensity
m/z
0.00e+00
5.00e+05
1.00e+06
1.50e+06
500
1000
1500
2000
M+e
c8
c9
y6
'z.4
z.1
b2
y2
y3
b4
c4
y4
c5
y5
c6
c7
z.7
y7
z.8
y8
z.9
y9
z.10
y10
'z.11
c11
c12
z.12
c13

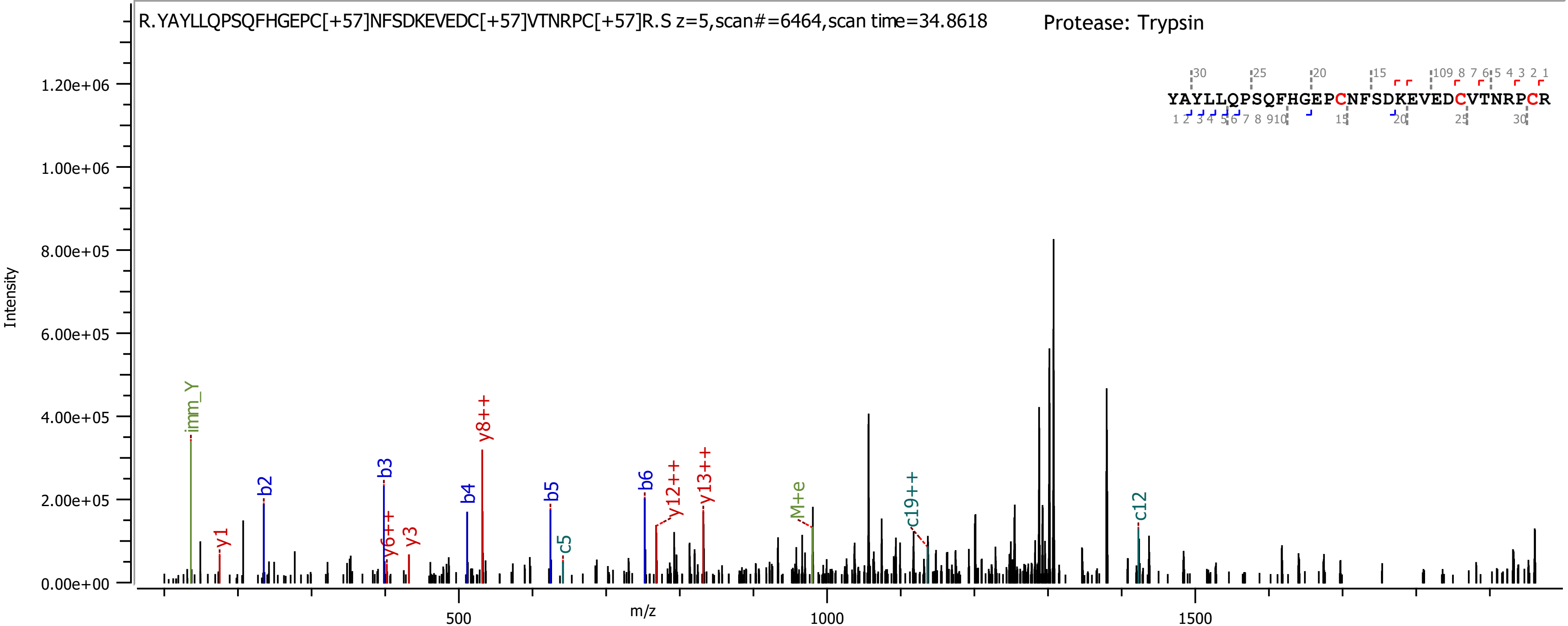

R.YAYLLQPSQFHGEPC[+57]NFSDKEVEDC[+57]VTNRPC[+57]R.S z=5,scan#=6464,scan time=34.8618
Protease: Trypsin
YAYLLQPSQFHGEPCNFSDKEVEDCVTNRPCR
Intensity
m/z
imm_Y
y1
b2
b3
y6++
y3
b4
y8++
b5
c5
b6
y12++
y13++
M+e
c19++
c12

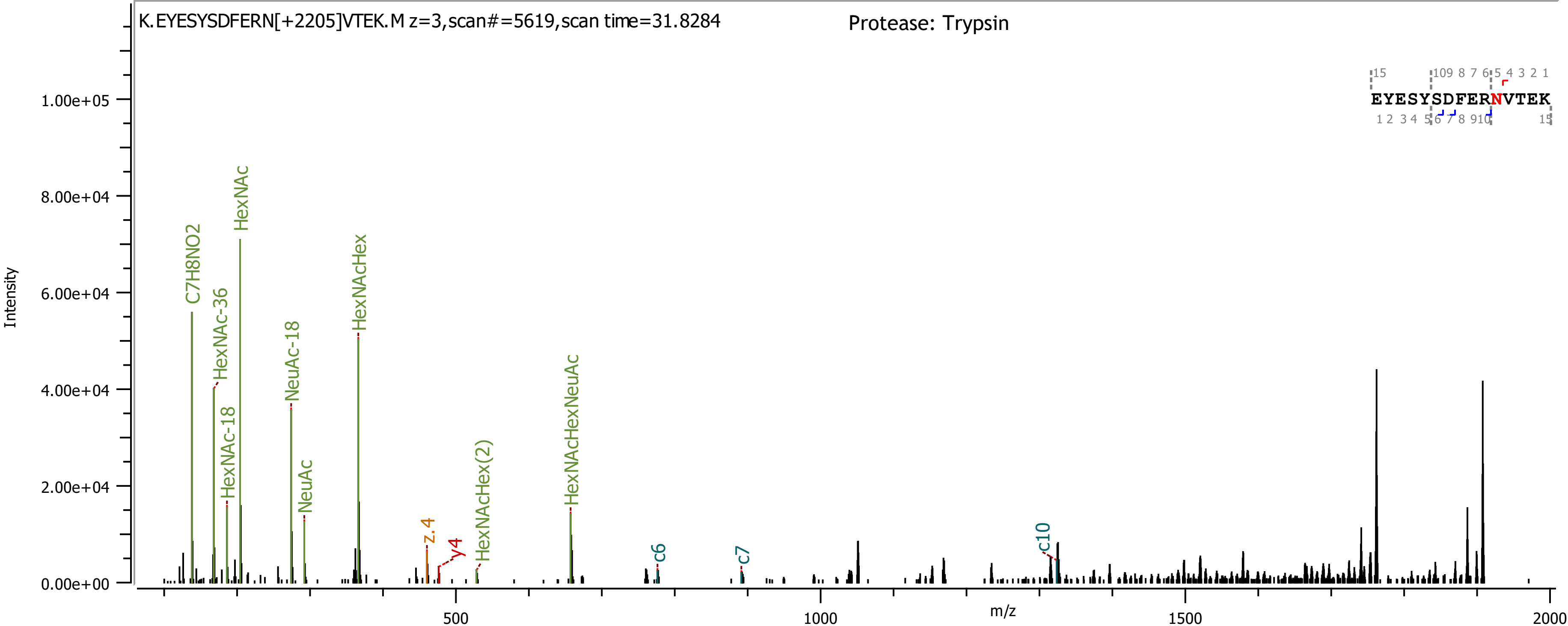
K.EYESYSDFERN[+2205]VTEK.M z=3,scan#=5619,scan time=31.8284
Protease: Trypsin
EYESYSDFERNVTEK
Intensity
m/z
C7H8NO2
HexNAc-36
HexNAc-18
HexNAc
NeuAc-18
NeuAc
HexNAcHex
z.4
y4
HexNAcHex(2)
HexNAcHexNeuAc
c6
c7
c10

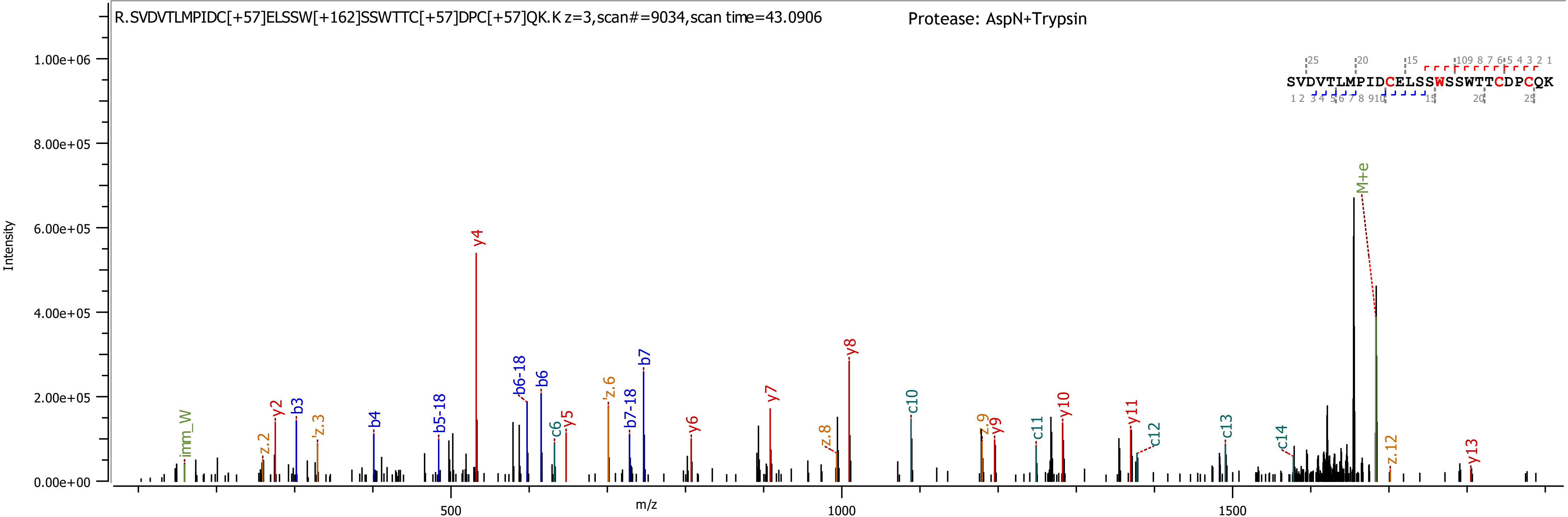
R.SVDVTLMPIDC[+57]ELSSW[+162]SSWTTC[+57]DPC[+57]QK.K z=3,scan#=9034,scan time=43.0906
Protease: AspN+Trypsin
SVDVTLMPIDCELSSWSSWTTCDPCQK
Intensity
m/z
1.00e+06
8.00e+05
6.00e+05
4.00e+05
2.00e+05
0.00e+00
500
1000
1500
imm_W
z.2
y2
b3
z.3
b4
b5-18
y4
b6-18
b6
c6
y5
z.6
b7-18
b7
y6
y7
z.8
y8
c10
z.9
y9
c11
y10
y11
c12
c13
c14
M+e
z.12
y13

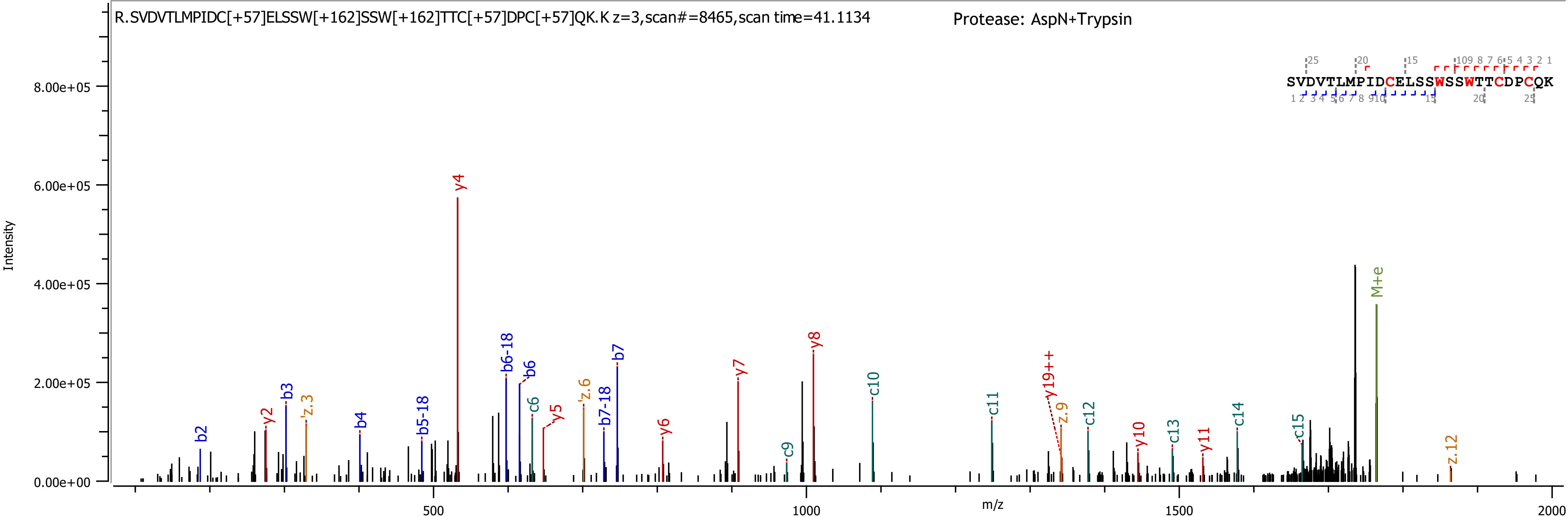
R.SVDVTLMPIDC[+57]ELSSW[+162]SSW[+162]TTC[+57]DPC[+57]QK.K z=3,scan#=8465,scan time=41.1134
Protease: AspN+Trypsin
SVDVTLMPIDCELSSWSSWTTCDPCQK
Intensity
m/z
0.00e+00
2.00e+05
4.00e+05
6.00e+05
8.00e+05
500
1000
1500
2000
b2
y2
b3
z.3
b4
b5-18
y4
b6-18
b6
c6
y5
z.6
b7-18
b7
y6
y7
c9
y8
c10
c11
y19++
z.9
c12
y10
c13
y11
c14
c15
M+e
z.12

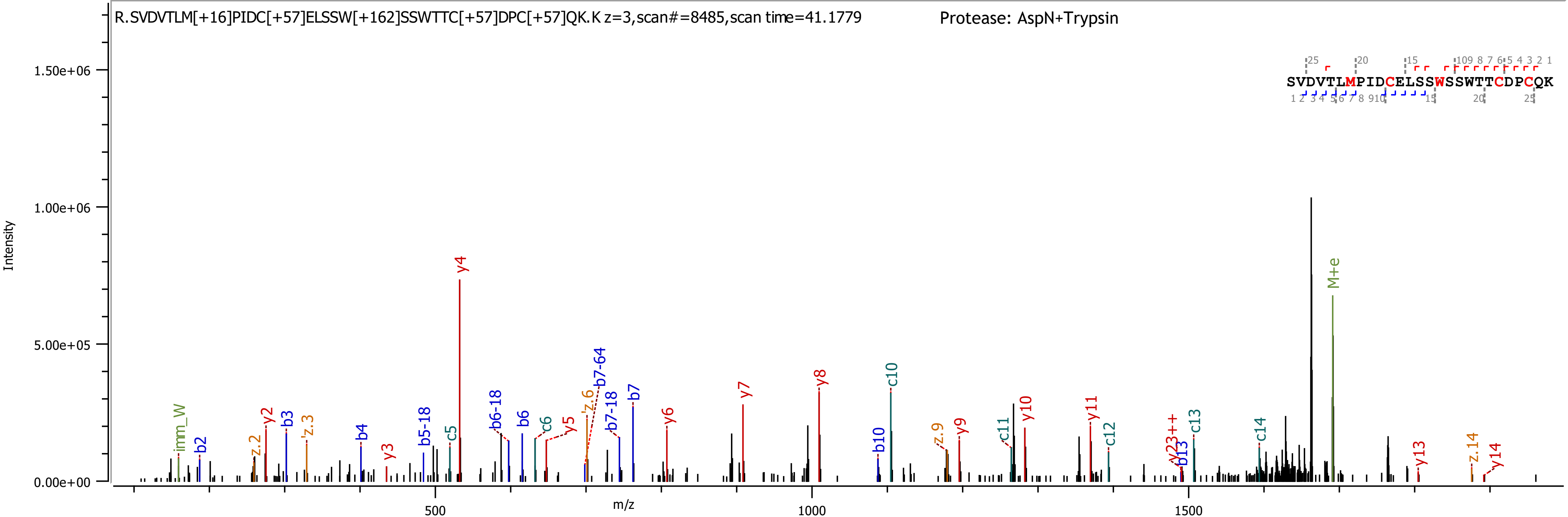

R.SVDVTLM[+16]PIDC[+57]ELSSW[+162]SSWTTC[+57]DPC[+57]QK.K z=3,scan#=8485,scan time=41.1779
Protease: AspN+Trypsin
SVDVTLMPIDCELSSWSSWTTCDPCQK
Intensity
m/z
1.50e+06
1.00e+06
5.00e+05
0.00e+00
500
1000
1500

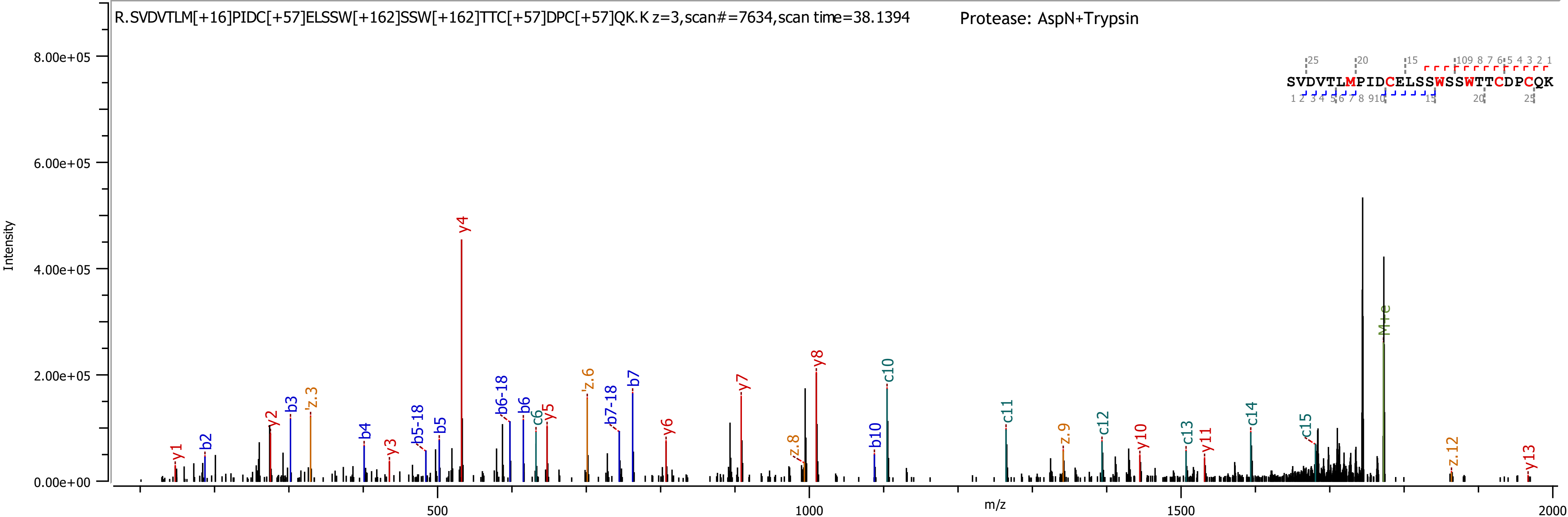

R.SVDVTLM[+16]PIDC[+57]ELSSW[+162]SSW[+162]TTC[+57]DPC[+57]QK.K z=3,scan#=7634,scan time=38.1394
Protease: AspN+Trypsin
SVDVTLMPIDCELSSWSSWTTCDPCQK
Intensity
m/z

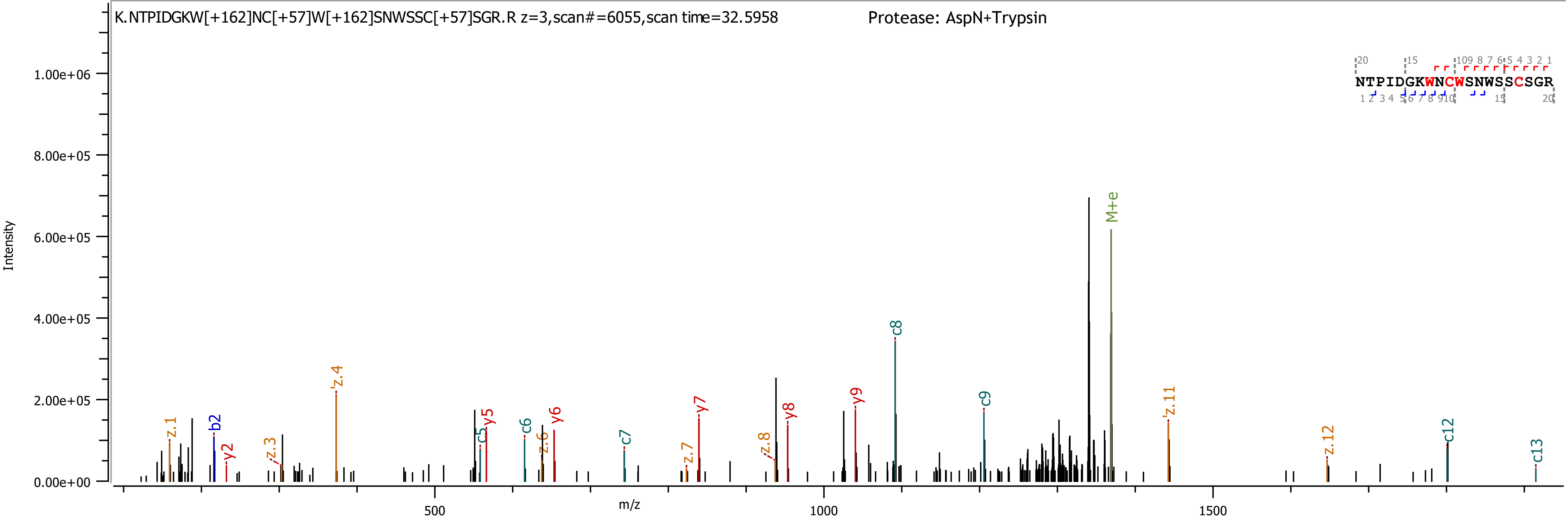

K.NTPIDGKW[+162]NC[+57]W[+162]SNWSSC[+57]SGR.R z=3,scan#=6055,scan time=32.5958
Protease: AspN+Trypsin
NTPIDGKWNCWSNWSSCSGR
Intensity
m/z
1.00e+06
8.00e+05
6.00e+05
4.00e+05
2.00e+05
0.00e+00
500
1000
1500
z.1
b2
y2
z.3
z.4
c5
y5
c6
z.6
y6
c7
z.7
y7
z.8
y8
y9
c8
c9
M+e
z.11
z.12
c12
c13

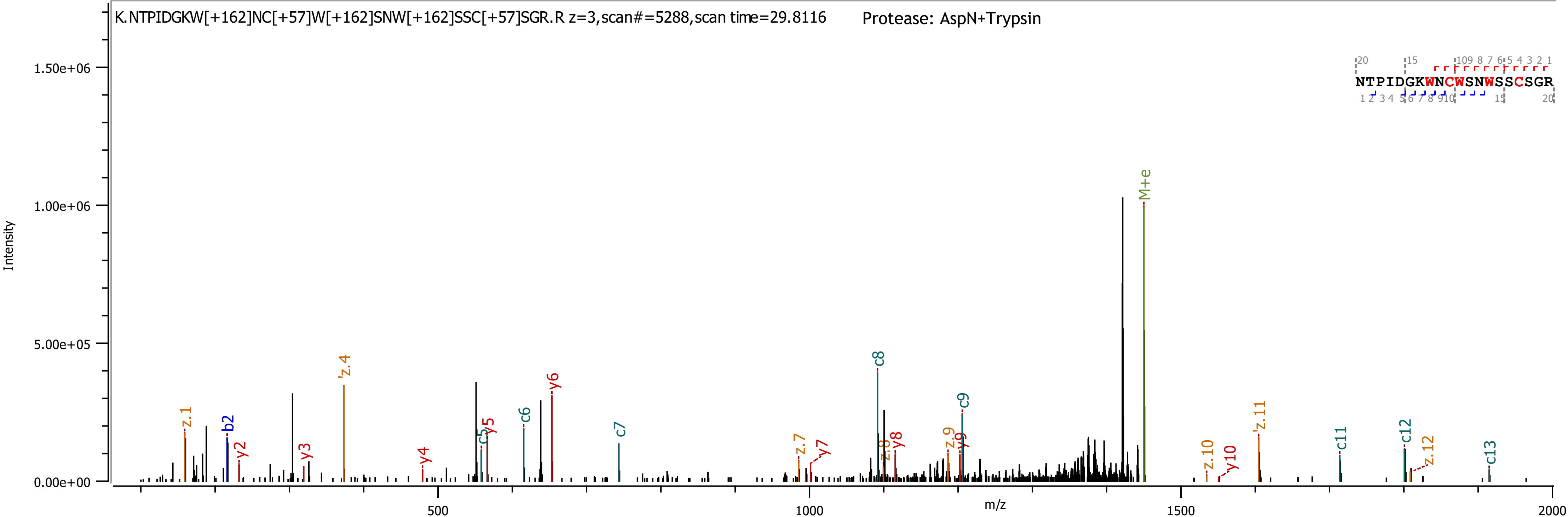

K.NTPIDGKW[+162]NC[+57]W[+162]SNW[+162]SSC[+57]SGR.R z=3,scan#=5288,scan time=29.8116
Protease: AspN+Trypsin
NTPIDGKWNCWSNWSSCSGR
Intensity
m/z
1.50e+06
1.00e+06
5.00e+05
0.00e+00
500
1000
1500
2000
M+e

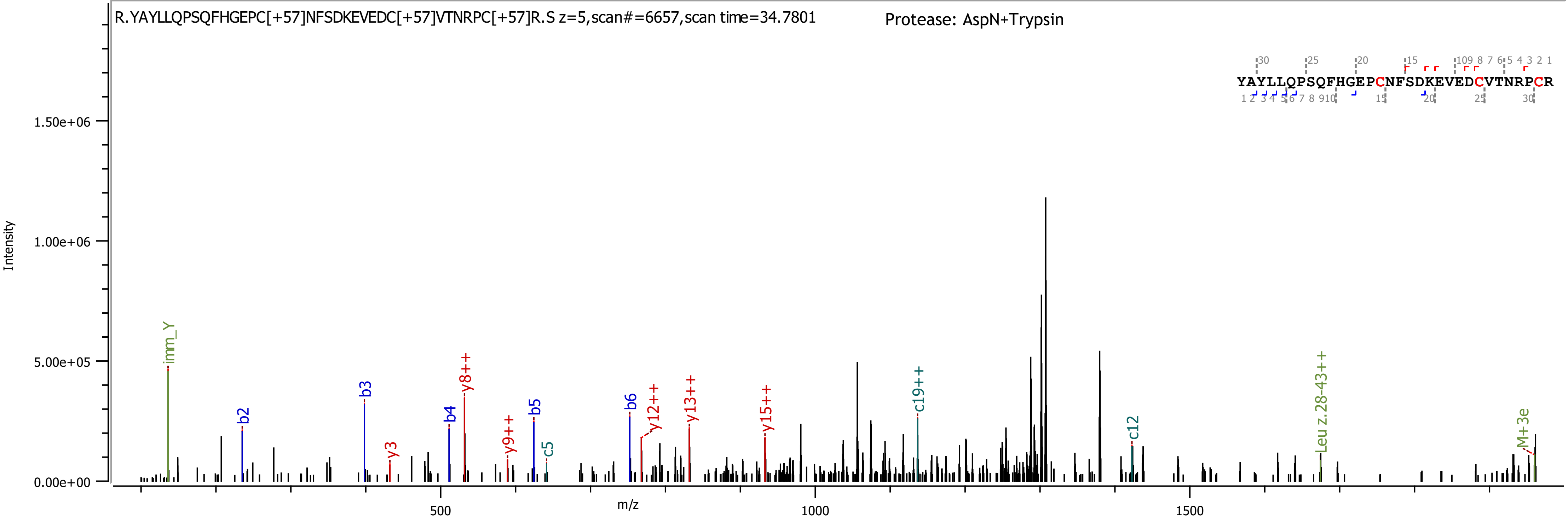
R.YAYLLQPSQFHGEPC[+57]NFSDKEVEDC[+57]VTNRPC[+57]R.S z=5,scan#=6657,scan time=34.7801
Protease: AspN+Trypsin
YAYLLQPSQFHGEPCNFSDKEVEDCVTNRPCR
Intensity
m/z
1.50e+06
1.00e+06
5.00e+05
0.00e+00
500
1000
1500
imm_Y
b2
b3
y3
b4
y8++
y9++
b5
c5
b6
y12++
y13++
y15++
c19++
c12
Leu z.28-43++
M+3e

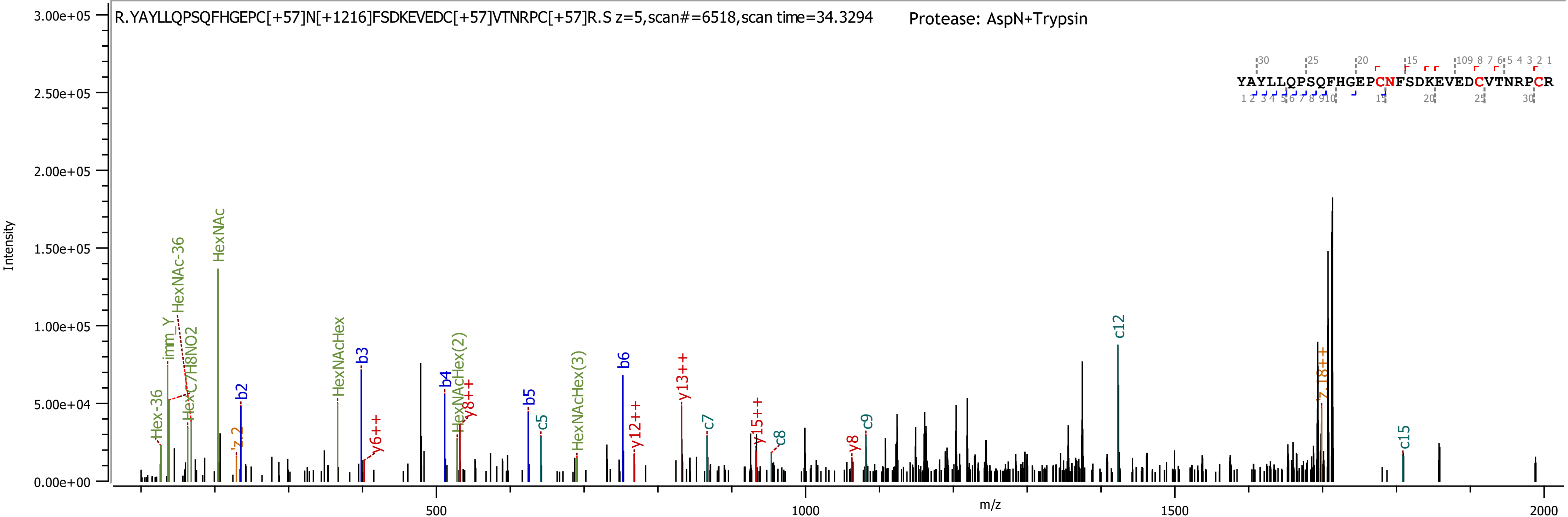
R.YAYLLQPSQFHGEPC[+57]N[+1216]FSDKEVEDC[+57]VTNRPC[+57]R.S z=5,scan#=6518,scan time=34.3294
Protease: AspN+Trypsin
YAYLLQPSQFHGEPCNFSDKEVEDCVTNRPCR
Intensity
m/z
3.00e+05
2.50e+05
2.00e+05
1.50e+05
1.00e+05
5.00e+04
0.00e+00
500
1000
1500
2000
Hex-36
imm_Y
HexNAc-36
HexC7H8NO2
HexNAc
z.2
b2
HexNAcHex
b3
y6++
b4
HexNAcHex(2)
y8++
b5
c5
HexNAcHex(3)
b6
y12++
y13++
c7
y15++
c8
y8
c9
c12
z.18++
c15

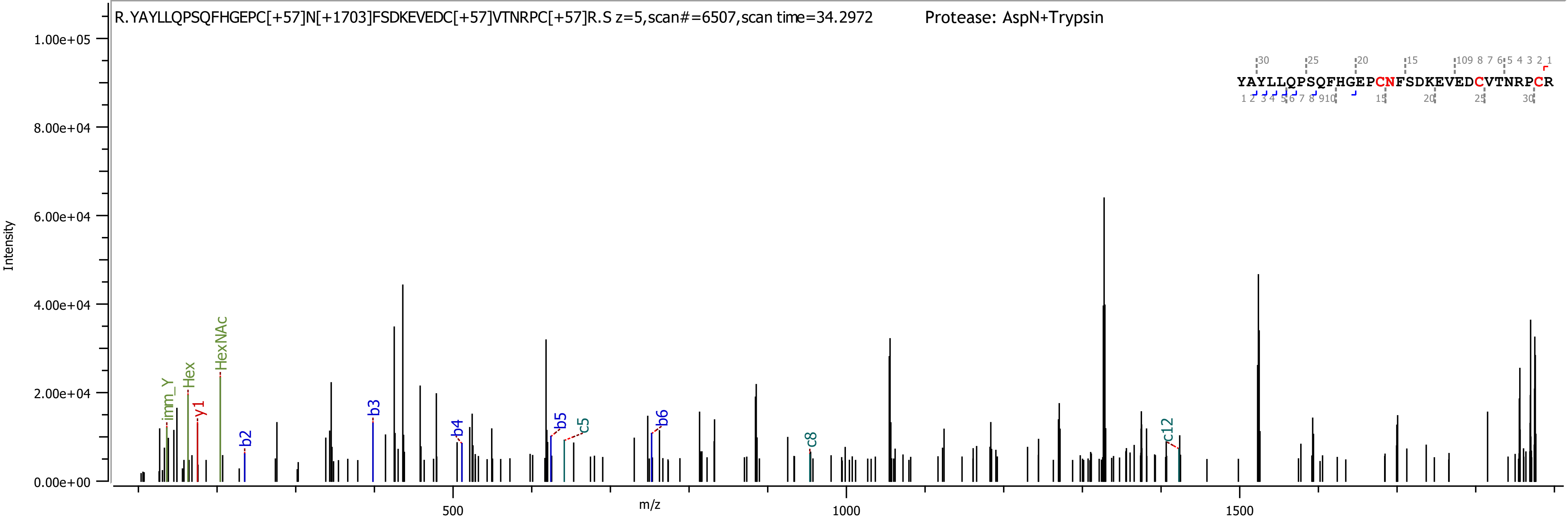
R.YAYLLQPSQFHGEPC[+57]N[+1703]FSDKEVEDC[+57]VTNRPC[+57]R.S z=5,scan#=6507,scan time=34.2972
Protease: AspN+Trypsin
YAYLLQPSQFHGEPCNFSDKEVEDCVTNRPCR
Intensity
m/z
0.00e+00
2.00e+04
4.00e+04
6.00e+04
8.00e+04
1.00e+05
500
1000
1500
imm_Y
Hex
y1
HexNAc
b2
b3
b4
b5
c5
b6
c8
c12

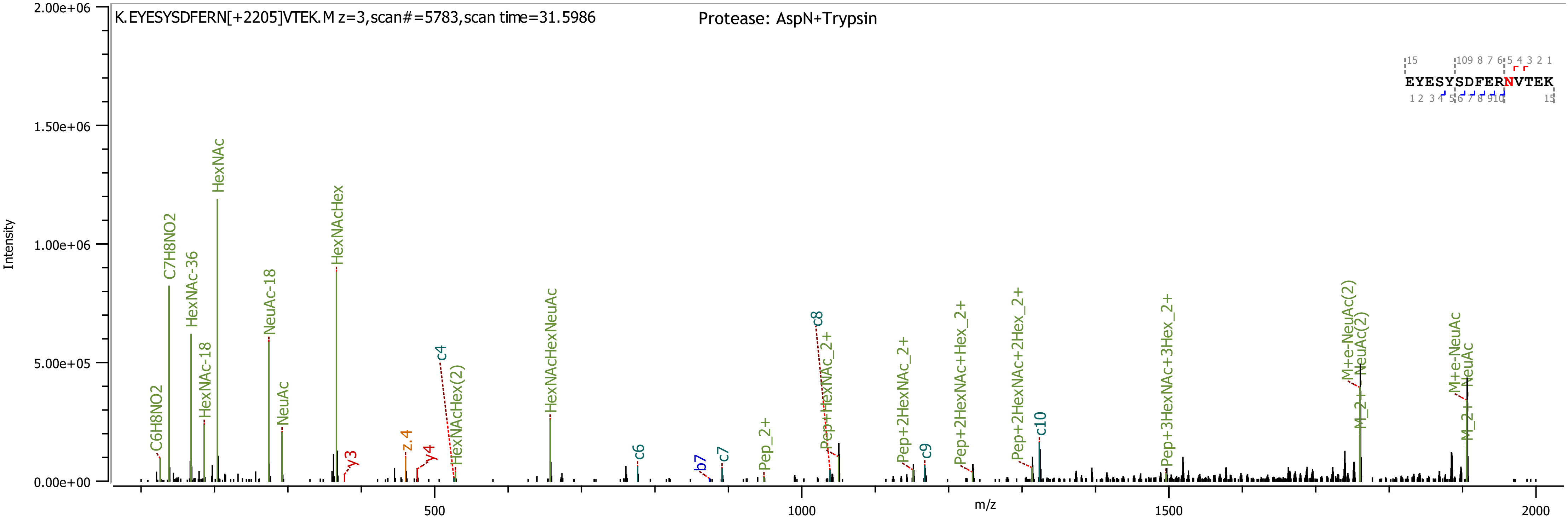

K.EYESYSDFERN[+2205]VTEK.M z=3,scan#=5783,scan time=31.5986
Protease: AspN+Trypsin
EYESYSDFERNVTEK
Intensity
m/z
2.00e+06
1.50e+06
1.00e+06
5.00e+05
0.00e+00
500
1000
1500
2000
C6H8NO2
C7H8NO2
HexNAc-36
HexNAc-18
HexNAc
NeuAc-18
NeuAc
HexNAcHex
y3
z.4
y4
c4
HexNAcHex(2)
HexNAcHexNeuAc
c6
b7
c7
Pep_2+
c8
Pep+HexNAc_2+
Pep+2HexNAc_2+
c9
Pep+2HexNAc+Hex_2+
Pep+2HexNAc+2Hex_2+
c10
Pep+3HexNAc+3Hex_2+
M+e-NeuAc(2)
NeuAc(2)
M_2+
M+e-NeuAc
NeuAc
M_2+

HCD spectra

Glycosylation Site

TSR1_WSSW	SVDVTLMPIDCELSSWSSWTTCDPCQK<br>SVDVTLMPIDCELSSWSSWTTCDPCQK<br>SVDVTLMPIDCELSSWSSWTTCDPCQK<br>SVDVTLMPIDCELSSWSSWTTCDPCQK
TSR2_WNCWSNW	NTPIDGKWNCWSNWSSCSGR<br>NTPIDGKWNCWSNWSSCSGR<br>NTPIDGKWNCWSNWSSCSGR<br>NTPIDGKWNCWSNWSSCSGR
N101	YAYLLQPSQFHGEPCNFSDKEVEDCVTNRPCR<br>YAYLLQPSQFHGEPCN[+1216.4]FSDKEVEDCVTNRPCR<br>YAYLLQPSQFHGEPCN[+1702.6]FSDKEVEDCVTNRPCR
N243	EYESYSDFERN[+2204.8]VTEK

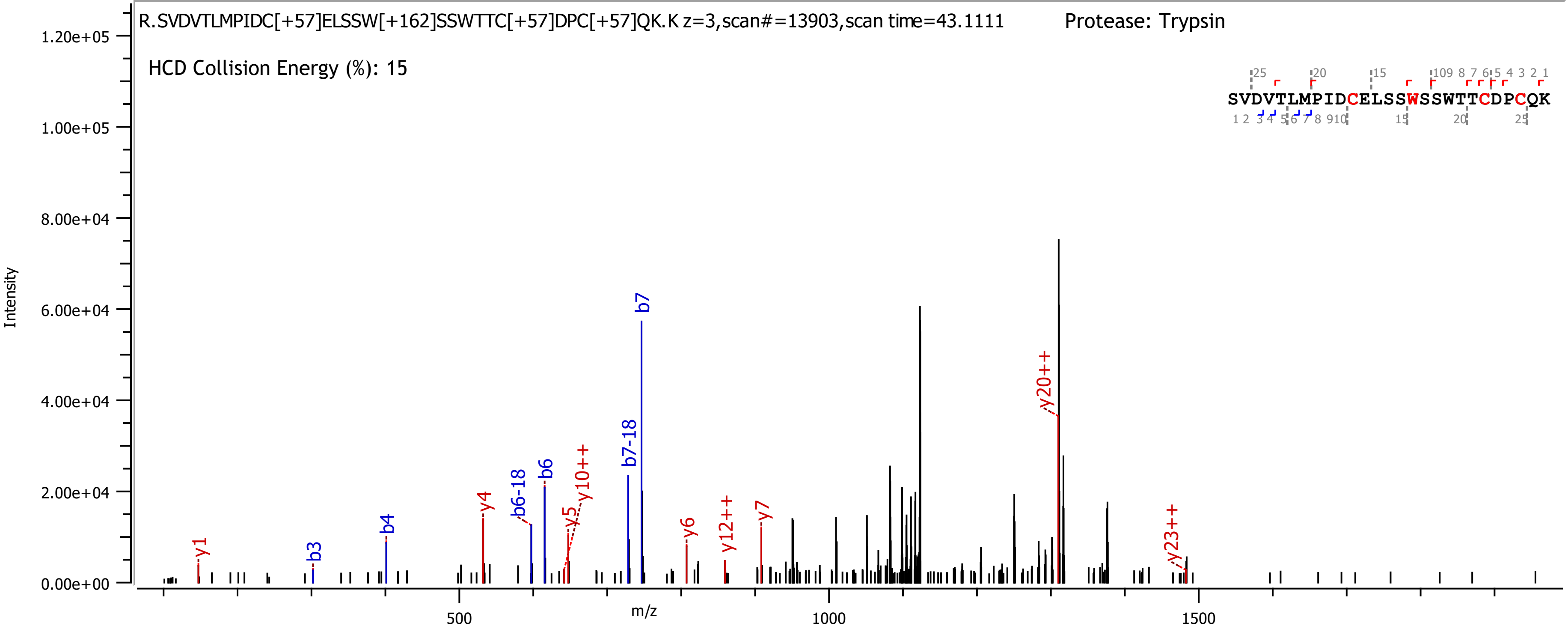
R.SVDVTLMPIDC[+57]ELSSW[+162]SSWTTC[+57]DPC[+57]QK.K z=3,scan#=13903,scan time=43.1111
Protease: Trypsin
HCD Collision Energy (%): 15
SVDVTLMPIDCELSSWSSWTTCDPCQK
Intensity
m/z
1.20e+05
1.00e+05
8.00e+04
6.00e+04
4.00e+04
2.00e+04
0.00e+00
500
1000
1500
y1
b3
b4
y4
b6-18
b6
y5
y10++
b7-18
b7
y6
y12++
y7
y20++
y23++

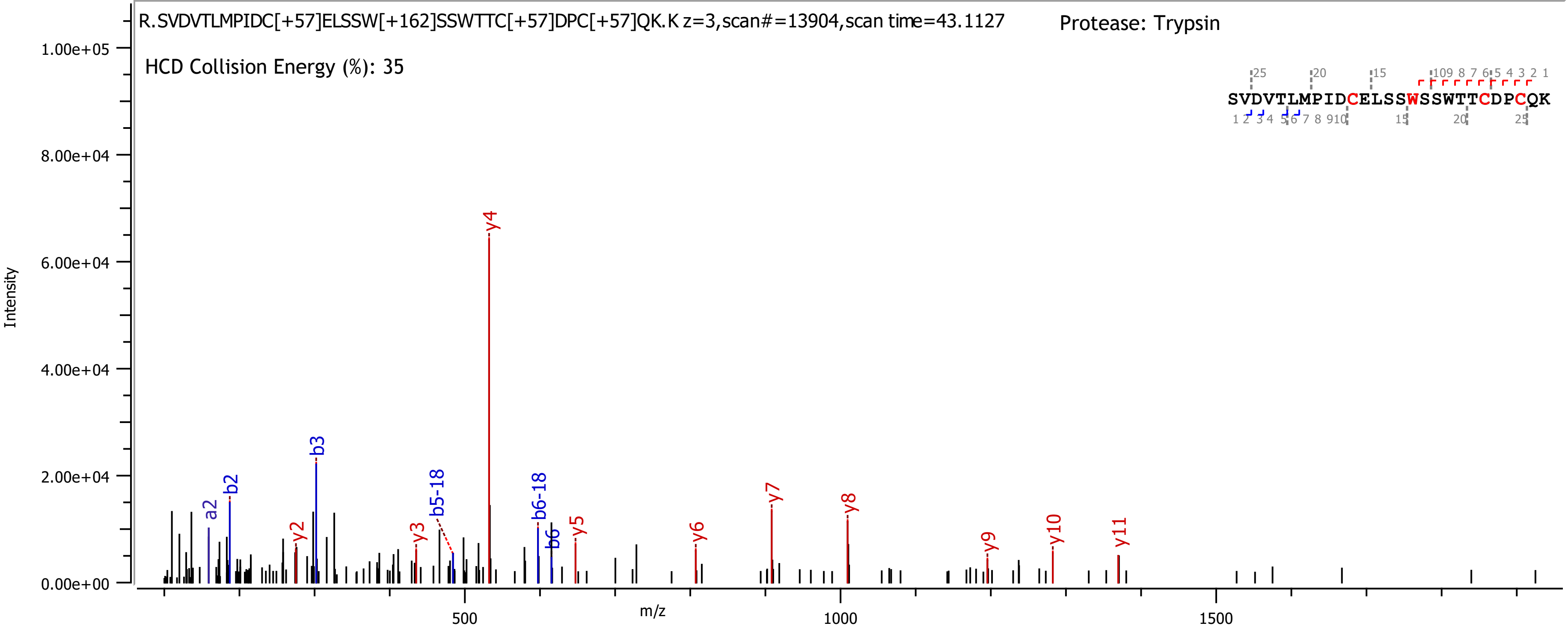

R.SVDVTLMPIDC[+57]ELSSW[+162]SSWTTC[+57]DPC[+57]QK.K z=3,scan#=13904,scan time=43.1127
Protease: Trypsin
HCD Collision Energy (%): 35
SVDVTLMPIDCELSSWSSWTTCDPCQK
Intensity
m/z
1.00e+05
8.00e+04
6.00e+04
4.00e+04
2.00e+04
0.00e+00
500
1000
1500
a2
b2
b3
b5-18
b6-18
b6
y2
y3
y4
y5
y6
y7
y8
y9
y10
y11

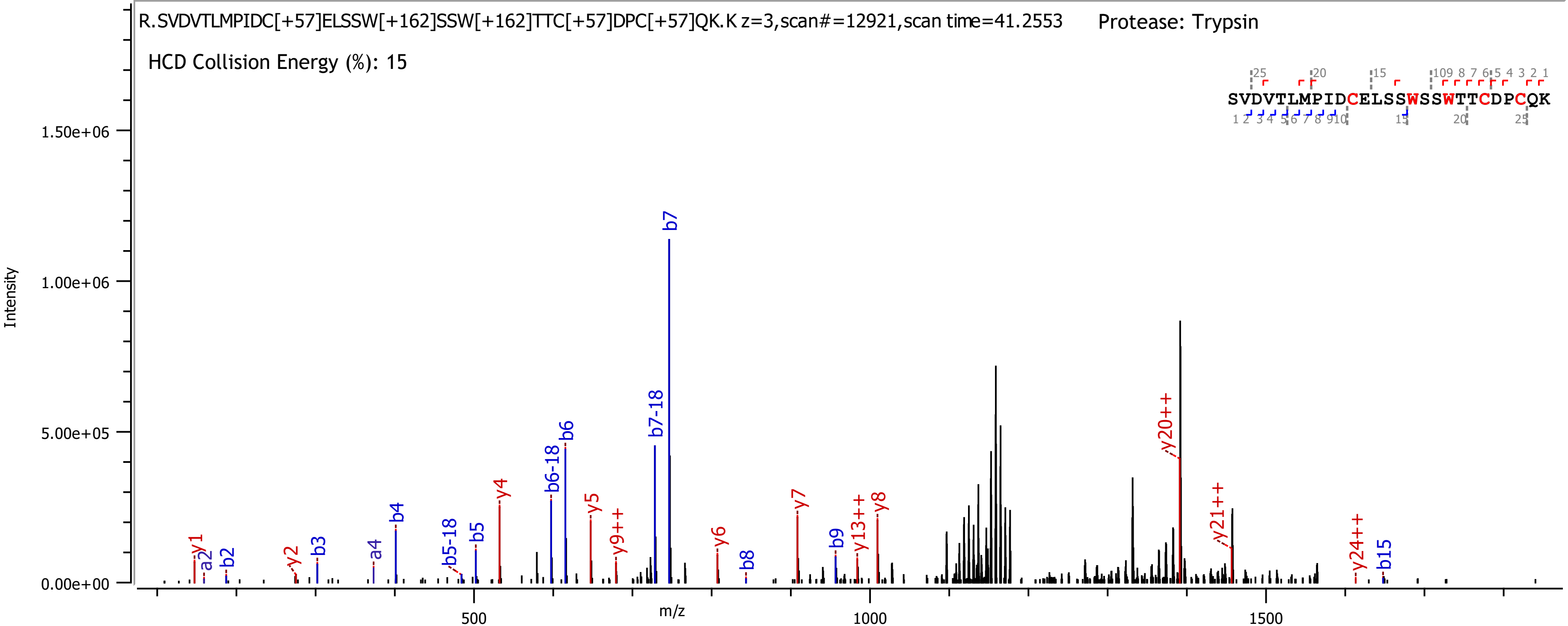
R.SVDVTLMPIDC[+57]ELSSW[+162]SSW[+162]TTC[+57]DPC[+57]QK.K z=3,scan#=12921,scan time=41.2553
Protease: Trypsin
HCD Collision Energy (%): 15
SVDVTLMPIDCELSSWSSWTTCDPCQK
Intensity
m/z
0.00e+00
5.00e+05
1.00e+06
1.50e+06
500
1000
1500
y1
a2
b2
y2
b3
a4
b4
b5-18
b5
y4
b6-18
b6
y5
y9++
b7-18
b7
y6
b8
y7
b9
y13++
y8
y20++
y21++
y24++
b15

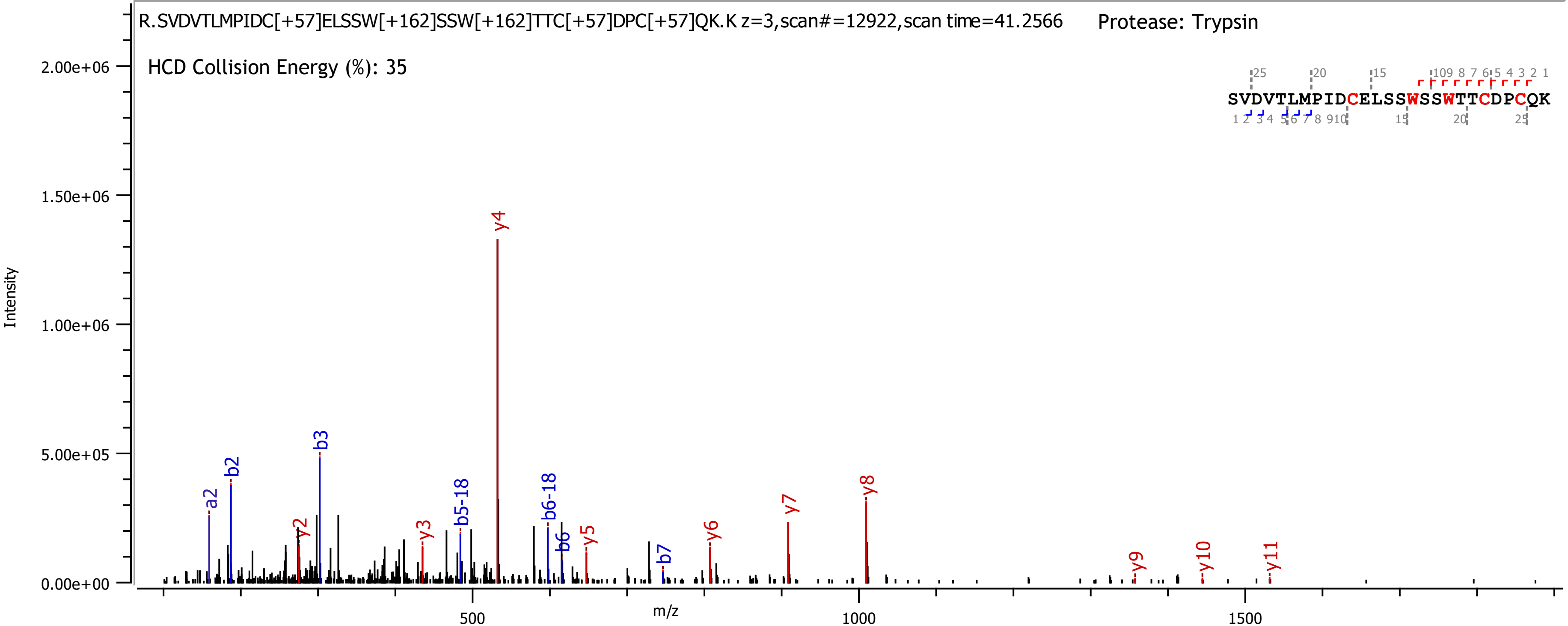
R.SVDVTLMPIDC[+57]ELSSW[+162]SSW[+162]TTC[+57]DPC[+57]QK.K z=3,scan#=12922,scan time=41.2566
Protease: Trypsin
HCD Collision Energy (%): 35
SVDVTLMPIDCELSSWSSWTTCDPCQK
Intensity
m/z
2.00e+06
1.50e+06
1.00e+06
5.00e+05
0.00e+00
500
1000
1500
a2
b2
b3
b5-18
b6-18
b6
b7
y2
y3
y4
y5
y6
y7
y8
y9
y10
y11

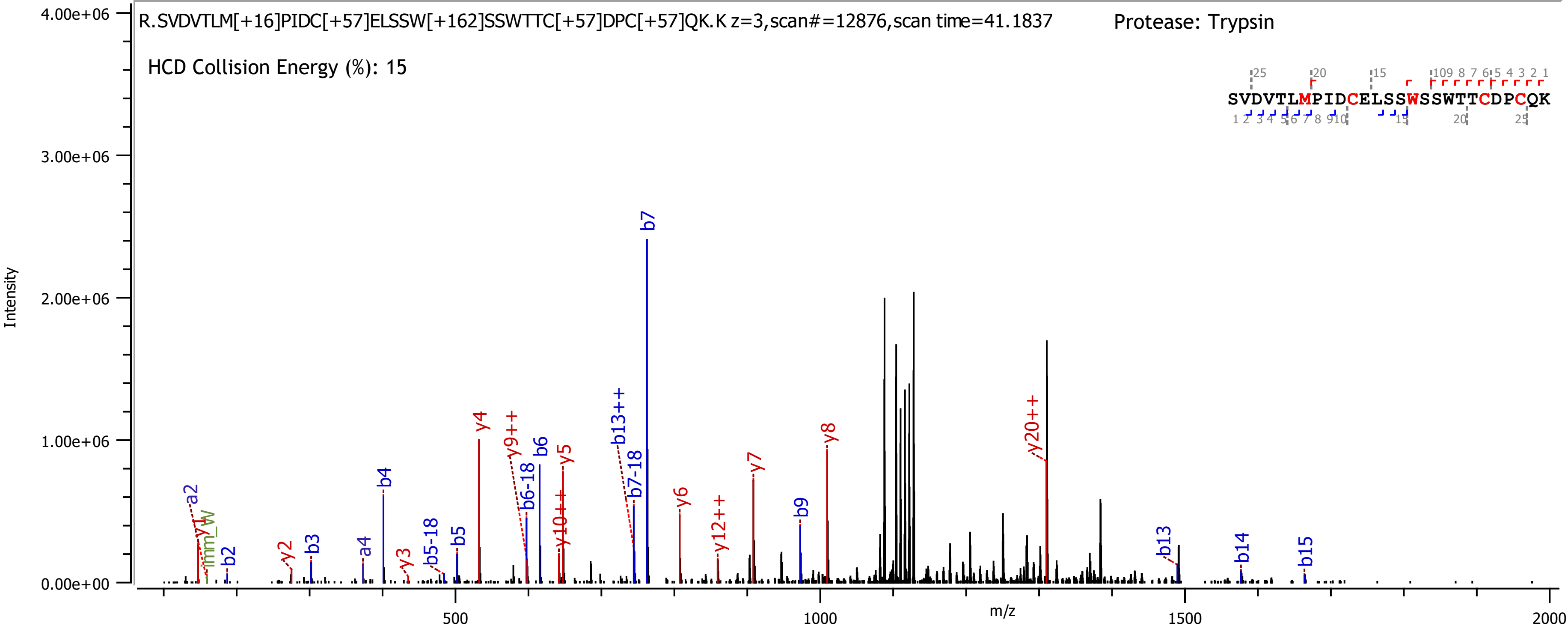

R.SVDVTLM[+16]PIDC[+57]ELSSW[+162]SSWTTC[+57]DPC[+57]QK.K z=3,scan#=12876,scan time=41.1837
Protease: Trypsin
HCD Collision Energy (%): 15
SVDVTLMPIDCELSSWSSWTTCDPCQK
Intensity
m/z
4.00e+06
3.00e+06
2.00e+06
1.00e+06
0.00e+00
500
1000
1500
2000
a2
y1
Imm_W
b2
y2
b3
a4
b4
y3
b5-18
b5
y4
y9++
b6-18
b6
y10++
y5
b13++
b7-18
b7
y6
y12++
y7
b9
y8
y20++
b13
b14
b15

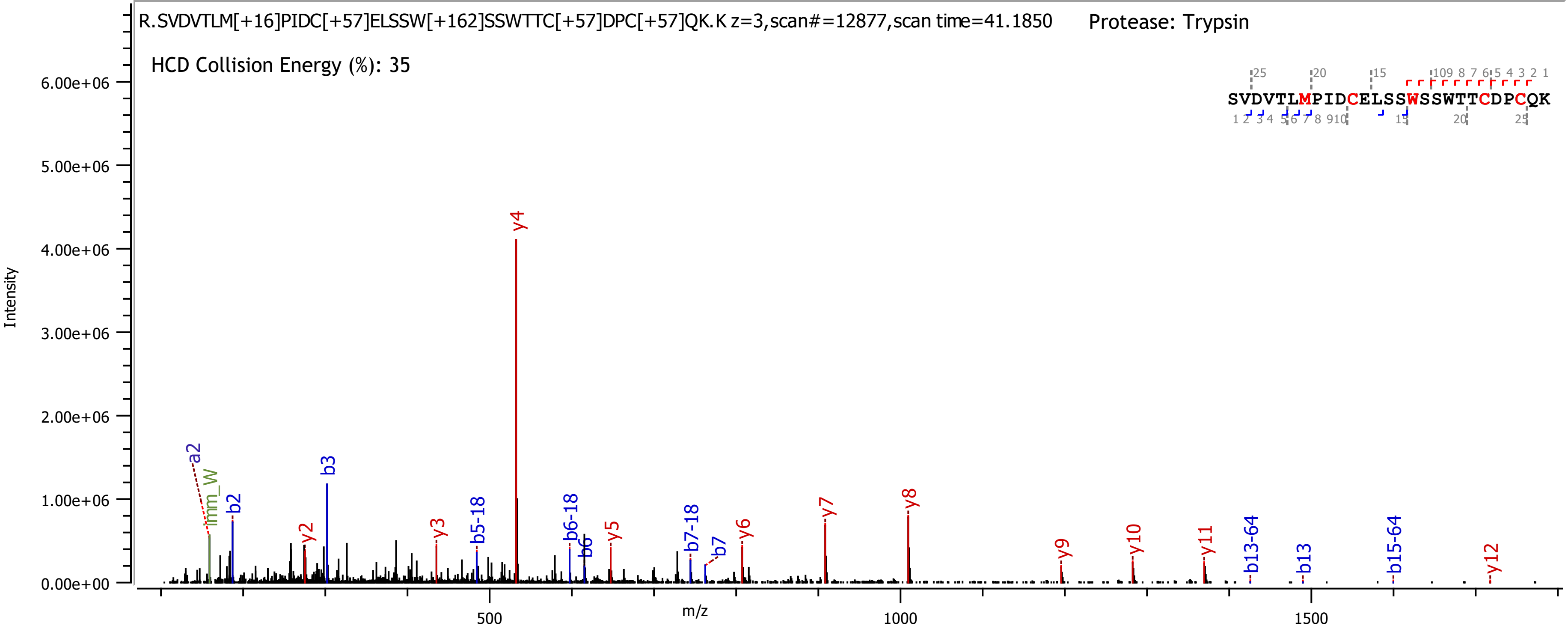
R.SVDVTLM[+16]PIDC[+57]ELSSW[+162]SSWTTC[+57]DPC[+57]QK.K z=3,scan#=12877,scan time=41.1850
Protease: Trypsin
HCD Collision Energy (%): 35
SVDVTLMPIDCELSSWSSWTTCDPCQK
Intensity
m/z
a2
imm_W
b2
y2
b3
y3
b5-18
y4
b6-18
b6
y5
b7-18
b7
y6
y7
y8
y9
y10
y11
b13-64
b13
b15-64
y12

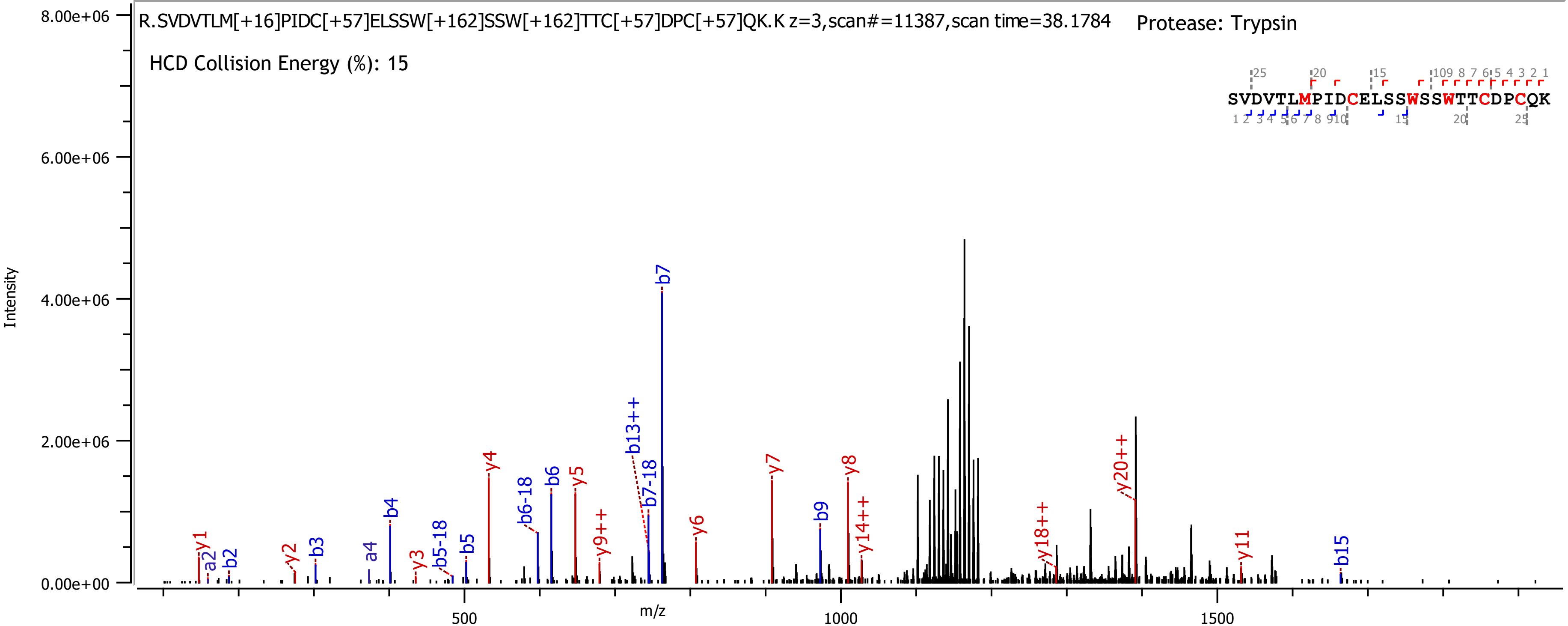
R.SVDVTLM[+16]PIDC[+57]ELSSW[+162]SSW[+162]TTC[+57]DPC[+57]QK.K z=3,scan#=11387,scan time=38.1784
Protease: Trypsin
HCD Collision Energy (%): 15
SVDVTLMPIDCELSSWSSWTTCDPCQK
Intensity
m/z
8.00e+06
6.00e+06
4.00e+06
2.00e+06
0.00e+00
500
1000
1500
y1
a2
b2
y2
b3
a4
b4
y3
b5-18
b5
y4
b6-18
b6
y5
y9++
b13++
b7-18
b7
y6
y7
b9
y8
y14++
y18++
y20++
y11
b15

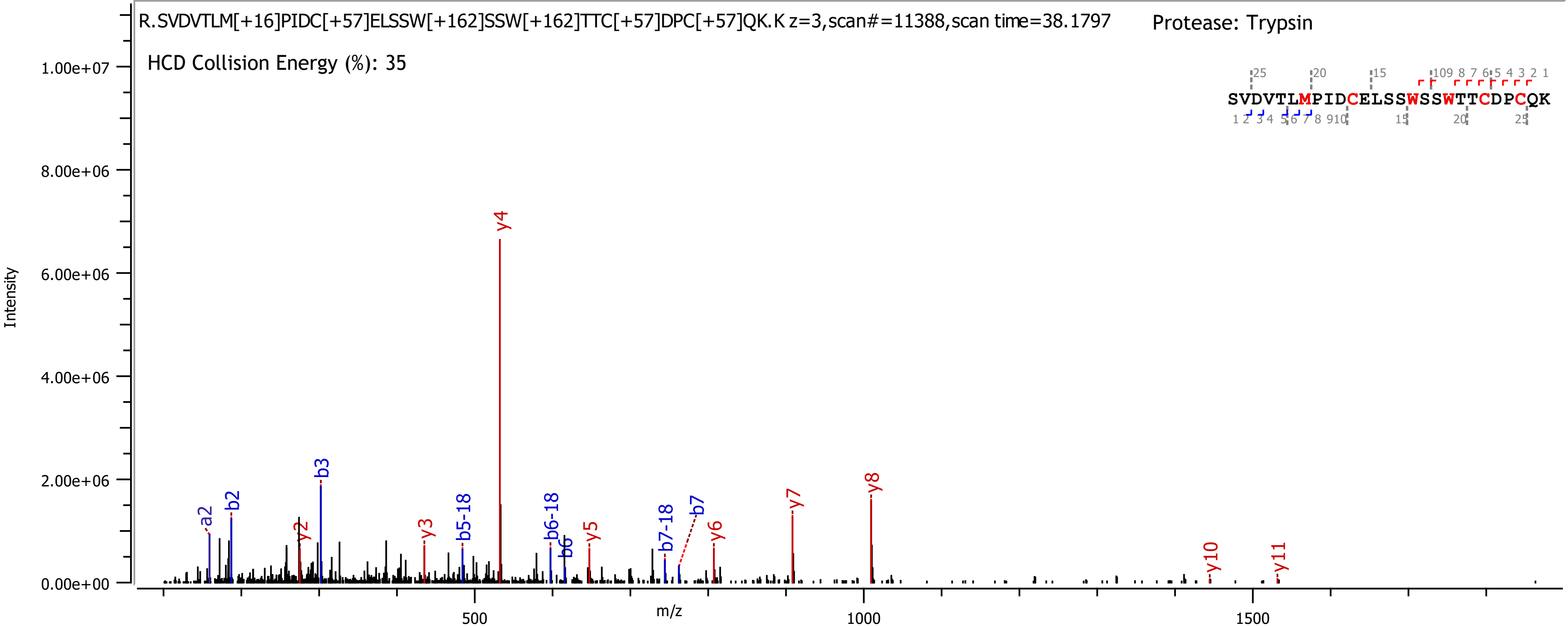

R.SVDVTLM[+16]PIDC[+57]ELSSW[+162]SSW[+162]TTC[+57]DPC[+57]QK.K z=3,scan#=11388,scan time=38.1797
Protease: Trypsin
HCD Collision Energy (%): 35
SVDVTLMPIDCELSSWSSWTTCDPCQK
Intensity
1.00e+07
8.00e+06
6.00e+06
4.00e+06
2.00e+06
0.00e+00
m/z
500
1000
1500
a2
b2
y2
b3
y3
b5-18
y4
b6-18
b6
y5
b7-18
b7
y6
y7
y8
y10
y11

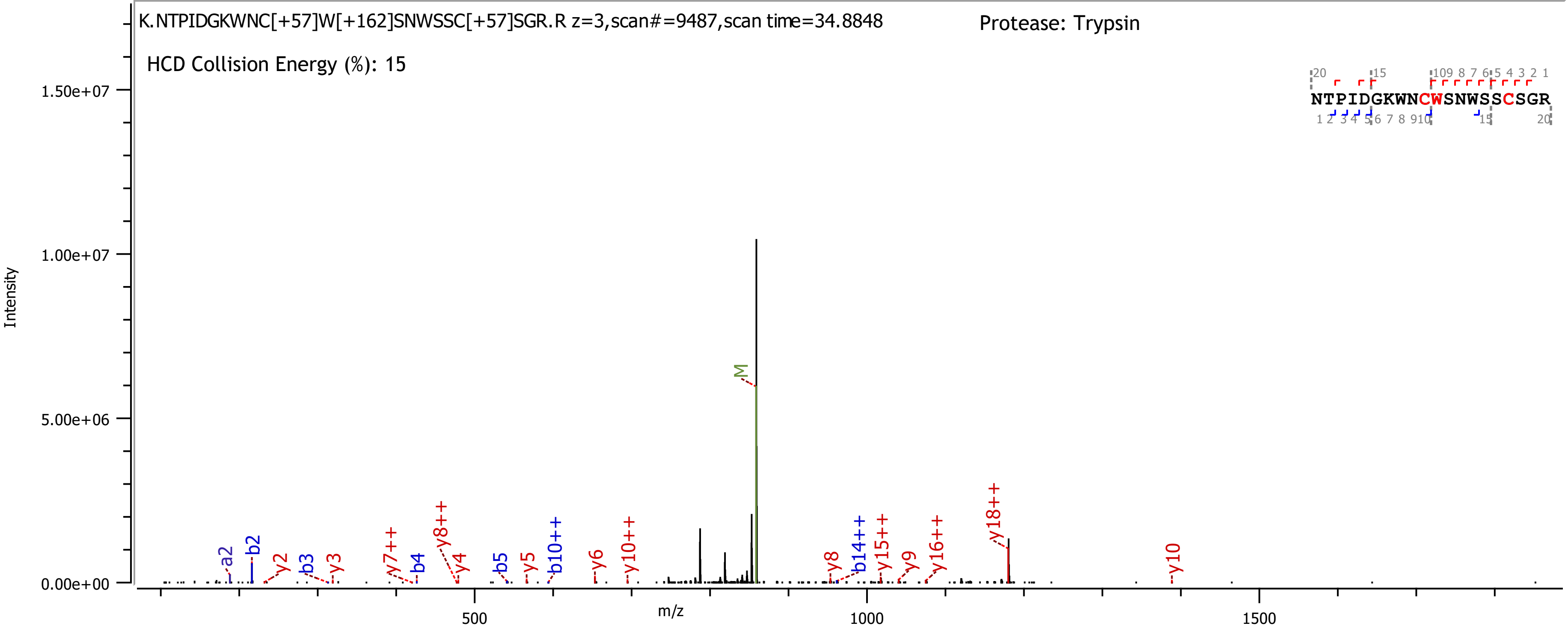
K.NTPIDGKWNC[+57]W[+162]SNWSSC[+57]SGR.R z=3,scan#=9487,scan time=34.8848
Protease: Trypsin
HCD Collision Energy (%): 15
NTPIDGKWNCWSNWSSCSGR
Intensity
m/z
1.50e+07
1.00e+07
5.00e+06
0.00e+00
500
1000
1500
a2
b2
y2
b3
y3
y7++
b4
y8++
y4
b5
y5
b10++
y6
y10++
M
y8
b14++
y15++
y9
y16++
y18++
y10

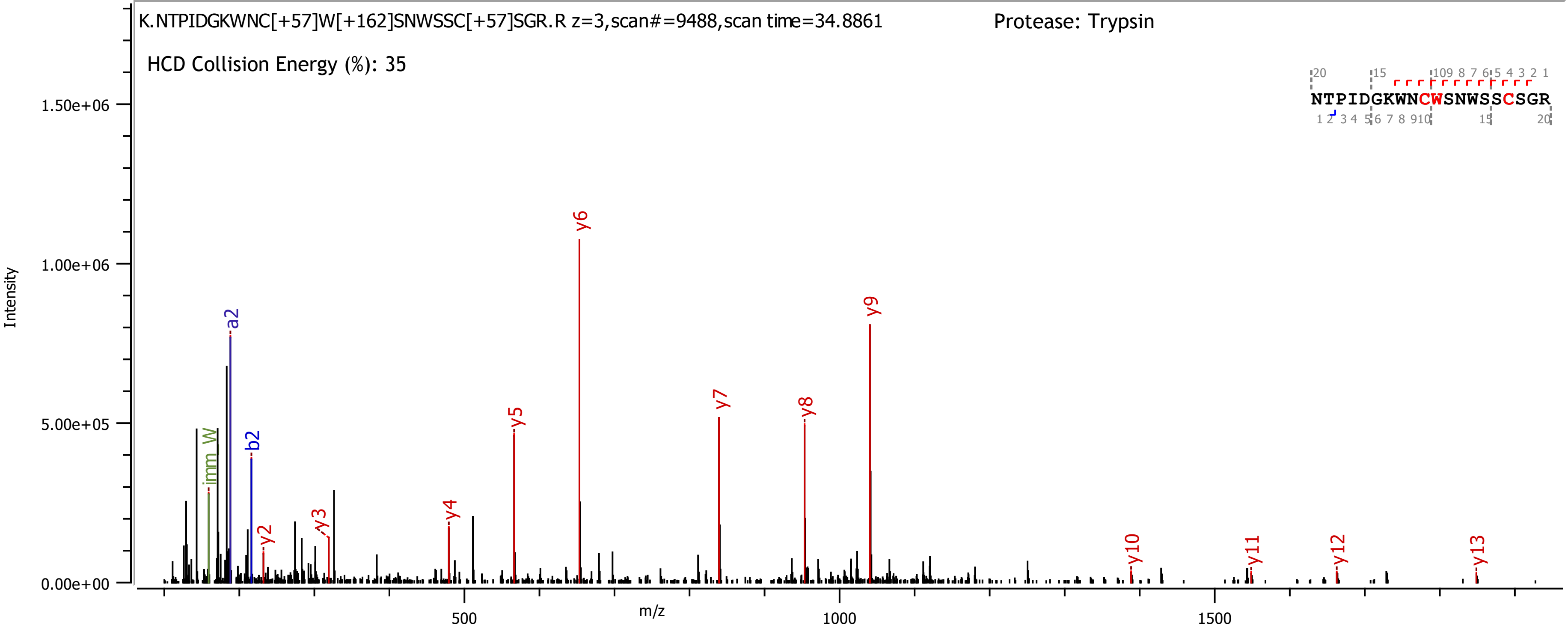

K.NTPIDGKWNC[+57]W[+162]SNWSSC[+57]SGR.R z=3,scan#=9488,scan time=34.8861
Protease: Trypsin
HCD Collision Energy (%): 35
NTPIDGKWNCWSNWSSCSGR
Intensity
1.50e+06
1.00e+06
5.00e+05
0.00e+00
m/z
500
1000
1500
imm W
a2
b2
y2
y3
y4
y5
y6
y7
y8
y9
y10
y11
y12
y13

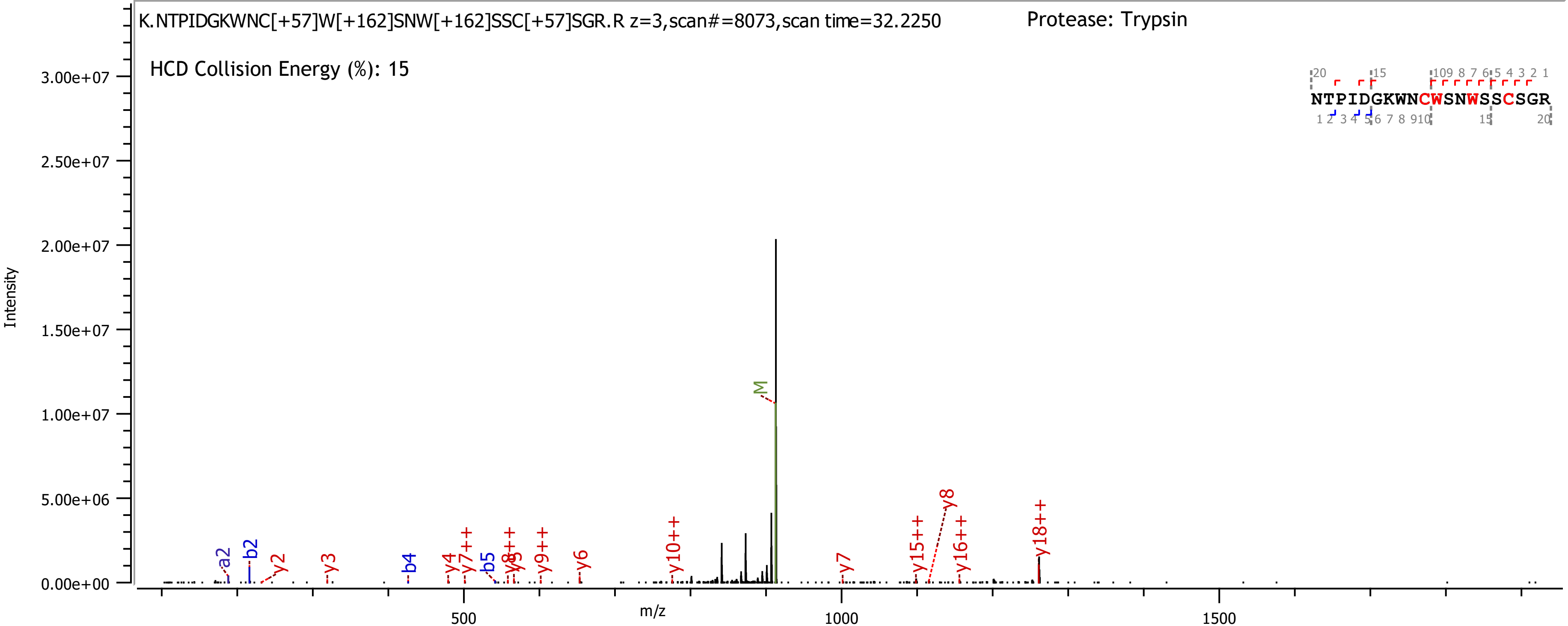

K.NTPIDGKWNC[+57]W[+162]SNW[+162]SSC[+57]SGR.R z=3,scan#=8073,scan time=32.2250
Protease: Trypsin
HCD Collision Energy (%): 15
NTPIDGKWNCWSNWSSCSGR
Intensity
m/z
3.00e+07
2.50e+07
2.00e+07
1.50e+07
1.00e+07
5.00e+06
0.00e+00
500
1000
1500
a2
b2
y2
y3
b4
y4
y7++
b5
y8++
y5
y9++
y6
y10++
M
y7
y15++
y8
y16++
y18++

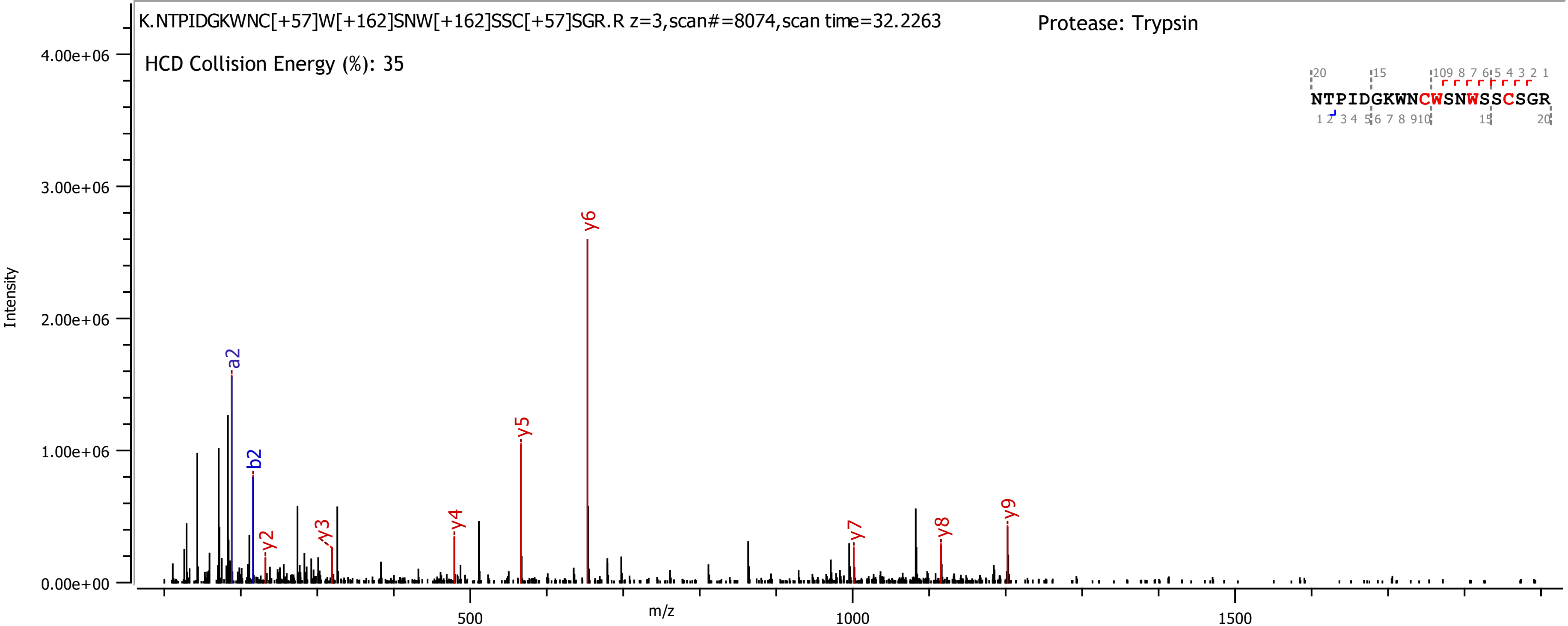
K.NTPIDGKWNC[+57]W[+162]SNW[+162]SSC[+57]SGR.R z=3,scan#=8074,scan time=32.2263
Protease: Trypsin
HCD Collision Energy (%): 35
NTPIDGKWNCWSNWSSCSGR
Intensity
m/z
4.00e+06
3.00e+06
2.00e+06
1.00e+06
0.00e+00
500
1000
1500
a2
b2
y2
y3
y4
y5
y6
y7
y8
y9

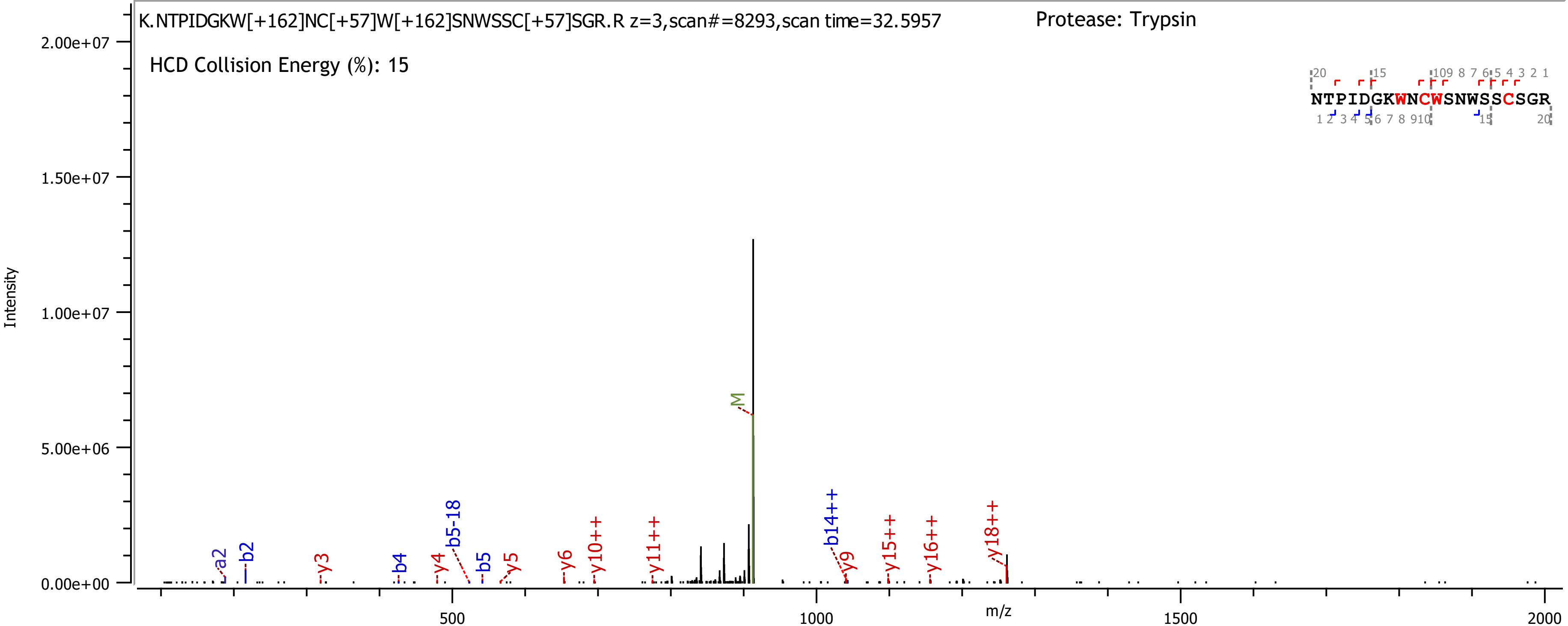
K.NTPIDGKW[+162]NC[+57]W[+162]SNWSSC[+57]SGR.R z=3,scan#=8293,scan time=32.5957
Protease: Trypsin
HCD Collision Energy (%): 15
NTPIDGKWNCWSNWSSCSGR
Intensity
m/z
M
a2
b2
y3
b4
y4
b5-18
b5
y5
y6
y10++
y11++
b14++
y9
y15++
y16++
y18++

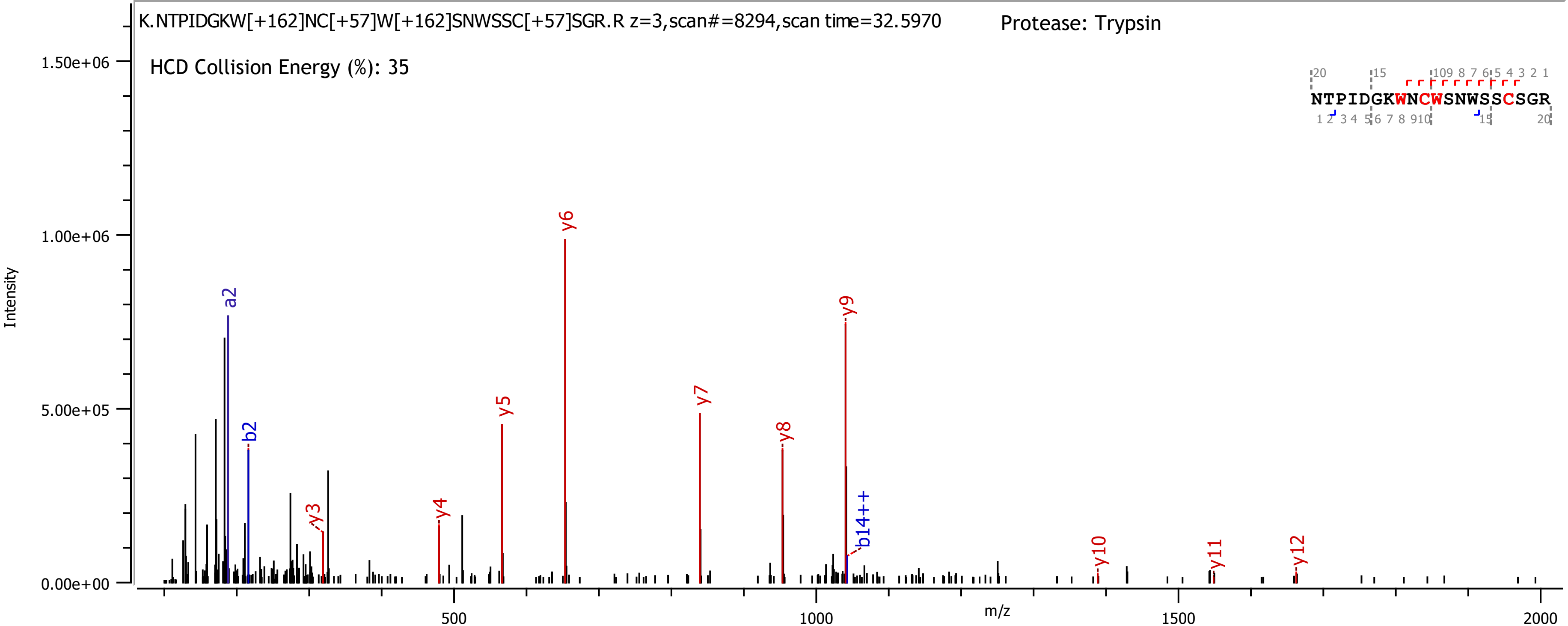

K.NTPIDGKW[+162]NC[+57]W[+162]SNWSSC[+57]SGR.R z=3,scan#=8294,scan time=32.5970
Protease: Trypsin
HCD Collision Energy (%): 35
NTPIDGKWNCWSNWSSCSGR
Intensity
m/z
a2
b2
y3
y4
y5
y6
y7
y8
y9
b14++
y10
y11
y12

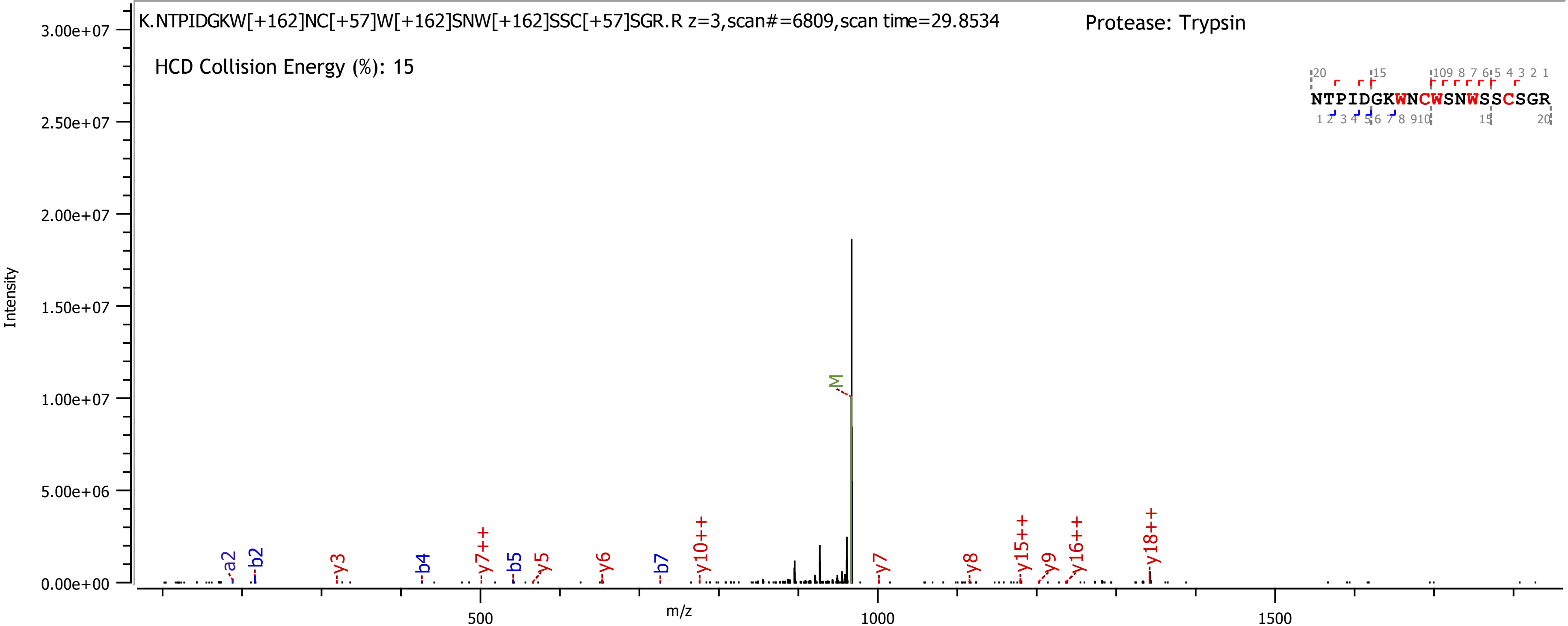
K.NTPIDGKW[+162]NC[+57]W[+162]SNW[+162]SSC[+57]SGR.R z=3,scan#=6809,scan time=29.8534
Protease: Trypsin
HCD Collision Energy (%): 15
NTPIDGKWNCWSNWSSCSGR
Intensity
m/z
3.00e+07
2.50e+07
2.00e+07
1.50e+07
1.00e+07
5.00e+06
0.00e+00
500
1000
1500
M
a2
b2
y3
b4
y7++
b5
y5
y6
b7
y10++
y7
y8
y15++
y9
y16++
y18++

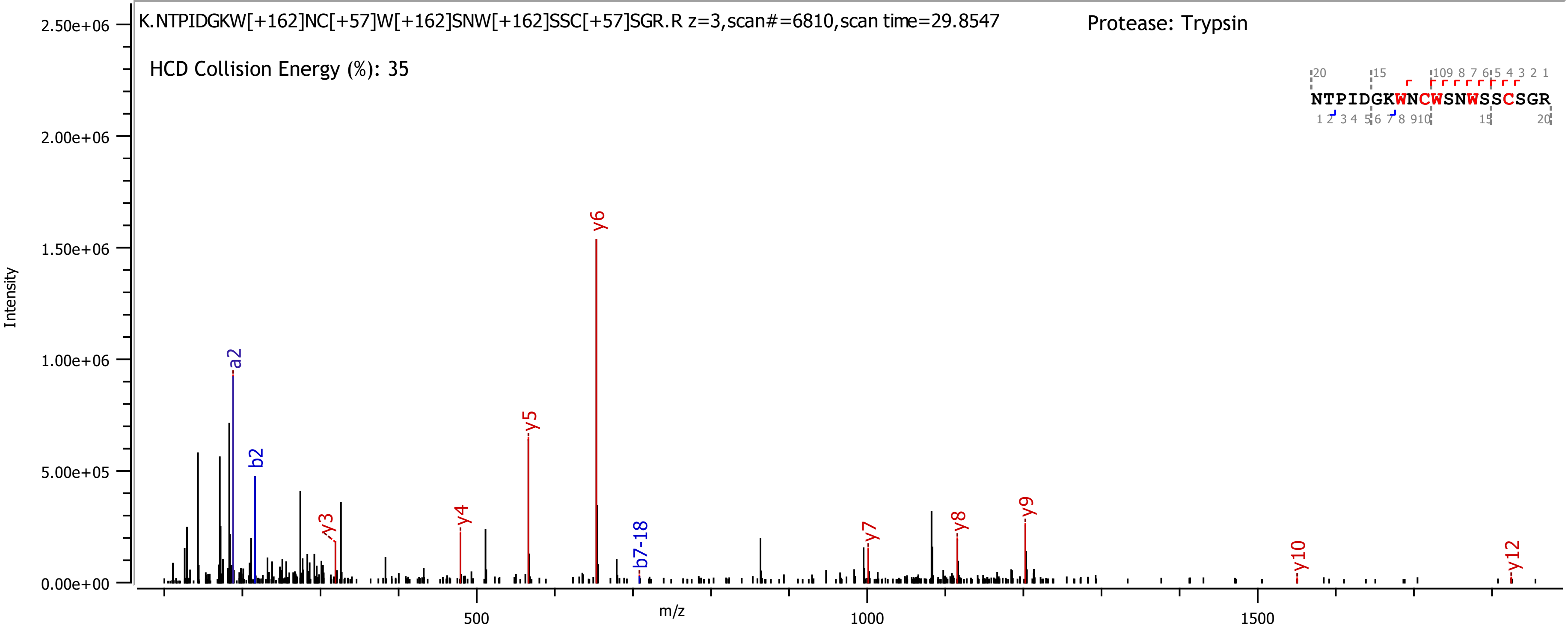

K.NTPIDGKW[+162]NC[+57]W[+162]SNW[+162]SSC[+57]SGR.R z=3,scan#=6810,scan time=29.8547
Protease: Trypsin
HCD Collision Energy (%): 35
NTPIDGKWNCWSNWSSCSGR
Intensity
m/z
2.50e+06
2.00e+06
1.50e+06
1.00e+06
5.00e+05
0.00e+00
500
1000
1500
a2
b2
y3
y4
y5
y6
b7-18
y7
y8
y9
y10
y12

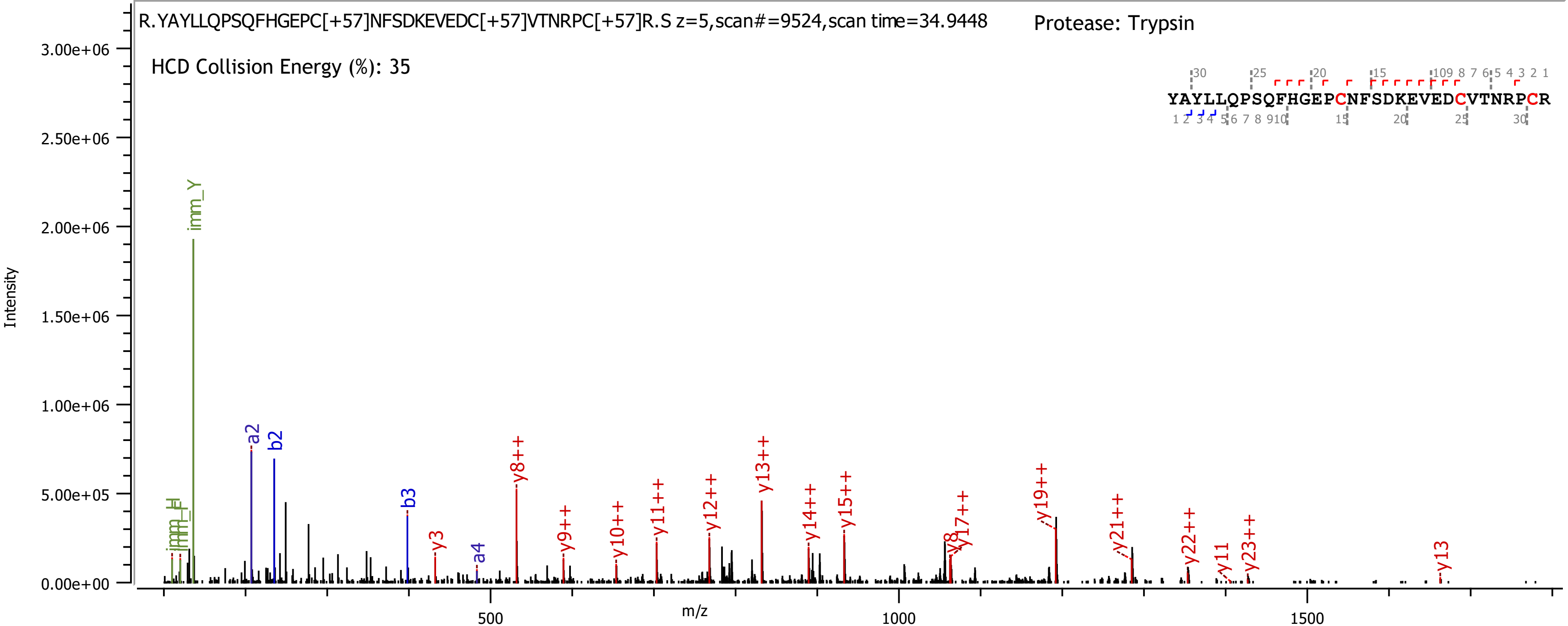
R.YAYLLQPSQFHGEPC[+57]NFSDKEVEDC[+57]VTNRPC[+57]R.S z=5,scan#=9524,scan time=34.9448
Protease: Trypsin
HCD Collision Energy (%): 35
YAYLLQPSQFHGEPCNFSDKEVEDCVTNRPCR
Intensity
m/z
imm_Y
a2
b2
b3
y3
a4
y8++
y9++
y10++
y11++
y12++
y13++
y14++
y15++
y8
y17++
y19++
y21++
y22++
y11
y23++
y13

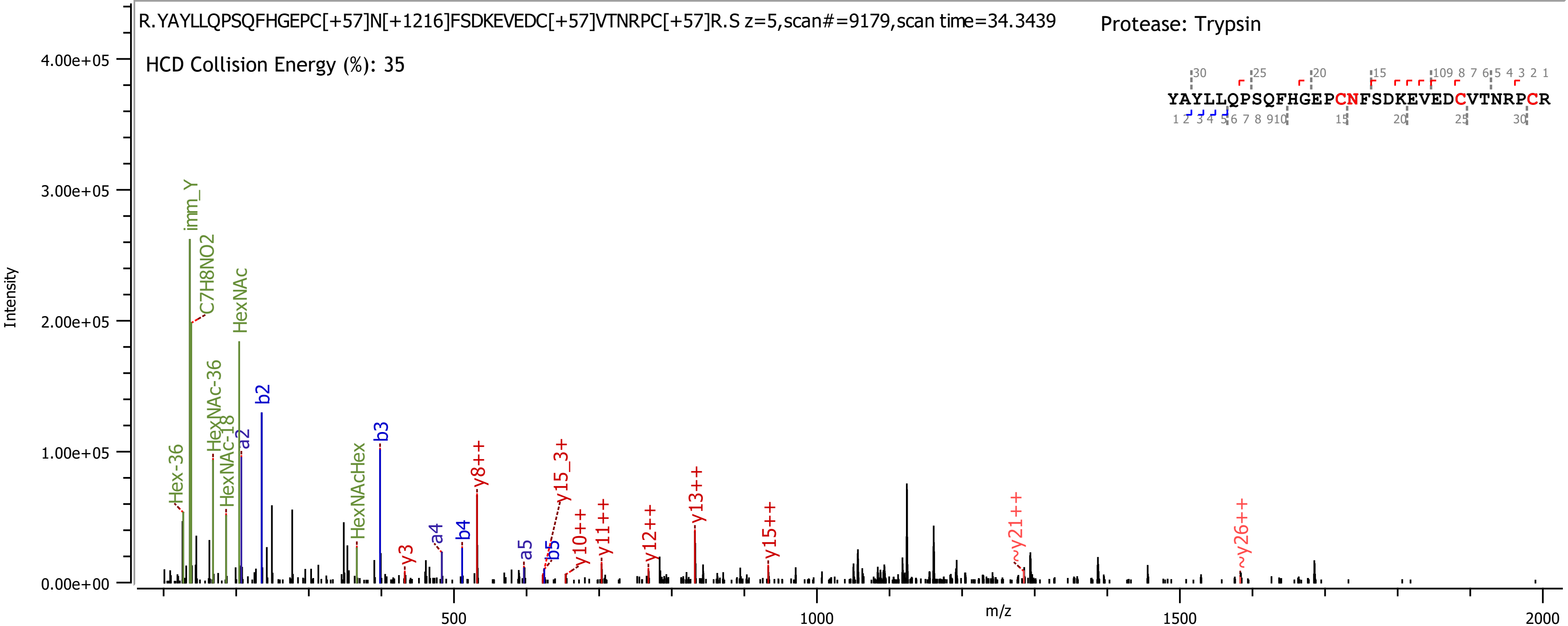
R.YAYLLQPSQFHGEPC[+57]N[+1216]FSDKEVEDC[+57]VTNRPC[+57]R.S z=5,scan#=9179,scan time=34.3439
Protease: Trypsin
HCD Collision Energy (%): 35
YAYLLQPSQFHGEPCNFSDKEVEDCVTNRPCR
Intensity
m/z
4.00e+05
3.00e+05
2.00e+05
1.00e+05
0.00e+00
500
1000
1500
2000
Hex-36
imm_Y
C7H8NO2
HexNAc-36
HexNAc-18
HexNAc
a2
b2
HexNAcHex
b3
y3
a4
b4
y8++
a5
b5
y15_3+
y10++
y11++
y12++
y13++
y15++
~y21++
~y26++

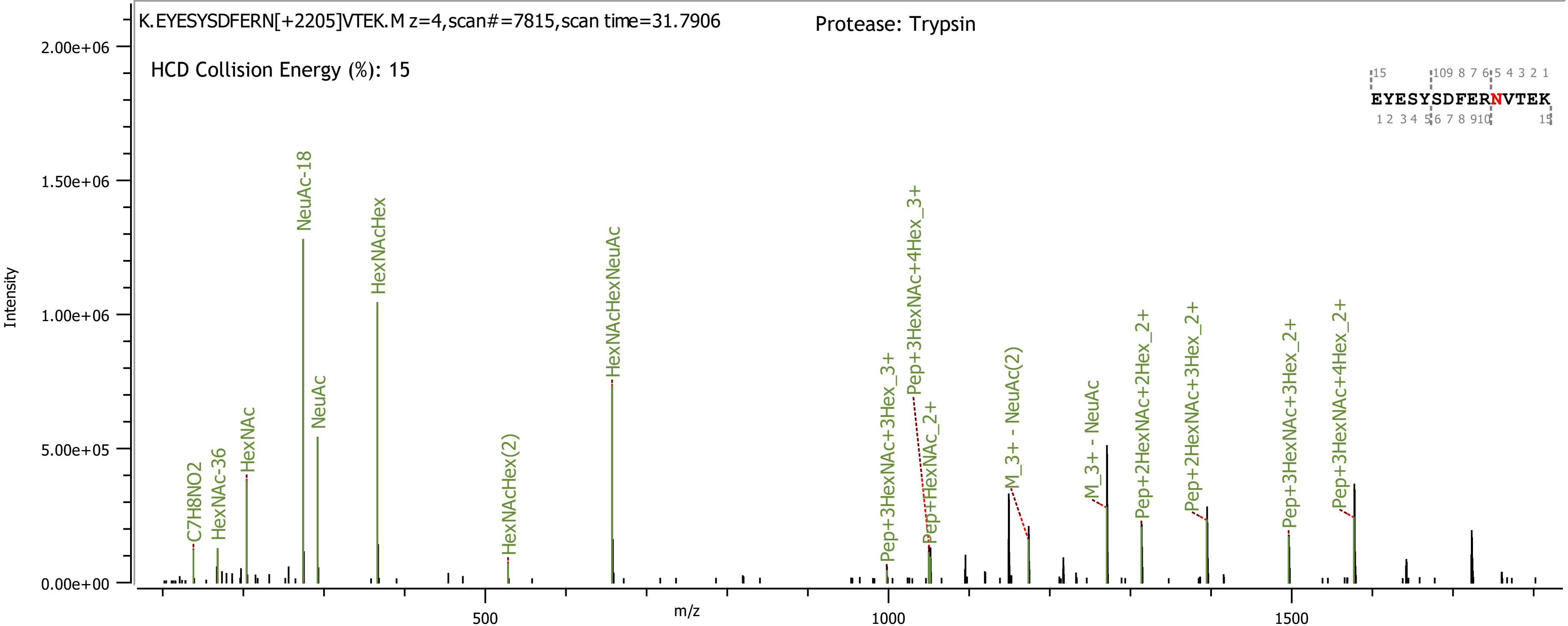

K.EYESYSDFERN[+2205]VTEK.M z=4,scan#=7815,scan time=31.7906
Protease: Trypsin
HCD Collision Energy (%): 15
EYESYSDFERNVTEK
Intensity
m/z
C7H8NO2
HexNAc-36
HexNAc
NeuAc-18
NeuAc
HexNAcHex
HexNAcHex(2)
HexNAcHexNeuAc
Pep+3HexNAc+3Hex_3+
Pep+3HexNAc+4Hex_3+
Pep+HexNAc_2+
M_3+ - NeuAc(2)
M_3+ - NeuAc
Pep+2HexNAc+2Hex_2+
Pep+2HexNAc+3Hex_2+
Pep+3HexNAc+3Hex_2+
Pep+3HexNAc+4Hex_2+

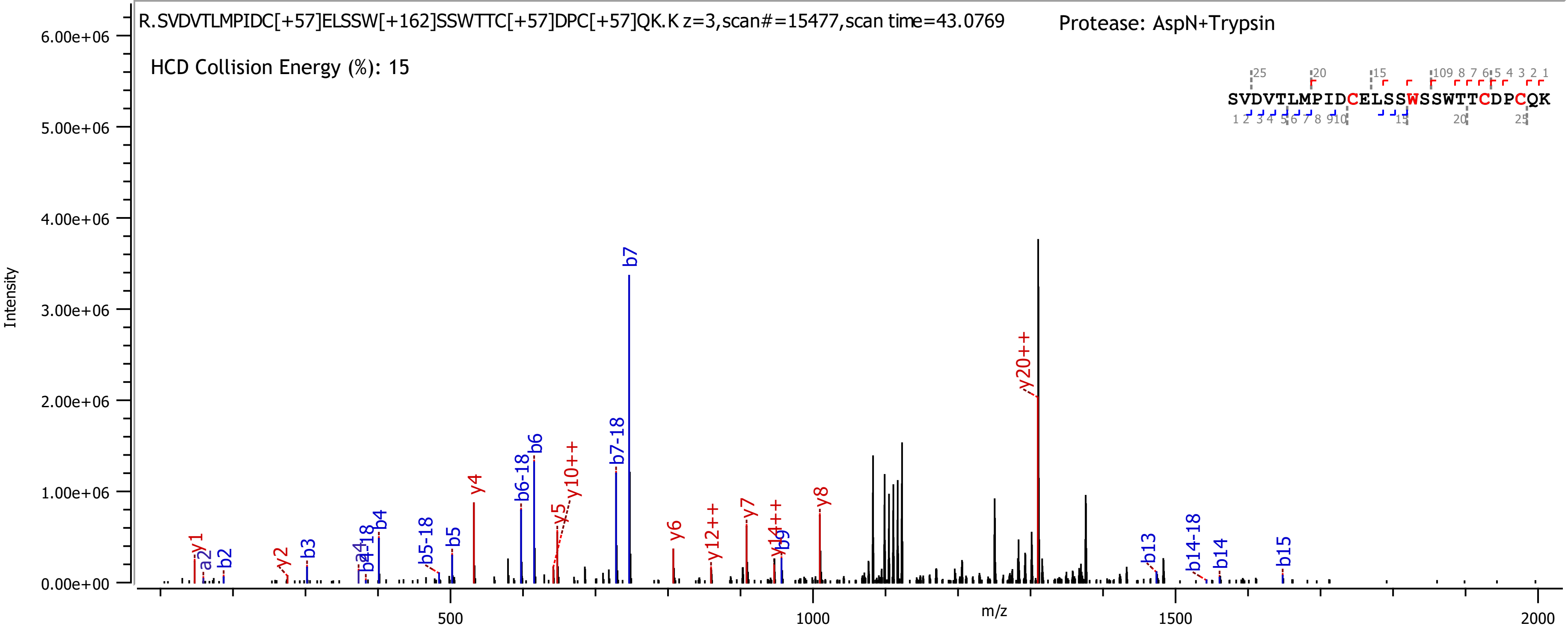
R.SVDVTLMPIDC[+57]ELSSW[+162]SSWTTC[+57]DPC[+57]QK.K z=3,scan#=15477,scan time=43.0769
Protease: AspN+Trypsin
HCD Collision Energy (%): 15
SVDVTLMPIDCELSSWSSWTTCDPCQK
Intensity
m/z
b7
y20++
b6
b6-18
b7-18
y10++
y4
y5
y8
y7
y14++
b9
y12++
y6
b4
b4-18
a4
b5
b5-18
b3
y2
y1
a2
b2
b13
b14-18
b14
b15

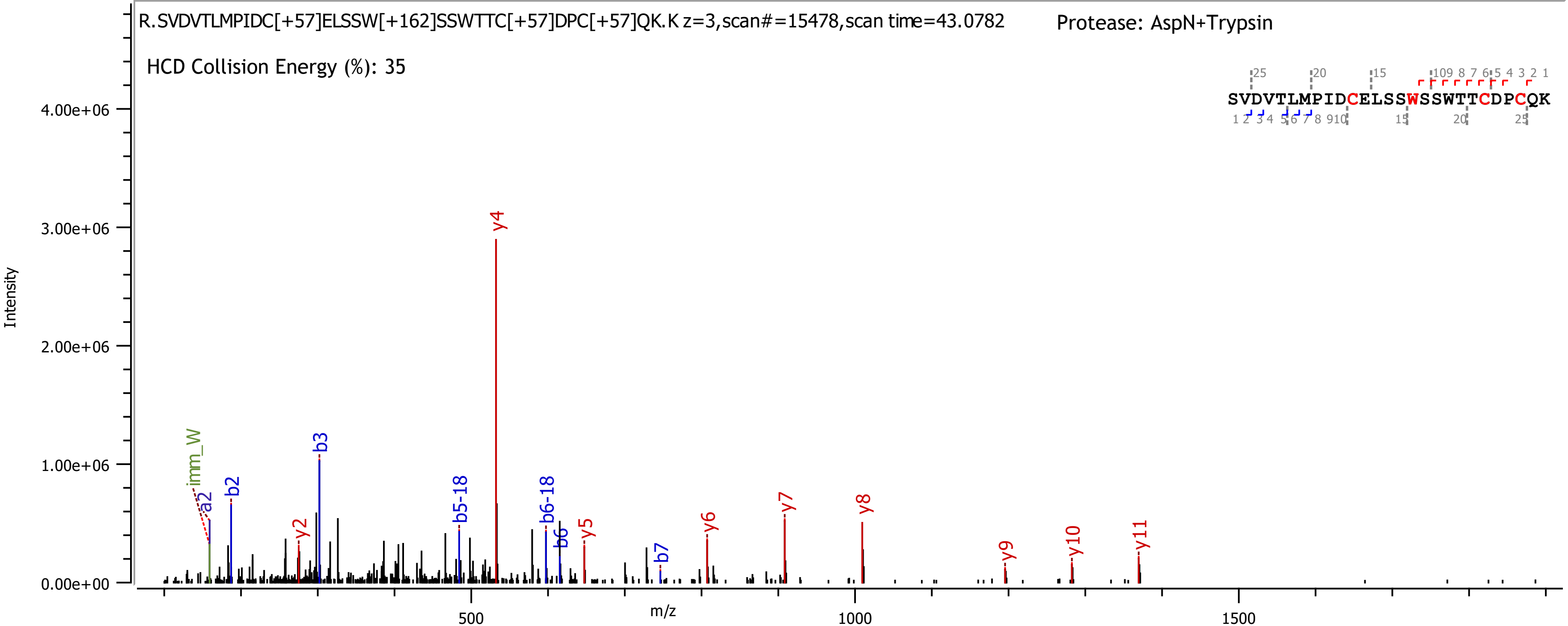

R.SVDVTLMPIDC[+57]ELSSW[+162]SSWTTC[+57]DPC[+57]QK.K z=3,scan#=15478,scan time=43.0782
Protease: AspN+Trypsin
HCD Collision Energy (%): 35
SVDVTLMPIDCELSSWSSWTTCDPCQK
Intensity
4.00e+06
3.00e+06
2.00e+06
1.00e+06
0.00e+00
m/z
500
1000
1500
imm_W
a2
b2
y2
b3
b5-18
y4
b6-18
b6
y5
b7
y6
y7
y8
y9
y10
y11

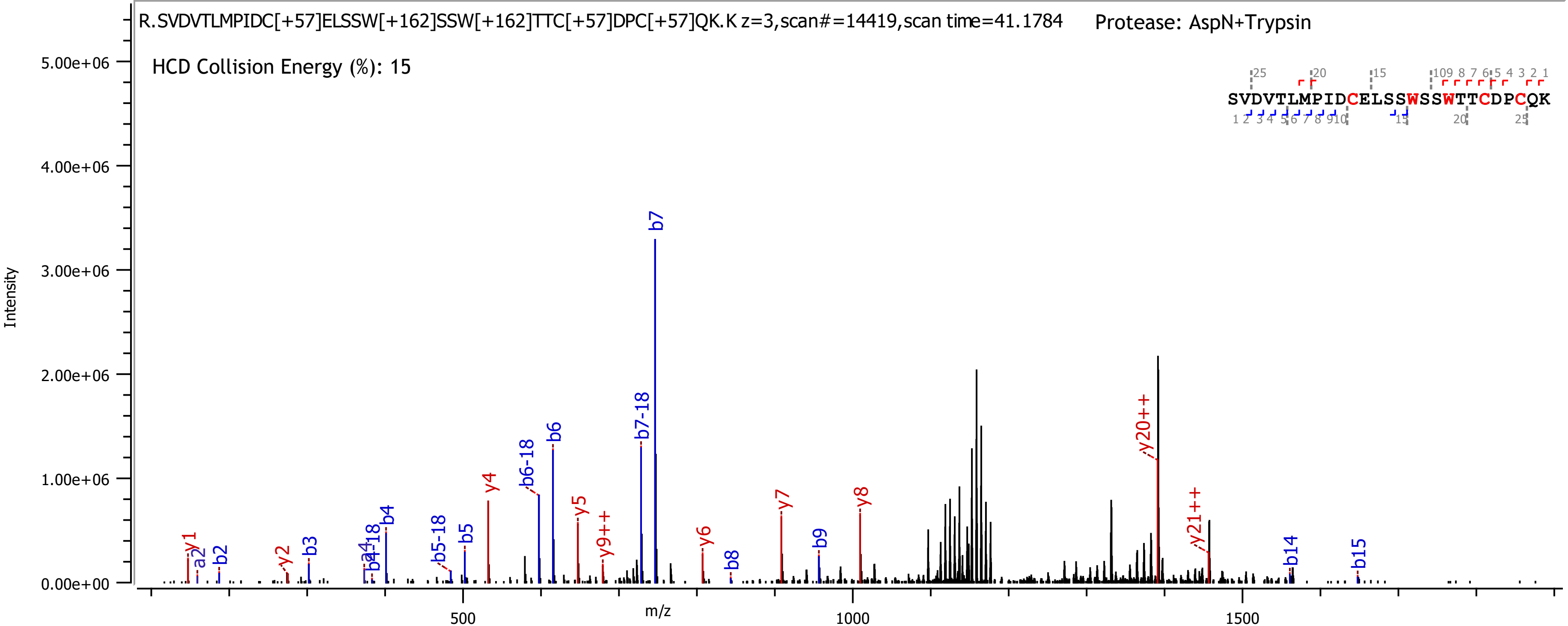
R.SVDVTLMPIDC[+57]ELSSW[+162]SSW[+162]TTC[+57]DPC[+57]QK.K z=3,scan#=14419,scan time=41.1784
Protease: AspN+Trypsin
HCD Collision Energy (%): 15
SVDVTLMPIDCELSSWSSWTTCDPCQK
Intensity
m/z
y1
a2
b2
y2
b3
a4
b4-18
b4
b5-18
b5
y4
b6-18
b6
y5
y9++
b7-18
b7
y6
b8
y7
b9
y8
y20++
y21++
b14
b15

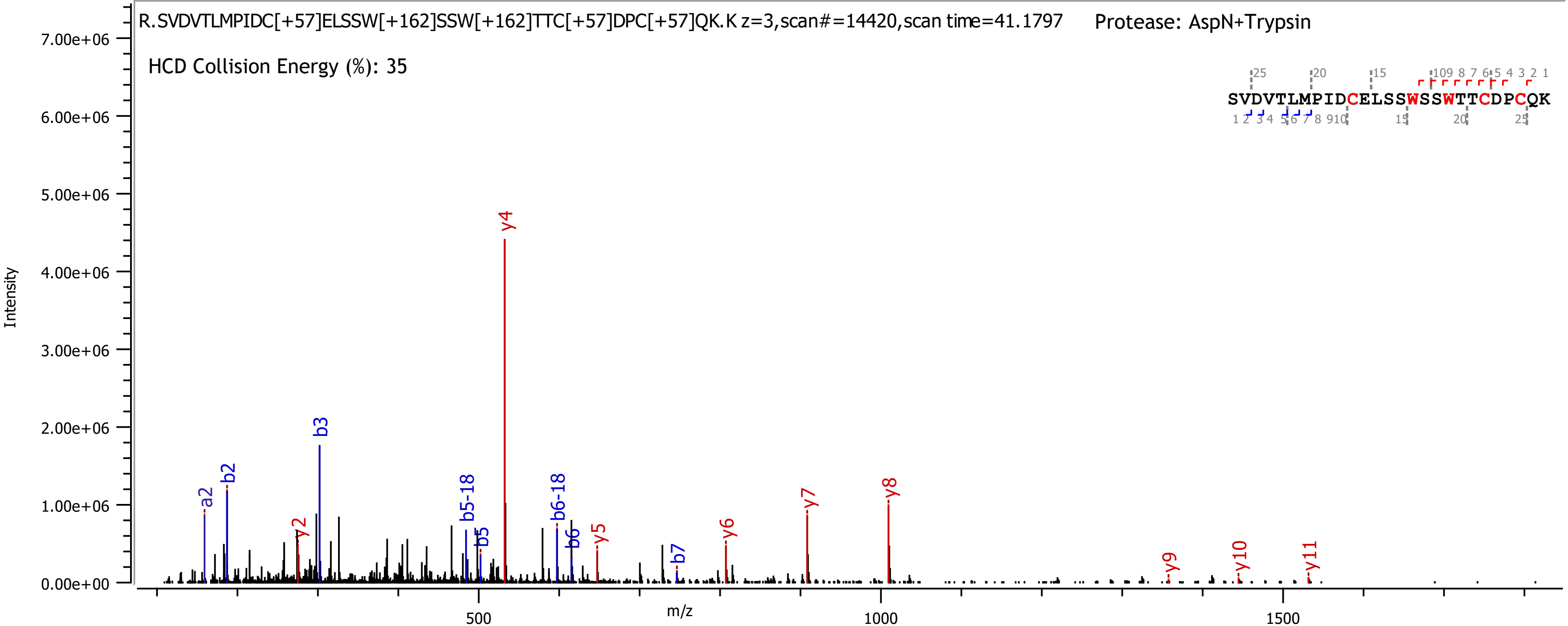
R.SVDVTLMPIDC[+57]ELSSW[+162]SSW[+162]TTC[+57]DPC[+57]QK.K z=3,scan#=14420,scan time=41.1797
Protease: AspN+Trypsin
HCD Collision Energy (%): 35
SVDVTLMPIDCELSSWSSWTTCDPCQK
Intensity
m/z
7.00e+06
6.00e+06
5.00e+06
4.00e+06
3.00e+06
2.00e+06
1.00e+06
0.00e+00
500
1000
1500
a2
b2
y2
b3
b5-18
b5
y4
b6-18
b6
y5
b7
y6
y7
y8
y9
y10
y11

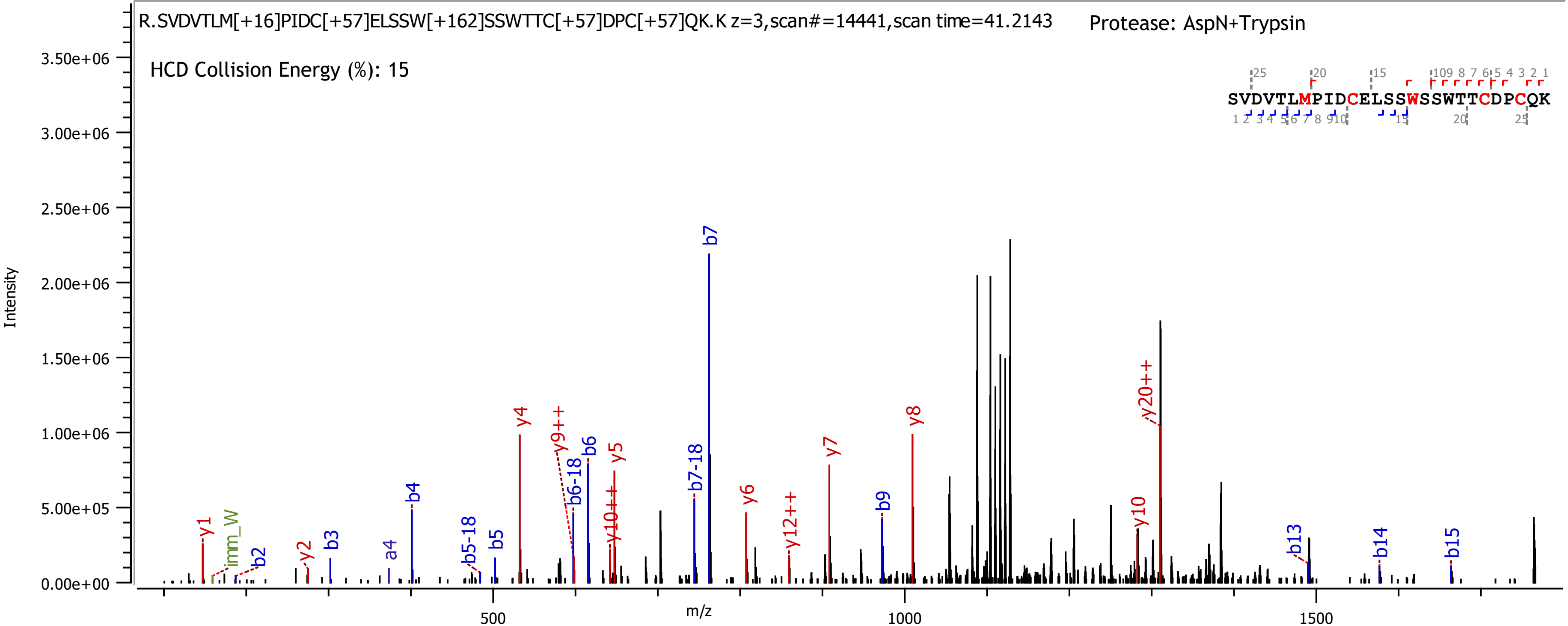

R.SVDVTLM[+16]PIDC[+57]ELSSW[+162]SSWTTC[+57]DPC[+57]QK.K z=3,scan#=14441,scan time=41.2143
Protease: AspN+Trypsin
HCD Collision Energy (%): 15
SVDVTLMPIDCELSSWSSWTTCDPCQK
Intensity
m/z
y1
imm_W
b2
y2
b3
a4
b4
b5-18
b5
y4
y9++
b6-18
b6
y5
y10++
b7-18
b7
y6
y12++
y7
b9
y8
y10
y20++
b13
b14
b15

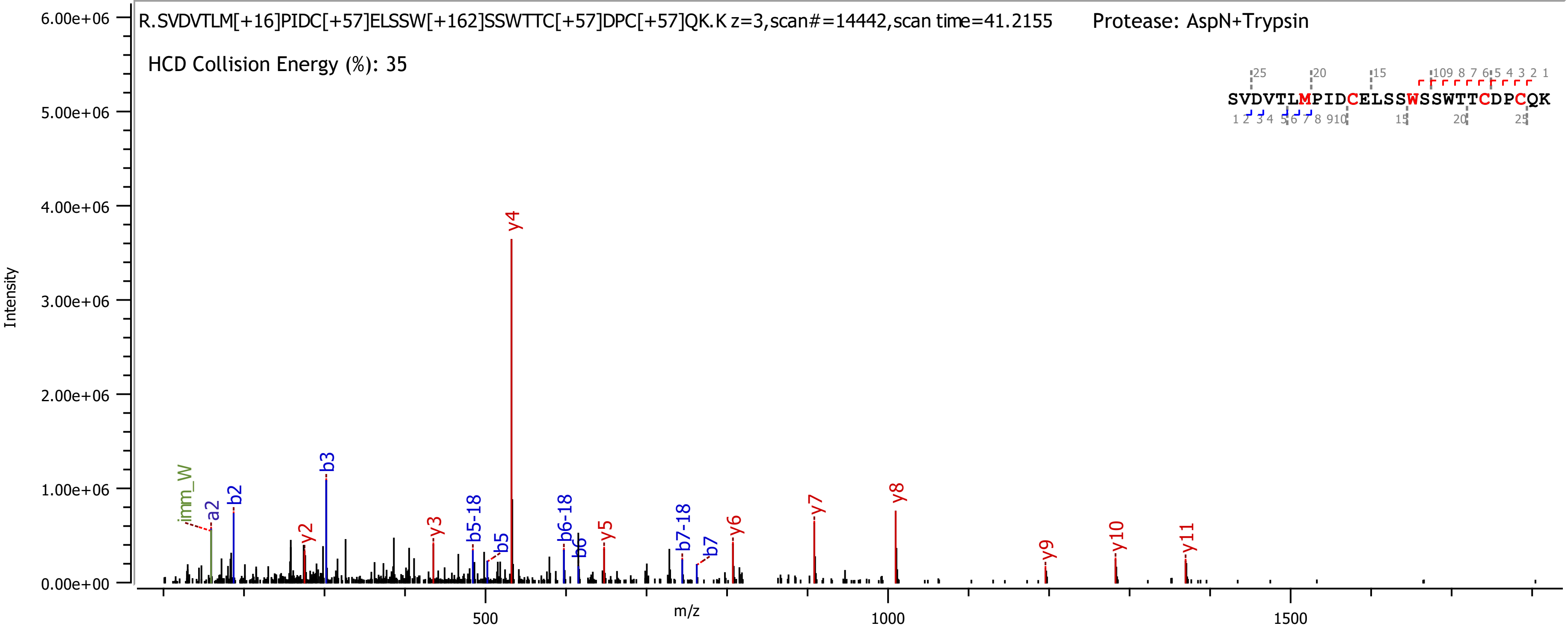
R.SVDVTLM[+16]PIDC[+57]ELSSW[+162]SSWTTC[+57]DPC[+57]QK.K z=3,scan#=14442,scan time=41.2155
Protease: AspN+Trypsin
HCD Collision Energy (%): 35
SVDVTLMPIDCELSSWSSWTTCDPCQK
Intensity
m/z
imm_W
a2
b2
b3
y2
y3
b5-18
b5
y4
b6-18
b6
y5
b7-18
b7
y6
y7
y8
y9
y10
y11

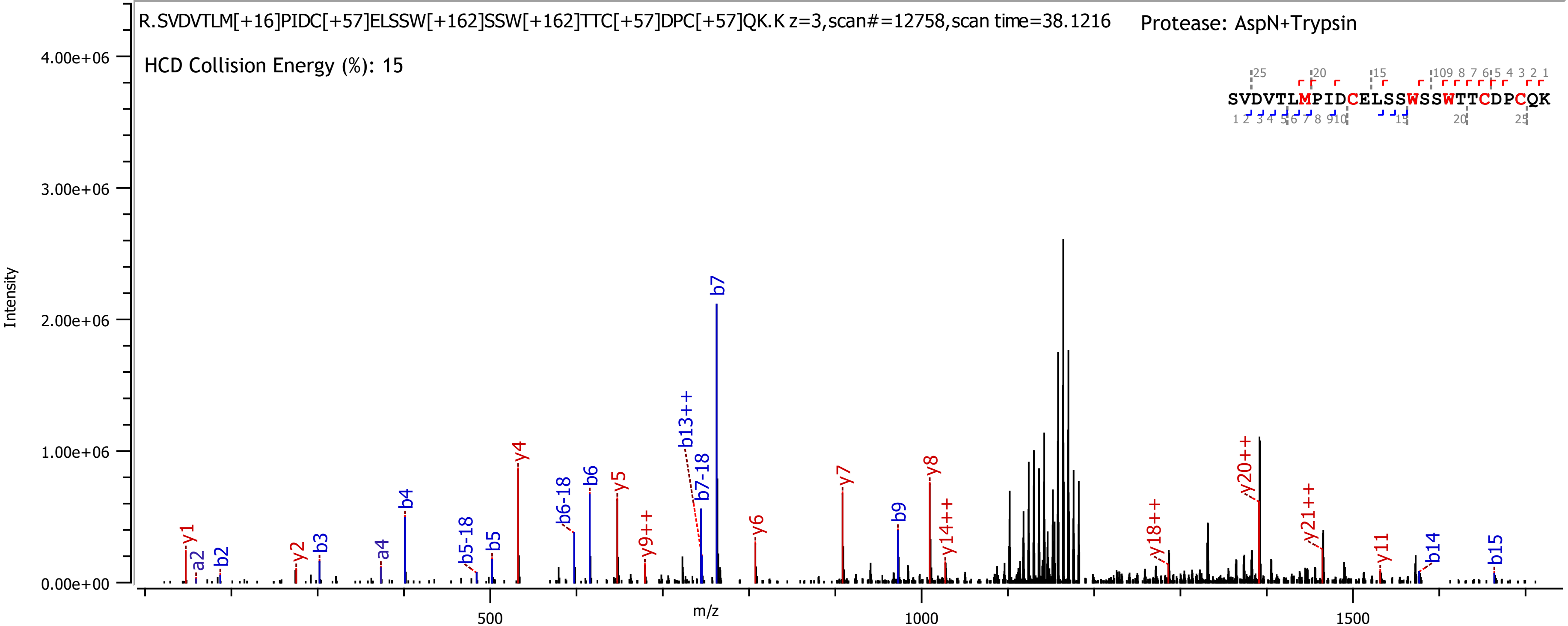
R.SVDVTLM[+16]PIDC[+57]ELSSW[+162]SSW[+162]TTC[+57]DPC[+57]QK.K z=3,scan#=12758,scan time=38.1216
Protease: AspN+Trypsin
HCD Collision Energy (%): 15
SVDVTLMPIDCELSSWSSWTTCDPCQK
Intensity
m/z
4.00e+06
3.00e+06
2.00e+06
1.00e+06
0.00e+00
500
1000
1500
y1
a2
b2
y2
b3
a4
b4
b5-18
b5
y4
b6-18
b6
y5
y9++
b13++
b7-18
b7
y6
y7
b9
y8
y14++
y18++
y20++
y21++
y11
b14
b15

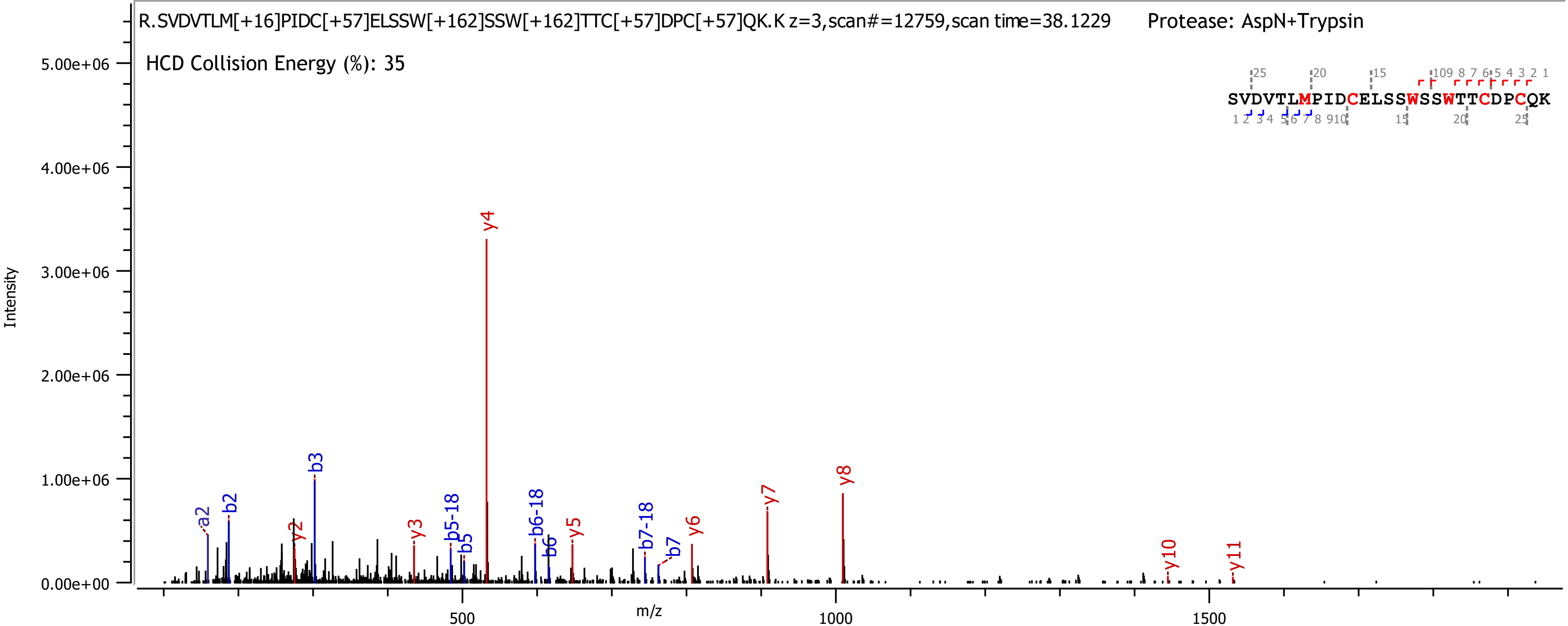
R.SVDVTLM[+16]PIDC[+57]ELSSW[+162]SSW[+162]TTC[+57]DPC[+57]QK.K z=3,scan#=12759,scan time=38.1229
Protease: AspN+Trypsin
HCD Collision Energy (%): 35
SVDVTLMPIDCELSSWSSWTTCDPCQK
Intensity
m/z
5.00e+06
4.00e+06
3.00e+06
2.00e+06
1.00e+06
0.00e+00
500
1000
1500
a2
b2
y2
b3
y3
b5-18
b5
y4
b6-18
b6
y5
b7-18
b7
y6
y7
y8
y10
y11

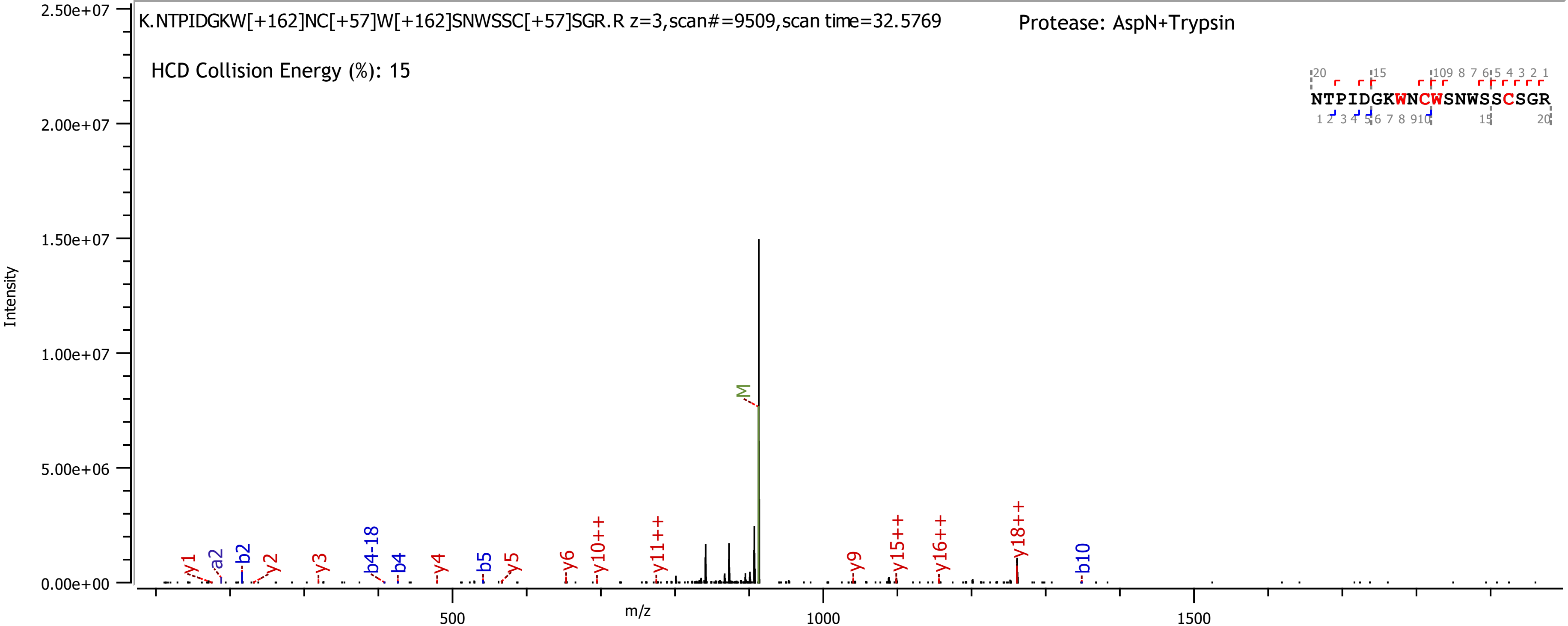
K.NTPIDGKW[+162]NC[+57]W[+162]SNWSSC[+57]SGR.R z=3,scan#=9509,scan time=32.5769
Protease: AspN+Trypsin
HCD Collision Energy (%): 15
NTPIDGKWNCWSNWSSCSGR
Intensity
m/z
2.50e+07
2.00e+07
1.50e+07
1.00e+07
5.00e+06
0.00e+00
500
1000
1500
M
y1
a2
b2
y2
y3
b4-18
b4
y4
b5
y5
y6
y10++
y11++
y9
y15++
y16++
y18++
b10

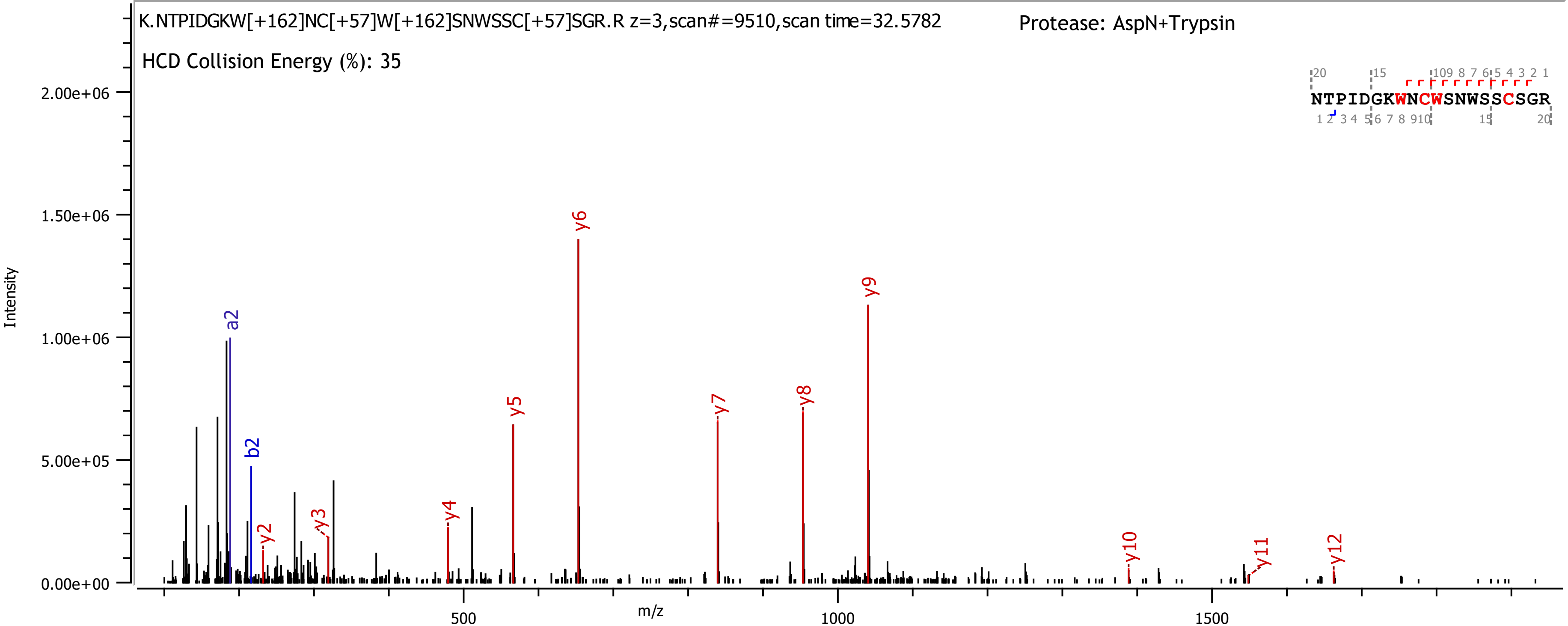

K.NTPIDGKW[+162]NC[+57]W[+162]SNWSSC[+57]SGR.R z=3,scan#=9510,scan time=32.5782
Protease: AspN+Trypsin
HCD Collision Energy (%): 35
NTPIDGKWNCWSNWSSCSGR
Intensity
2.00e+06
1.50e+06
1.00e+06
5.00e+05
0.00e+00
a2
b2
y2
y3
y4
y5
y6
y7
y8
y9
y10
y11
y12
m/z
500
1000
1500

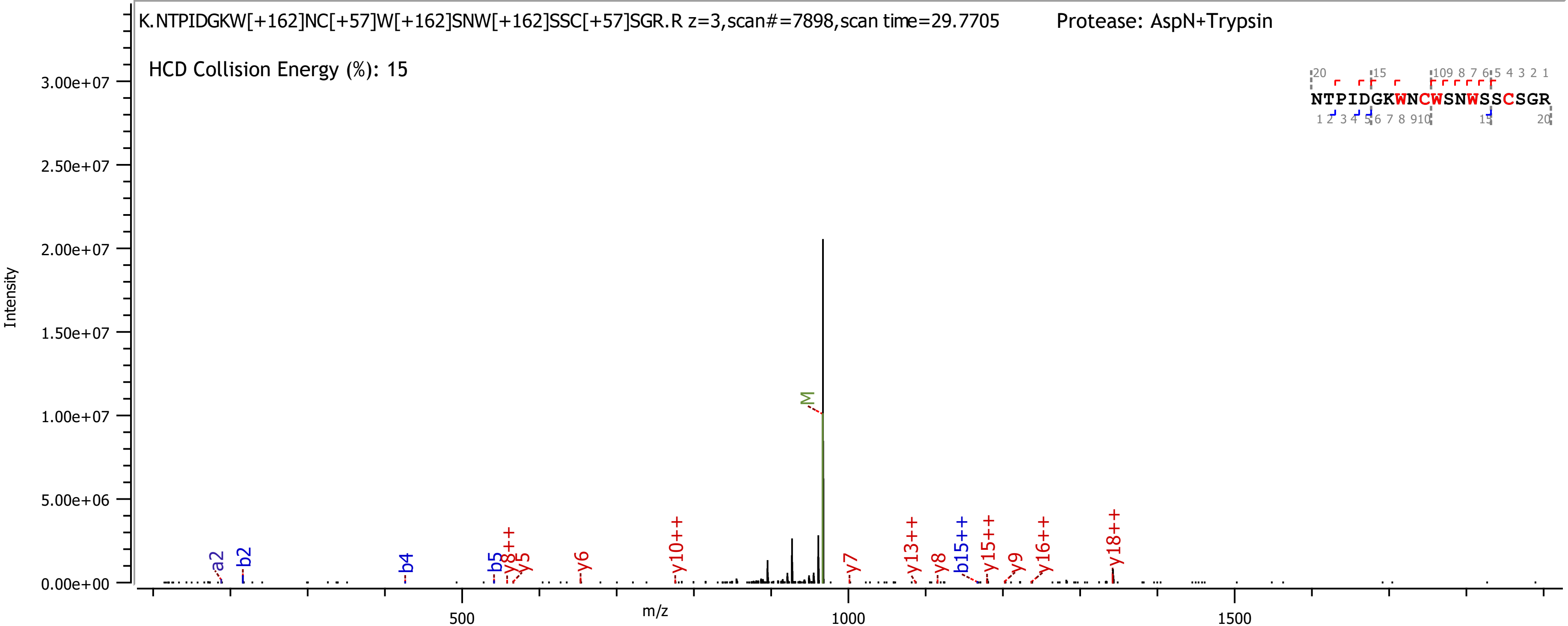

K.NTPIDGKW[+162]NC[+57]W[+162]SNW[+162]SSC[+57]SGR.R z=3,scan#=7898,scan time=29.7705
Protease: AspN+Trypsin
HCD Collision Energy (%): 15
NTPIDGKWNCWSNWSSCSGR
Intensity
m/z
M
a2
b2
b4
b5
y8++
y5
y6
y10++
y7
y13++
y8
b15++
y15++
y9
y16++
y18++

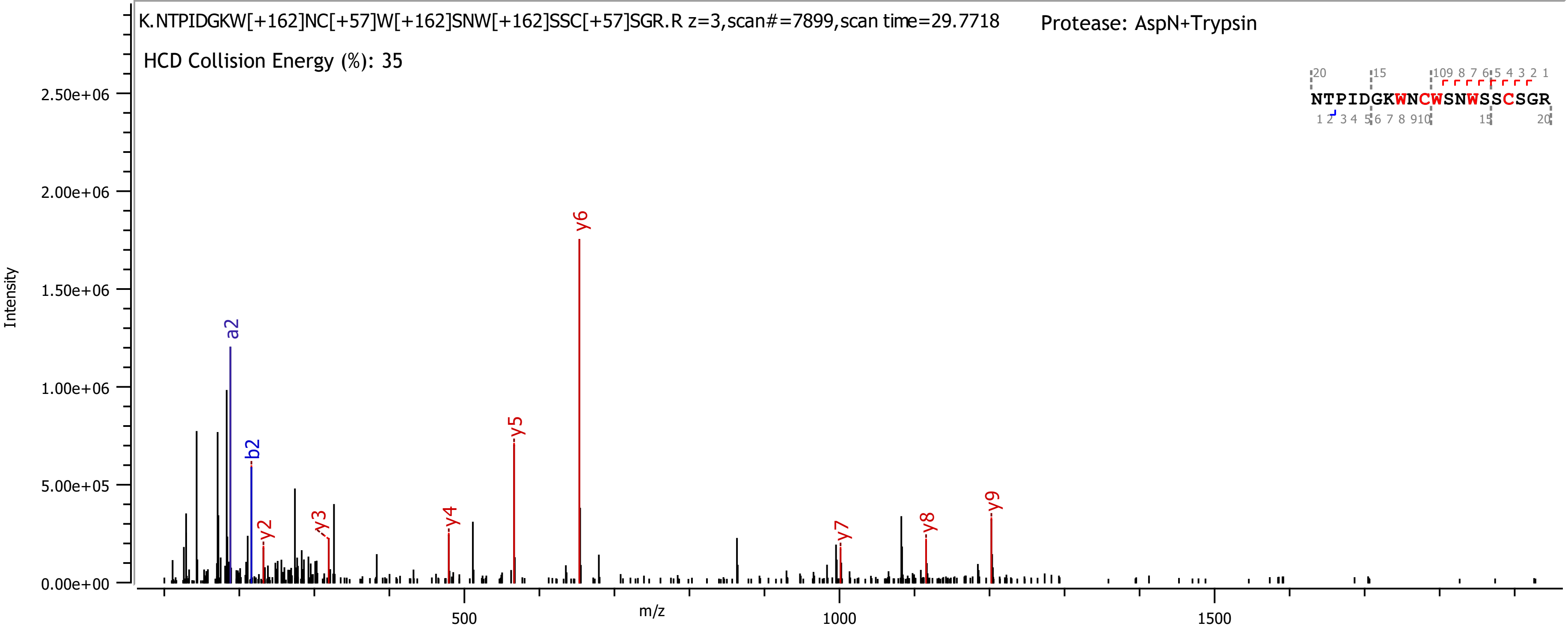

K.NTPIDGKW[+162]NC[+57]W[+162]SNW[+162]SSC[+57]SGR.R z=3,scan#=7899,scan time=29.7718
Protease: AspN+Trypsin
HCD Collision Energy (%): 35
NTPIDGKWNCWSNWSSCSGR
Intensity
m/z
a2
b2
y2
y3
y4
y5
y6
y7
y8
y9
2.50e+06
2.00e+06
1.50e+06
1.00e+06
5.00e+05
0.00e+00
500
1000
1500

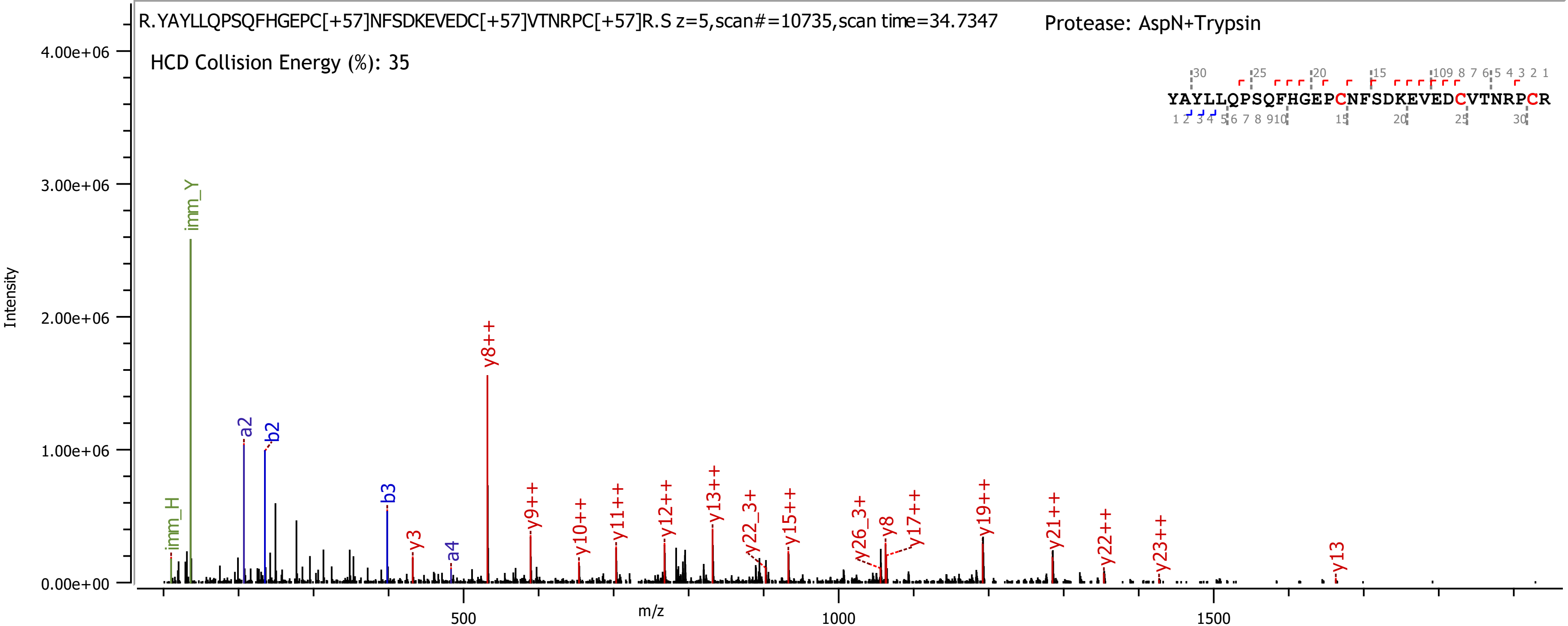
R.YAYLLQPSQFHGEPC[+57]NFSDKEVEDC[+57]VTNRPC[+57]R.S z=5,scan#=10735,scan time=34.7347
Protease: AspN+Trypsin
HCD Collision Energy (%): 35
YAYLLQPSQFHGEPCNFSDKEVEDCVTNRPCR
Intensity
m/z
imm_Y
imm_H
a2
b2
b3
y3
a4
y8++
y9++
y10++
y11++
y12++
y13++
y22_3+
y15++
y26_3+
y8
y17++
y19++
y21++
y22++
y23++
y13

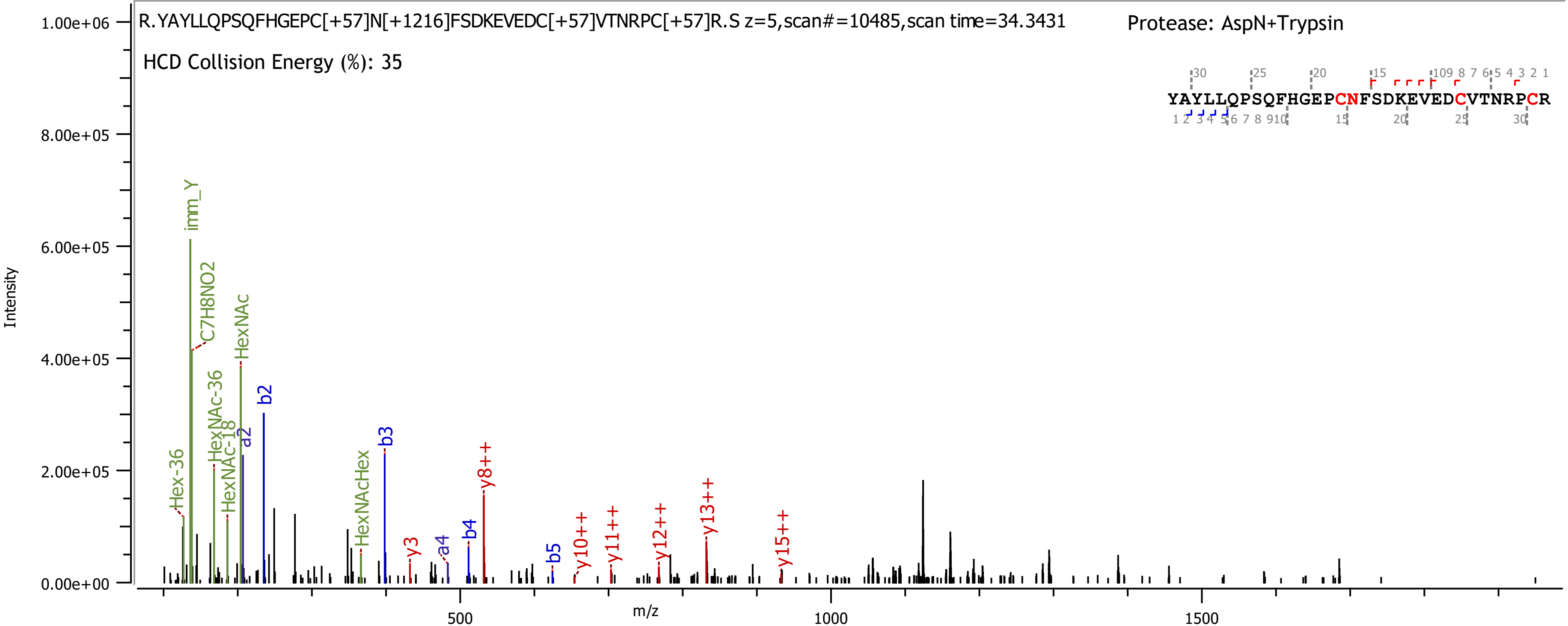

R.YAYLLQPSQFHGEPC[+57]N[+1216]FSDKEVEDC[+57]VTNRPC[+57]R.S z=5,scan#=10485,scan time=34.3431
Protease: AspN+Trypsin
HCD Collision Energy (%): 35
YAYLLQPSQFHGEPCNFSDKEVEDCVTNRPCR
Intensity
m/z
1.00e+06
8.00e+05
6.00e+05
4.00e+05
2.00e+05
0.00e+00
500
1000
1500
imm_Y
C7H8NO2
HexNAc
HexNAc-36
HexNAc-18
Hex-36
HexNAcHex
a2
b2
b3
a4
b4
b5
y3
y8++
y10++
y11++
y12++
y13++
y15++

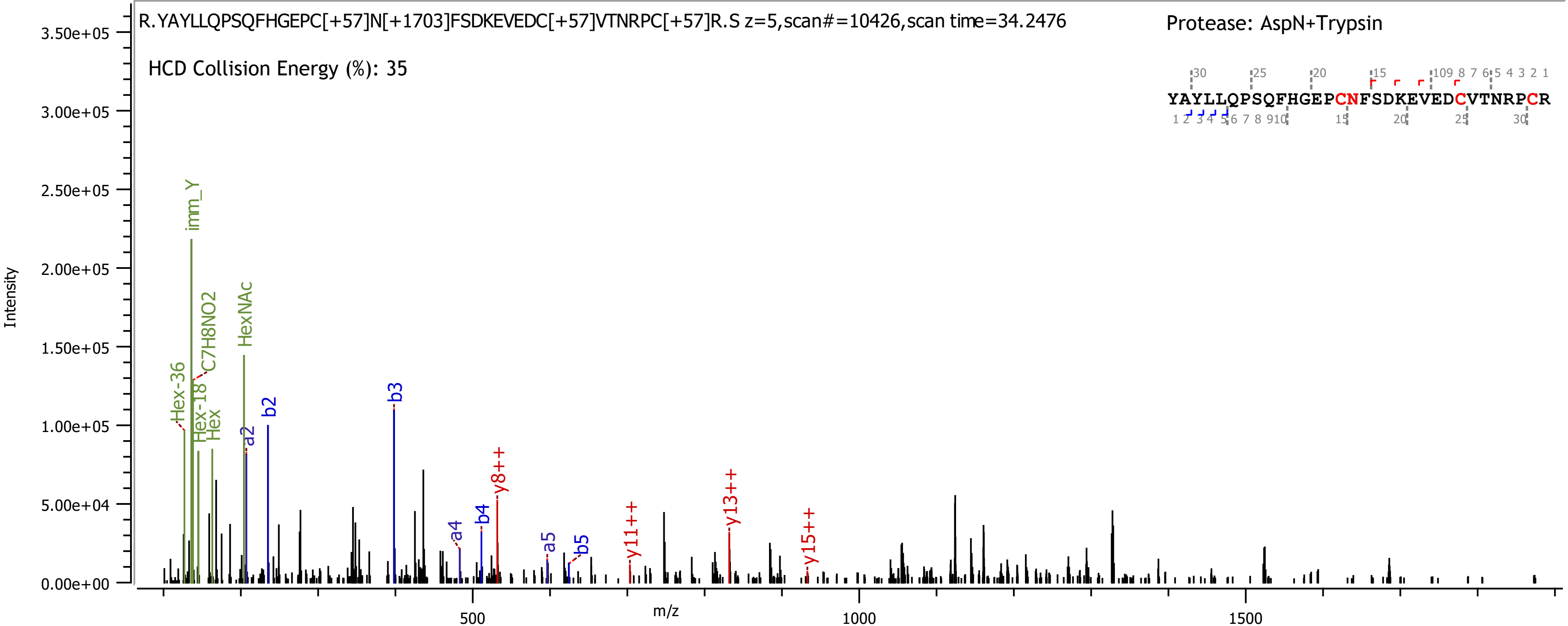

R.YAYLLQPSQFHGEPC[+57]N[+1703]FSDKEVEDC[+57]VTNRPC[+57]R.S z=5,scan#=10426,scan time=34.2476
Protease: AspN+Trypsin
HCD Collision Energy (%): 35
YAYLLQPSQFHGEPCNFSDKEVEDCVTNRPCR
Intensity
m/z
imm_Y
C7H8NO2
HexNAc
Hex-36
Hex-18
Hex
a2
b2
b3
a4
b4
y8++
a5
b5
y11++
y13++
y15++

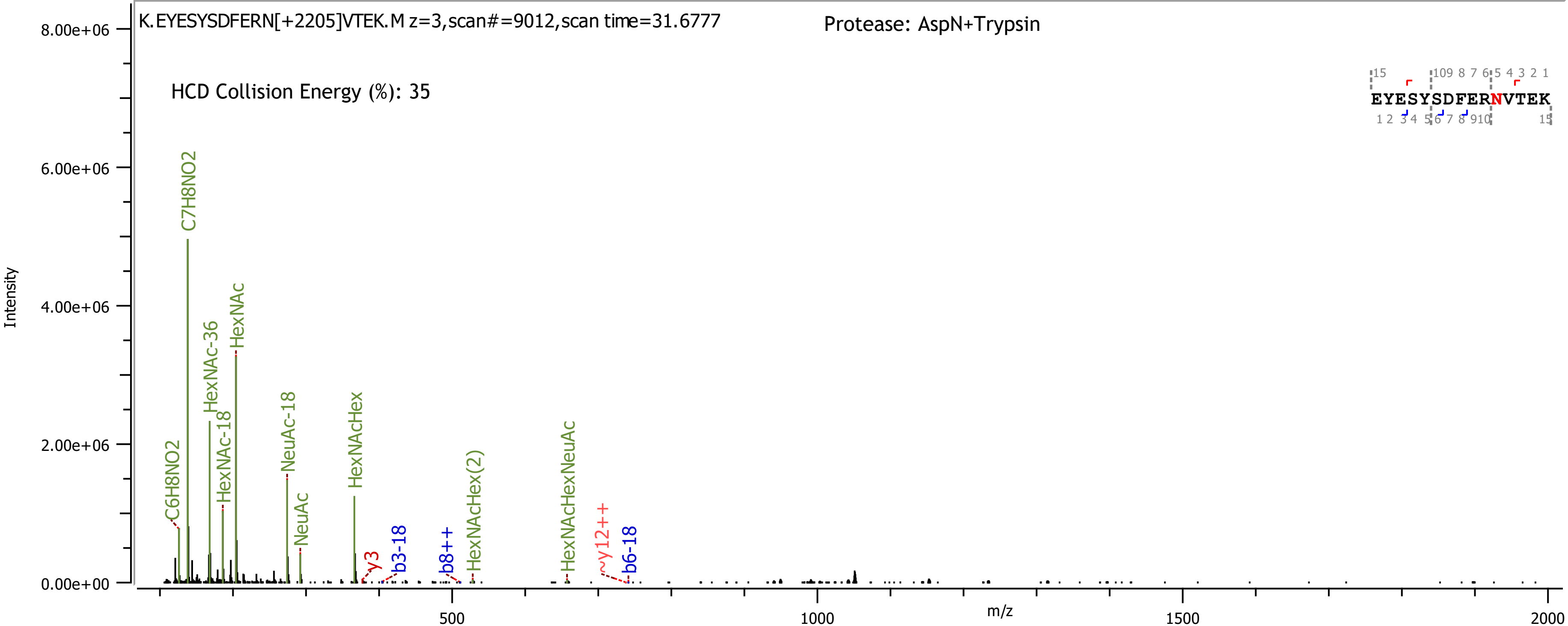
K.EYESYSDFERN[+2205]VTEK.M z=3,scan#=9012,scan time=31.6777
Protease: AspN+Trypsin
HCD Collision Energy (%): 35
EYESYSDFERNVTEK
Intensity
m/z
8.00e+06
6.00e+06
4.00e+06
2.00e+06
0.00e+00
500
1000
1500
2000
C6H8NO2
C7H8NO2
HexNAc-36
HexNAc-18
HexNAc
NeuAc-18
NeuAc
HexNAcHex
y3
b3-18
b8++
HexNAcHex(2)
HexNAcHexNeuAc
y12++
b6-18

C8 gamma subunit: MS/MS spectra of all PTM modified peptides

EThcD spectra

Glycosylation Site

T35/S34	RPASPISTIQPK
	RPASPIS*T*[+656.2]IQPK
	RPASPIS*T*[+365.1]IQPK
	RPASPIS*T*[+203.1]IQPK

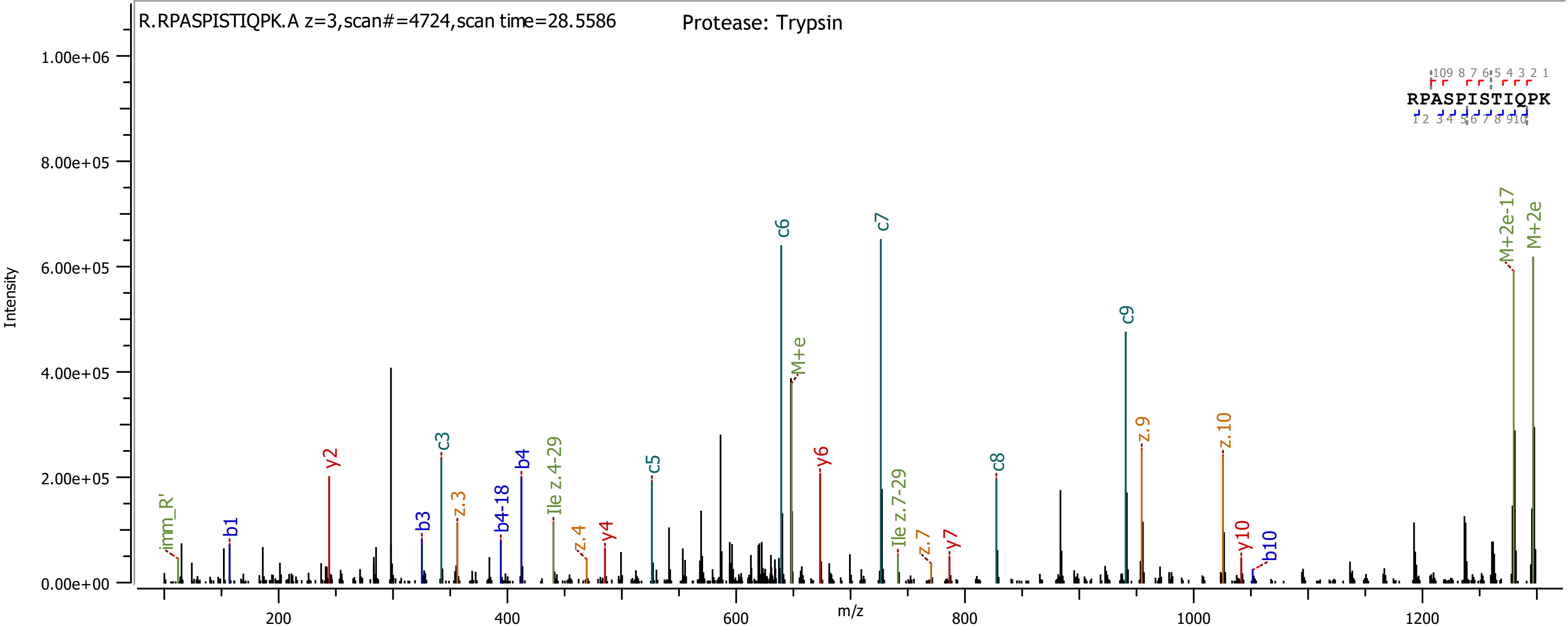

R.RPASPISTIQPK.A z=3,scan#=4724,scan time=28.5586
Protease: Trypsin
RPASPISTIQPK
Intensity
m/z
1.00e+06
8.00e+05
6.00e+05
4.00e+05
2.00e+05
0.00e+00
200
400
600
800
1000
1200
imm_R'
b1
y2
b3
c3
z.3
b4-18
b4
Ile z.4-29
z.4
y4
c5
c6
M+e
y6
c7
Ile z.7-29
z.7
y7
c8
c9
z.9
z.10
y10
b10
M+2e-17
M+2e

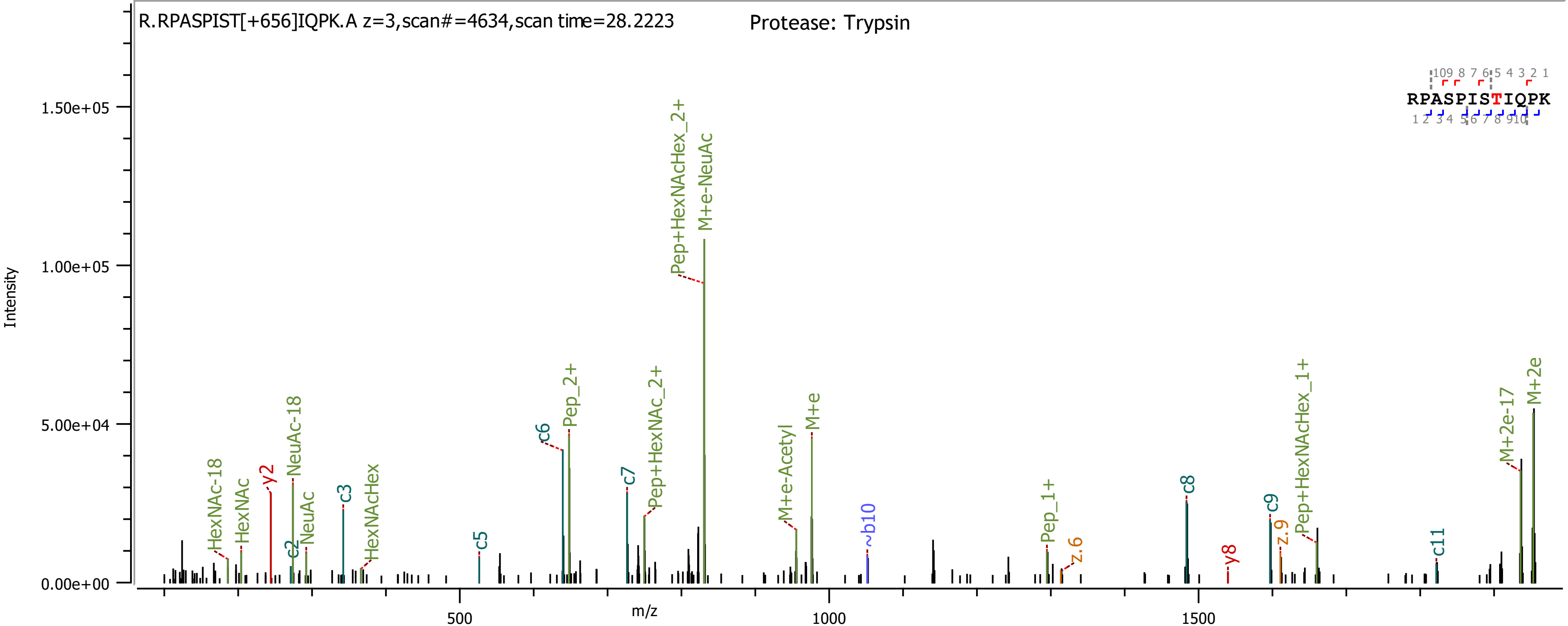

R.RPASPIST[+656]IQPK.A z=3,scan#=4634,scan time=28.2223
Protease: Trypsin
RPASPISTIQPK
Intensity
m/z
0.00e+00
5.00e+04
1.00e+05
1.50e+05
500
1000
1500
HexNAc-18
HexNAc
y2
c2
NeuAc-18
NeuAc
c3
HexNAcHex
c5
c6
Pep_2+
c7
Pep+HexNAc_2+
Pep+HexNAcHex_2+
M+e-NeuAc
M+e-Acetyl
M+e
~b10
Pep_1+
z.6
c8
y8
c9
z.9
Pep+HexNAcHex_1+
c11
M+2e-17
M+2e

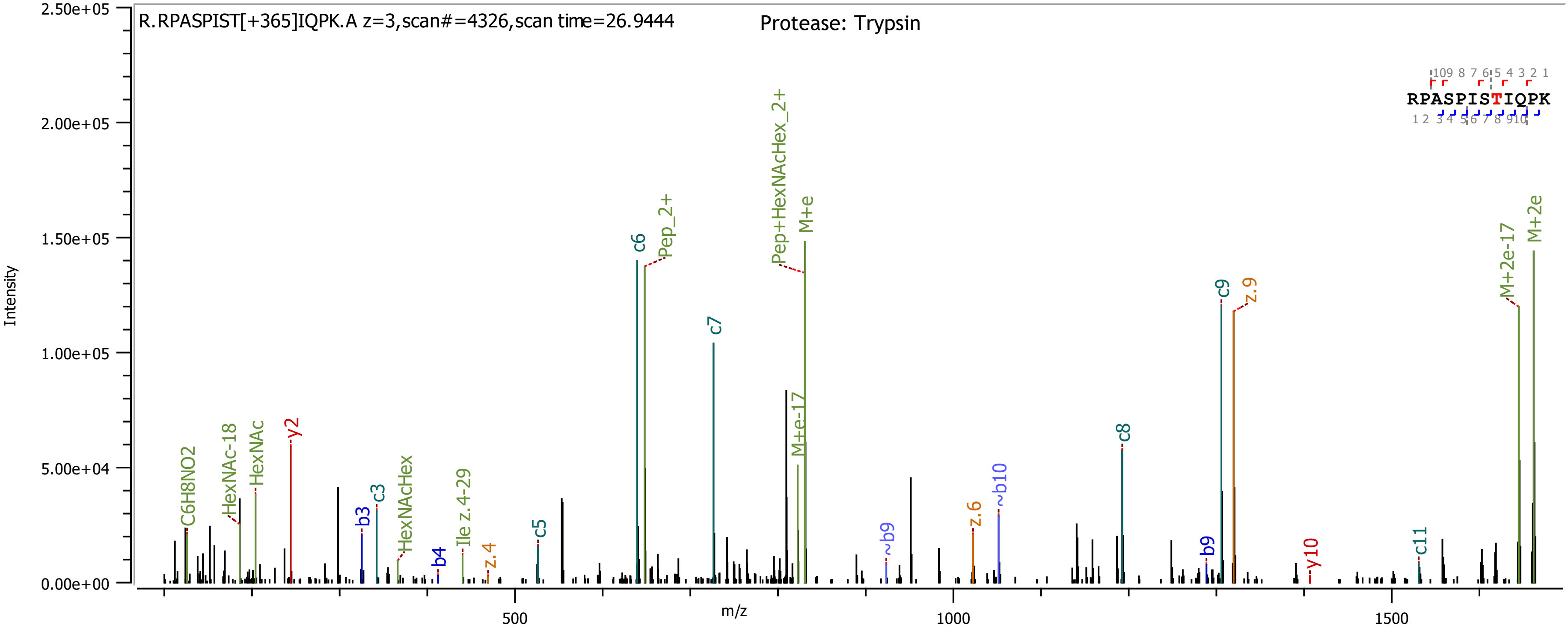

R.RPASPIST[+365]IQPK.A z=3,scan#=4326,scan time=26.9444
Protease: Trypsin
RPASPISTIQPK
Intensity
m/z
2.50e+05
2.00e+05
1.50e+05
1.00e+05
5.00e+04
0.00e+00
500
1000
1500
C6H8NO2
HexNAc-18
HexNAc
y2
b3
c3
HexNAcHex
b4
Ile z.4-29
z.4
c5
c6
Pep_2+
c7
Pep+HexNAcHex_2+
M+e-17
M+e
~b9
z.6
~b10
c8
b9
c9
z.9
y10
c11
M+2e-17
M+2e

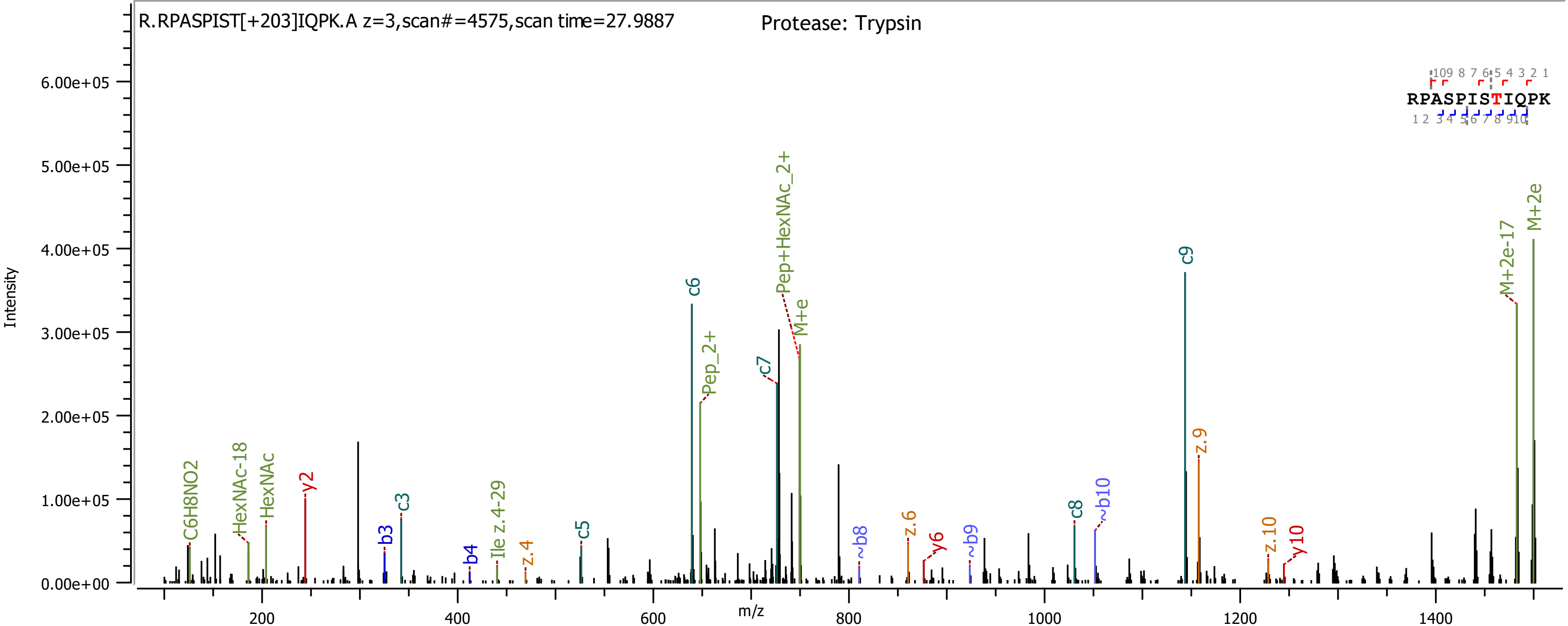

R.RPASPIST[+203]IQPK.A z=3,scan#=4575,scan time=27.9887
Protease: Trypsin
RPASPISTIQPK
Intensity
m/z
6.00e+05
5.00e+05
4.00e+05
3.00e+05
2.00e+05
1.00e+05
0.00e+00
200
400
600
800
1000
1200
1400
C6H8NO2
HexNAc-18
HexNAc
y2
b3
c3
b4
Ile z.4-29
z.4
c5
c6
Pep_2+
c7
Pep+HexNAc_2+
M+e
~b8
z.6
y6
~b9
c8
~b10
c9
z.9
z.10
y10
M+2e-17
M+2e

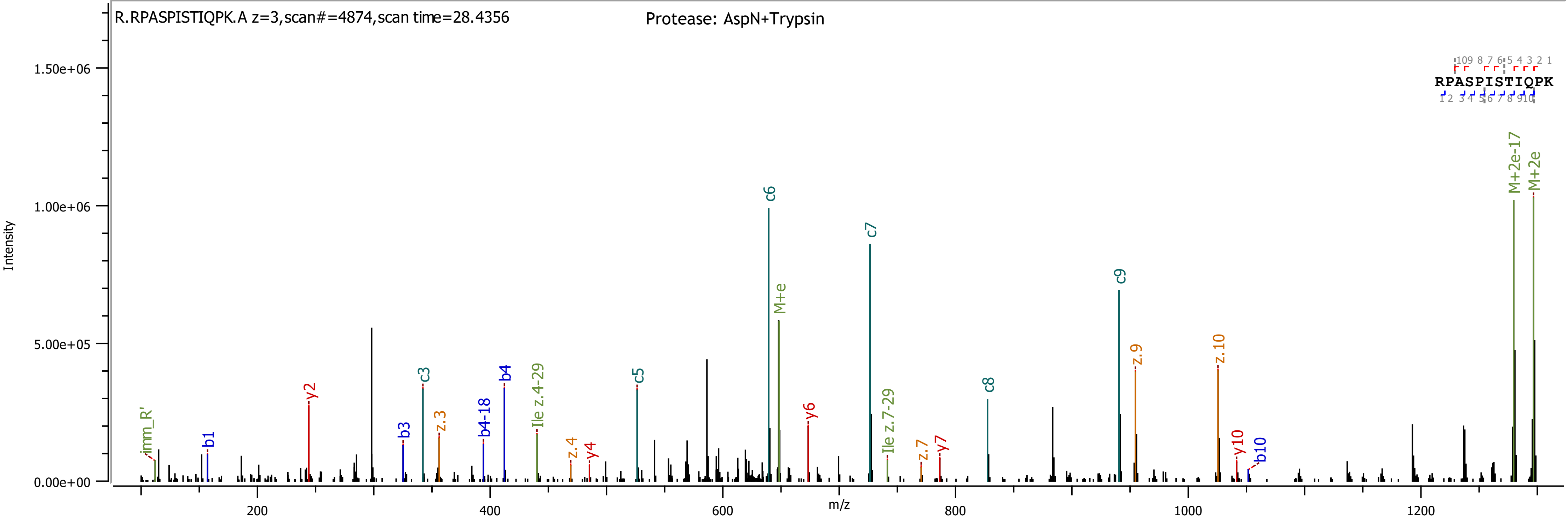
R.RPASPISTIQPK.A z=3,scan#=4874,scan time=28.4356
Protease: AspN+Trypsin
RPASPISTIQPK
Intensity
m/z
imm_R'
b1
y2
b3
c3
z.3
b4-18
b4
Ile z.4-29
z.4
y4
c5
c6
M+e
y6
c7
Ile z.7-29
z.7
y7
c8
c9
z.9
z.10
y10
b10
M+2e-17
M+2e
1.50e+06
1.00e+06
5.00e+05
0.00e+00
200
400
600
800
1000
1200

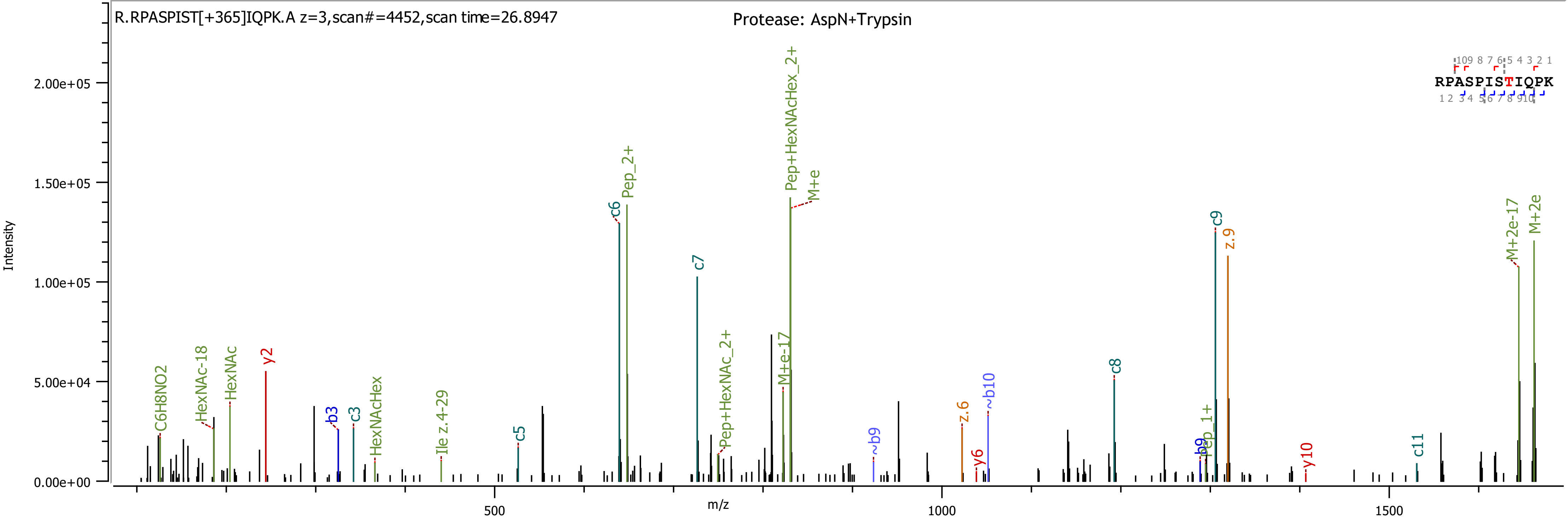
R.RPASPIST[+365]IQPK.A z=3,scan#=4452,scan time=26.8947
Protease: AspN+Trypsin
RPASPISTIQPK
Intensity
m/z
0.00e+00
5.00e+04
1.00e+05
1.50e+05
2.00e+05
500
1000
1500
C6H8NO2
HexNAc-18
HexNAc
y2
b3
c3
HexNAcHex
Ile z.4-29
c5
c6
Pep_2+
c7
Pep+HexNAc_2+
M+e-17
Pep+HexNAcHex_2+
M+e
~b9
z.6
y6
~b10
c8
b9
Pep_1+
c9
z.9
y10
c11
M+2e-17
M+2e

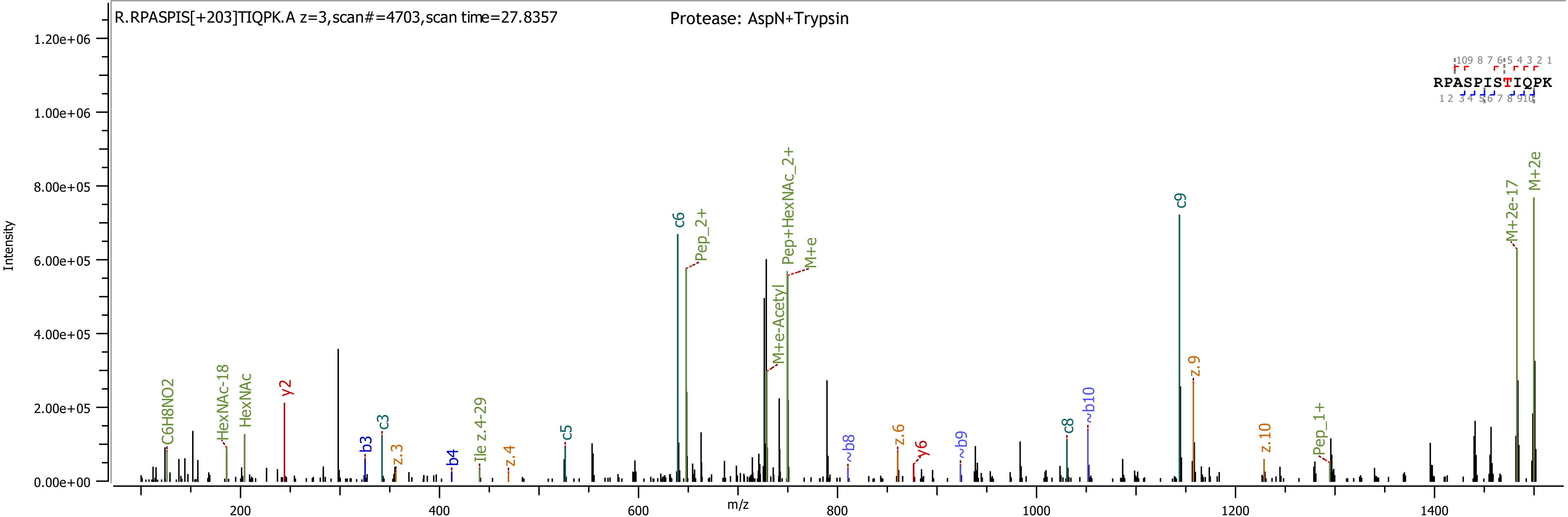
R.RPASPIS[+203]TIQPK.A z=3,scan#=4703,scan time=27.8357
Protease: AspN+Trypsin
RPASPISTIQPK
Intensity
m/z
C6H8NO2
HexNAc-18
HexNAc
y2
b3
c3
z.3
b4
Ile z.4-29
z.4
c5
c6
Pep_2+
M+e-Acetyl
Pep+HexNAc_2+
M+e
~b8
z.6
y6
~b9
c8
~b10
c9
z.9
z.10
Pep_1+
M+2e-17
M+2e

HCD spectra

Glycosylation Site

T35/S34	RPASPISTIQPK
	RPASPIS*T*[+203.1]IQPK
	RPASPIS*T*[+365.1]IQPK

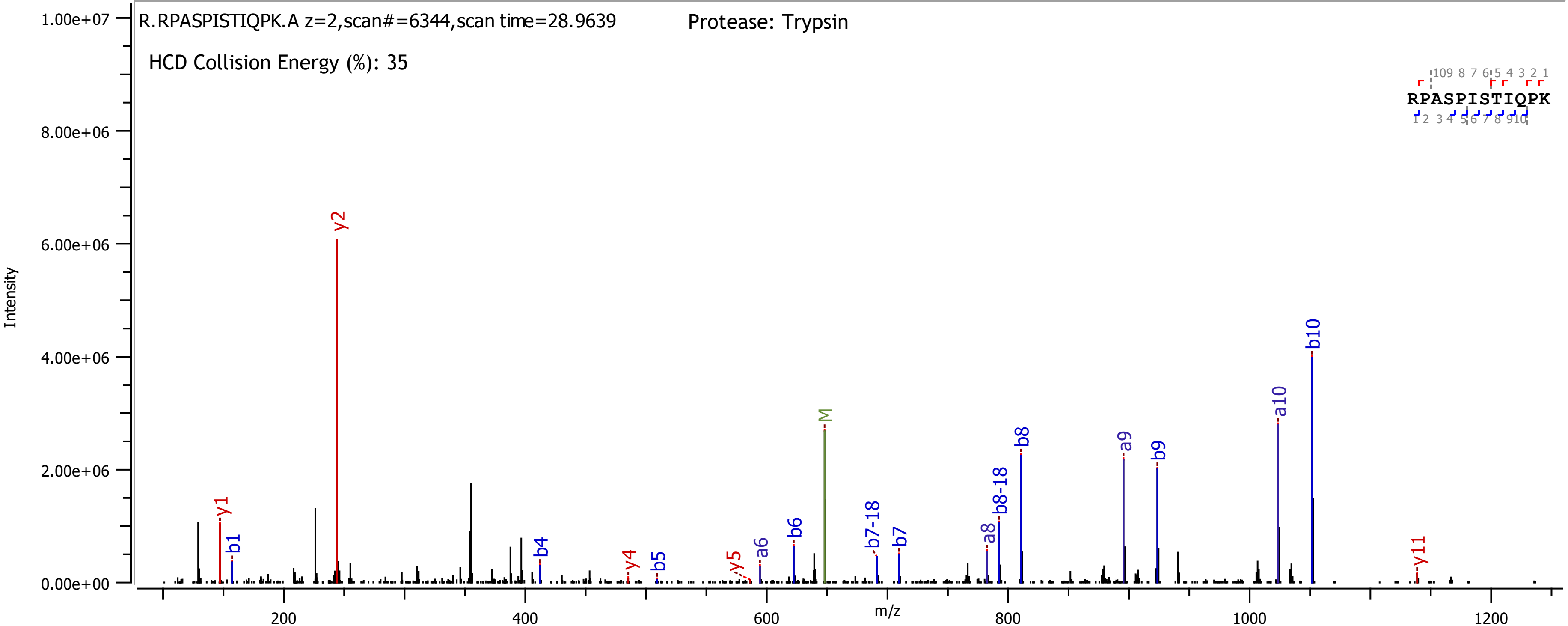

R.RPASPISTIQPK.A z=2,scan#=6344,scan time=28.9639
Protease: Trypsin
HCD Collision Energy (%): 35
RPASPISTIQPK
Intensity
m/z
y1
b1
y2
b4
y4
b5
y5
a6
b6
M
b7-18
b7
a8
b8-18
b8
a9
b9
a10
b10
y11

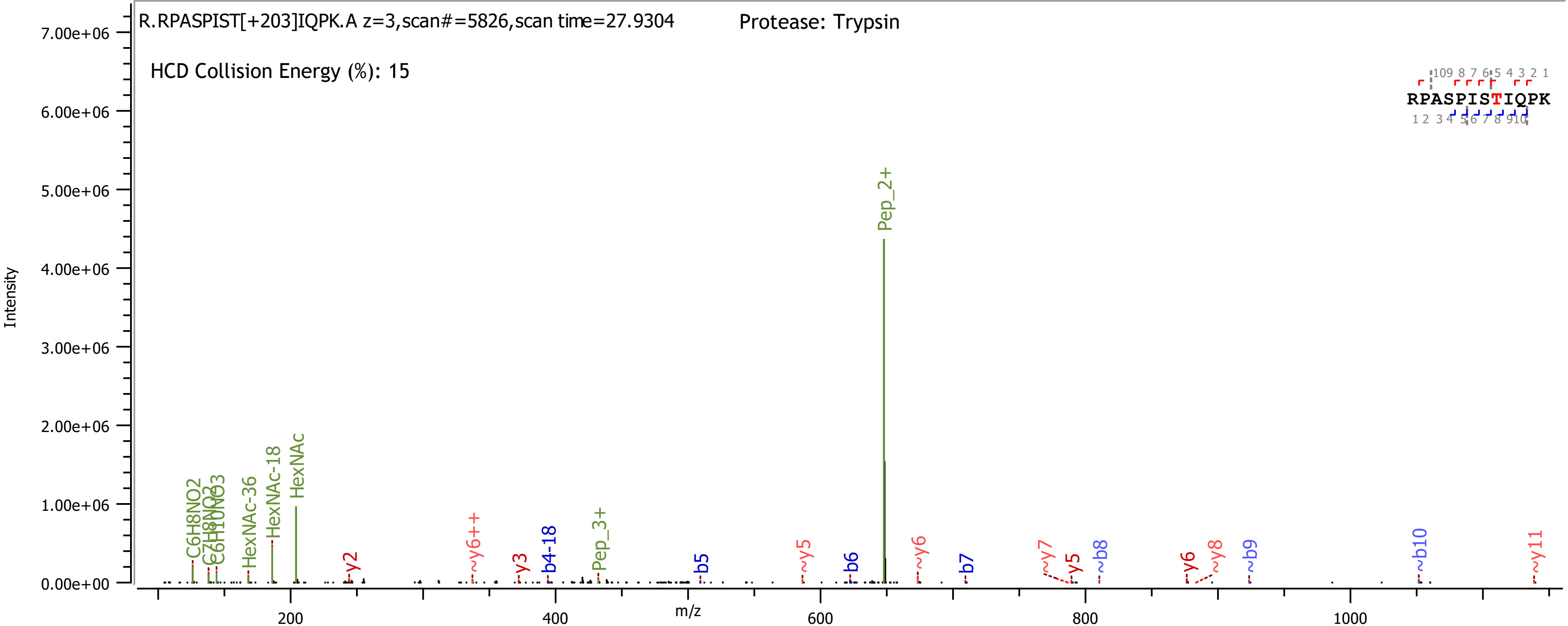

R.RPASPIST[+203]IQPK.A z=3,scan#=5826,scan time=27.9304
Protease: Trypsin
HCD Collision Energy (%): 15
RPASPISTIQPK
Intensity
m/z
Pep_2+
HexNAc
HexNAc-18
HexNAc-36
C6H8NO2
Pep_3+
~y6++
y3
b4-18
y2
b5
~y5
b6
~y6
b7
~y7
y5
~b8
y6
~y8
~b9
~b10
~y11

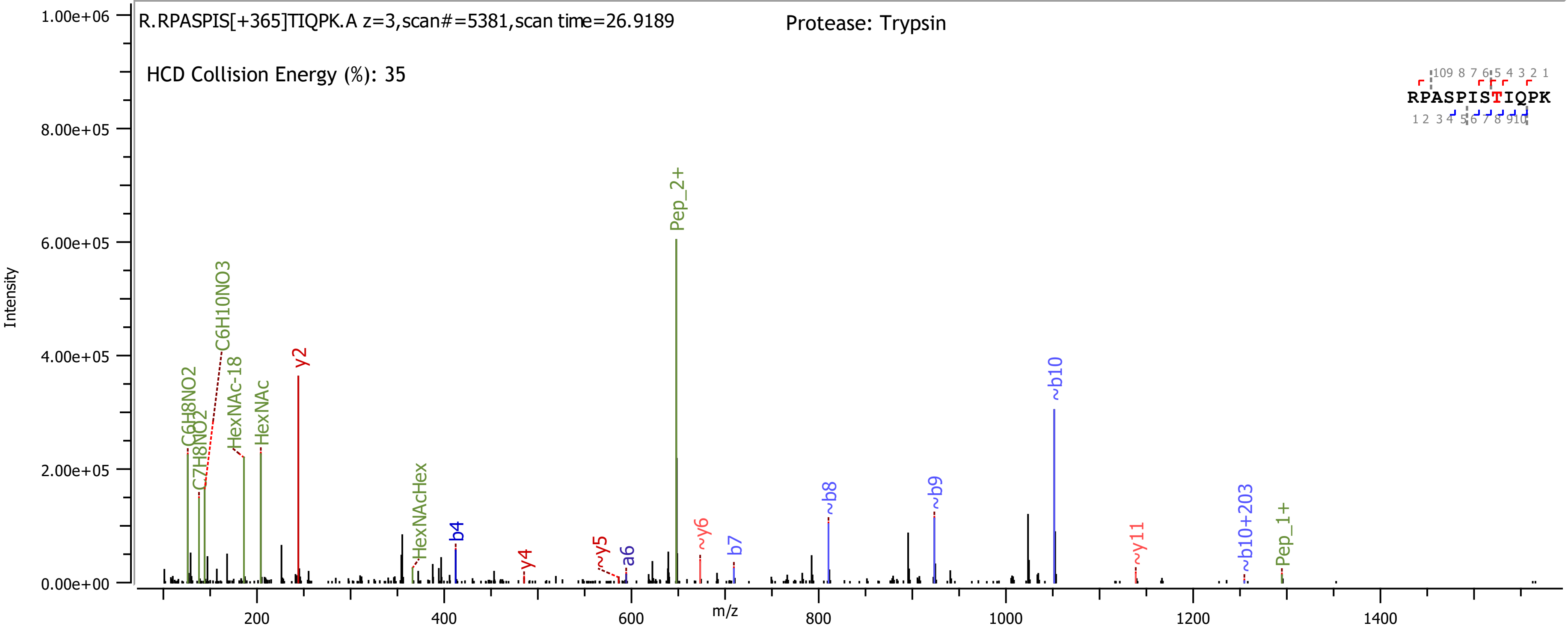

R.RPASPIS[+365]TIQPK.A z=3,scan#=5381,scan time=26.9189
Protease: Trypsin
HCD Collision Energy (%): 35
RPASPISTIQPK
Intensity
m/z
Pep_2+
y2
~b10
C6H10NO3
C6H8NO2
C7H8NO2
HexNAc-18
HexNAc
HexNAcHex
b4
y4
~y5
a6
~y6
b7
~b8
~b9
~y11
~b10+203
Pep_1+

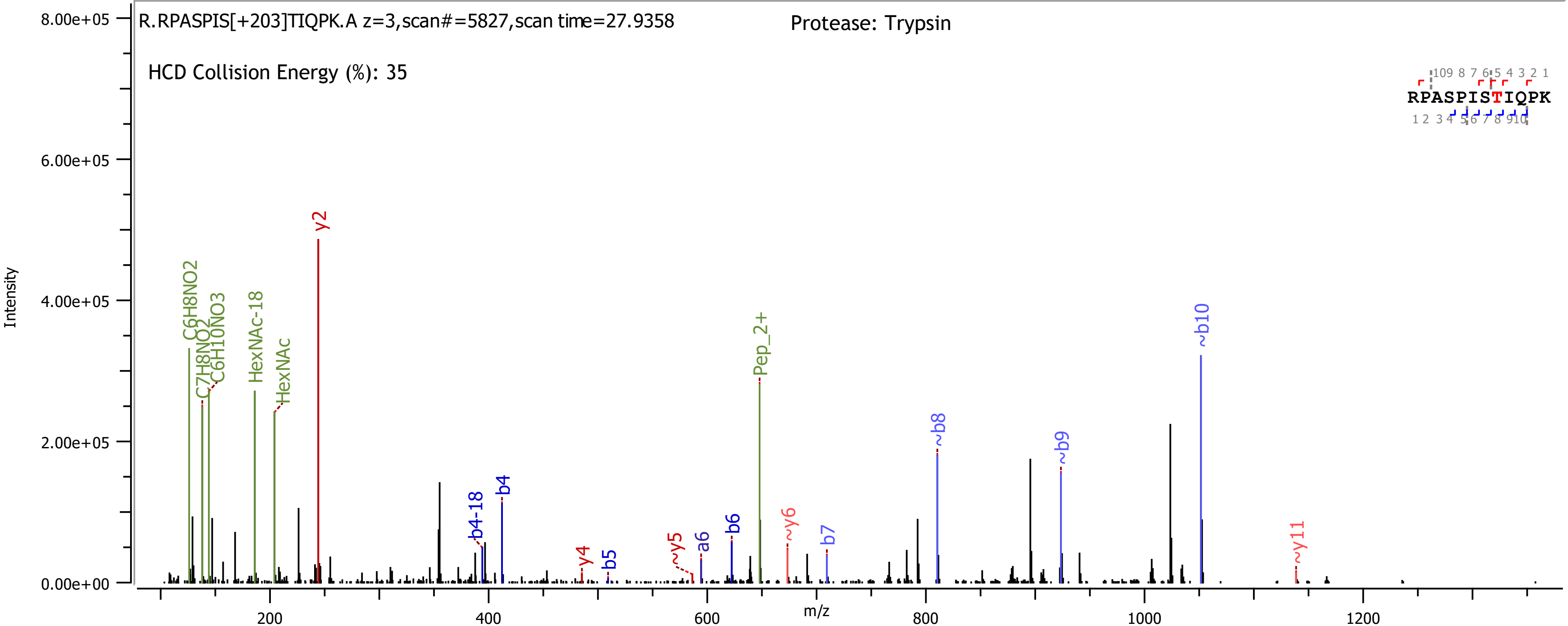

R.RPASPIS[+203]TIQPK.A z=3,scan#=5827,scan time=27.9358
Protease: Trypsin
HCD Collision Energy (%): 35
RPASPISTIQPK
Intensity
m/z
C6H8NO2
C7H8NO2
C6H10NO3
HexNAc-18
HexNAc
y2
b4-18
b4
y4
b5
~y5
a6
b6
Pep_2+
~y6
b7
~b8
~b9
~b10
~y11

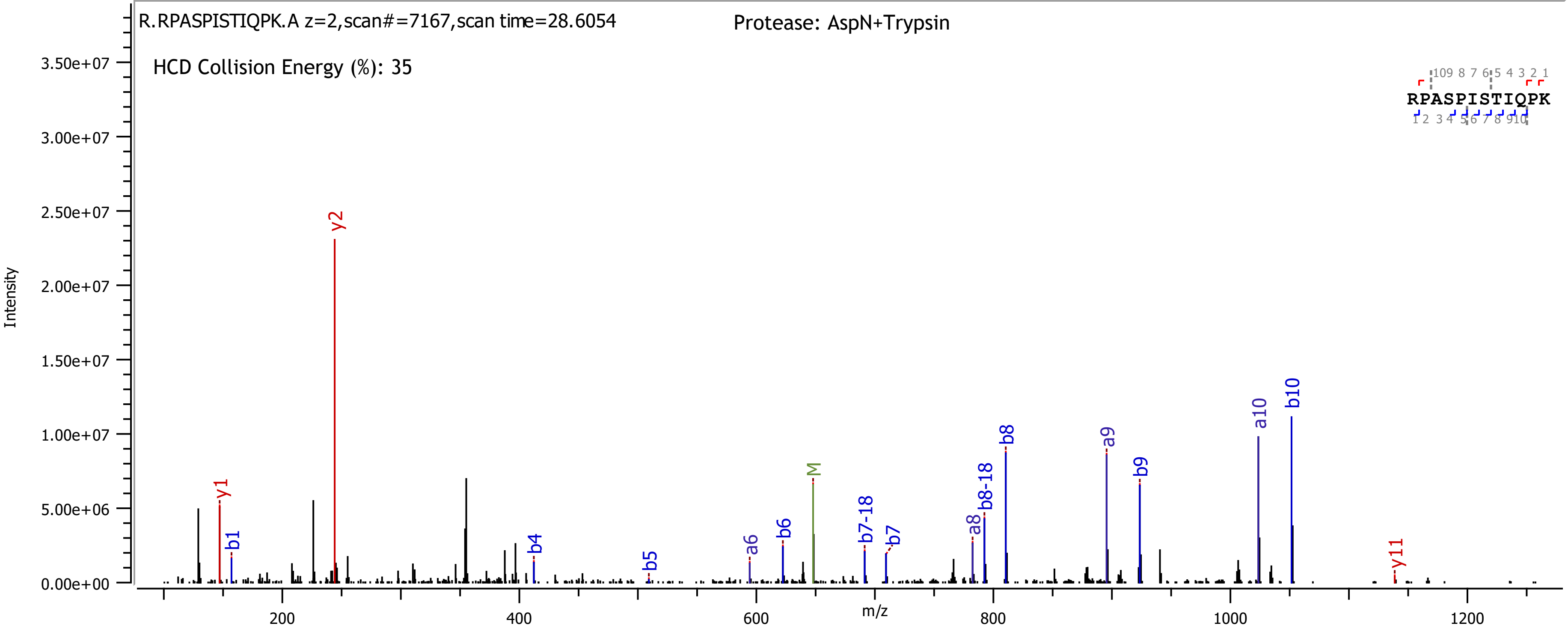

R.RPASPISTIQPK.A z=2,scan#=7167,scan time=28.6054
Protease: AspN+Trypsin
HCD Collision Energy (%): 35
RPASPISTIQPK
Intensity
m/z
y1
b1
y2
b4
b5
a6
b6
M
b7-18
b7
a8
b8-18
b8
a9
b9
a10
b10
y11

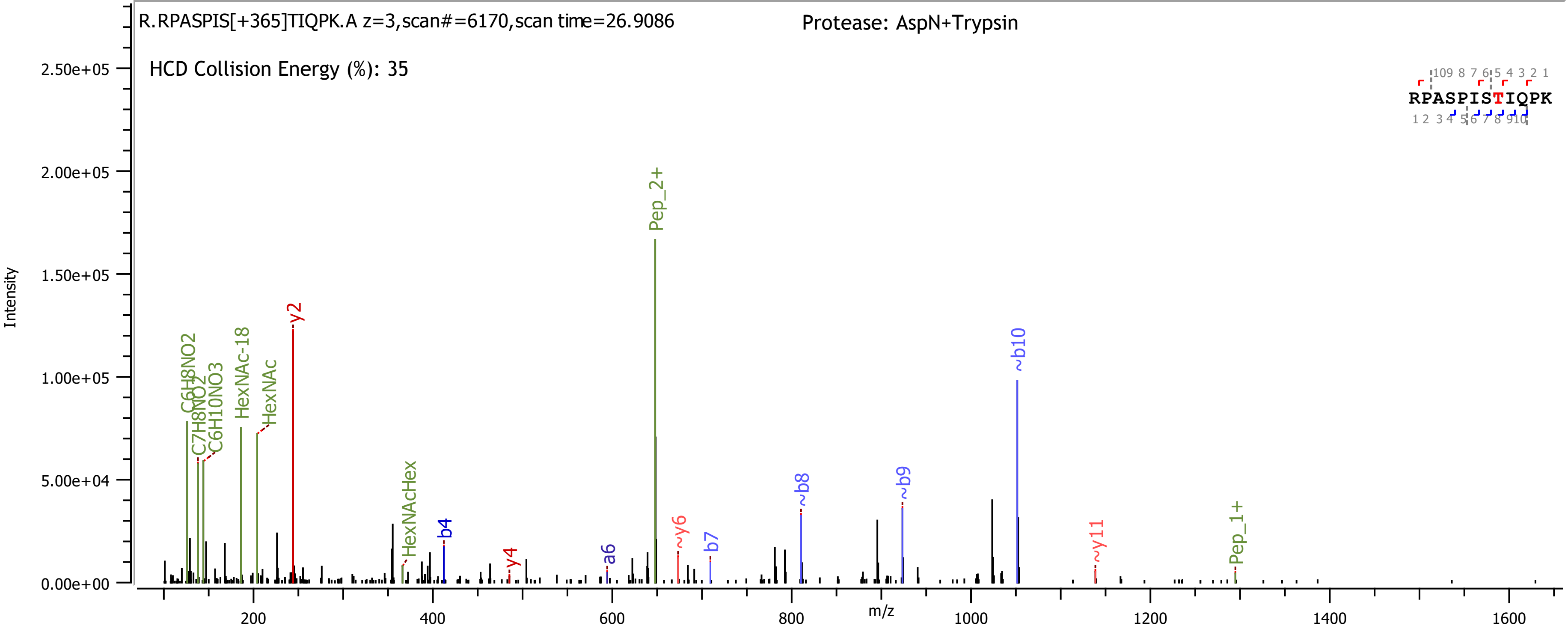
R.RPASPIS[+365]TIQPK.A z=3,scan#=6170,scan time=26.9086
Protease: AspN+Trypsin
HCD Collision Energy (%): 35
RPASPISTIQPK
Intensity
m/z
C6H8NO2
C7H8NO2
C6H10NO3
HexNAc-18
HexNAc
y2
HexNAcHex
b4
y4
a6
Pep_2+
~y6
b7
~b8
~b9
~b10
~y11
Pep_1+
